# A systematic review and meta-analysis of HLA class II associations in patients with IgG4 autoimmunity

**DOI:** 10.1038/s41598-022-13042-2

**Published:** 2022-06-02

**Authors:** Anja Panhuber, Giovanni Lamorte, Veronica Bruno, Hakan Cetin, Wolfgang Bauer, Romana Höftberger, Astrid C. Erber, Florian Frommlet, Inga Koneczny

**Affiliations:** 1 Division of Neuropathology and Neurochemistry, Department of Neurology, Medical University of Vienna, Vienna, Austria; 2 Department of Neurology, Medical University of Vienna, Vienna, Austria; 3 Department of Dermatology, Medical University of Vienna, Vienna, Austria; 4 Department of Epidemiology, Center for Public Health, Medical University of Vienna, Vienna, Austria; 5 Centre for Tropical Medicine and Global Health, Nuffield Department of Medicine, University of Oxford, Oxford, UK; 6 Center for Medical Statistics Informatics and Intelligent Systems, Section for Medical Statistics, Medical University of Vienna, Vienna, Austria

**Keywords:** Autoimmunity, Neuroimmunology

## Abstract

Autoimmune diseases caused by pathogenic IgG4 subclass autoantibodies (IgG4-AID) include diseases like MuSK myasthenia gravis, pemphigus vulgaris or thrombotic thrombocytopenic purpura. Their etiology is still unknown. Polymorphisms in the human leukocyte antigen (HLA) gene locus, particularly in *HLA-DRB1*, are known genetic susceptibility factors for autoimmune diseases. We hypothesized a similar role for HLA polymorphisms in IgG4-AID and conducted a systematic review and meta-analysis with case–control studies on IgG4-AID based on MOOSE/ HuGENet guidelines. Genotype (G) and allele (A) frequencies of HLA-DQB1*05 (G: OR 3.8; 95% CI 2.44–5.9; *p* < 0.00001; A: OR 2.54; 95% CI 1.82–3.55; *p* < 0.00001) and HLA-DRB1*14 (G: OR 4.31; 95% CI 2.82–6.59; *p* < 0.00001; A: OR 4.78; 95% CI 3.52–6.49; *p* < 0.00001) and the HLA-DRB1*14-DQB1*05 haplotype (OR 6.3; 95% CI 3.28–12.09; *p* < 0.00001/OR 4.98; 95% CI 3.8–6.53; *p* < 0.00001) were increased while HLA-DRB1*13 (G: OR 0.48; 95% CI 0.34–0.68; *p* < 0.0001; A: OR 0.46; 95% CI 0.34–0.62; *p* < 0.00001) was decreased in IgG4-AID patients. In conclusion, the HLA-DQB1*05, HLA-DRB1*14 alleles and the HLA-DQB1*05-DRB1*14 haplotype could be genetic risk factors that predispose for the production of pathogenic IgG4 autoantibodies and the HLA-DRB1*13 allele may protect from IgG4 autoimmunity.

## Introduction

IgG4 autoimmune diseases (IgG4-AID) were first collectively described in 2015^1^ and include diseases such as myasthenia gravis with antibodies against muscle-specific kinase (MuSK MG), pemphigus vulgaris (PV) or thrombotic thrombocytopenic purpura (TTP)^2^. IgG4-AID are distinct from other autoantibody-mediated autoimmune diseases, as IgG4 is normally considered as an anti-inflammatory antibody that has structural differences to other IgG subclasses (including functional monovalency) and lacks typical antibody effector mechanisms, such as complement activation^3–6^. IgG4 is thought to play a protective role, e.g. in allergy or autoimmunity, by competing with effector antibodies for epitope binding^3,7–11^. Interestingly, in IgG4-AID the autoantibodies belong predominantly to the IgG4 subclass, and they are directly pathogenic by functional blocking of protein–protein interaction^1,12^. IgG4 pathogenicity could be demonstrated by passive transfer to experimental animals in (1) MuSK MG (MuSK-IgG4), (2) PV (desmoglein 3-IgG4), (3) pemphigus foliaceus (PF, desmoglein 1 and/or 3-IgG4), (4) chronic inflammatory demyelinating polyneuropathy (CIDP, contactin-1-IgG4), (5) CIDP (neurofascin 155-IgG4), and (6) TTP (ADAMTS13-IgG4)^13^. Notably, IgG4-AID differ from clinically distinct IgG4-related diseases^14^ that are therefore not part of our study. IgG4-AID share also further important pathophysiological and therapeutic commonalities^15–17^ including severe disease course, low disease prevalence (equal or less than 5/10,000) and good response to B-cell depletion therapy with rituximab^17^.

Whether IgG4-AID have distinct genetic risk factors that may predispose for the production of pathogenic IgG4 is unknown. A major contributor to genetic susceptibility to autoimmunity are the highly polymorphic human leukocyte antigen (HLA) genes on chromosome 6p21.3 that encode the major histocompatibility complex (MHC)^18,19^. *HLA-DR, HLA-DQ*, and *HLA-DP* encode the MHC II molecules on antigen-presenting cells and thymic epithelial cells that present self- and foreign antigen peptides to CD4 + T helper cells, which is essential for T-cell activation or the development and maintenance of tolerance^20,21^.

*HLA-DR* has been linked to aberrant presentation of self-peptide to autoreactive T helper cells in the thymus^22^, and genetic polymorphisms in the *HLA-DRB1* gene are associated with a range of autoimmune diseases, such as rheumatoid arthritis, diabetes mellitus type I or systemic lupus erythematosus^23^. There is also evidence that the HLA can influence the production of IgG4: distinct HLA variants were shown to determine the immune response towards autoimmunity or tolerance in animal models^24,25^ by directly affecting T-cell fate towards conventional (Tconv) or regulatory T cells (Tregs), and production of pro- or anti-inflammatory cytokines, including interleukin-10 (IL-10). IL-10, which is in part produced by Tregs^26^, induces activation, IgG4 class switch and antibody production in naïve CD40-primed B cells and is therefore a key regulator of IgG4 production^26–31^. Increased IgG4 production was linked to HLA-DRB1*15 in patients with IgG4-related disease^32^and MuSK MG patients carrying HLA-DRB1*14 expressed elevated levels of IL-10 and MuSK antibodies compared to patients with other *HLA* variants^33^. Furthermore, IL-10 was found to be elevated in patients with pemphigus^34,35^, MuSK-MG^36^ and thrombotic thrombocytopenic purpura^37^. This suggests a link between HLA polymorphisms and production of IgG4 via IL-10^29–31,38^. In a previous review, we observed that individual IgG4-AID were frequently reported to be associated with the same recurrent HLA alleles: HLA*DRB1* 04, 11, 14 or 15, and/or HLA-DQB1*05^15^. GWAS data also suggests that HLA class II gene polymorphisms play a role for susceptibility to several different IgG4-AID^15^, and specifically the *HLA-DRB1* and *DQB1* loci were associated with individual diseases^39,40^. We hypothesized that distinct HLA variants may contribute to a genetic susceptibility resulting in a predominant production of IgG4 subclass antibodies and may therefore be associated with several distinct IgG4-AID. Therefore, we wanted to investigate HLA associations first in individual IgG4-AID to identify disease- specific variants, and then across diseases to identify which HLA variants are shared among different IgG4 associated diseases that may predispose to developing pathogenic IgG4 autoantibodies. To this end, we conducted a systematic review and meta-analysis of case–control studies reporting HLA class II associations in individual IgG4-AID.

We found that patients with IgG4-AID had significantly increased frequencies of the HLA-DQB1*05 and HLA-DRB1*14 alleles and the HLA-DRB1*14-DQB1*05 haplotype, and a significant negative association with HLA-DRB1*13. Notably, HLA-DQB1*05 is not positively associated with classical autoimmunity and could be a genetic risk factor for the production of IgG4 subclass autoantibodies.

## Methods

The systematic review was based on recommendations by the HuGENet™ HuGE Review Handbook, version 1.0 (released by the EQUATOR network, 2015^41^), and MOOSE guidelines for Meta-Analyses and Systematic Reviews of Observational Studies^42^.

### Study design

The protocol, including the research question, search strategy, inclusion/exclusion criteria, data to be extracted, and the planned statistical analysis and bias assessment, was designed at the start of the study. The research question was developed with guidance from the PICOS (PI(E)CO) method^43^. The population (P) was defined as the participants in case–control studies and the intervention/exposure (I/E) was defined as the presence of distinct HLA alleles. The comparators (C) were the participants (patients and controls) without the distinct HLA allele and the outcome (O) was the occurrence of one of the six class I IgG4 AID. Regarding the study design, only case–control studies were considered, due to the rare nature of the disease. Only case–control studies with patients with IgG4-AID of class I (MuSK MG, PV, PF, TTP and CIDP with autoantibodies against NF155 or CNTN1^13^) and ethnically, age- and gender-matched controls were included in the study.

### Search strategy

Three individual researchers (A.P., G.L. and V.B.) used electronic search of 34 bibliographic databases and archives (supplementary Table S1), including PubMed/MEDLINE, Cochrane CENTRAL and Cochrane CDSR, Web of Science (core collection and all databases), BIOSIS, Scopus, Ovid Global Health, clinical trial registries (ClinicalTrials.gov and WHO ICTRP), and databases of systematic reviews (Epistemonikos, PROSPERO), BioOne, Centre for Reviews and Dissemination, CINAHL, DOAJ, EMBASE, EU Clinical Trials Register, GlaxoSmithKline's Clinical Study Register, Godort, HSRProj, JSTOR, Mendeley, metaRegister of Controlled Trials (Current controlled trials), Research gate, Science Citation Index (ISI), Science direct, TRIP Database, U.S. Government Documents, Worldcat, Biorxiv and Medrxiv as well as using other sources including grey literature (open grey) and hand searching. The search strategy included the search for key words, MeSH terms, including the use of a truncation operator (*, e.g. “antibod*” to identify the terms “antibody” and “antibodies”), and misspelling including the terms “HLA,” “human leucocyte antigen”, “DRB1”, “DQB1”,“MuSK myasthenia gravis”, “pemphigus”, “thrombotic thrombocytopenic purpura”, “ CIDP”, “chronic inflammatory demyelinating polyneuropathy”, “MuSK”, “Desmoglein 3”, “Desmoglein 1”, “blistering skin disease”, “ADAMTS13”, Neurofascin 155”, “NF155”, “Contactin-1”, “CNTN1″ and related terms in titles and abstracts and full text using Boolean search strategies. The search was conducted between May 5, 2020 until June 16, 2020. As only a limited number of studies investigating HLA associations in IgG4-AID exist, no restrictions for the date of publication were made and all studies available at the time of the search were included. Due to the language proficiency of the researchers, studies in German, English, Italian and Spanish were considered, if applicable using wildcards (*) for the search terms.

### Screening and study selection

After deduplication, three researchers screened the obtained records for eligibility independently of each other, based on inclusion/exclusion criteria in two phases (first phase: screening of title/abstract, second phase: screening of the full text) using Rayyan software^44^. The inclusion criteria for selecting the studies were as follows:

- Studies in humans with a case-control design that
- Reported the association of gene variants of the HLA class II gene locus, including allele, genotype or haplotype frequency.
- Studies in which cases were patients with class I IgG4-AID that were tested positive for the corresponding antibodies ((MuSK myasthenia gravis (antibodies to MuSK) pemphigus vulgaris (antibodies to desmoglein 3), pemphigus foliaceus (antibodies to desmoglein 1 and/or 3), peripheral neuropathies, including CIDP, (antibodies against Contactin 1), peripheral neuropathies, including CIDP, (antibodies against Neurofascin 155), thrombotic thrombocytopenic purpura (antibodies against ADAMTS13)) by standardized laboratory tests, including the following tests: ELISA, cell-based assay (CBA), radioimmuno(precipitation)assay (RIA), direct or indirect immunofluorescence test.
- Studies with a minimum of 1 control per case, that were age and gender matched, and that described the controls as either ethnically matched or with controls from the same geographical region as the patient cohort.
- Studies with controls that are either healthy individuals or patients with a different type of the same disease that were negative for their relevant autoantibodies, as well as any other IgG4 associated autoantibody or with an unrelated disease.

The exclusion criteria were as follows:

f. Studies in which the controls had any immunodeficiencies/abnormalities in the HLA locus,
g. Studies in which data on an individual patient level was not available,
h. Studies including subjects already included in other published studies,
i. Studies where a full text version was not available and
j. Studies where insufficient data was available to calculate an odds ratio.

During the two screening phases, the three researchers were blinded to each other’s decisions in order to prevent bias. Any discrepancies in the assessment after unblinding were resolved via discussion. The search and selection of studies was documented and visualized with a PRISMA flow chart^45^.

### Data extraction

Data was extracted from tables and running text in the included manuscripts and collected in Excel (Microsoft Office, USA). If data was incomplete, unpublished or unavailable, it was attempted to retrieve the data by contacting the corresponding authors of the study by email. The following information from each included study was extracted: primary author, year of publication, full bibliographic information, demographic information of patients and controls (sex, age), country of study site, type of IgG4-AID and/or type of autoantibody, affected organ, type of control, HLA typing method, sample size, genotype frequency, allele frequency, haplotype frequency, HLA supertype frequency, OR and 95% CI. Combinable data was analysed by meta-analysis for association between HLA alleles (genotype or allele frequency) and class I IgG4-AID individually and collectively.

### Statistics

Combinable data (haplotype, genotype and allele frequencies of HLA class II alleles, analyzed separately) was included in the analysis. To study genetic associations with the HLA class II alleles, we used the dominant genetic model of association to analyze genotype frequencies and the allelic model of association to analyze allele frequencies^46^. Depending on the information provided in the individual studies, haplotype analysis was conducted either with the dominant genetic model of association (based on haplotype frequency, defined as the number of individuals with a specific haplotype out of the number of total individuals (n)), or using the allelic model of association (based on haplotype frequency, defined as the total number of a specific haplotype out of the total number of alleles of all study participants (2n)). Both datasets were analyzed and presented separately.

The combined effect of the included studies (pooled OR) was calculated using the Review Manager ((RevMan) [Computer program] Version 5.4.1 The Cochrane Collaboration, 2020).

Mantel–Haenszel tests for the ORs were performed with a random-effects model for different studies, which was important to address heterogeneity in the studies between diseases, and visualized using forest plots.

The heterogeneity of the included studies was measured using *X*^2^, I^2^ and Tau^2^. The publication bias was inspected by funnel plots. To overcome bias due to the predominance of pemphigus studies, which comprised > 50% of the studies, the analysis was repeated, excluding studies on pemphigus. Uncorrected p values < 0.05 were reported as statistically significant. To correct for multiple testing, we applied a Bonferroni-correction for k = 40 tests (20 marker positions, analyzed either as alleles or genotypes), which leads to a corrected significance level of *p* < 0.00125. Results that were only significant at the uncorrected level are shown in italics.

### Consent for publication

All authors declare their consent for publication.

## Results

### Number and characteristics of included studies

**Figure 1:**
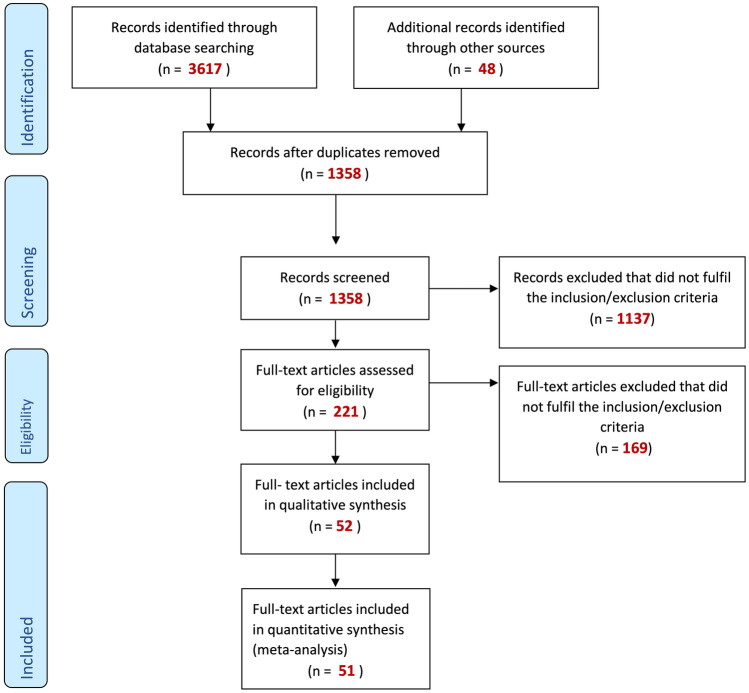
PRISMA flow chart of study identification and eligibility screening. For specific inclusion and exclusion criteria see methods. Figure modified from Moher et al.^47^.

After search and screening, 52 full-text articles with a total of 64 datasets (Tables 1,2) were included in the qualitative synthesis and 51 full-text articles with 62 datasets in the quantitative synthesis (Fig. 1). The following number of studies was identified: 36 on pemphigus, seven on TTP, five on MuSK MG, three on CIDP. Allele, genotype or haplotype frequencies were extracted and analyzed separately.

**Table 1:** Characteristics of included studies.

First author	Year	Country	Disease	Number of patients	Number of controls	HLA alleles (list of reported alleles)	HLA typing method	Genotype, Allele or haplotype frequency	Reference
Dere	2020	Turkey	PV	30	30	DRB1*01, *03, *04, *07, *08, *11, *12, *13, *14, *15, DQB1*02, *03, *04, *05, *06	PCR-SSP	Genotype frequency	^80^
Ehsan	2015	Iran	MuSK MG	24	200	DRB1*01, *03, *04, *07, *08, *09, *10, *11, *12, *1301, *1302, *1303, *14, *15, *16, DQB1*0201, *0301, *0302, *0303, *05, *0601, *0602, *0603, *0604 DRB1*14-DQA1*0104, DQB1*05, DRB1*15-DQA1*0102-DQB1*0601, DRB1*15-DQA1*0102-DQB1*0602, DRB1*15-DQA1*0103-DQB1*0601, DRB1*16-DQA1*0102-DQB1*05	PCR-SSP	Allele frequency, haplotype frequency	^81^
Alahgholi-Hajibehzad	2013	Turkey	MuSK MG	48	250	DRB1*03, DRB1*14, DRB1*16, DQB1*05 DRB1*14-DQB1*05, DRB1*16-DQB1*05	PCR-SSP	Genotype frequency, haplotype frequency	^82^
Harfouch	2014	Syria	PV	91	270	DRB1*01, *03, *04, *07, *08, *09, *10, *11, *12, *13, *14, *15, *16	PCR-SSP	Genotype frequency	^83^
Gonzalez-Escribano	1998	Spain	PV	26	200	DR1, DR2, DR3, DR4, DR7, DR8, DR9, DR10, DR11, DR12, DR13, DR14	PCR-SSOP, PCR-SSP	Genotype frequency	^84^
Brochado	2016	Brazil	PF	86 (172)	1592 (3184)	DRB1*01:01, DRB1*01:02, DRB1*04:02, DRB1*07:01, DRB1*08:04, DRB1*11:01, DRB1*13:01, DRB1*14:01, DRB1*14:04 DQB1*03:01, *03:02, *05:01, *05:03, *06:02, *06:03 DRB1*14-DQA1*01-DQB1*05, DRB1*16-DQA1*01-DQB1*05	Commercial kits form One Lambda	Allele frequency, haplotype frequency	^85^
			PV	82 (164)	1592 (3184)	DRB1*01:01, *01:02, *04:02, *07:01, *08:04, *11:01, *13:01, *14:01, *14:04 DQB1*03:01, *03:02, *05:01, *05:03, *06:02, *06:03 DRB1*15-DQA1*01-DQB1*06, DRB1*14-DQA1*01-DQB1*05, DRB1*16-DQA1*01-DQB1*05			
Martel	2002	France	PF	31	84	DRB1*01, *03, *04, *07, *08, *09, *10, *11, *12, *13, *14, *15, *16,	PCR-SSO, PCR-SSP, PCR–RFLP	Genotype frequency	^86^
				30	64	DQB1*02, *03:02, *05:03			
Párnická	2013	Slovakia	PV	43 (86)	113 (226)	DRB1*01, *03, *04, *07, *08, *09, *10, *11, *12, *13, *14, *15, *16 DQB1*02, *03, *04, *05, *06 DRB1*14-DQB1*05:03, DRB1*14:54-DQB1*05:03, DRB1*14:04-DQB1*05:03, DRB1*14:05-DQB1*05:03	PCR-SSP	Allele frequency, haplotype frequency	^87^
Nikolic	2014	Serbia	MuSK MG	31 (62)	1992 (3984)	DRB1*01, *03, *04, *07, *08, *09, *10, *11, *12, *13, *14, *15, *16	PCR with sequence-specific oligonucleotides	Genotype frequency, allele frequency, haplotype frequency	^78^
				31 (62)	159 (318)	DQB1*02, *03, *04, *05, *06 DRB1*14-DQB1*05, DRB1*16-DQB1*05, DRB1*15-DQB1*06			
De Sena Nogueira Maehara	2017	Brazil	Fogo selvagem	42 (84)	478 (956)	DRB1*02, *04, *05, *07, *16	PCR-SSO	Allele frequency	^88^
		Netherlands	PF	17 (34)	447 (894)	DRB1*04			
Coppo	2010	France	TTP	61	172	DRB1*01, *03, *04, *07, *08, *09, *10, *11, *12, *13, *14, *15, *16	PCR-SSO	Genotype frequency	^89^
				60	172	DQB1*02, *03, *04, *05, *06			
Kanai	2016	Japan	MuSK MG	14	100	DRB1*01, *04, *08, *09, *11, *12, *13, *14, *15, 16 DQB1*03, *04, *05, *06	PCR-SSP	Genotype frequency, haplotype frequency	^90^
Piccinelli	2019	Italy	CIDP	24 (48)	216 (432)	DRB1*01, *03, *04, *07, *08, *10, *11, *13, *14, *15, *16 DQB1*02, *03, *04, *05, *06 DRB1*15-DQB1*06	PCR-SSP	Allele frequency, haplotype frequency	^91^
Zivanovic	2016	Serbia	PV	72 (144)	1992 (3984)	DRB1*01, *03, *04, *07, *11, *12, *13, *14, *15, *16	PCR-SSP	Allele frequency, haplotype frequency	^79^
				72 (144)	159 (318)	DQB1*02, *03, *04, *05, *06 DRB1*14-DQB1*05, DRB1*16-DQB1*05, DRB1*15-DQB1*06			
Saha	2019	UK	PF (Caucasian white British)	25 (50)	100 (200)	DRB1*01, *04, *14 DQB1*0302, *0501, *0502, *0503	PCR-SSP	Allele frequency	^92^
			PF (Indo-Asians)	10 (20)	59 (118)	DRB1*01, *04, *14 DQB1*0302, *0501, *0503			
Gil	2017	Brazil	PV	102	594	DRB1*01, *03, *04, *07, *08, *09, *10, *11, *12, *13, *14, *15, *16 DQB1*02, *03, *04, *05, *06	PCR-SSP	Genotype frequency	^93^
Torzecka	2003	Poland	PF	15 (30)	152 (304)	DRB1*01, *03, *04, *07, *08, *09, *10 *11, *12, *13, *14, *15	Dynal RELI SSO HLA-DRB Test	Allele frequency	^74^
			PV	38 (76)	152 (304)	DRB1*01, *03, *04, *07, *08, *09, *10 *11, *12, *13, *14, *15			
Abida	2009	Tunisia	PF	90 (180)	270 (540)	DRB1*03, *04, *11, *13, *15 DQB1*0301, *0302, *06	PCR-SSP	Allele frequency	^94^
Ogata	2020	Japan	CIDP	22 (44)	418 (836)	DRB1*01, *03, *04, *08, *09, *10, *11, *12, *13, *14, *15, *16 DQB1*02, *03, *04, *05, *06 DRB1*14:05-DQB1*05:03, DRB1*15:01-DQB1*06:02, DRB1*15:02-DQB1*06:01	Next-generation sequencing	Allele frequency, haplotype frequency	^95^
Priyadarshini	2018	India	PV	50	50	DRB1*01, *03, *04, *07, *08, *09, *10, *11, *12, *13, *14, *15, *16 DQB1*02, *03, *04, *05, *06 DRB1*14-DQB1*05, DRB1*15-DQB1*06	PCR-SSOP	Genotype frequency, haplotype frequency	^96^
Tunca	2010	Turkey	PV	25	113	DRB1*01, *03, *04, *05, *06, *07, *08, *09, *10, *11, *12, *13, *14, *15, *16 DQB1*02, *03, *04, *05, *06	PCR-SSP	Genotype frequency	^97^
Haase	2015	Germany	PV (German)	46 (92)	74 (148)	DRB1*01, *02, *03, *04, *07, *08, *09, *10, *11, *12, *13, *14, *15, *16	PCR-SSP	Allele frequency	^98^
			PV (Egypt)	47 (94)	73 (146)				
Martinez-Martinez	2017	Spain	CIDP	13	941	DRB1*15	DNA sequence analysis and SSP methodology	Genotype frequency, Haplotype frequency	^99^
				13	35	DRB1*15-DQB1*06			
Glorio	1999	Argentina	PV	30	199	DR3, DR4, DR8, DR14, DR15 DQB1*03, *05	PCR-SSO	Genotype frequency	^100^
Pavoni	2003	Brazil	Fogo selvagem	128	402	DRB1*01, *03, *04, 07, *08, *09, *10, *11, *12, *13, *14, *15, *16	PCR-SSOP	Genotype frequency	^101^
Thomas	1998	Spain	PV	26	200	DR1, DR2, DR3, DR4, DR7, DR8, DR9, DR10, DR11, DR12, DR13, DR14	PCR-SSOP, PCR-SSP	Genotype frequency	^102^
Zhang	2019	China	PF	72	501	DRB1*01, *03, *04, *07, *08, *09, *10, *11, *12, *13, *14, *15, *16 DQB1*02, *03, *04, *05, *06	Affymetrix axiom precision medicine research array based GWAS	Genotype frequency, allele frequency	^75^
			PV	255	501				
Moraes	1991	Brazil	Fogo selvagem	37	49	DR1, DR16, DQ2	PCR-SSP	Genotype frequency	^103^
				38	46	DRB1*15			
				38	41	DQB1*06			
Lee	1998	South Korea	PF	15	100	DRB1*01, *03, *04, *07, *08, *09, *10, *11, *12, *13, *14, *15, *16 DQB1*02, *03, *04, *05, *06	PCR-SSP	Genotype frequency	^76^
			PV	15	100				
Shams	2008	Iran	PV	52 (104)	180 (360)	DRB1*01, *03, *04, *07, *08, *09, *10, *11, *12, *13, *14, *15, *16 DQB1*02, *03, *04, *05, *06 DRB1*15-DQA1*01-DQB1*06 DRB1*16-DQA1*01, DQB1*05	PCR-SSP	Allele frequency, haplotype frequency	^104^
Orouji	2014	Mid-east origin	PV	54	85	DQB1*02, *03, *04, *05, *06	PCR-SSP	Genotype frequency, allele frequency	^105^
Miyagawa	1997	Japan	PF	9	525	DRB1*04, *14 DQB1*03, *05 DRB1*14-DQA1*01-DQB1*05	NA	Genotype frequency, haplotype frequency	^77^
			PV	7	525				
Joly †	2020	France	TTP	26	172	DRB1*04, *11 DQB1*03	PCR-SSP	Genotype frequency, allele frequency	^106^
Glorio	2002	Argentina	PV	47	199	DR3, DR4, DR8, DR14, DR15 DQB1*03, *05	PCR-SSO	Genotype frequency	^107^
Sakai	2020	Japan	TTP	52 (104)	523 (1046)	DRB1*01, *04, *08, *09, *11, *12, *13, *14, *15 DQB1*03, *04, *05, *06	Commercial kits with Illumina MiSeq technology	Allele frequency	^108^
Koc	2012	Turkey	Pemphigus	60	60	DRB1*04, *11, *14 DQB1*02, *05, *06	PCR-SSP	Genotype frequency	^109^
Scully	2010	UK	TTP	50	200	DRB1*01, *03, *04, *07, *08, *09, *10, *11, *12, *13, *14, *15, *16 DQB1*02, *03, *04, *05, *06	PCR-SSP, PCR-SSOP	Genotype frequency	^110^
Mobini	1997	Iran	PV	38	57	DRB1*14-DRB3*02-DQB1*05-DQA1*01, DRB1*15-DRB5*01-DQB1*06-DQA1*01, DRB1*15-DRB5*01-DQB1*06-DQA1*05	PCR-SSOP	Haplotype frequency	^111^
Delgado	1997	Pakistan	PV (Pakistan)	19 (38)	13 (26)	DRB1*01, *02, *03, *04, *07, *10, *11, *13, *14 DQB1*02, *03, *04, *05, *06	PCR-SSOP	Allele frequency	^112^
		Europe	PV (Europe)	19 (38)	248 (496)	DRB1*01, *02, *04, *07, *08, *11, *12, *13, *14 DQB1*02, *03, *05, *06			
Yamashina	1998	Japan	PV	17	525	DRB1*04, *14 DQB1*03, *05	PCR–RFLP	Genotype frequency, haplotype frequency	^113^
Al Haddad	2019	Lebanon	TTP	30	30	DRB1*01, *03, *04, *07, *08, *09, *10, *11, *12, *13, *14, *15, *16 DQB1*02, *03, *04, *05, *06	PCR-SSP	Genotype frequency	^114^
John	2011	Germany	TTP	54	11,407	DRB1*01, *03, *04, *07, *08 *09, *11, *12, *13, *14	PCR-SSP, PCR-SSO	Genotype frequency	^115^
				50	174	DQB1*02			
				54	174	DQB1*04, *05			
				47	174	DQB1*03			
				52	174	DQB1*06			
Martino	2016	France	TTP	26 (52)	663 (1326)	DRB1*04, DRB1*11	PCR-SSO	Allele frequency	^116^
				26 (52)	437 (874)	DQB1*03			
				24 (48)	100 (200)	DRB1*04, DRB1*11 DQB1*03			
Cerna	1993	Brazil	Fogo selvagem	10	74	DRB1*04, *08, *14, *16 DQB1*03, *04	PCR-SSOP	Genotype frequency	^117^
Birol	2002	Turkey	Pemphigus	33	100	DR4, DR11, DR14 DQ2, DQ4	Microdroplet lymphocyte test	Genotype frequency	^118^
Rangel-Gamboa	2015	Mexico	PV	43 (86)	99 (198)	DR1, DR4, DR7, DR8, DR11, DR13, DR14, DR16	PCR-SSO	Allele frequency	^119^
Khan	2015	Pakistan	PV	28	150	DRB1*01, *03, *04, *07, *08, *09, *10, *11, *12, *13, *14, *15, *16	PCR-SSP	Genotype frequency	^120^
Carcassi	1996	Italy	PV (Sardinians)	16 (32)	91 (182)	DRB1*03, *04, *08, *14 DQB1*02, *03, *05	PCR-SSO	Allele frequency	^121^
			PV (Italians)	16 (32)	284 (568)	DRB1*03, *04, *08, *14			
				16 (32)	406 (812)	DQB1*02, *03, *05			
Lombardi	1996	Italy	PV	33	102	DRB1*01, *03, *04, *07, *08, *11, *13, *14, *15, *16 DQB1*02, *03, *04, *05, *06	PCR-SSO	Genotype frequency	^122^
Saha	2010	UK	PV (white Europeans)	96 (192)	100 (200)	DRB1*03, *04, *07, *14, *15 DQB1*02, *03, *05, *06	PCR-SSP	Allele frequency	^123^
			PV (Indo-Asians)	57 (114)	59 (118)				
Niks	2006	Netherlands	MuSK MG	23	2440	DR1, DR3, DR14, DR16, DQ5, DQ6	PCR-amplified fragments and biotin labelled oligonucleotides	Genotype frequency, Haplotype frequency	^124^
				23 (46)	321 (642)	DRB1*14-DQB1*05, DRB1*15-DQB1*05			
Sinkovits	2017	Hungary	TTP	75	204	DRB1*01, *03, *04, *07, *08, *09, *10, *11, *12, *13, *14, *15, *16	PCR-SSO	Genotype frequency, Haplotype frequency	^125^
				75	162	DRB1*14-DQB1*05, DRB1*16-DQB1*05, DRB1*15-DQB1*06			

*PV* pemphigus vulgaris, *PF* pemphigus foliaceus, *CIDP* chronic inflammatory demyelinating polyneuropathy, *TTP* thrombotic thrombocytopenic purpura, *MuSK MG* muscle-specific kinase myasthenia gravis, *PCR* polymerase chain reaction, *SSP* sequence specific primer, *SSO(P)* sequence specific oligonucleotides (probes), *RFLP*, restriction fragment length polymorphism. † Study was included in the qualitative synthesis, but excluded from the meta-analysis as it did not fit all selection criteria.

**Table 2:** HLA class II associations identified in IgG4-AID.

Disease	Positive association	Negative association
Pemphigus	DRB1*04, *14, DQB1*03, *05	DRB1*03, *07, *09, *11, *13, *15 DQB1*02, *06
TTP	DRB1*11, *12, *15	DRB1*04, *13
MuSK myasthenia gravis	DRB1*14, *16 DQB1*05 DRB1*14-DQB1*05, DRB1*16-DQB1*05	*No significant associations*
All IgG4-AID	DRB1*04, *14 DQB1*03, *05 DRB1*14-DQB1*05	DRB1*03, *07, *09, *11, *13, *15 DQB1*02, *06
MuSK, TTP, CIDP combined	DRB1*11, *12, *14, *15, *16 DQB1*05 DRB1*14-DQB1*05	DRB1*04, *13*,*

Bold: significant results only for either genotype or allele frequency.

Due to lack of data on *HLA-DP,* only polymorphisms in the *HLA-DR* and *HLA-DQ* genes were extracted. The following studies and datasets were included in the qualitative synthesis but excluded from the meta-analysis as they did not fit all selection criteria: the study by Joly et al., 2020, and one dataset from the Delgado study (1997).

### Genetic associations with individual IgG4-AID

We wanted to study genetic HLA associations of the individual diseases. Data of 15 *HLA-DRB1* alleles (DRB1*01–16) and five *HLA-DQB1* alleles (DQB1*02–06) could be extracted from studies on pemphigus, MuSK MG and TTP (summarized in Tables 1,2). Due to a lack of data, no separate analysis for CIDP was undertaken.

#### Pemphigus

In a substantial proportion of the studies, there was little to no distinction between pemphigus vulgaris and pemphigus foliaceus. In this study, we therefore analyzed all subtypes of pemphigus collectively (supplementary Figs. S46–S66). The pooled ORs and 95% CIs indicated that four HLA variants were associated with a significantly increased frequency in pemphigus patients: HLA-DRB1*04 (genotype: OR 4.86; 95% CI 3.61–6.54; *p* < 0.00001; allele: OR 4.18; 95% CI 3.14–5.56; *p* < 0.00001), HLA-DRB1*14 (genotype: OR 4.81; 95% CI 2.88–8.05; *p* < 0.00001; allele: OR 6.14; 95% CI 4.98–7.58), HLA-DQB1*03 (genotype: OR 2.77; 95% CI 1.56–4.92; *p* = 0.0005; allele: OR 1.99; 95% CI 1.39–2.83; *p* = 0.0002) and HLA-DQB1*05 (genotype: OR 4.3; 95% CI 2.53–7.28; *p* < 0.00001; allele: OR 3.04; 95% CI 2.10–4.41; *p* < 0.00001). Eight variants were significantly decreased in pemphigus patients, suggesting a protective role: HLA-DRB1*03 (genotype: OR 0.34; 95% CI 0.25–0.47; *p* < 0.00001; allele: OR 0.35; 95% CI 0.17–0.70; *p* = *0.003*), HLA-DRB1*07 (genotype: OR 0.38; 95% CI 0.25–0.58; *p* < 0.00001; allele: OR 0.45; CI 95% 0.32–0.61; *p* < 0.00001), HLA-DRB1*09 (genotype: OR 0.57; 95% CI 0.43–0.77; *p* = 0.0002; allele: OR 0.62; 95% CI 0.47–0.81; *p* = 0.0005), HLA-DRB1*11 (genotype: OR 0.42; 95% CI 0.27–0.65; *p* < 0.0001; allele: OR 0.47; 95% CI 0.31–0.72; *p* = 0.0005), HLA-DRB1*13 (genotype: OR 0.51; 95% CI 0.31–0.82; *p* = *0.006*; allele: OR 0.44; 95% CI 0.32–0.6; *p* < 0.00001), HLA-DRB1 *15 (genotype: OR 0.47; 95% CI 0.37–0.59; *p* < 0.00001; allele: OR 0.37; 95% CI 0.3–0.47; *p* < 0.00001), HLA-DQB1*02 (genotype: OR 0.33; 95% CI 0.24–0.45; *p* < 0.00001; allele: OR 0.4; 95% CI 0.31–0.52; *p* < 0.00001) and HLA-DQB1*06 (genotype: OR 0.48; 95% CI 0.31–0.74; *p* = 0.0009; allele: OR 0.43; 95% CI 0.36–0.53; *p* < 0.00001).

#### Thrombotic thrombocytopenic purpura (TTP)

Seven TTP studies were analyzed, but due to a lack of data, quantitative analysis was only conducted on genotype frequency of alleles with data from at least three studies, and allele frequency was only analyzed qualitatively. We observed significantly increased genotype frequencies of *HLA*-DRB1*11 (genotype: OR 3.38; 95% CI 2.04–5.60; *p* < 0.00001), HLA-DRB1*12 (genotype: OR 2.52; 95% CI 1.32–4.84; *p* = 0.005), HLA-DRB1*15 (genotype: OR 1.67; 95% CI 1.11–2.51; *p* = *0.01*) and significantly decreased genotype frequencies, and a trend towards a reduced allele frequency of HLA-DRB1*04 (genotype: OR 0.38; 95% CI 0.25–0.56; *p* < 0.00001) and HLA-DRB1*13 (genotype: OR 0.43, 95% CI 0.29–0.64; *p* < 0.0001) (supplementary Figs. S67–S84).

#### MuSK Myasthenia gravis

Five studies were available on MuSK MG, and quantitative analysis was only performed for alleles with a minimum of three studies per allele. MuSK MG patients had a strong, significant increase in genotype frequency of HLA-DRB1*14 (genotype: OR 6.36, 95% CI 2.75–14.75, *p* < 0.0001), HLA-DRB1*16 (genotype: OR 5.03, 95% CI 3.16–7.99; *p* < 0.00001) and HLA-DQB1*05 (genotype: OR 7.94, 95% CI 3.44–18.30, *p* < 0.00001). The haplotypes HLA-DRB1*14-DQB1*05 and HLA-DRB1*16-DQB1*05 showed an increased frequency in the two studies that defined the haplotype frequency like the genotype frequency (n), and a significant increase in the three studies that defined the haplotype frequency like the allele frequency (2n, HLA-DRB1*14-DQB1*05: OR: 4.78; 95% CI 2.65–8.62; *p* < 0.00001; HLA-DRB1*16-DQB1*05: OR 3.47, 95% CI 2.16–5.57; *p* < 0.00001). A tendency towards a decreased frequency of HLA-DQB1*06 was observed (supplementary Figs. S85–S105).

### HLA alleles with increased frequency across IgG4-AID

**Figure 2:**
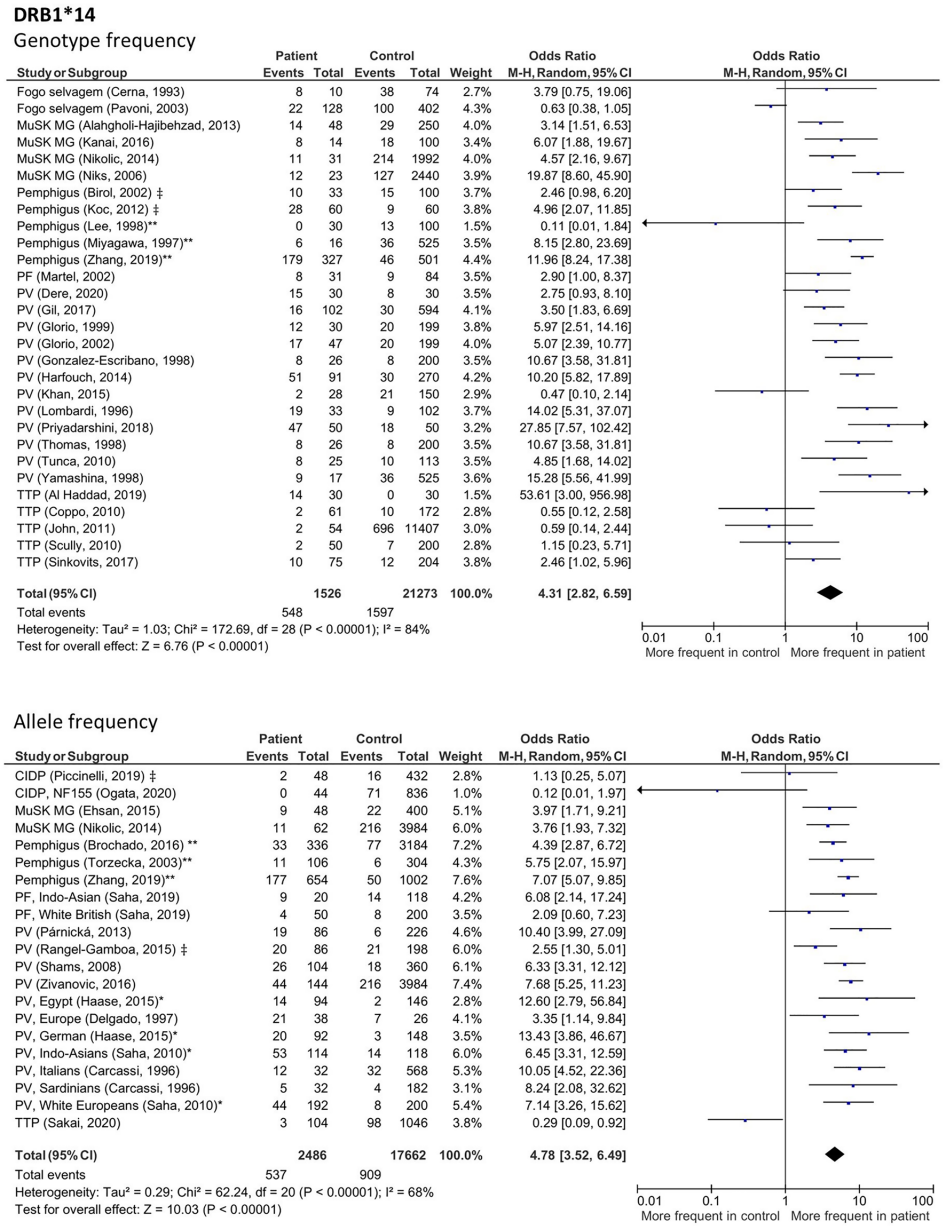
Forest plot depicting allele and genotype frequency of HLA-DRB1*14 in patients with class I IgG4-AID. Cumulative meta-analysis with a random-effects model demonstrated a significant increased frequency in patients compared to controls. ‡ Study did not differentiate between disease subgroups of pemphigus or CIDP. * Study was included after discussion with W.B. **Study in which the same control group was used for pemphigus foliaceus and pemphigus vulgaris, here data was pooled for analysis.

**Figure 3:**
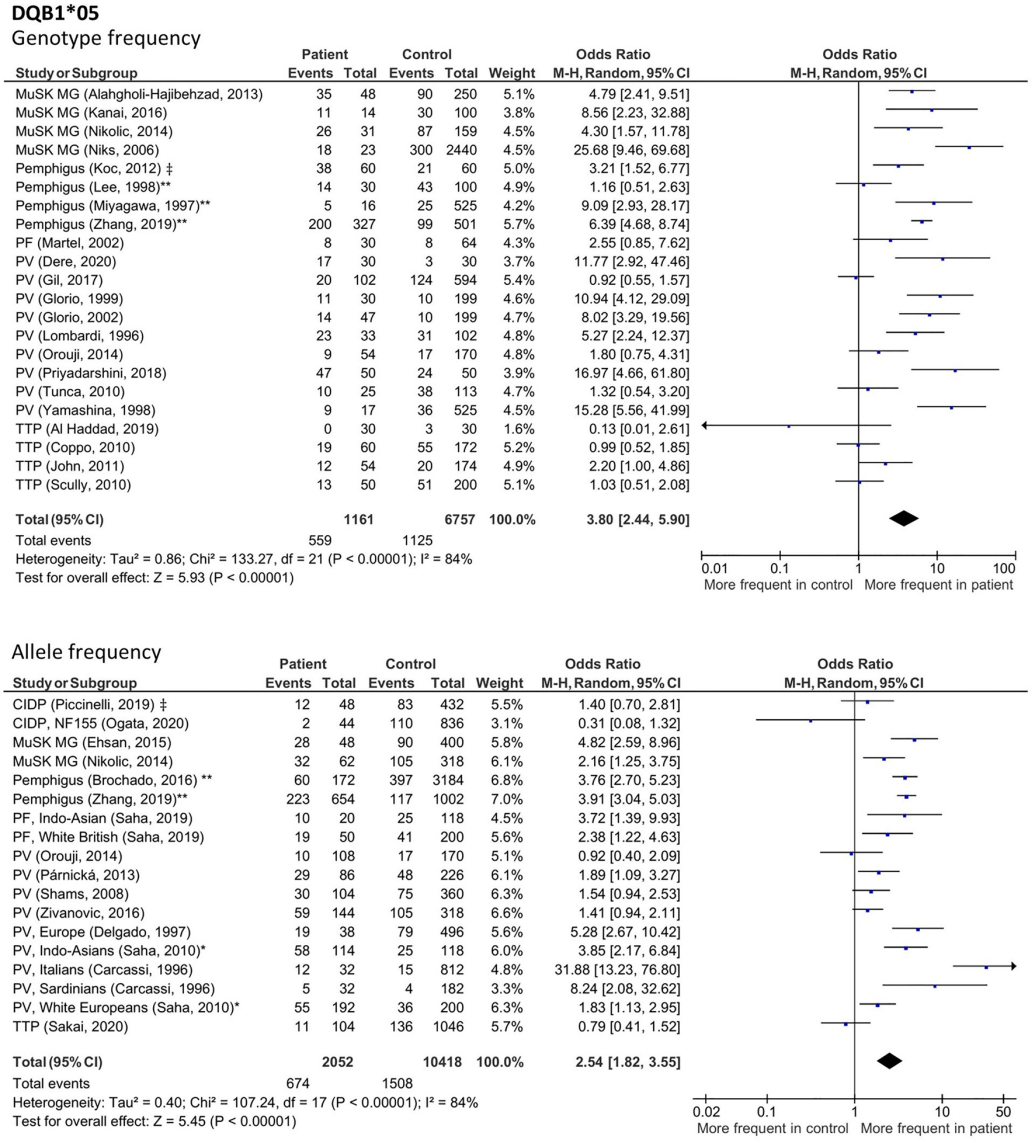
Forest plots of the allele and genotype frequency for HLA-DQB1*05 in patients with class I IgG4-AID. Meta-analysis using a random-effects model demonstrated a significant increased frequency in patients compared to controls. ‡ Study did not differentiate between disease subgroups of pemphigus or CIDP. * Study was included after discussion with W.B. ** Study in which the same control group was used for pemphigus foliaceus and pemphigus vulgaris, here data was pooled for analysis.

**Figure 4:**
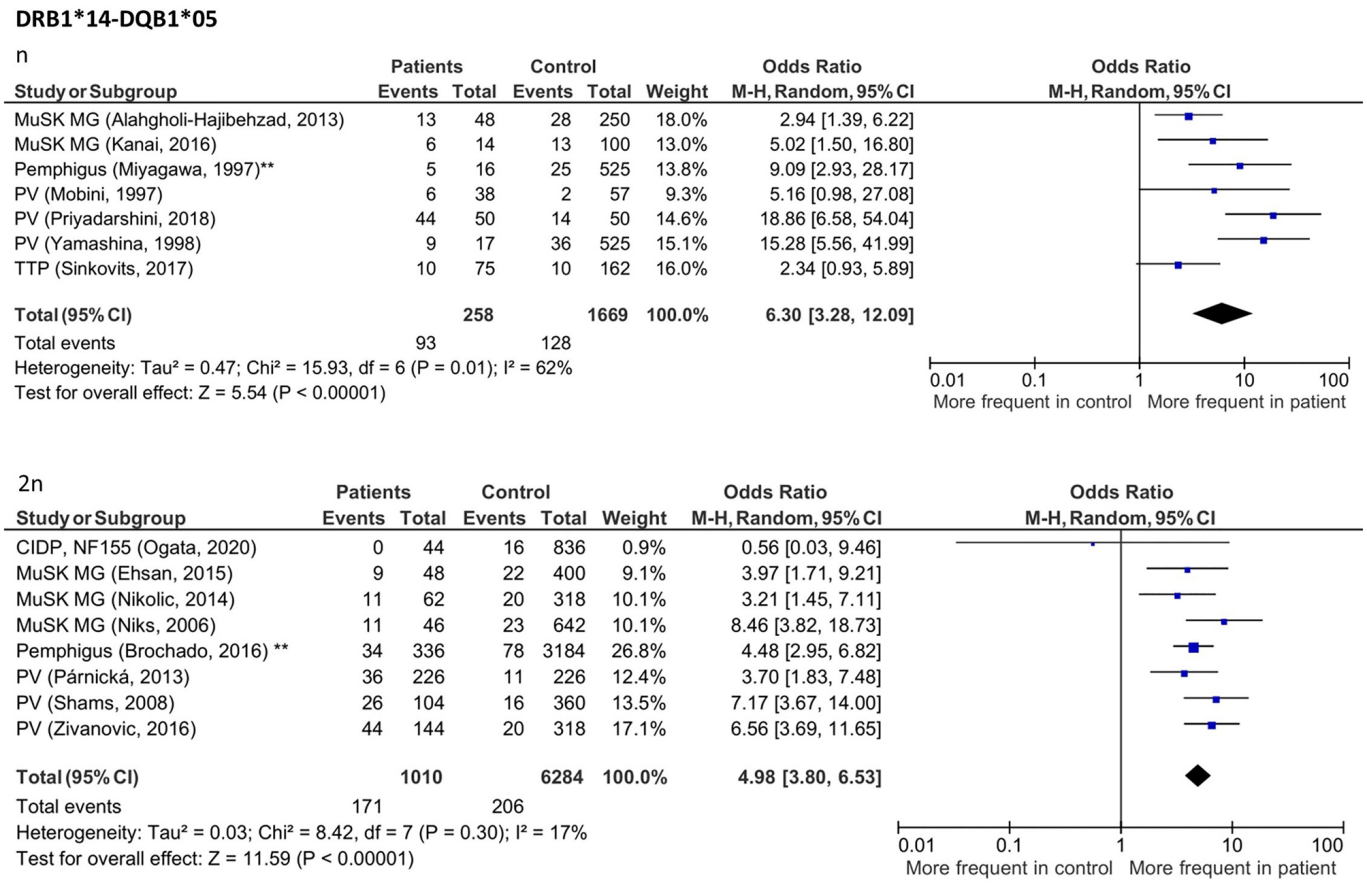
Forest plots of haplotype frequencies for HLA-DRB1*14-DQB1*05 in patients with class I IgG4-AID. Meta-analysis using a random-effects model showed a significant positive association in patients throughout all six diseases. (n): haplotype frequency was defined as the number of individuals with a specific haplotype out of the number of total individuals; (2n): haplotype frequency was defined as the total number of a specific haplotype out of the total number of alleles of all study participants. ** Study in which the same control group was used for pemphigus foliaceus and pemphigus vulgaris, here data was pooled for analysis.

To identify possible genetic risk factors that may be shared across diseases and that may predispose for the development of IgG4 autoantibodies, we analyzed HLA associations in all IgG4 patients (Fig. S19–28). Only figures with results that remained statistically significant after additional analysis (described below) are shown in the main manuscript, the remaining results are shown in the supplementary. We observed increased frequencies of HLA-DRB1*14 (Fig. 2, genotype: OR 4.31; 95% CI 2.82–6.59; *p* < 0.00001; allele: OR 4.78; 95% CI 3.52–6.49; *p* < 0.00001), HLA-DQB1*05 (Fig. 3, genotype: OR 3.8; 95% CI 2.44–5.9; *p* < 0.00001; allele: OR 2.54; 95% CI 1.82–3.55; p < 0.00001) as well as the HLA-DRB1*14-DQB1*05 haplotype (Fig. 4, n: OR 6.3; 95% CI 3.28–12.09; *p* < 0.00001, 2n: OR 4.98; 95% CI 3.8–6.53; *p* < 0.00001). Further associations were found in HLA-DQB1*03 (Fig. S2, genotype: OR 2.53; 95% CI 1.67–3.97; *p* < 0.0001; allele: OR 1.65; 95% CI 1.24–2.19; *p* = 0.0007) and HLA-DRB1*04 (Fig. S1, genotype: OR 2.72; 95% CI 1.81–4.10; *p* < 0.00001; allele: OR 2.72; 95% CI 1.94–3.81, *p* < 0.00001).

Since the predominance of pemphigus studies (36/52 studies) may have skewed the data towards pemphigus-specific risk alleles, the data was re-analyzed after excluding the pemphigus studies to validate the findings (Figs. S3–S7 and S29–S45).

While we could confirm the positive association with HLA-DRB1*14, HLA-DQB1*05 and the HLA-DRB1*14-DQB1*05 haplotype after exclusion of pemphigus patients (Fig. S5–S7), the frequency of HLA-DRB1*04 (Fig. S3) was significantly decreased, suggesting this association is specific for pemphigus. Further positive associations after exclusion of pemphigus were observed in HLA-DRB1*11, *12, *15 and *16 (Fig. S36–S39).

### Reduced frequency of HLA alleles in IgG4-AID

**Figure 5:**
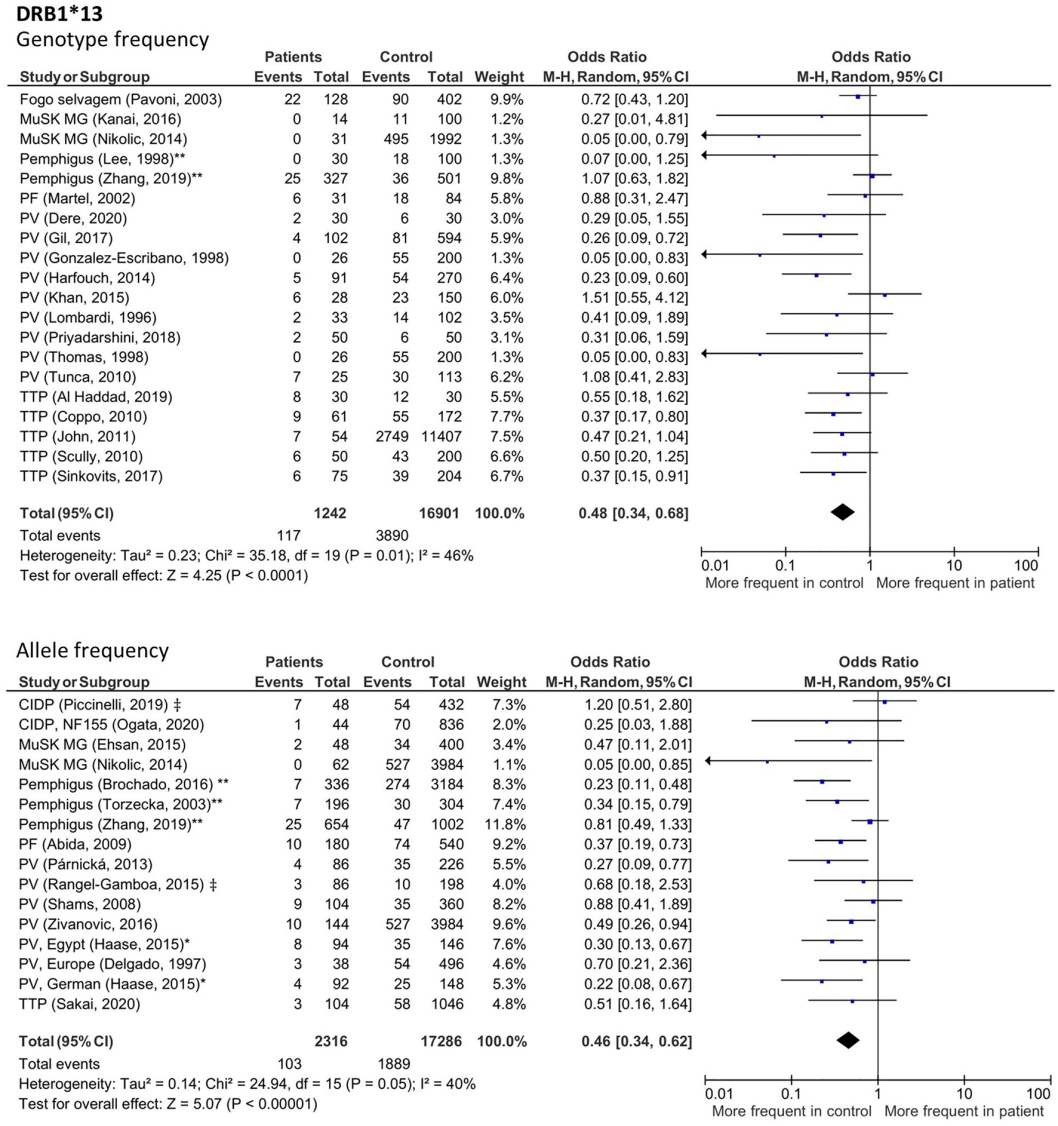
Forest plots of allele and genotype frequency of HLA-DRB1*13 in patients with class I IgG4-AID. Meta-analysis using a random-effects model demonstrated a significant decreased frequency in patients compared to controls. ‡ Study did not differentiate between disease subgroups of pemphigus or CIDP. *Study was included after discussion with W.B. **Study in which the same control group was used for pemphigus foliaceus and pemphigus vulgaris, here data was pooled for analysis.

Several HLA variants were significantly decreased in patients with IgG4-AID, which is interesting as these may potentially contribute to a protection from IgG4 autoimmunity (Figs. 5; Suppl Fig. S8–S12). Reduced frequencies were observed for HLA-DRB1*03 (genotype: OR 0.54; 95% CI 0.35–0.83; *p* = *0.005*; allele: OR 0.46; 95% CI 0.25–0.84; *p* = *0.01*), HLA-DRB1*07 (genotype: OR 0.49; 95% CI 0.34–0.69; p < 0.00001; allele: OR 0.52; 95% CI 0.37–0.74; p = 0.0003), HLA-DRB1*09 (genotype: OR 0.62; 95% CI 0.47–0.82; p = 0.0008; allele: OR 0.70; 95% CI 0.56–0.89; *p* = *0.003*), HLA-DRB1*13 (genotype: OR 0.48; 95% CI 0.34–0.68; p < 0.0001; allele: OR 0.46; 95% CI 0.34–0.62; p < 0.00001), HLA-DQB1*02 (genotype: OR 0.5; 95% CI 0.28–0.89; *p* = *0.02*; allele: OR 0.51; 95% CI 0.36–0.71; p < 0.0001) and HLA-DQB1*06 (genotype: OR 0.61; 95% CI 0.44–0.84; *p* = *0.003*; allele: OR 0.59; 95% CI 0.38–0.9; *p* = *0.01*).

The negative associations were less pronounced, and after exclusion of pemphigus (Figs. S30, S32, S34, S40, S43), only HLA-DRB1*13 (Fig. S4) was found at reduced frequency (genotype: OR 0.41; 95% CI 0.28–0.61; *p* < 0.00001, allele: OR: 0.49, 95% CI 0.20–1.21, *p* = *0.12*).

### Analysis of higher resolution data

We were interested to know whether the association was due to specific alleles, but high-resolution data was only available for a fraction of studies, as most studies only reported one-field resolution data (supplementary Table S3 and S4). We analyzed the available datasets with higher resolution data, which was mostly derived from pemphigus studies. Data of the available variants (Fig. S13–S15) was analyzed and positive associations with HLA-DRB1*14:01, HLA-DRB1*14:04, HLA-DRB1*04:02 and HLA-DQB1*05:03 were observed.

### Within-ancestry analysis

To study the potential effect of ancestry, we conducted a within-ancestry analysis from the three countries with the highest number of datasets (Brazil: 6 studies, Turkey and Japan: each 5 studies) separately (Fig. S16–S18). A trend for similar outcomes could be observed in all three populations where enough data was available, but there was variation in the strength of the association, e.g. the OR for HLA-DRB1*14 was higher in Japan than in Brazil or Turkey. An across-ancestry analysis was not considered feasible with the available data.

### Evaluation of heterogeneity and publication bias

The heterogeneity was assessed by Tau^2^, *X*^2^ and I^2^ tests (supplementary Table S5), whereas potential publication bias was assessed by funnel plots (Figs. 6; Suppl Figs.S106–112).

**Figure 6:**
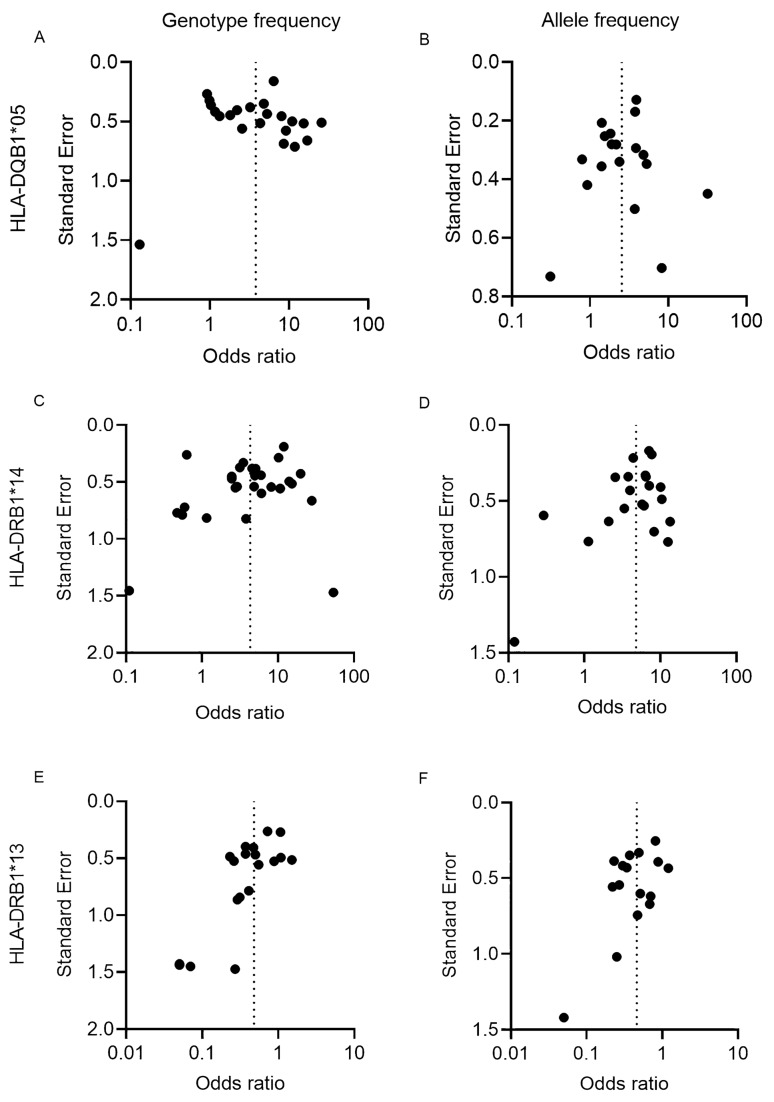
Funnel plot analysis of genotype and allele frequency data for HLA-DQB1*05, DRB1*13 and HLA-DRB1*14 in all IgG4 patients. A funnel plot analysis was undertaken to assess publication bias. Odds ratios were plotted against the standard error and the studies demonstrated symmetrical scattering along the funnel axis (pooled effect estimate from meta-analysis).

There was substantial heterogeneity for most of the alleles with the exception of HLA-DRB1*14, which showed a low level of heterogeneity only in the pemphigus allele frequency, but was highly heterogenic otherwise. HLA-DRB1*13 showed low heterogeneity in TTP and IgG4-AID excluding pemphigus, but moderate heterogeneity in all IgG4 AID collectively.

Due to the high level of heterogeneity between the studies, the publication bias was assessed only by funnel plots. We found a low to moderate and mostly symmetrical publication bias in HLA-DQB1*05 and HLA-DRB1*14, with very few outliers in both directions, while 1–2 outliers towards lower ORs were found for HLA-DRB1*13.

## Discussion

We conducted a systematic review and meta-analysis on the genotype, haplotype and allele frequency of reported HLA class II alleles across IgG4-AID and found that HLA- DQB1*05, an allele that is not typically associated with autoimmunity, is significantly more frequent in patients with IgG4-AID. This suggests it may be a genetic susceptibility factor for the production of IgG4 subclass antibodies. In addition, HLA-DRB1*14, a known genetic susceptibility factor for autoimmunity, is also associated with IgG4 autoimmunity, as is the HLA- DQB1*05-DRB1*14 haplotype. HLA-DRB1*13, which is considered as protective for autoimmunity in general, is also negatively associated with IgG4-AID. HLA-DRB1*03 and *04, which are often associated with autoimmunity, did not correlate with IgG4-AID, with the notable exception of pemphigus, which showed a strong association with HLA-DRB1*04.

Therefore, HLA-DRB1*14 and HLA-DQB1*05 may be genetic risk factors for IgG4 AID, and HLA-DRB1*13 may have a protective effect.

### Genetic associations with individual IgG4 autoimmune diseases

This is to the best of our knowledge the first systematic review and meta-analysis investigating a potential association of HLA class II alleles with IgG4-AID. Systematic reviews on individual IgG4-AID (Pemphigus, MuSK MG) agree with our findings^39,40^. A significant positive association of MuSK MG with HLA-DRB1*14, HLA-DRB1*16 and HLA-DQB1*05 could be confirmed in our study^40^. In contrast, a significant negative association for HLA-DQB1*03 reported in the MuSK MG study could not be reproduced in our analysis, and the reported negative association with HLA-DQB1*06 did not reach significance in our study. Possible reasons for this might be 1) the exclusion of one Italian study^48^ from our analysis that was included in the Hong study^40^ as it did not match our inclusion/exclusion criteria and 2) the use of different statistical methodology (random- vs fixed- effects model).

Our analysis of pemphigus data is in line with previous meta-analyses. Increased frequencies of HLA-DRB1*04 and HLA-DRB1*14 and decreased frequencies of HLA-DRB1*03, HLA-DRB1*07 and HLA-DRB1*15 were observed in the pemphigus patients^39^. In contrast to the latter study, we found HLA-DRB1*09, HLA-DRB1*11 and HLA-DRB1*13 also to be significantly decreased in pemphigus patients, but with a very broad 95% CI. In contrast to the Yan study^39^, there was no positive association with HLA-DRB1*08 and pemphigus, but analysis of pemphigus vulgaris studies only (data not shown) could reproduce the positive association for the genotype frequency. In a different study^49^ HLA-DQB1*05 and HLA-DQB1*03 were positively associated with pemphigus vulgaris, which is in line with our findings.

There were only few studies with haplotype data in IgG4-AID available, but the increased frequency of the HLA-DQB1*05-DRB1*14 haplotype may be due to linkage disequilibrium between the two genes.

Interestingly, while MuSK MG and pemphigus seem to have very similar genetic associations, TTP showed opposite effects for several alleles, and in HLA-DRB1*04 and HLA-DRB1*11 these were significant. Perhaps the different type and location of the antigen play a role: MuSK MG and pemphigus antibodies target antigens of the cell surface/extracellular matrix (type II hypersensitivity reactions, Gell and Coombs classification^50^), while ADAMTS13 is a soluble antigen (type III hypersensitivity reactions). Another explanation could be that there are shared sequence motifs between e.g. MuSK and desmoglein 1/3 that facilitate binding to the peptide binding groove that are not present in ADAMTS13, causing a decreased affinity of ADAMTS13 derived peptides to specific HLA alleles.

Systematic reviews on genetic associations of the HLA with TTP or CIDP were not available. Although antibodies against CNTN1 and NF155 are known since the early 2000s^51,52^, possible associations with HLA polymorphisms have only recently been determined and investigated. A (non-systematic) review^53^ also reports a handful of individual papers with genetic associations of neurological IgG4-AID with HLA-DQB1*05, namely MuSK MG and anti-IgLON5 disease, but different alleles for IgG4-AID with antibodies against LGI1 (HLA-DRB1*07:01), Caspr2 (HLA-DRB1*11:01) or neurofascin (HLA-DRB1*15). Whether these diseases are not associated with HLA-DRB1*14 and HLA-DQB1*05 cannot be concluded without further studies, as these were few studies with a low number of participants. HLA-DRB1*11 and *15* were also positively associated with IgG4-AID after exclusion of pemphigus (in addition to HLA-DRB1*12 and *16*), these could play a role in a different subset of patients, perhaps in neurological IgG4-AID.

Furthermore, the *DQB1* locus was not investigated in all studies. Nevertheless, it is very likely that several different genetic associations may exist that may predispose for the production of IgG4 autoantibodies in different forms of IgG4-AID, also depending on the structure of the autoantigens.

### Comparison of HLA associations between classical and IgG4 autoimmune diseases

We wanted to compare genetic HLA associations with classical autoimmune diseases (i.e. autoimmune diseases that are not caused by IgG4 autoantibodies) with the associations observed in IgG4-AID. In our study, HLA-DQB1*05 was associated strongly with IgG4-AID, and where higher resolution data was available, it was the HLA-DQB1*05:03 allele that was associated with IgG4-AID. Only few autoimmune diseases were reported to be associated with HLA-DQB1*05, and these are mostly IgG4-AID, including MuSK MG, pemphigus and anti-IgLON5 disease. In other autoimmune diseases, negative associations were found with the HLA-DQB1*05:02 in T1D^54,55^ and Sjögren’s syndrome^56^. One single study reported HLA-DQB1*05:02 to be positively associated with myelin oligodendrocyte glycoprotein-associated disorders (MOGAD), a rare neurological autoimmune disease^57^. Overall this suggests that HLA-DQB1*05 may be specifically associated with IgG4-AID. 

HLA-DRB1*14 also is strongly associated with IgG4-AID in our study, and was also reported as increased in patients with rheumatoid arthritis, Guillain-Barré syndrome and MuSK MG^22^, suggesting it may be a genetic risk factor to develop autoimmune diseases. HLA-DRB1*13 was found to be less frequent in IgG4-AID in our study, and this was also observed in classical AID, including T1D and autoimmune hepatitis^58–60^.

The HLA-DRB1*03 allele frequency is increased in classical AID, including diabetes mellitus type 1^54,55^, multiple sclerosis^61^, neuromyelitis optica^62^, systemic lupus erythematosus^63^, Graves’ disease^64^ and Sjögren’s syndrome^56^, but we observed no association across IgG4-AID, only a decrease in studies on pemphigus. A similar difference could be found for HLA-DRB1*04, which is increased in classical AID diabetes mellitus type 1, rheumatoid arthritis and autoimmune hepatitis patients^58,65^, but decreased in IgG4-AID (MuSK, TTP and CIDP)—with the exception of pemphigus, where a strong association was observed.

### HLA polymorphisms and the induction of IgG4 autoantibodies

Autoimmune diseases are thought to have a multifactorial etiology with a cumulative effect of genetic predispositions and environmental triggers. The shared pathophysiology indicates a common origin, leading to the investigation of common genetic factors in AIDs^66^. One genetic compound suggested for this susceptibility are the HLA class II genes, which encode proteins required for antigen presentation to CD4 + T-cells in the thymus and the periphery, thereby affecting central tolerance development and T-cell activation in the periphery. *HLA-DRB1*, the most polymorphic gene with over 1800 alleles, is frequently associated with autoimmune diseases^22^. Different HLA alleles present distinct peptide repertoires, and may directly affect T-cell fate by inducing Tconv or Tregs and a proinflammatory or tolerogenic cytokine environment^24,25^. The latter also includes IL-10, which is an important regulator for IgG4 production^26–31^. MuSK MG patients with the HLA-DRB1*14 allele were found to have higher autoantibody titers and higher levels of the cytokine IL-10 than patients with other HLA alleles^33^, and elevated IL-10 levels were found in several IgG4-AID, including pemphigus^34,35^, MuSK-MG^36^ and thrombotic thrombocytopenic purpura^37^. We hypothesize that HLA-DRB1*14, HLA-DQB1*05 and/or other HLA alleles may have therefore a direct effect on T-cell fate, favoring IL-10 producing Tregs and the production of tolerogenic cytokines which then induce class switch of B-cells to IgG4 and the production of IgG4 autoantibodies. How may HLA variants affect T-cell fate? The mechanism could depend for example on characteristics of the peptide repertoires presented in the different MHC II variants^25,67–69^, by differential interactions between the TCR and the HLA that may affect peptide recognition^70^ or by different cell type specific expression levels of the MHC II depending on the HLA variant^71^.

### IgG4-AID, IgG4-related diseases and IgG4 subclass

IgG4-related disease (IgG4-RLD) is the umbrella term for a distinct group of diseases associated with the IgG4 subclass, that is unrelated to IgG4-AID^14^. IgG4-RLD are clinically distinct from IgG4-AID, their pathogenic mechanism is unknown, the role of IgG4 in these diseases is unclear, and clinical characteristics of IgG4-RLD include fibrosis, IgG4 + plasma cell infiltrates in the tissue, organ swelling and increased serum IgG4 concentrations, which are not characteristic for IgG4-AID^14^. In line with these findings, HLA associations also differ for IgG4-RLD, which was found to be associated with HLA-DRB1*04 allele^72^.The pathogenic mechanisms of IgG4 and the regulatory mechanisms that lead to the production of pathogenic IgG4 in IgG4-AID are not well understood, and are subject of an ongoing review series^13,15,17^.

### Study limitations

The main limitation of the study was owed to the low prevalence of IgG4-AID, including (1) small numbers of patient per individual study (mostly between 30 and 100 patients), and (2) a low number of available studies, leading to (3) substantial heterogeneity, which was especially pronounced in studies on TTP. Pooling of data was not always possible due to different types of analysis and the differential use of nomenclature (e.g. genotype, haplotype, allele and phenotype frequency). Lack of information on homozygosity or heterozygosity in studies with genotype frequencies prevented a combined analysis for allele and genotype frequency, and since the HLA genes are in linkage disequilibrium^73^, homo- and heterozygosity cannot be “re-calculated” by using the Hardy–Weinberg equilibrium. Therefore, we only included studies where the frequency was given in absolute and relative numbers and data for allele and genotype frequency was analyzed individually. Several studies used a single control group for two different datasets, and to avoid overestimating the number of controls, data of these studies was pooled where possible^74–77^ (exception: two studies from Serbia^78,79^). All studies included in the meta-analysis reported that the controls and patients derived from the same geographic location or that the controls were ethnically matched to the patients, but most studies did not provide further details on the ethnical matching.

Furthermore, high-resolution data was only available from a subset of studies, mostly on pemphigus, therefore the observed associations with the specific HLA-DQB1*05:03, HLA-DRB1*14:01 and DRB1*14:04 alleles need to be validated in further studies. Heterogeneity in ancestries across countries was addressed by only including studies with patients and controls that were ethnically matched and/or derived from the same population and use of the random-effects model for the meta-analysis.

Our understanding of the proposed kinship between individual IgG4-AID is very limited^1,12,16^, and it is likely that there are different true effects of the HLA alleles in the distinct diseases. To account for this possibility, we used a random-effects model and also analyzed the diseases individually. Since there was a predominance of pemphigus studies (37/52 studies), we re-analyzed the data after exclusion of the pemphigus studies and could reproduce the associations with the HLA-DRB1*13, HLA-DRB1*14 and HLA-DQB1*05 alleles and the HLA-DRB1*14-DQB1*05 haplotype. In contrast, the HLA-DRB1*04 allele, which was more frequent in pemphigus patients, was not associated with the other diseases.

Antibody tests were not described in a substantial number of studies on pemphigus, but histopathologic diagnosis implicates the presence of the relevant IgG4 autoantibodies (mostly desmoglein 1 and desmoglein 3, < 0.5% of patients desmocollin), the inclusion criteria were changed during the second round of screening to include the pemphigus studies in the quantitative analysis. The PRISMA statement acknowledges this iterative process and accepts that modifications in the review protocol during the synthesis may sometimes be inevitable^45^.

## Conclusions

With the limitations of this study in mind, we observed an increased frequency of HLA-DRB1*14 and HLA-DQB1*05 alleles as well as the HLA -DQB1*05 -DRB1*14 haplotype in patients with IgG4 AID. These findings agree with the literature, where these alleles are also associated with individual IgG4-AIDs. Thus HLA-DRB1*14 and HLA-DQB1*05 individually—or in combination as haplotype—might pose a genetic risk factor for the susceptibility to develop IgG4 AID. HLA-DRB1*13 seems to be consistently less frequent in patients, indicating a possible protective effect. Nevertheless, the low number of individual studies and the relatively small patient cohorts contributed to the substantial heterogeneity, therefore further HLA association studies are needed to validate the findings.

## Supplementary Information

Supplementary Information.

## Data Availability

To foster transparency, we provide all data generated in this study in the supplementary materials.

## Abbreviations

CIDP: Chronic inflammatory demyelinating polyneuropathy
HLA: Human leukocyte antigen
IgG4-AID: IgG4 autoimmune diseases
FS: Fogo selvagem
MuSK: Muscle-specific kinase
MuSK MG: MuSK myasthenia gravis
MHC: Major histocompatibility complex
PF: Pemphigus foliaceus
PV: Pemphigus vulgaris
TTP: Thrombotic thrombocytopenic purpura

## References

[1] Huijbers MG (2015). The expanding field of IgG4-mediated neurological autoimmune disorders. Eur. J. Neurol..

[2] Koneczny I (2018). A new classification system for IgG4 autoantibodies. Front. Immunol..

[3] van der Neut Kolfschoten M (2007). Anti-inflammatory activity of human IgG4 antibodies by dynamic Fab arm exchange. Science.

[4] Davies AM (2014). Structural determinants of unique properties of human IgG4-Fc. J. Mol. Biol..

[5] Aalberse RC, Stapel SO, Schuurman J, Rispens T (2009). Immunoglobulin G4: an odd antibody. Clin. Exp. Allergy.

[6] Lighaam LC, Rispens T (2016). The immunobiology of immunoglobulin G4. Semin. Liver Dis..

[7] Kemeny DM, MacKenzie-Mills M, Harries MG, Youlten LJ, Lessof MH (1983). Antibodies to purified bee venom proteins and peptides II A detailed study of changes in IgE and IgG antibodies to individual bee venom antigens. J. Allergy Clin. Immunol..

[8] Kemeny DM, McKenzie-Mills M, Harries MG, Youlten LJ, Lessof MH (1983). Changes in the levels of anti-phospholipase A2 and hyaluronidase antibodies during bee venom immunotherapy. Monogr. Allergy.

[9] Subbarayal B (2013). Kinetics, cross-reactivity, and specificity of Bet v 1-specific IgG4 antibodies induced by immunotherapy with birch pollen. Allergy.

[10] van Neerven RJ (1999). Blocking antibodies induced by specific allergy vaccination prevent the activation of CD4+ T cells by inhibiting serum-IgE-facilitated allergen presentation. J. Immunol..

[11] Bodtger U (2005). Is immunotherapy-induced birch-pollen-specific IgG4 a marker for decreased allergen-specific sensitivity?. Int. Arch. Allergy Immunol..

[12] Koneczny I (2018). A New Classification System for IgG4 Autoantibodies. Front. Immunol..

[13] Koneczny I (2020). Update on IgG4-mediated autoimmune diseases: New insights and new family members. Autoimmun. Rev..

[14] Endmayr V (2021). Anti-neuronal IgG4 autoimmune diseases and IgG4-related diseases may not be part of the same spectrum: a comparative study. Front. Immunol..

[15] Koneczny I (2021). Common denominators in the immunobiology of IgG4 autoimmune diseases: what do glomerulonephritis, pemphigus vulgaris, myasthenia gravis, thrombotic thrombocytopenic purpura and autoimmune encephalitis have in common?. Front. Immunol..

[16] Huijbers MG, Plomp JJ, van der Maarel SM, Verschuuren JJ (2018). IgG4-mediated autoimmune diseases: a niche of antibody-mediated disorders. Ann. N. Y. Acad. Sci..

[17] Koneczny I (2022). IgG4 autoantibodies in organ-specific autoimmunopathies: reviewing class switching, antibody-producing cells, and specific immunotherapies. Front. Immunol..

[18] Matzaraki V, Kumar V, Wijmenga C, Zhernakova A (2017). The MHC locus and genetic susceptibility to autoimmune and infectious diseases. Genome Biol..

[19] Lokki ML, Paakkanen R (2019). The complexity and diversity of major histocompatibility complex challenge disease association studies. HLA.

[20] Unanue ER, Turk V, Neefjes J (2016). Variations in MHC class II antigen processing and presentation in health and disease. Annu. Rev. Immunol..

[21] Tsai S, Santamaria P (2013). MHC class II polymorphisms, autoreactive T-cells, and autoimmunity. Front. Immunol..

[22] Arango MT (2017). HLA-DRB1 the notorious gene in the mosaic of autoimmunity. Immunol. Res..

[23] Alvarez I (2015). Central T cell tolerance: Identification of tissue-restricted autoantigens in the thymus HLA-DR peptidome. J. Autoimmun..

[24] Logunova NN (2020). MHC-II alleles shape the CDR3 repertoires of conventional and regulatory naive CD4(+) T cells. Proc. Natl. Acad. Sci. U. S. A..

[25] Ooi JD (2017). Dominant protection from HLA-linked autoimmunity by antigen-specific regulatory T cells. Nature.

[26] Satoguina JS, Weyand E, Larbi J, Hoerauf A (2005). T regulatory-1 cells induce IgG4 production by B cells: role of IL-10. J. Immunol..

[27] Satoguina JS (2008). Tr1 and naturally occurring regulatory T cells induce IgG4 in B cells through GITR/GITR-L interaction, IL-10 and TGF-beta. Eur. J. Immunol..

[28] Punnonen J (1993). Interleukin 13 induces interleukin 4-independent IgG4 and IgE synthesis and CD23 expression by human B cells. Proc. Natl. Acad. Sci. U. S. A..

[29] Meiler F (2008). In vivo switch to IL-10-secreting T regulatory cells in high dose allergen exposure. J. Exp. Med..

[30] Meiler F, Klunker S, Zimmermann M, Akdis CA, Akdis M (2008). Distinct regulation of IgE, IgG4 and IgA by T regulatory cells and toll-like receptors. Allergy.

[31] Jeannin P, Lecoanet S, Delneste Y, Gauchat JF, Bonnefoy JY (1998). IgE versus IgG4 production can be differentially regulated by IL-10. J. Immunol..

[32] Berntsen NL (2015). Association between HLA haplotypes and increased serum levels of IgG4 in patients with primary sclerosing cholangitis. Gastroenterology.

[33] Cebi M (2019). Relation of HLA-DRB1 to IgG4 autoantibody and cytokine production in muscle-specific tyrosine kinase myasthenia gravis (MuSK-MG). Clin. Exp. Immunol..

[34] Bhol KC, Rojas AI, Khan IU, Ahmed AR (2000). Presence of interleukin 10 in the serum and blister fluid of patients with pemphigus vulgaris and pemphigoid. Cytokine.

[35] Satyam A, Khandpur S, Sharma VK, Sharma A (2009). Involvement of T(H)1/T(H)2 cytokines in the pathogenesis of autoimmune skin disease-Pemphigus vulgaris. Immunol. Invest..

[36] Yilmaz V (2015). Differential cytokine changes in patients with myasthenia gravis with antibodies against AChR and MuSK. PLoS ONE.

[37] Westwood JP, Langley K, Heelas E, Machin SJ, Scully M (2014). Complement and cytokine response in acute Thrombotic Thrombocytopenic Purpura. Br. J. Haematol..

[38] Aversa G (1993). An interleukin 4 (IL-4) mutant protein inhibits both IL-4 or IL-13-induced human immunoglobulin G4 (IgG4) and IgE synthesis and B cell proliferation: support for a common component shared by IL-4 and IL-13 receptors. J. Exp. Med..

[39] Yan L, Wang JM, Zeng K (2012). Association between HLA-DRB1 polymorphisms and pemphigus vulgaris: a meta-analysis. Br. J. Dermatol..

[40] Hong Y, Li HF, Romi F, Skeie GO, Gilhus NE (2018). HLA and MuSK-positive myasthenia gravis: a systemic review and meta-analysis. Acta Neurol. Scand..

[41] Little J, H. J. e. (Centers for Disease Control and Prevention, Equator network, 2006).

[42] Stroup DF (2000). Meta-analysis of observational studies in epidemiology: a proposal for reporting. Meta-analysis Of Observational Studies in Epidemiology (MOOSE) group. JAMA.

[43] Methley AM, Campbell S, Chew-Graham C, McNally R, Cheraghi-Sohi S (2014). PICO, PICOS and SPIDER: a comparison study of specificity and sensitivity in three search tools for qualitative systematic reviews. BMC Health Serv. Res..

[44] Ouzzani M, Hammady H, Fedorowicz Z, Elmagarmid A (2016). Rayyan-a web and mobile app for systematic reviews. Syst. Rev..

[45] Moher, D., Liberati, A., Tetzlaff, J., Altman, D. G. & Group, P. Preferred reporting items for systematic reviews and meta-analyses: the PRISMA statement. *J. Clin. Epidemiol.* **62**, 1006-1012, 10.1016/j.jclinepi.2009.06.005 (2009). 10.1016/j.jclinepi.2009.06.005 19631508

[46] Clarke GM (2011). Basic statistical analysis in genetic case-control studies. Nat. Protoc..

[47] Moher, D., Liberati, A., Tetzlaff, J., Altman, D. G. & Group, P. Preferred reporting items for systematic reviews and meta-analyses: the PRISMA statement. *PLoS Med.* **6**, e1000097, 10.1371/journal.pmed.1000097 (2009). 10.1371/journal.pmed.1000097 PMC2707599 19621072

[48] Bartoccioni E (2009). HLA class II allele analysis in MuSK-positive myasthenia gravis suggests a role for DQ5. Neurology.

[49] Li S (2018). Association between HLA-DQB1 polymorphisms and pemphigus vulgaris: a meta-analysis. Immunol. Invest..

[50] Gell PGH, Coombs RRA (1963). Clinical Aspects of Immunology.

[51] Boyle MET (2001). Contactin orchestrates assembly of the septate-like junctions at the paranode in myelinated peripheral nerve. Neuron.

[52] Tait S (2000). An oligodendrocyte cell adhesion molecule at the site of assembly of the paranodal axo-glial junction. J. Cell Biol..

[53] Muniz-Castrillo S, Vogrig A, Honnorat J (2020). Associations between HLA and autoimmune neurological diseases with autoantibodies. Auto-immun. Highlights.

[54] Rojas-Villarraga A, Botello-Corzo D, Anaya JM (2010). HLA-Class II in Latin American patients with type 1 diabetes. Autoimmun. Rev..

[55] Hajjej A (2019). Association of HLA-DRB1 and -DQB1 alleles with type 1 (autoimmune) diabetes in African Arabs: systematic review and meta-analysis. Immunol. Invest..

[56] Cruz-Tapias P, Rojas-Villarraga A, Maier-Moore S, Anaya JM (2012). HLA and Sjogren's syndrome susceptibility. a meta-analysis of worldwide studies. Autoimmun. Rev..

[57] Sun X (2020). Myelin oligodendrocyte glycoprotein-associated disorders are associated with HLA subtypes in a Chinese paediatric-onset cohort. J. Neurol. Neurosurg. Psychiatry.

[58] Cruz-Tapias P (2012). Shared HLA class II in Six autoimmune diseases in Latin America: a meta-analysis. Autoimmun. Dis..

[59] Bettencourt A (2015). The protective role of HLA-DRB1(∗)13 in autoimmune diseases. J. Immunol. Res..

[60] Gough SCL, Simmonds MJ (2007). The HLA region and autoimmune disease: associations and mechanisms of action. Curr. Genom..

[61] Zhang Q, Lin CY, Dong Q, Wang J, Wang W (2011). Relationship between HLA-DRB1 polymorphism and susceptibility or resistance to multiple sclerosis in Caucasians: a meta-analysis of non-family-based studies. Autoimmun. Rev..

[62] Alvarenga MP (2021). Neuromyelitis optica is an HLA associated disease different from multiple sclerosis: a systematic review with meta-analysis. Sci. Rep..

[63] Hachicha, H. *et al.* HLA-DRB1*03 is associated with systemic lupus erythematosus and anti -SSB production in South Tunisia. *Int. J. Health Sci.* **12** (2018). PMC5870315 29623013

[64] Heward JM (1998). Linkage disequilibrium between the human leukocyte antigen class II region of the major histocompatibility complex and graves’ disease: replication using a population case control and family-based study1. J. Clin. Endocrinol. Metab..

[65] Yang M (2013). Meta-analysis of the association of HLA-DRB1 with rheumatoid arthritis in Chinese populations. BMC Musculoskelet. Disord..

[66] Anaya J-M (2012). Common mechanisms of autoimmune diseases (the autoimmune tautology). Autoimmun. Rev..

[67] Scholz EM (2017). Human Leukocyte Antigen (HLA)-DRB1*15:01 and HLA-DRB5*01:01 Present Complementary Peptide Repertoires. Front. Immunol..

[68] Logunova NN (2020). MHC-II alleles shape the CDR3 repertoires of conventional and regulatory naive CD4(+) T cells. Proc. Natl. Acad. Sci. U. S. A..

[69] Hrdinova J (2018). Mass spectrometry-assisted identification of ADAMTS13-derived peptides presented on HLA-DR and HLA-DQ. Haematologica.

[70] Coles CH (2020). T cell receptor interactions with human leukocyte antigen govern indirect peptide selectivity for the cancer testis antigen MAGE-A4. J. Biol. Chem..

[71] Houtman M (2021). Haplotype-specific expression analysis of MHC class II genes in healthy individuals and rheumatoid arthritis patients. Front. Immunol..

[72] Terao C (2019). IgG4-related disease in the Japanese population: a genome-wide association study. Lancet Rheumatol..

[73] Vandiedonck C, Knight JC (2009). The human Major Histocompatibility Complex as a paradigm in genomics research. Brief Funct. Genomic Proteomic.

[74] Torzecka JD (2003). Tumour necrosis factor-alpha polymorphism as one of the complex inherited factors in pemphigus. Mediators Inflamm..

[75] Zhang SY (2019). Subtype-specific inherited predisposition to pemphigus in the Chinese population. Br. J. Dermatol..

[76] Lee CW, Yang HY, Kim SC, Jung JH, Hwang JJ (1998). HLA class II allele associations in korean patients with pemphigus. Dermatology.

[77] Miyagawa S (1997). HLA-DRB1*04 and DRB1*14 alleles are associated with susceptibility to pemphigus among Japanese. J. Invest. Dermatol..

[78] Nikolic AV (2015). High frequency of DQB1*05 and absolute absence of DRB1*13 in muscle-specific tyrosine kinase positive myasthenia gravis. Eur. J. Neurol..

[79] Zivanovic D, Bojic S, Medenica L, Andric Z, Popadic D (2016). Human leukocyte antigen class II (DRB1 and DQB1) alleles and haplotypes frequencies in patients with pemphigus vulgaris among the Serbian population. HLA.

[80] Dere G (2020). Assessment of HLA-A, HLA-DR, and HLA-DQ alleles in patients with pemphigus vulgaris from eastern of Turkey. J. Cosmet. Dermatol..

[81] Ehsan S (2015). Association of HLA class II (DRB1, DQA1, DQB1) alleles and haplotypes with myasthenia gravis and its subgroups in the Iranian population. J. Neurol. Sci..

[82] Alahgholi-Hajibehzad M (2013). Association of HLA-DRB1 *14, -DRB1 *16 and -DQB1 *05 with MuSK-myasthenia gravis in patients from Turkey. Hum. Immunol..

[83] Harfouch E, Daoud S (2014). Allelic variation in HLA-DRB1* loci in Syrian pemphigus vulgaris patients. Int. J. Dermatol..

[84] González-Escribano MF (1998). Distribution of HLA class II alleles among Spanish patients with pemphigus vulgaris. Tissue Antigens.

[85] Brochado MJ (2016). Differential HLA class I and class II associations in pemphigus foliaceus and pemphigus vulgaris patients from a prevalent Southeastern Brazilian region. J. Autoimmun..

[86] Martel P (2002). Epistasis between DSG1 and HLA class II genes in pemphigus foliaceus. Genes Immun..

[87] Párnická Z, Švecová D, Javor J, Shawkatová I, Buc M (2013). High susceptibility to pemphigus vulgaris due to HLA-DRB1*14:54 in the Slovak population. Int. J. Immunogenet..

[88] de Sena Nogueira Maehara L (2018). HLA class II alleles of susceptibility and protection in Brazilian and Dutch pemphigus foliaceus. Br. J. Dermatol..

[89] Coppo P (2010). HLA-DRB1*11: a strong risk factor for acquired severe ADAMTS13 deficiency-related idiopathic thrombotic thrombocytopenic purpura in Caucasians. J. Thromb. Haemost..

[90] Kanai T (2016). HLA-DRB1*14 and DQB1*05 are associated with Japanese anti-MuSK antibody-positive myasthenia gravis patients. J. Neurol. Sci..

[91] Cotti Piccinelli S (2019). Human leukocyte antigens class II in CIDP spectrum neuropathies. J. Neurol. Sci..

[92] Saha M (2019). Sporadic pemphigus foliaceus and class II human leucocyte antigen allele associations in the white British and Indo-Asian populations in the UK. Clin. Exp. Dermatol..

[93] Gil JM (2017). Study of the association between human leukocyte antigens (HLA) and pemphigus vulgaris in Brazilian patients. Int. J. Dermatol..

[94] Abida O (2009). Tunisian endemic pemphigus foliaceus is associated with the HLA-DR3 gene: anti-desmoglein 1 antibody-positive healthy subjects bear protective alleles. Br. J. Dermatol..

[95] Ogata H (2020). Unique HLA haplotype associations in IgG4 anti-neurofascin 155 antibody-positive chronic inflammatory demyelinating polyneuropathy. J. Neuroimmunol..

[96] Priyadarshini A, George R, Daniel D, Varughese S, Jayaseelan V (2018). Association between human leukocyte antigen-DRB1 and human leukocyte antigen-DQB1 alleles and pemphigus vulgaris in Indian patients: a case-control study. Indian J. Dermatol. Venereol. Leprol..

[97] Tunca M, Musabak U, Sagkan RI, Koc E, Akar A (2010). Association of human leukocyte antigen class II alleles with pemphigus vulgaris in a Turkish population. J. Dermatol..

[98] Haase O (2015). Association with HLA-DRB1 in Egyptian and German pemphigus vulgaris patients. Tissue Antigens.

[99] Martinez-Martinez L (2017). Anti-NF155 chronic inflammatory demyelinating polyradiculoneuropathy strongly associates to HLA-DRB15. J. Neuroinflammation.

[100] Glorio RR (1999). Determinacion por PCR de la asociacion entre antigenos HLA clase II y penfigo vulgar. Med. (Buenos Aires).

[101] Pavoni DP, Roxo VMMS, Marquart Filho A, Petzl-Erler ML (2003). Dissecting the associations of endemic Pemphigus Foliaceus (Fogo Selvagem) with HLA-DRB1 alleles and genotypes. Genes Immun..

[102] Thomas GJJ, Conejo-Mir JS, Escribano FG, Bernal AMP, Roldán AN (1998). Estudio de los alelos de HLA de clase II que confieren susceptibilidad al pénfigo vulgar en una población andaluza. ACTAS Dermo-Sifiliograficas.

[103] Moraes JR (1991). HLA antigens and risk for development of pemphigus foliaceus (fogo selvagem) in endemic areas of Brazil. Immunogenetics.

[104] Shams S (2009). HLA Class II (DRB, DQA1 and DQB1) allele and haplotype frequencies in the patients with pemphigus vulgaris. J. Clin. Immunol..

[105] Orouji E, Tavakkol Afshari J, Schmieder A, Layegh P (2014). HLA-DQB1 gene and pemphigus vulgaris in patients with Mid-East origin. J. Dermatol. Sci..

[106] Joly BS (2020). HLA-DRB1*11 is a strong risk factor for acquired thrombotic thrombocytopenic purpura in children. Haematologica.

[107] Glorio R (2002). HLA haplotypes and class II molecular alleles in argentinian patients with pemphigus vulgaris. JCMS.

[108] Sakai K (2020). HLA loci predisposing to immune TTP in Japanese: potential role of the shared ADAMTS13 peptide bound to different HLA-DR. Blood.

[109] Koc CK, Sallakci N, Akman-Karakaş A, Alpsoy E, Yegin O (2013). Human leukocyte antigens class I and class II in patients with pemphigus in southern Turkey. Int. J. Dermatol..

[110] Scully M (2010). Human leukocyte antigen association in idiopathic thrombotic thrombocytopenic purpura: evidence for an immunogenetic link. J. Thromb. Haemost..

[111] Mobini N (1997). Identical MHC markers in non-jewish iranian and ashkenazi jewish patients with pemphigus vulgaris: possible common central asian ancestral origin. Hum. Immunol..

[112] Delgado JC (1997). Pemphigus vulgaris autoantibody response is linked to HLA-DQB10503 in Pakistani Patients. Hum. Immunol..

[113] Yamashina Y (1998). Polymorphisms of HLA class II genes in Japanese patients with pemphigus vulgaris. Tissue Antigens.

[114] Al Haddad C, Finianos P, Zgheib E, Germanos M, Coppo P (2019). Risk factors associated with the human leucocyte antigen system in Lebanese patients with immune-mediated thrombotic thrombocytopenic purpura. La Presse Médicale.

[115] John M-L, Hitzler W, Scharrer I (2012). The role of human leukocyte antigens as predisposing and/or protective factors in patients with idiopathic thrombotic thrombocytopenic purpura. Ann. Hematol..

[116] Martino S (2016). Thrombotic thrombocytopenic purpura in black people: impact of ethnicity on survival and genetic risk factors. PLoS ONE.

[117] Cerna M (1993). Genetic markers for susceptibility to endemic Brazilian pemphigus foliaceus (Fogo Selvagem) in Xavante Indians. Tissue Antigens.

[118] Birol A, Anadolu RY, Tutkak H, Gürgey E (2002). HLA-class 1 and class 2 antigens in Turkish patients with pemphigus. Int. J. Dermatol..

[119] Rangel-Gamboa L, Vega-Memije ME, Acuña-Alonzo V, Granados-Arriola J (2016). HLA class II in Mexican patients with pemphigus vulgaris: shared epitope for autoimmunity. Gac. Med. Mex..

[120] Khan SW, Iftikhar N, Ahmed TA, Bashir M (2015). HLA- DR alleles in pakistani patients of pemphigus vulgaris. J. Coll. Phys. Surg. Pak..

[121] Carcassi C (1996). HLA haplotypes and class II molecular alleles in Sardinian and Italian patients with pemphigus vulgaris. Tissue Antigens.

[122] Lombardi ML (1996). Molecular analysis of HLA DRB1 and DQB1 in Italian patients with pemphigus vulgaris. Tissue Antigens.

[123] Saha M (2010). Pemphigus Vulgaris in White Europeans Is Linked with HLA Class II Allele HLA DRB1*1454 but Not DRB1*1401. J. Investig. Dermatol..

[124] Niks EH (2006). Strong association of MuSK antibody-positive myasthenia gravis and HLA-DR14-DQ5. Neurology.

[125] Sinkovits G (2017). The role of human leukocyte antigen DRB1-DQB1 haplotypes in the susceptibility to acquired idiopathic thrombotic thrombocytopenic purpura. Hum. Immunol..

