# Supplementary material for: A systematic review and meta-analysis of HLA class II associations in patients with IgG4 autoimmunity
Source: Sci Rep. 2022 Jun 2;12:9229. doi: 10.1038/s41598-022-13042-2 (PMC9163138; doi:10.1038/s41598-022-13042-2)
Supplement: Supplementary file 1 — Supplementary Information. [file 41598_2022_13042_MOESM1_ESM.pdf]

Supplementary Material

Supplementary results

Supplementary Table S1: List of all 34 bibliographic databases for electronic database search

Databases	URL
Analysis & policy observatory	https://apo.org.au/
ArXiv	https://arxiv.org
BioOne	https://bioone.org
BIOSIS	https://www.ebsco.com/products/research-databases/biosis-previews
BioRxiv	https://www.biorxiv.org/
MedRxiv	https://www.medrxiv.org/
Centre for Reviews and Dissemination	https://www.york.ac.uk/crd/
CINAHL	https://www.ebscohost.com/nursing/products/cinahl-databases/cinahl-complete
ClinicalTrials.gov	https://clinicaltrials.gov
Cochrane Central Register of Controlled Trials (CENTRAL)	https://www.cochranelibrary.com/central
Cochrane Library	https://www.cochranelibrary.com/
DOAJ	https://doaj.org/
EMBASE	https://www.embase.com/welcome.jsp#search
Epistemonikos	https://www.epistemonikos.org/
EU Clinical Trials Register	https://www.clinicaltrialsregister.eu/ctr-search/search
GlaxoSmithKline's Clinical Study Register	https://www.gsk-studyregister.com/en/?legacy=true
Godort	http://www.ala.org/rt/godort
HSRProj	https://hsrproject.nlm.nih.gov
JSTOR	https://www.jstor.org/
mendeley	https://www.mendeley.com/
metaRegister of Controlled Trials (Current controlled trials)	http://www.isrctn.com/mrct/
open grey	http://www.opengrey.eu
Ovid global health	https://www.ovid.com/product-details.901.html
Prospero	https://www.crd.york.ac.uk/prospero/
PubMed	www.ncbi.nlm.nih.gov/pubmed/
Research gate	https://www.researchgate.net
Science Citation Index (ISI)	https://isindexing.com/
Science direct	https://www.sciencedirect.com
Scopus	https://www.scopus.com/home.uri
TRIP Database	https://www.tripdatabase.com
U.S. Government Documents	https://guides.library.upenn.edu/usgovdocuments
Web of Science	www.webofknowledge.com
WHO ICTRP	https://apps.who.int/trialsearch/
Worldcat	https://www.worldcat.org/

Supplementary Table S2: List of 52 studies included in qualitative or quantitative synthesis

Reference	Study title	Quantitative/Qualitative
1	Assessment of HLA-A, HLA-DR, and HLA-DQ alleles in patients with pemphigus vulgaris from eastern of Turkey	Quantitative/Qualitative
2	Association of HLA class II (DRB1, DQA1, DQB1) alleles and haplotypes with myasthenia gravis and its subgroups in the Iranian population	Quantitative/Qualitative
3	Association of HLA-DRB1∗14, -DRB1∗16 and -DQB1∗05 with MuSK-myasthenia gravis in patients from Turkey	Quantitative/Qualitative
4	Allelic variation in HLA-DRB1* loci in Syrian pemphigus vulgaris patients	Quantitative/Qualitative
5	Distribution of HLA class II alleles among Spanish patients with pemphigus vulgaris.	Quantitative/Qualitative
6	Differential HLA class I and class II associations in pemphigus foliaceus and pemphigus vulgaris patients from a prevalent Southeastern Brazilian region	Quantitative/Qualitative
7	Epistasis between DSG1 and HLA class II genes in pemphigus foliaceus	Quantitative/Qualitative
8	High susceptibility to pemphigus vulgaris due to HLA-DRB1*14:54 in the Slovak population	Quantitative/Qualitative
9	High frequency of DQB1*05 and absolute absence of DRB1*13 in muscle-specific tyrosine kinase positive myasthenia gravis	Quantitative/Qualitative
10	HLA class II alleles of susceptibility and protection in Brazilian and Dutch pemphigus foliaceus	Quantitative/Qualitative
11	HLA-DRB1*11: a strong risk factor for acquired severe ADAMTS13 deficiency-related idiopathic thrombotic thrombocytopenic purpura in Caucasians.	Quantitative/Qualitative
12	HLA-DRB1*14 and DQB1*05 are associated with Japanese anti-MuSK antibody-positive myasthenia gravis patients	Quantitative/Qualitative
13	Human leukocyte antigens class II in CIDP spectrum neuropathies	Quantitative/Qualitative
14	Human leukocyte antigen class II (DRB1 and DQB1) alleles and haplotypes frequencies in patients with pemphigus vulgaris among the Serbian population	Quantitative/Qualitative
15	Sporadic pemphigus foliaceus and class II human leucocyte antigen allele associations in the white British and Indo-Asian populations in the UK	Quantitative/Qualitative
16	Study of the association between human leukocyte antigens (HLA) and pemphigus vulgaris in Brazilian patients	Quantitative/Qualitative
17	Tumour necrosis factor-α polymorphism as one of the complex inherited factors in pemphigus	Quantitative/Qualitative
18	Tunisian endemic pemphigus foliaceus is associated with the HLA-DR3 gene: Anti-desmoglein 1 antibody-positive healthy subjects bear protective alleles	Quantitative/Qualitative
19	Unique HLA haplotype associations in IgG4 anti-neurofascin 155 antibody-positive chronic inflammatory	Quantitative/Qualitative
20	Association between human leukocyte antigen-DRB1 and human leukocyte antigen-DQB1 alleles and pemphigus vulgaris in Indian patients: A case-control study	Quantitative/Qualitative
21	Association of human leukocyte antigen class II alleles with pemphigus vulgaris in a Turkish population	Quantitative/Qualitative
22	Association with HLA-DRB1 in Egyptian and German pemphigus vulgaris patients	Quantitative/Qualitative

23	Anti-NF155 chronic inflammatory demyelinating polyradiculoneuropathy strongly associates to HLA-DRB15	Quantitative/Qualitative
24	Determinacion por PCR de la asociacion entre antigenos HLA clase II y penfigo vulgar	Quantitative/Qualitative
25	Dissecting the associations of endemic pemphigus foliaceus (fogo selvagem) with HLA-DRB1 alleles and genotypes	Quantitative/Qualitative
26	Estudio de los alelos de HLA de clase II que confierent susceptibilidad al pénfigo vulgar en una población andaluza	Quantitative/Qualitative
27	(GWAS of Pemphigus) Subtype-specific inherited predisposition to pemphigus in the Chinese population	Quantitative/Qualitative
28	HLA antigens and risk for development of pemphigus foliaceus (fogo selvagem) in endemic areas of Brazil	Quantitative/Qualitative
29	HLA class II allele associations in Korean patients with pemphigus.	Quantitative/Qualitative
30	HLA class II (DRB, DQA1 and DQB1) allele and haplotype frequencies in the patients with pemphigus vulgaris	Quantitative/Qualitative
31	HLA-DQB1 gene and pemphigus vulgaris in patients with mid-east origin	Quantitative/Qualitative
32	HLA-DRB1*04 and DRB1*14 alleles are associated with susceptibility to pemphigus among Japanese	Quantitative/Qualitative
33	HLA-DRB1*11 is a strong risk factor for acquired thrombotic thrombocytopenic purpura in children	Qualitative
34	HLA haplotypes and class II molecular alleles in Argentinian patients with pemphigus vulgaris	Quantitative/Qualitative
35	HLA loci predisposing to immune TTP in Japanese: potential role of the shared ADAMTS13 peptide bound to different HLA-DR	Quantitative/Qualitative
36	Human leukocyte antigens class I and class II in patients with pemphigus in southern Turkey	Quantitative/Qualitative
37	Human leukocyte antigen association in idiopathic thrombotic thrombocytopenic purpura: Evidence for an immunogenetic link	Quantitative/Qualitative
38	Identical MHC markers in Non-Jewish Iranian and Ashkenazi Jewish patients with pemphigus vulgaris: Possible common Central Asian Ancestral Origin	Quantitative/Qualitative
39	Pemphigus vulgaris autoantibody response is linked to HLA-DQB1*0503 in Pakistani patients.	Quantitative/Qualitative
40	Polymorphisms of HLA Class II Genes in Japanese Patients with Pemphigus Vulgaris and Pemphigus Foliaceus	Quantitative/Qualitative
41	Risk factors associated with the human leucocyte antigen system in Lebanese patients with immune-mediated thrombotic thrombocytopenic purpura	Quantitative/Qualitative
42	The role of human leukocyte antigens as predisposing and/or protective factors in patients with idiopathic thrombotic thrombocytopenic purpura	Quantitative/Qualitative
43	Thrombotic Thrombocytopenic Purpura in Black People: Impact of Ethnicity on Survival and Genetic Risk Factors.	Quantitative/Qualitative
44	Genetic markers for susceptibility to endemic Brazilian pemphigus foliaceus (Fogo Selvagem) in Xavante Indians.	Quantitative/Qualitative
45	HLA-class 1 and class 2 antigens in Turkish patients with pemphigus	Quantitative/Qualitative
46	HLA class II in Mexican patients with pemphigus vulgaris: shared epitope for autoimmunity	Quantitative/Qualitative
47	HLA- DR Alleles in Pakistani patients of pemphigus vulgaris	Quantitative/Qualitative
48	HLA haplotypes and class II molecular alleles in Sardinian and Italian patients with pemphigus vulgaris	Quantitative/Qualitative
49	Molecular analysis of HLA DRB1 and DQB1 in Italian patients with pemphigus vulgaris	Quantitative/Qualitative
50	Pemphigus vulgaris in white Europeans is linked with HLA class II allele HLA DRB1 1454 but not DRB1 1401	Quantitative/Qualitative

51	Strong association of MuSK antibody–positive myasthenia gravis and HLA-DR14-DQ5	Quantitative/Qualitative
52	The role of human leukocyte antigen DRB1-DQB1 haplotypes in the susceptibility to acquired idiopathic thrombotic thrombocytopenic purpura.	Quantitative/Qualitative

Supplementary Table S3: Table of high-resolution alleles and number of studies (in allele and genotype frequency, respectively)

DRB1*04			DRB1*13		
	genotype	allele		genotype	allele
*04:02	7	7	*13:01	2	4
			*13:02	1	5

DRB1*14			DQB1*05		
	genotype	allele		genotype	allele
*14:01	8	8	*05:01	4	9
*14:04	2	6	*05:02	5	6
*1454	0	6	*05:03	7	9

Supplementary Table S4: Table of high-resolution alleles and number of studies (no separation in allele and genotype frequency)

DRB1*04		DRB1*13	
	overall		overall
*04:02	14	*13:01	6
		*13:02	6

DRB1*14		DQB1*05	
	overall		overall
*14:01	16	*05:01	13
*14:04	8	*05:02	11
*14:54	6	*05:03	16

Supplementary Table S5: Heterogeneity between studies in the analysis of individual alleles for pemphigus, TTP, MuSK MG and all diseases collectively with and without pemphigus

Disease	HLA allele	Allele/ genotype/ haplotype frequency	Tau^2	Chi^2	p	I^2
Low heterogeneity						
Pemphigus	*DRB1*03*	Genotype	0	13.92	0.46	0%
	*DRB1*07*	Allele	0.10	17.55	0.13	32%
	*DRB1*09*	Genotype	0	4.35	0.93	0%
	*DRB1*09*	Allele	0	1.96	0.58	0%
	*DRB1*10*	Genotype	0.21	14.13	0.17	29%
	*DRB1*10*	Allele	0.28	6.58	0.25	24%
	*DRB1*12*	Genotype	0.27	16.08	0.1	38%
	*DRB1*12*	Allele	0	6.34	0.5	0%
	*DRB1*14*	Allele	0.04	20.36	< 0.00001	26%
	*DRB1*15*	Genotype	0	9.6	0.65	0%
	*DRB1*15*	Allele	0	7.37	0.6	0%
	*DQB1*02*	Genotype	0.01	11.57	0.4	5%
	*DQB1*02*	Allele	0	9.54	0.39	6%
	*DQB1*04*	Allele	0.05	4.36	0.36	8%
	*DQB1*06*	Allele	0.01	9.89	0.36	9%
	*HLA-DRB1*14-DQB1*05*	Haplotype (n)	0	2.14	0.54	0%
	*HLA-DRB1*14-DQB1*05*	Haplotype (2n)	0	2.88	0.41	0%
	*HLA-DRB1*15-DQB1*06*	Haplotype (n)	0	0.99	0.32	0%
	*HLA-DRB1*15-DQB1*06*	Haplotype (2n)	0	0.22	0.9	0%
TTP	*DRB1*04*	Genotype	0	3.1	0.54	0%
	*DRB1*12*	Genotype	0	3.49	0.48	0%
	*DRB1*13*	Genotype	0	0.59	0.96	0%
	*DRB1*15*	Genotype	0.02	3.3	0.35	9%
MuSK MG	*DRB1*16*	Genotype	0	0.25	0.97	0%
	*HLA-DRB1*14-DQB1*05*	Haplotype (2n)	0.1	3.19	0.2	37%
	*HLA-DRB1*16-DQB1*05*	Haplotype (2n)	0	0.59	0.74	0%
High heterogeneity						
Pemphigus	*DRB1*01*	Allele	0.89	68.27	<0.00001	82%
	*DRB1*01*	Genotype	2.47	113.47	<0.00001	89%
	*DRB1*03*	Allele	1.11	64.33	<0.00001	83%
	*DRB1*04*	Allele	0.28	82.97	< 0.00001	78%
	*DRB1*04*	Genotype	0.27	57.38	<0.00001	67%
	*DRB1*07*	Genotype	0.26	22.41	0.03	46%
	*DRB1*08*	Allele	1.33	58.12	<0.00001	83%
	*DRB1*08*	Genotype	0.88	63.24	<0.00001	76%
	*DRB1*11*	Allele	0.29	26.61	0.003	62%
	*DRB1*11*	Genotype	0.43	43.57	< 0.0001	68%
	*DRB1*13*	Genotype	0.37	28.36	0.005	58%
	*DRB1*13*	Allele	0.12	16.98	0.07	41%
	*DRB1*14*	Genotype	1.09	134.89	< 0.00001	86%
	*DRB1*16*	Genotype	0.59	22.45	0.02	51%
	*DRB1*16*	Allele	1.6	26.39	0.002	77%

	*DQB1*03*	Genotype	0.89	87.57	< 0.00001	85%
	*DQB1*03*	Allele	0.34	99.95	<0.00001	87%
	*DQB1*04*	Genotype	0.7	23.18	0.006	61%
	*DQB1*05*	Genotype	0.79	80.26	<0.00001	84%
	*DQB1*05*	Allele	0.36	75.49	<0.00001	84%
	*DQB1*06*	Genotype	0.27	23.89	0.004	62%
	*HLA-DRB1*16-DQB1*05*	Haplotype (n)	0.82	9.74	0.008	79%
TTP	*DRB1*07*	Genotype	0.2	9.05	0.06	56%
	*DRB1*11*	Genotype	0.2	11.14	0.03	64%
	*DQB1*03*	Genotype	0.52	12.27	0.007	76%
MuSK MG	*DRB1*14*	Genotype	0.54	11.69	0.009	74%
	*DQB1*05*	Genotype	0.47	8.75	0.03	66%
Low/moderate heterogeneity						
All IgG4 AID collectively	*DRB1*07*	Genotype	0.33	43.26	0.0007	58%
	*DRB1*07*	Allele	0.26	32.37	0.006	54%
	*DRB1*09*	Genotype	0	9.47	0.92	0%
	*DRB1*09*	Allele	0	6.49	0.48	0%
	*DRB1*10*	Genotype	0.12	17.79	0.27	16%
	*DRB1*10*	Allele	0.46	12.86	0.17	30%
	*DRB1*11*	Allele	0.26	41.23	0.0009	59%
	*DRB1*12*	Genotype	0.17	23.48	0.13	28%
	*DRB1*12*	Allele	0	7.92	0.72	0%
	*DRB1*13*	Genotype	0.23	35.18	< 0.00001	46%
	*DRB1*13*	Allele	0.14	24.94	< 0.00001	40%
	*DQB1*02*	Allele	0.18	25.33	0.02	49%
	*DQB1*04*	Genotype	0.66	35.56	0.002	58%
	*DQB1*04*	Allele	0.41	17.98	0.02	56%
	*DRB1*14-DQB1*05*	Haplotype (2n)	0.03	8.42	0.3	17%
High heterogeneity						
All IgG4 AID collectively	*DRB1*01*	Genotype	2.57	216.32	< 0.00001	91%
	*DRB1*01*	Allele	0.72	75.91	< 0.00001	78%
	*DRB1*03*	Genotype	0.69	73.09	0.00001	70%
	*DRB1*03*	Allele	1.08	78.92	0.01	81%
	*DRB1*04*	Genotype	0.95	190.29	< 0.00001	86%
	*DRB1*04*	Allele	0.56	172.97	< 0.00001	86%
	*DRB1*08*	Genotype	0.76	75.62	< 0.00001	71%
	*DRB1*08*	Allele	0.92	69.89	< 0.00001	79%
	*DRB1*11*	Genotype	1.34	182.18	< 0.00001	88%
	*DRB1*14*	Genotype	1.03	172.69	< 0.00001	84%
	*DRB1*14*	Allele	0.29	62.24	< 0.00001	68%
	*DRB1*15*	Genotype	0.54	68.2	< 0.00001	72%
	*DRB1*15*	Allele	0.69	76.54	< 0.00001	82%
	*DRB1*16*	Genotype	0.68	52.7	< 0.00001	64%
	*DRB1*16*	Allele	1.35	42.94	< 0.00001	79%
	*DQB1*02*	Genotype	1.13	96.95	< 0.00001	83%
	*DQB1*03*	Genotype	0.76	110.59	< 0.00001	83%
	*DQB1*03*	Allele	0.33	130.94	< 0.00001	85%
	*DQB1*05*	Genotype	0.86	133.27	< 0.00001	84%
	*DQB1*05*	Allele	0.4	107.24	< 0.00001	84%
	*DQB1*06*	Genotype	0.26	41.37	0.0005	61%
	*DQB1*06*	Allele	0.55	93.06	< 0.00001	85%
	*DRB1*14-DQB1*05*	Haplotype (n)	0.47	15.93	0.01	62%

	*DRB1*16-DQB1*05*	Haplotype (n)	1	8.93	0.01	78%
	*DRB1*16-DQB1*05*	Haplotype (2n)	0.81	27.04	< 0.00001	82%
	*DRB1*15-DQB1*06*	Haplotype (n)	2.78	24.02	< 0.0001	88%
	*DRB1*15-DQB1*06*	Haplotype (2n)	0.92	35.05	< 0.00001	83%
Low/moderate heterogeneity						
IgG4-AID minus pemphigus	*DRB1*01*	Allele	0.1	5.19	0.27	23%
	*DRB1*04*	Genotype	0	3.35	0.76	0%
	*DRB1*04*	Allele	0.21	9.52	0.15	37%
	*DRB1*07*	Allele	0.24	3.92	0.14	49%
	*DRB1*08*	Genotype	0.55	12.4	0.05	52%
	*DRB1*08*	Allele	0.09	5.11	0.28	22%
	*DRB1*09*	Genotype	0	2.68	0.85	0%
	*DRB1*09*	Allele	0	1.16	0.76	0%
	*DRB1*10*	Genotype	0	3.78	0.44	0%
	*DRB1*10*	Allele	1.96	6.21	0.1	52%
	*DRB1*11*	Allele	0.05	7.46	0.28	20%
	*DRB1*12*	Genotype	0	4.23	0.65	0%
	*DRB1*12*	Allele	0	0.15	0.98	0%
	*DRB1*13*	Genotype	0	3.28	0.77	0%
	*DRB1*13*	Allele	0.49	7.8	0.1	49%
	*DRB1*16*	Allele	0.14	2.54	0.28	21%
	*DQB1*02*	Allele	0	1.58	0.67	0%
	*DQB1*04*	Genotype	0.86	11.54	0.04	57%
	*DQB1*06*	Genotype	0.11	9.31	0.16	36%
	*DRB1*14-DQB1*05*	Haplotype (n)	0	0.98	0.61	0%
	*DRB1*14-DQB1*05*	Haplotype (2n)	0.21	5.53	0.14	46%
	*DRB1*16-DQB1*05*	Haplotype (2n)	0	0.59	0.74	0%
High heterogeneity						
IgG4-AID minus pemphigus	*DRB1*01*	Genotype	3.26	98.76	< 0.00001	93%
	*DRB1*03*	Genotype	0.45	23.34	0.001	70%
	*DRB1*03*	Allele	1.34	11.08	0.01	73%
	*DRB1*07*	Genotype	0.28	13.68	0.02	63%
	*DRB1*11*	Genotype	0.45	25.33	0.0003	76%
	*DRB1*14*	Genotype	1.02	35.92	< 0.0001	78%
	*DRB1*14*	Allele	1.76	27	< 0.0001	85%
	*DRB1*15*	Genotype	0.43	18.07	0.006	67%
	*DRB1*15*	Allele	1.05	28.74	< 0.00001	86%
	*DRB1*16*	Genotype	0.6	21.71	0.003	68%
	*DQB1*02*	Genotype	1.47	41.58	< 0.00001	90%
	*DQB1*03*	Genotype	0.67	22.37	0.0004	78%
	*DQB1*03*	Allele	0.4	29.39	< 0.0001	80%
	*DQB1*04*	Allele	0.9	8.35	0.04	64%
	*DQB1*05*	Genotype	1.15	46.92	< 0.00001	85%
	*DQB1*05*	Allele	0.59	22.81	0.0001	82%
	*DQB1*06*	Allele	0.92	32.8	< 0.00001	88%
	*DRB1*16-DQB1*05*	Haplotype (n)	1	8.93	0.01	78%

	*DRB1*15-DQB1*06*	Haplotype (n)	0.53	2.55	0.11	61%
	*DRB1*15-DQB1*06*	Haplotype (2n)	1.18	16.05	0.001	81%

Legend for supplementary figures S1-S104:

† Study was included in the qualitative synthesis, but excluded from the meta-analysis as it did not fit all selection criteria.

‡ Study did not differentiate between disease subgroups of pemphigus or CIDP.

* Study was included after discussion with W.B..

** Study in which the same control group was used for pemphigus foliaceus and pemphigus vulgaris, here data were pooled for analysis.

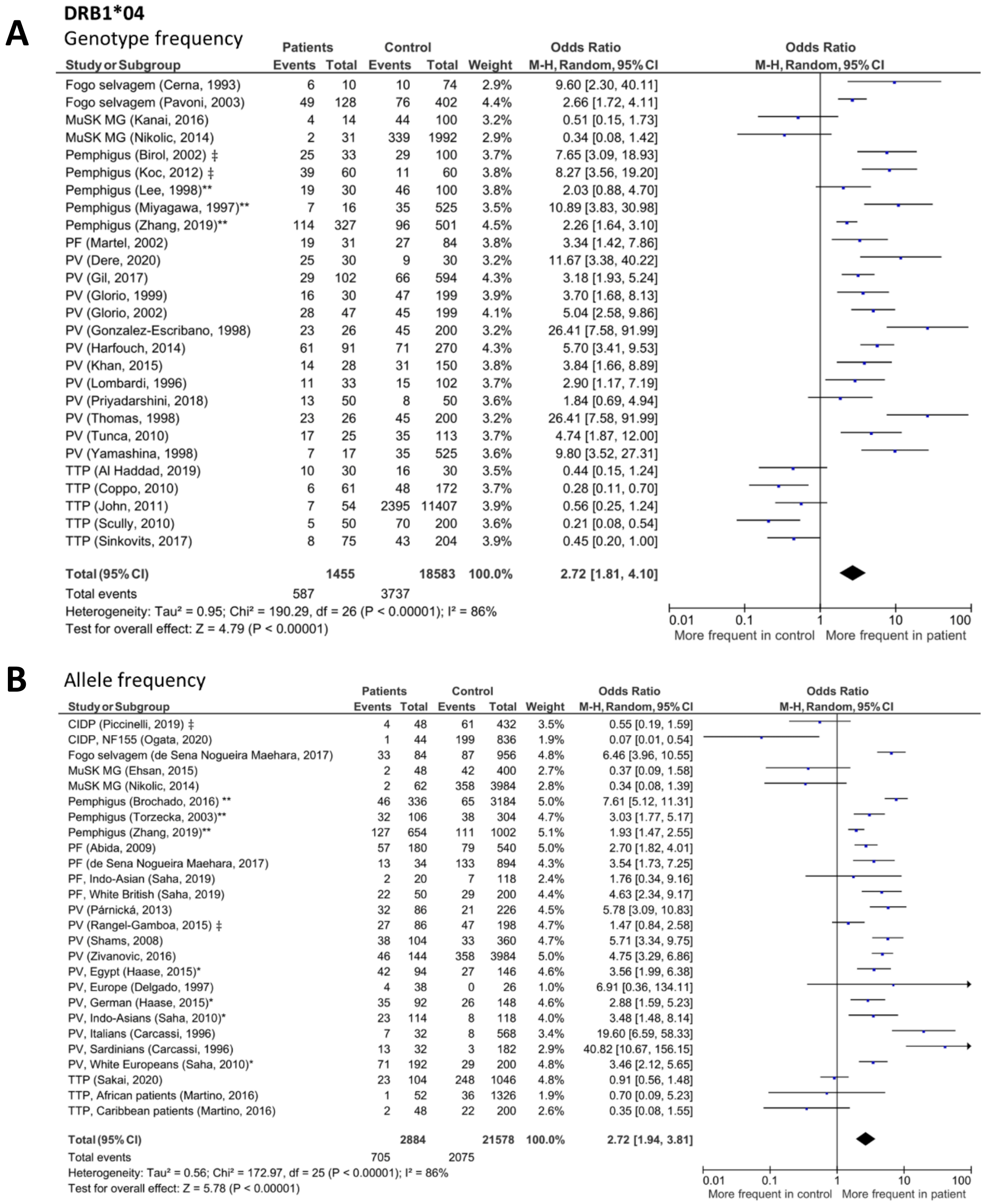

Figure S 1: Genotype (A) and allele (B) frequency of HLA-DRB1*04 in all diseases.

DQB1*03

A

Genotype frequency

Study or Subgroup	Patients Events	Patients Total	Control Events	Control Total	Weight	Odds Ratio M-H, Random, 95% CI
Fogo selvagem (Cerna, 1993)	8	10	63	74	3.5%	0.70 [0.13, 3.73]
MuSK MG (Kanai, 2016)	9	14	51	100	4.7%	1.73 [0.54, 5.52]
MuSK MG (Nikolic, 2014)	9	21	87	159	5.3%	0.62 [0.25, 1.56]
Pemphigus (Lee, 1998)**	17	30	64	100	5.6%	0.74 [0.32, 1.69]
Pemphigus (Miyagawa, 1997)**	8	16	90	525	5.1%	4.83 [1.77, 13.22]
Pemphigus (Zhang, 2019)**	241	327	365	501	6.6%	1.04 [0.76, 1.43]
PF (Martel, 2002)	13	30	13	64	5.3%	3.00 [1.17, 7.72]
PV (Dere, 2020)	29	30	22	30	2.7%	10.55 [1.23, 90.66]
PV (Gil, 2017)	51	102	159	594	6.5%	2.74 [1.78, 4.20]
PV (Glorio, 1999)	15	30	18	199	5.5%	10.06 [4.24, 23.86]
PV (Glorio, 2002)	25	47	18	199	5.8%	11.43 [5.40, 24.20]
PV (Lombardi, 1996)	28	33	68	102	5.0%	2.80 [0.99, 7.90]
PV (Orouji, 2014)	54	54	75	85	1.8%	15.16 [0.87, 264.26]
PV (Priyadarshini, 2018)	14	50	25	50	5.6%	0.39 [0.17, 0.89]
PV (Tunca, 2010)	21	25	71	113	4.8%	3.11 [1.00, 9.66]
PV (Yamashina, 1998)	9	17	90	525	5.2%	5.44 [2.04, 14.47]
TTP (Al Haddad, 2019)	28	30	26	30	3.3%	2.15 [0.36, 12.76]
TTP (Coppo, 2010)	46	60	92	172	6.0%	2.86 [1.46, 5.58]
TTP (John, 2011)	29	47	29	174	5.9%	8.06 [3.96, 16.39]
TTP (Scully, 2010)	36	50	129	200	5.9%	1.42 [0.72, 2.80]
**Total (95% CI)**		**1023**		**3996**	**100.0%**	**2.53 [1.61, 3.97]**
Total events	690		1555			

Heterogeneity: Tau² = 0.76; Chi² = 110.59, df = 19 (P < 0.00001); I² = 83%

Test for overall effect: Z = 4.05 (P < 0.0001)

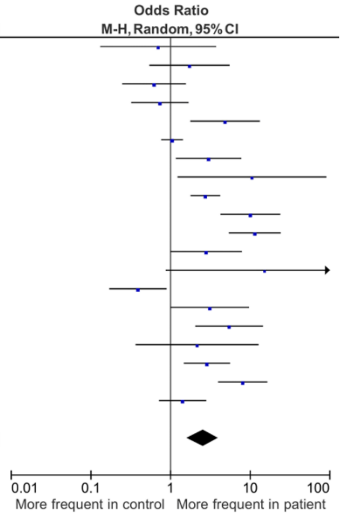

B

Allele frequency

Study or Subgroup	Patients Events	Patients Total	Control Events	Control Total	Weight	Odds Ratio M-H, Random, 95% CI
CIDP (Piccinelli, 2019) ‡	20	48	196	432	5.0%	0.86 [0.47, 1.57]
CIDP, NF155 (Ogata, 2020)	13	33	309	836	4.6%	1.11 [0.54, 2.26]
MuSK MG (Ehsan, 2015)	8	48	54	400	4.2%	1.28 [0.57, 2.88]
MuSK MG (Nikolic, 2014)	11	62	106	318	4.7%	0.43 [0.22, 0.86]
Pemphigus (Brochado, 2016) **	123	336	923	3184	6.1%	1.41 [1.12, 1.79]
Pemphigus (Zhang, 2019)**	309	654	529	1002	6.2%	0.80 [0.66, 0.98]
PF (Abida, 2009)	53	180	171	540	5.8%	0.90 [0.62, 1.30]
PF, Indo-Asian (Saha, 2019)	2	20	8	118	2.1%	1.53 [0.30, 7.78]
PF, White British (Saha, 2019)	18	50	18	200	4.4%	5.69 [2.68, 12.08]
PV (Orouji, 2014)	82	108	105	170	5.2%	1.95 [1.14, 3.35]
PV (Párnická, 2013)	45	86	74	226	5.3%	2.25 [1.36, 3.74]
PV (Shams, 2008)	58	104	138	360	5.6%	2.03 [1.30, 3.15]
PV (Zivanovic, 2016)	58	144	106	318	5.7%	1.35 [0.90, 2.03]
PV, Europe (Delgado, 1997)	4	38	6	26	2.6%	0.39 [0.10, 1.56]
PV, Indo-Asians (Saha, 2010)*	20	114	10	118	4.3%	2.30 [1.02, 5.15]
PV, Italians (Carcassi, 1996)	7	32	37	812	3.9%	5.86 [2.38, 14.44]
PV, Sardinians (Carcassi, 1996)	13	32	9	182	3.7%	13.15 [4.97, 34.80]
PV, White Europeans (Saha, 2010)*	66	192	31	200	5.4%	2.86 [1.76, 4.64]
TTP (Sakai, 2020)	37	104	378	1046	5.6%	0.98 [0.64, 1.49]
TTP, African patients (Martino, 2016)	17	52	82	874	4.9%	4.69 [2.52, 8.74]
TTP, Caribbean patients (Martino, 2016)	12	48	50	200	4.5%	1.00 [0.48, 2.07]
**Total (95% CI)**		**2485**		**11562**	**100.0%**	**1.65 [1.24, 2.19]**
Total events	976		3340			

Heterogeneity: Tau² = 0.33; Chi² = 130.94, df = 20 (P < 0.00001); I² = 85%

Test for overall effect: Z = 3.41 (P = 0.0007)

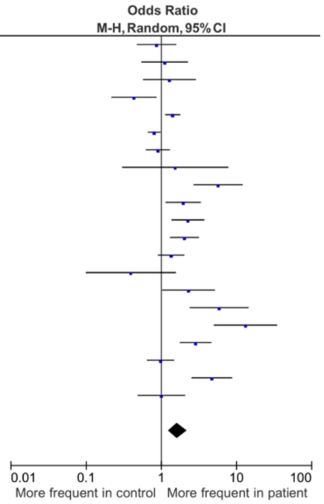

Figure S 2: Genotype (A) and allele (B) frequency of HLA-DQB1*03 in all diseases

A **DRB1*04**

Genotype frequency

Study or Subgroup	Patients Events	Patients Total	Control Events	Control Total	Weight	Odds Ratio M-H, Random, 95% CI
MuSK MG (Kanai, 2016)	4	14	44	100	8.9%	0.51 [0.15, 1.73]
MuSK MG (Nikolic, 2014)	2	31	339	1992	6.5%	0.34 [0.08, 1.42]
TTP (Al Haddad, 2019)	10	30	16	30	12.3%	0.44 [0.15, 1.24]
TTP (Coppo, 2010)	6	61	48	172	16.3%	0.28 [0.11, 0.70]
TTP (John, 2011)	7	54	2395	11407	21.2%	0.56 [0.25, 1.24]
TTP (Scully, 2010)	5	50	70	200	14.3%	0.21 [0.08, 0.54]
TTP (Sinkovits, 2017)	8	75	43	204	20.6%	0.45 [0.20, 1.00]
**Total (95% CI)**		**315**		**14105**	**100.0%**	**0.39 [0.27, 0.56]**
Total events	42		2955			

Heterogeneity: Tau² = 0.00; Chi² = 3.35, df = 6 (P = 0.76); I² = 0%

Test for overall effect: Z = 5.10 (P < 0.00001)

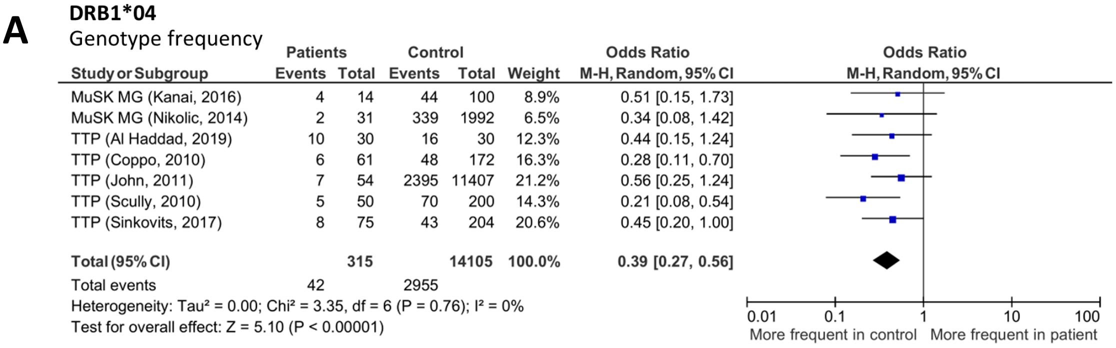

B Allele frequency

Study or Subgroup	Patients Events	Patients Total	Control Events	Control Total	Weight	Odds Ratio M-H, Random, 95% CI
CIDP (Piccinelli, 2019) ‡	4	48	61	432	17.7%	0.55 [0.19, 1.59]
CIDP, NF155 (Ogata, 2020)	1	44	199	836	7.2%	0.07 [0.01, 0.54]
MuSK MG (Ehsan, 2015)	2	48	42	400	11.8%	0.37 [0.09, 1.58]
MuSK MG (Nikolic, 2014)	2	62	358	3984	12.2%	0.34 [0.08, 1.39]
TTP (Sakai, 2020)	23	104	248	1046	32.6%	0.91 [0.56, 1.48]
TTP, African patients (Martino, 2016)	1	52	36	1326	7.1%	0.70 [0.09, 5.23]
TTP, Caribbean patients (Martino, 2016)	2	48	22	200	11.4%	0.35 [0.08, 1.55]
**Total (95% CI)**		**406**		**8224**	**100.0%**	**0.49 [0.27, 0.88]**
Total events	35		966			

Heterogeneity: Tau² = 0.21; Chi² = 9.52, df = 6 (P = 0.15); I² = 37%

Test for overall effect: Z = 2.39 (P = 0.02)

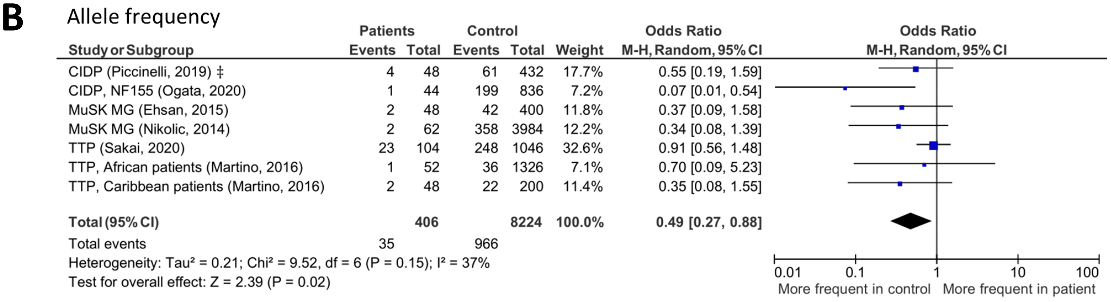

Figure S 3: Genotype (A) and allele (B) frequency of HLA-DRB1*04 in MuSK MG, TTP, CIDP

A **DRB1*13**

Genotype frequency

Study or Subgroup	Patients Events	Patients Total	Control Events	Control Total	Weight	Odds Ratio M-H, Random, 95% CI
MuSK MG (Kanai, 2016)	0	14	11	100	1.8%	0.27 [0.01, 4.81]
MuSK MG (Nikolic, 2014)	0	31	495	1992	1.9%	0.05 [0.00, 0.79]
TTP (Al Haddad, 2019)	8	30	12	30	12.5%	0.55 [0.18, 1.62]
TTP (Coppo, 2010)	9	61	55	172	24.6%	0.37 [0.17, 0.80]
TTP (John, 2011)	7	54	2749	11407	23.5%	0.47 [0.21, 1.04]
TTP (Scully, 2010)	6	50	43	200	17.6%	0.50 [0.20, 1.25]
TTP (Sinkovits, 2017)	6	75	39	204	18.1%	0.37 [0.15, 0.91]
**Total (95% CI)**		**315**		**14105**	**100.0%**	**0.41 [0.28, 0.61]**
Total events	36		3404			

Heterogeneity: Tau² = 0.00; Chi² = 3.28, df = 6 (P = 0.77); I² = 0%

Test for overall effect: Z = 4.50 (P < 0.00001)

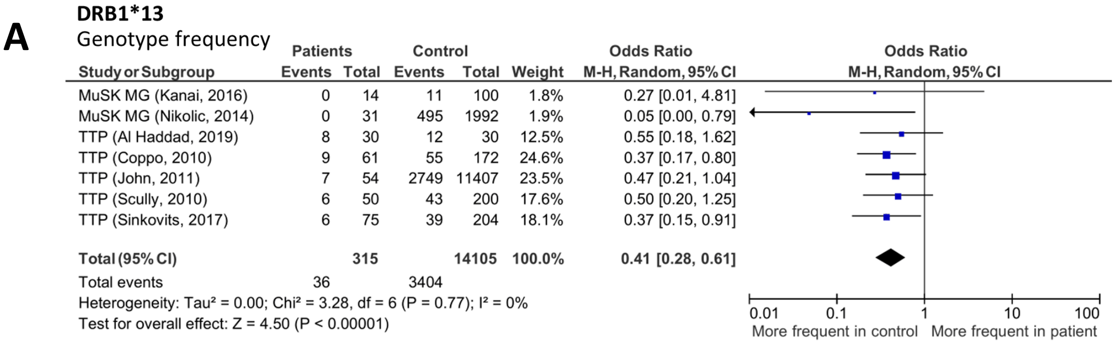

B Allele frequency

Study or Subgroup	Patients Events	Patients Total	Control Events	Control Total	Weight	Odds Ratio M-H, Random, 95% CI
CIDP (Piccinelli, 2019) ‡	7	48	54	432	31.7%	1.20 [0.51, 2.80]
CIDP, NF155 (Ogata, 2020)	1	44	70	836	14.0%	0.25 [0.03, 1.88]
MuSK MG (Ehsan, 2015)	2	48	34	400	20.6%	0.47 [0.11, 2.01]
MuSK MG (Nikolic, 2014)	0	62	527	3984	8.5%	0.05 [0.00, 0.85]
TTP (Sakai, 2020)	3	104	58	1046	25.2%	0.51 [0.16, 1.64]
**Total (95% CI)**		**306**		**6698**	**100.0%**	**0.49 [0.20, 1.21]**
Total events	13		743			

Heterogeneity: Tau² = 0.49; Chi² = 7.80, df = 4 (P = 0.10); I² = 49%

Test for overall effect: Z = 1.55 (P = 0.12)

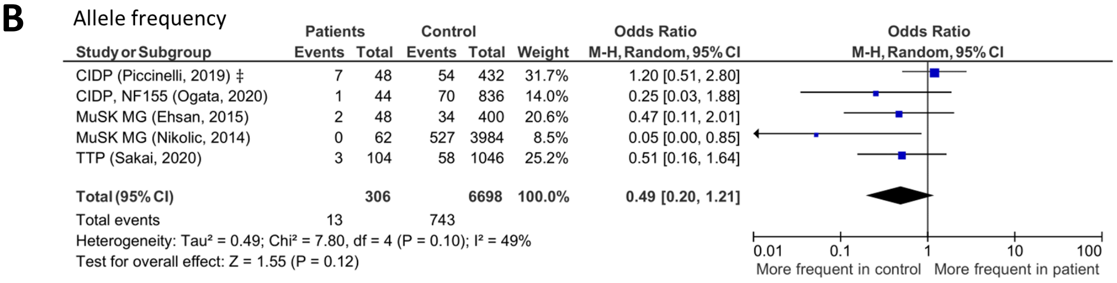

Figure S 4: Genotype (A) and allele (B) frequency of HLA-DRB1*13 in MuSK MG, TTP, CIDP

A

DRB1*14

Genotype frequency

Study or Subgroup	Patient Events	Patient Total	Control Events	Control Total	Weight	Odds Ratio M-H, Random, 95% CI
MuSK MG (Alahgholi-Hajibehzad, 2013)	14	48	29	250	13.8%	3.14 [1.51, 6.53]
MuSK MG (Kanai, 2016)	8	14	18	100	11.6%	6.07 [1.88, 19.67]
MuSK MG (Nikolic, 2014)	11	31	214	1992	13.7%	4.57 [2.16, 9.67]
MuSK MG (Niks, 2006)	12	23	127	2440	13.3%	19.87 [8.60, 45.90]
TTP (Al Haddad, 2019)	14	30	0	30	5.0%	53.61 [3.00, 956.98]
TTP (Coppo, 2010)	2	61	10	172	9.7%	0.55 [0.12, 2.58]
TTP (John, 2011)	2	54	696	11407	10.4%	0.59 [0.14, 2.44]
TTP (Scully, 2010)	2	50	7	200	9.5%	1.15 [0.23, 5.71]
TTP (Sinkovits, 2017)	10	75	12	204	13.1%	2.46 [1.02, 5.96]
**Total (95% CI)**		**386**		**16795**	**100.0%**	**3.29 [1.50, 7.19]**
Total events	75		1113			

Heterogeneity: Tau² = 1.02; Chi² = 35.92, df = 8 (P < 0.0001); I² = 78%

Test for overall effect: Z = 2.98 (P = 0.003)

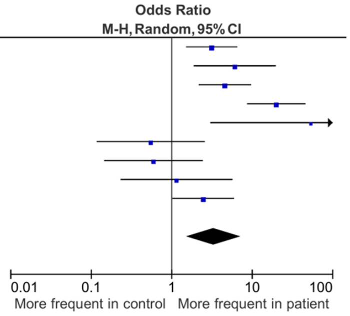

B

Allele frequency

Study or Subgroup	Patient Events	Patient Total	Control Events	Control Total	Weight	Odds Ratio M-H, Random, 95% CI
CIDP (Piccinelli, 2019) ‡	2	48	16	432	19.3%	1.13 [0.25, 5.07]
CIDP, NF155 (Ogata, 2020)	0	44	71	836	11.9%	0.12 [0.01, 1.97]
MuSK MG (Ehsan, 2015)	9	48	22	400	23.3%	3.97 [1.71, 9.21]
MuSK MG (Nikolic, 2014)	11	62	216	3984	24.1%	3.76 [1.93, 7.32]
TTP (Sakai, 2020)	3	104	98	1046	21.4%	0.29 [0.09, 0.92]
**Total (95% CI)**		**306**		**6698**	**100.0%**	**1.16 [0.31, 4.32]**
Total events	25		423			

Heterogeneity: Tau² = 1.76; Chi² = 27.00, df = 4 (P < 0.0001); I² = 85%

Test for overall effect: Z = 0.21 (P = 0.83)

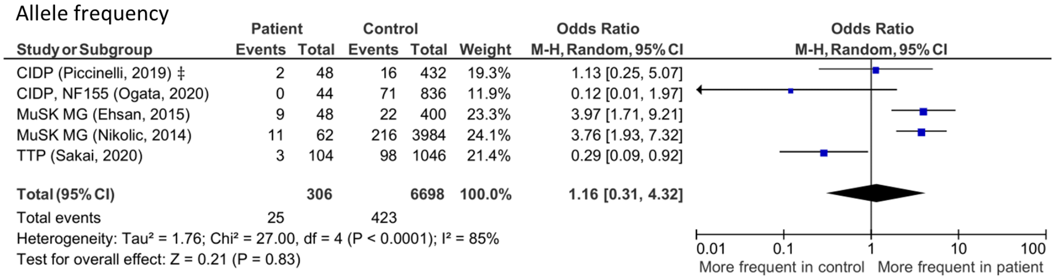

Figure S 5: Genotype (A) and allele (B) frequency of HLA-DRB1*14 in MuSK MG, TTP, CIDP

A

DQB1*05

Genotype frequency

Study or Subgroup	Patient Events	Patient Total	Control Events	Control Total	Weight	Odds Ratio M-H, Random, 95% CI
MuSK MG (Alahgholi-Hajibehzad, 2013)	35	48	90	250	14.4%	4.79 [2.41, 9.51]
MuSK MG (Kanai, 2016)	11	14	30	100	11.3%	8.56 [2.23, 32.88]
MuSK MG (Nikolic, 2014)	26	31	87	159	13.0%	4.30 [1.57, 11.78]
MuSK MG (Niks, 2006)	18	23	300	2440	13.0%	25.68 [9.46, 69.68]
TTP (Al Haddad, 2019)	0	30	3	30	5.2%	0.13 [0.01, 2.61]
TTP (Coppo, 2010)	19	60	55	172	14.7%	0.99 [0.52, 1.85]
TTP (John, 2011)	12	54	20	174	14.0%	2.20 [1.00, 4.86]
TTP (Scully, 2010)	13	50	51	200	14.4%	1.03 [0.51, 2.08]
**Total (95% CI)**		**310**		**3525**	**100.0%**	**2.96 [1.28, 6.86]**
Total events	134		636			

Heterogeneity: Tau² = 1.15; Chi² = 46.92, df = 7 (P < 0.00001); I² = 85%

Test for overall effect: Z = 2.54 (P = 0.01)

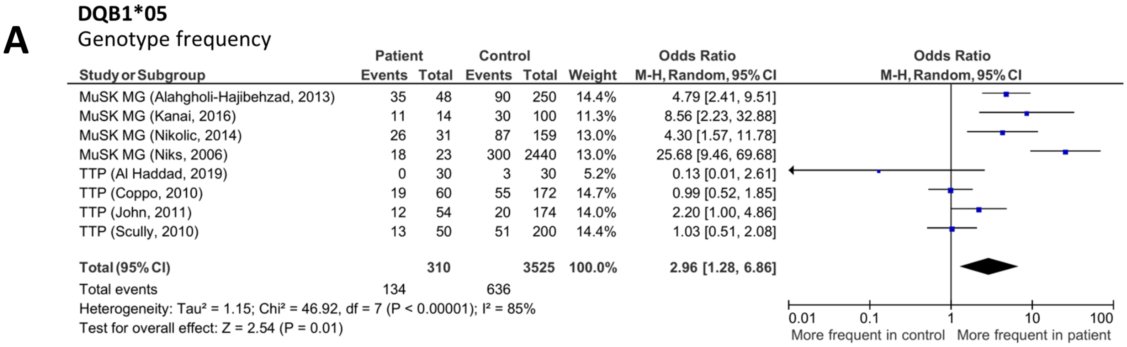

B

Allele frequency

Study or Subgroup	Patient Events	Patient Total	Control Events	Control Total	Weight	Odds Ratio M-H, Random, 95% CI
CIDP (Piccinelli, 2019) ‡	12	48	83	432	21.0%	1.40 [0.70, 2.81]
CIDP, NF155 (Ogata, 2020)	2	44	110	836	13.4%	0.31 [0.08, 1.32]
MuSK MG (Ehsan, 2015)	28	48	90	400	21.8%	4.82 [2.59, 8.96]
MuSK MG (Nikolic, 2014)	32	62	105	318	22.4%	2.16 [1.25, 3.75]
TTP (Sakai, 2020)	11	104	136	1046	21.4%	0.79 [0.41, 1.52]
**Total (95% CI)**		**306**		**3032**	**100.0%**	**1.46 [0.68, 3.13]**
Total events	85		524			

Heterogeneity: Tau² = 0.59; Chi² = 22.81, df = 4 (P = 0.0001); I² = 82%

Test for overall effect: Z = 0.98 (P = 0.33)

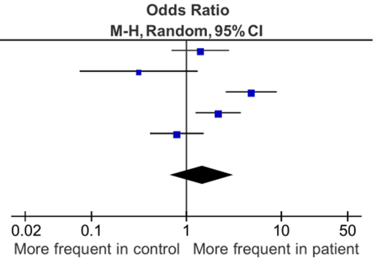

Figure S 6: Genotype (A) and allele (B) frequency of HLA-DQB1*05 in MuSK MG, TTP, CIDP

DRB1*14-DQB1*05

n

Study or Subgroup	Patients Events	Patients Total	Control Events	Control Total	Weight	Odds Ratio M-H, Random, 95% CI
MuSK MG (Alahgholi-Hajibehzad, 2013)	13	48	28	250	49.0%	2.94 [1.39, 6.22]
MuSK MG (Kanai, 2016)	6	14	13	100	18.8%	5.02 [1.50, 16.80]
TTP (Sinkovits, 2017)	10	75	10	162	32.2%	2.34 [0.93, 5.89]
**Total (95% CI)**		**137**		**512**	**100.0%**	**3.02 [1.79, 5.10]**
Total events	29		51			

Heterogeneity: Tau² = 0.00; Chi² = 0.98, df = 2 (P = 0.61); I² = 0%
Test for overall effect: Z = 4.14 (P < 0.0001)

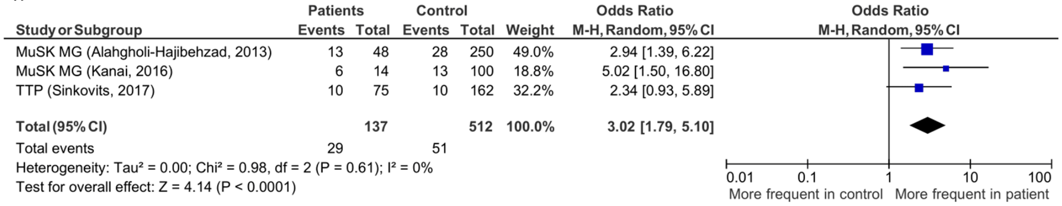

2n

Study or Subgroup	Patients Events	Patients Total	Control Events	Control Total	Weight	Odds Ratio M-H, Random, 95% CI
CIDP, NF155 (Ogata, 2020)	0	44	16	836	5.2%	0.56 [0.03, 9.46]
MuSK MG (Ehsan, 2015)	9	48	22	400	30.5%	3.97 [1.71, 9.21]
MuSK MG (Nikolic, 2014)	11	62	20	318	32.2%	3.21 [1.45, 7.11]
MuSK MG (Niks, 2006)	11	46	23	642	32.1%	8.46 [3.82, 18.73]
**Total (95% CI)**		**200**		**2196**	**100.0%**	**4.27 [2.17, 8.39]**
Total events	31		81			

Heterogeneity: Tau² = 0.21; Chi² = 5.53, df = 3 (P = 0.14); I² = 46%
Test for overall effect: Z = 4.21 (P < 0.0001)

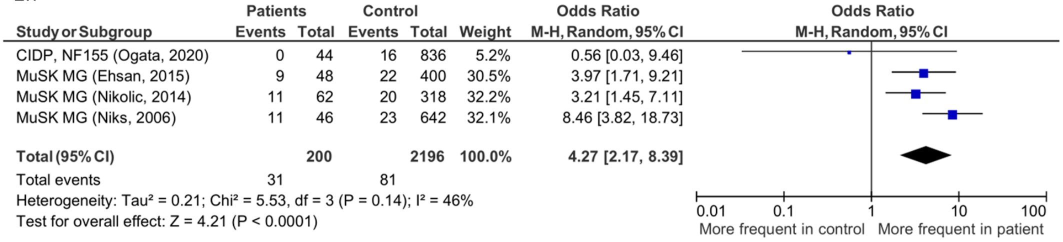

Figure S 7: Haplotype frequency of HLA-DRB1*14-DQB1*05 in MuSK MG, TTP, CIDP. n, calculations similar to the genotype frequency by dividing the number of individuals with a specific haplotype by the number of total individuals; 2n calculations similar to the allele frequency by dividing the total number of a specific haplotype by the total number of alleles in the cohort.

A

DRB1*03

Genotype frequency

Study or Subgroup	Patients Events	Patients Total	Control Events	Control Total	Weight	Odds Ratio M-H, Random, 95% CI
Fogo selvagem (Pavoni, 2003)	14	128	71	402	6.3%	0.57 [0.31, 1.06]
MuSK MG (Alahgholi-Hajibehzad, 2013)	1	48	44	250	2.9%	0.10 [0.01, 0.74]
MuSK MG (Nikolic, 2014)	5	31	418	1992	5.3%	0.72 [0.28, 1.90]
MuSK MG (Niks, 2006)	2	23	21	2440	3.9%	10.97 [2.42, 49.79]
Pemphigus (Lee, 1998)**	0	30	5	100	1.7%	0.28 [0.02, 5.30]
Pemphigus (Zhang, 2019)**	4	327	39	501	5.1%	0.15 [0.05, 0.41]
PF (Martel, 2002)	2	31	18	84	3.8%	0.25 [0.06, 1.16]
PV (Dere, 2020)	1	30	8	30	2.6%	0.09 [0.01, 0.82]
PV (Gil, 2017)	4	102	66	594	5.1%	0.33 [0.12, 0.92]
PV (Glorio, 1999)	2	30	42	199	4.0%	0.27 [0.06, 1.17]
PV (Glorio, 2002)	3	47	42	199	4.6%	0.25 [0.08, 0.86]
PV (Gonzalez-Escribano, 1998)	1	26	42	200	2.8%	0.15 [0.02, 1.14]
PV (Harfouch, 2014)	2	91	40	270	4.0%	0.13 [0.03, 0.55]
PV (Khan, 2015)	4	28	31	150	4.9%	0.64 [0.21, 1.98]
PV (Lombardi, 1996)	4	33	15	102	4.7%	0.80 [0.25, 2.60]
PV (Priyadarshini, 2018)	1	50	2	50	2.2%	0.49 [0.04, 5.58]
PV (Thomas, 1998)	1	26	42	200	2.8%	0.15 [0.02, 1.14]
PV (Tunca, 2010)	2	25	26	113	3.9%	0.29 [0.06, 1.32]
TTP (Al Haddad, 2019)	18	30	8	30	5.0%	4.13 [1.39, 12.27]
TTP (Coppo, 2010)	10	61	32	172	5.9%	0.86 [0.39, 1.87]
TTP (John, 2011)	15	54	2281	11407	6.3%	1.54 [0.85, 2.80]
TTP (Scully, 2010)	11	50	50	200	6.0%	0.85 [0.40, 1.78]
TTP (Sinkovits, 2017)	18	75	41	204	6.3%	1.26 [0.67, 2.36]
**Total (95% CI)**		**1376**		**19889**	**100.0%**	**0.54 [0.35, 0.83]**
Total events	125		3384			

Heterogeneity: Tau² = 0.69; Chi² = 73.09, df = 22 (P < 0.00001); I² = 70%

Test for overall effect: Z = 2.80 (P = 0.005)

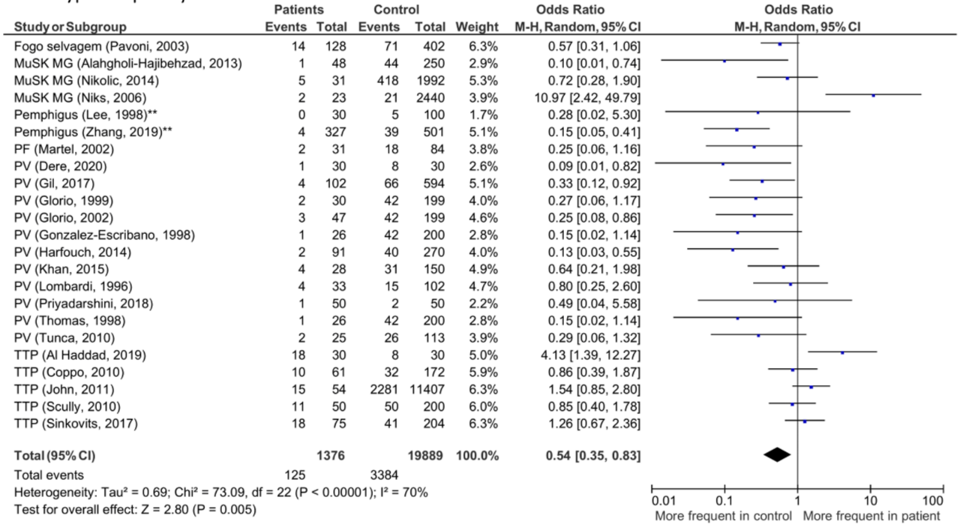

B

Allele frequency

Study or Subgroup	Patients Events	Patients Total	Control Events	Control Total	Weight	Odds Ratio M-H, Random, 95% CI
CIDP (Piccinelli, 2019) ‡	6	48	17	432	7.4%	3.49 [1.30, 9.32]
CIDP, NF155 (Ogata, 2020)	0	44	1	836	2.6%	6.26 [0.25, 155.82]
MuSK MG (Ehsan, 2015)	1	48	40	400	4.6%	0.19 [0.03, 1.43]
MuSK MG (Nikolic, 2014)	5	62	433	3984	7.5%	0.72 [0.29, 1.80]
Pemphigus (Torzecka, 2003)**	11	106	31	304	8.0%	1.02 [0.49, 2.11]
Pemphigus (Zhang, 2019)**	4	654	39	1002	7.2%	0.15 [0.05, 0.43]
PF (Abida, 2009)	46	180	79	540	8.7%	2.00 [1.33, 3.02]
PV (Párnická, 2013)	4	86	18	226	7.0%	0.56 [0.19, 1.72]
PV (Shams, 2008)	2	104	32	360	6.0%	0.20 [0.05, 0.85]
PV (Zivanovic, 2016)	6	144	433	3984	7.8%	0.36 [0.16, 0.81]
PV, Egypt (Haase, 2015)*	7	94	18	146	7.5%	0.57 [0.23, 1.43]
PV, German (Haase, 2015)*	4	92	21	148	7.0%	0.27 [0.09, 0.83]
PV, Indo-Asians (Saha, 2010)*	1	114	17	118	4.5%	0.05 [0.01, 0.40]
PV, Italians (Carcassi, 1996)	0	32	59	568	3.1%	0.13 [0.01, 2.18]
PV, Sardinians (Carcassi, 1996)	0	32	45	182	3.1%	0.05 [0.00, 0.77]
PV, White Europeans (Saha, 2010)*	8	192	29	200	7.8%	0.26 [0.11, 0.58]
**Total (95% CI)**		**2032**		**13430**	**100.0%**	**0.46 [0.25, 0.84]**
Total events	105		1312			

Heterogeneity: Tau² = 1.08; Chi² = 78.92, df = 15 (P < 0.00001); I² = 81%

Test for overall effect: Z = 2.50 (P = 0.01)

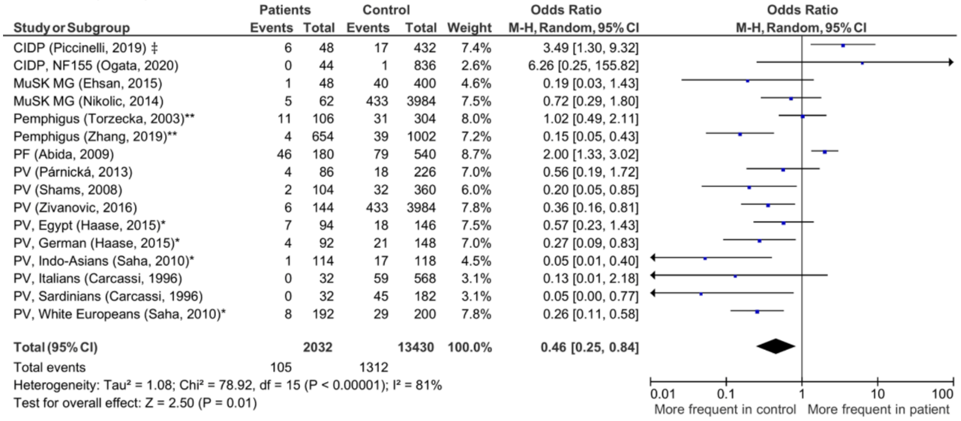

Figure S 8: Genotype (A) and allele (B) frequency of HLA-DRB1*03 in all diseases

A

DRB1*07

Genotype frequency

Study or Subgroup	Patients Events	Patients Total	Control Events	Control Total	Weight	Odds Ratio M-H, Random, 95% CI
Fogo selvagem (Pavoni, 2003)	3	128	83	402	4.8%	0.09 [0.03, 0.30]
MuSK MG (Nikolic, 2014)	7	31	276	1992	6.3%	1.81 [0.77, 4.25]
Pemphigus (Lee, 1998)**	0	30	12	100	1.3%	0.12 [0.01, 2.02]
Pemphigus (Zhang, 2019)**	17	327	26	501	7.6%	1.00 [0.53, 1.88]
PF (Martel, 2002)	5	31	24	84	5.2%	0.48 [0.17, 1.40]
PV (Dere, 2020)	1	30	5	30	2.0%	0.17 [0.02, 1.58]
PV (Gil, 2017)	8	102	80	594	6.8%	0.55 [0.26, 1.17]
PV (Gonzalez-Escribano, 1998)	4	26	72	200	5.1%	0.32 [0.11, 0.97]
PV (Harfouch, 2014)	6	91	55	270	6.2%	0.28 [0.11, 0.66]
PV (Khan, 2015)	4	28	46	150	5.0%	0.38 [0.12, 1.15]
PV (Lombardi, 1996)	1	33	29	102	2.3%	0.08 [0.01, 0.60]
PV (Priyadarshini, 2018)	8	50	14	50	5.7%	0.49 [0.18, 1.30]
PV (Thomas, 1998)	4	26	72	200	5.1%	0.32 [0.11, 0.97]
PV (Tunca, 2010)	3	25	14	113	4.1%	0.96 [0.26, 3.65]
TTP (Al Haddad, 2019)	7	30	9	30	4.8%	0.71 [0.22, 2.25]
TTP (Coppo, 2010)	12	61	44	172	7.1%	0.71 [0.35, 1.46]
TTP (John, 2011)	15	54	2692	11407	7.7%	1.25 [0.69, 2.26]
TTP (Scully, 2010)	7	50	59	200	6.3%	0.39 [0.17, 0.91]
TTP (Sinkovits, 2017)	8	75	55	204	6.6%	0.32 [0.15, 0.72]
**Total (95% CI)**		**1228**		**16801**	**100.0%**	**0.49 [0.34, 0.69]**
Total events	120		3667			

Heterogeneity: Tau² = 0.33; Chi² = 43.26, df = 18 (P = 0.0007); I² = 58%

Test for overall effect: Z = 3.99 (P < 0.0001)

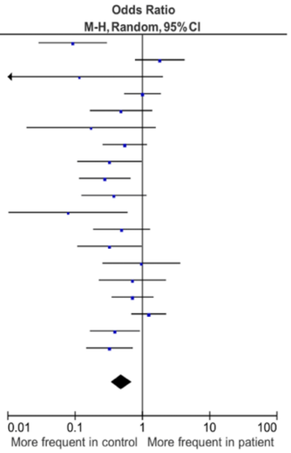

B

Allele frequency

Study or Subgroup	Patients Events	Patients Total	Control Events	Control Total	Weight	Odds Ratio M-H, Random, 95% CI
CIDP (Piccinelli, 2019) ‡	3	48	57	432	5.1%	0.44 [0.13, 1.46]
Fogo selvagem (de Sena Nogueira Maehara, 2017)	2	84	128	956	4.2%	0.16 [0.04, 0.65]
MuSK MG (Ehsan, 2015)	5	48	26	400	6.2%	1.67 [0.61, 4.58]
MuSK MG (Nikolic, 2014)	7	62	284	3984	7.7%	1.66 [0.75, 3.67]
Pemphigus (Brochado, 2016) **	11	336	342	3184	9.2%	0.28 [0.15, 0.52]
Pemphigus (Torzecka, 2003)**	4	106	44	304	6.0%	0.23 [0.08, 0.66]
Pemphigus (Zhang, 2019)**	17	654	26	1002	9.1%	1.00 [0.54, 1.86]
PV (Párnická, 2013)	4	86	33	226	5.9%	0.29 [0.10, 0.83]
PV (Rangel-Gamboa, 2015) ‡	4	86	22	198	5.7%	0.39 [0.13, 1.17]
PV (Shams, 2008)	2	104	26	360	4.0%	0.25 [0.06, 1.08]
PV (Zivanovic, 2016)	7	144	284	3984	7.9%	0.67 [0.31, 1.44]
PV, Egypt (Haase, 2015)*	4	94	17	146	5.6%	0.34 [0.11, 1.04]
PV, Europe (Delgado, 1997)	1	38	1	26	1.4%	0.68 [0.04, 11.31]
PV, German (Haase, 2015)*	7	92	13	148	6.5%	0.86 [0.33, 2.23]
PV, Indo-Asians (Saha, 2010)*	8	114	14	118	6.9%	0.56 [0.23, 1.39]
PV, White Europeans (Saha, 2010)*	14	192	29	200	8.7%	0.46 [0.24, 0.91]
**Total (95% CI)**		**2288**		**15668**	**100.0%**	**0.52 [0.37, 0.74]**
Total events	100		1346			

Heterogeneity: Tau² = 0.26; Chi² = 32.37, df = 15 (P = 0.006); I² = 54%

Test for overall effect: Z = 3.60 (P = 0.0003)

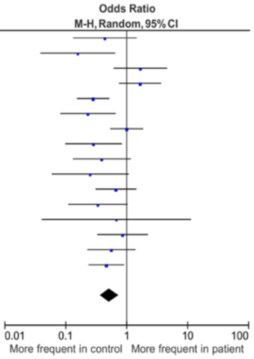

Figure S 9: Genotype (A) and allele (B) frequency of HLA-DRB1*07 in all diseases

A

DRB1*09

Genotype frequency

Study or Subgroup	Patients Events	Patients Total	Control Events	Control Total	Weight	Odds Ratio M-H, Random, 95% CI
Fogo selvagem (Pavoni, 2003)	1	128	11	402	1.8%	0.28 [0.04, 2.19]
MuSK MG (Kanai, 2016)	3	14	23	100	4.1%	0.91 [0.23, 3.55]
MuSK MG (Nikolic, 2014)	0	31	14	1992	0.9%	2.17 [0.13, 37.11]
Pemphigus (Lee, 1998)**	0	30	20	100	0.9%	0.06 [0.00, 1.10]
Pemphigus (Zhang, 2019)**	79	327	175	501	77.0%	0.59 [0.43, 0.81]
PF (Martel, 2002)	1	31	1	84	1.0%	2.77 [0.17, 45.64]
PV (Gil, 2017)	1	102	9	594	1.7%	0.64 [0.08, 5.13]
PV (Gonzalez-Escribano, 1998)	0	26	6	200	0.9%	0.56 [0.03, 10.31]
PV (Harfouch, 2014)	0	91	2	270	0.8%	0.59 [0.03, 12.34]
PV (Khan, 2015)	0	28	6	150	0.9%	0.39 [0.02, 7.12]
PV (Priyadarshini, 2018)	0	50	1	50	0.7%	0.33 [0.01, 8.21]
PV (Thomas, 1998)	0	26	6	200	0.9%	0.56 [0.03, 10.31]
PV (Tunca, 2010)	0	25	4	113	0.9%	0.48 [0.02, 9.15]
TTP (Al Haddad, 2019)	2	30	0	30	0.8%	5.35 [0.25, 116.31]
TTP (Coppo, 2010)	2	61	3	172	2.3%	1.91 [0.31, 11.71]
TTP (John, 2011)	1	54	217	11407	1.9%	0.97 [0.13, 7.07]
TTP (Scully, 2010)	1	50	6	200	1.6%	0.66 [0.08, 5.61]
TTP (Sinkovits, 2017)	0	75	4	204	0.9%	0.30 [0.02, 5.55]
**Total (95% CI)**		**1179**		**16769**	**100.0%**	**0.62 [0.47, 0.82]**
Total events	91		508			

Heterogeneity: Tau² = 0.00; Chi² = 9.47, df = 17 (P = 0.92); I² = 0%

Test for overall effect: Z = 3.37 (P = 0.0008)

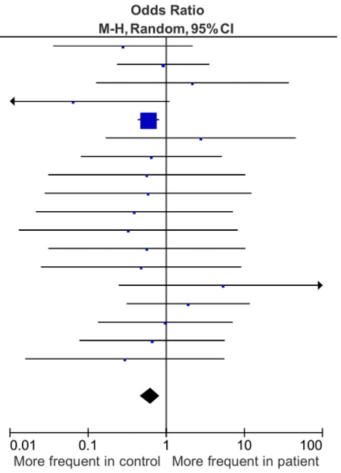

B

Allele frequency

Study or Subgroup	Patients Events	Patients Total	Control Events	Control Total	Weight	Odds Ratio M-H, Random, 95% CI
CIDP, NF155 (Ogata, 2020)	5	44	127	836	6.1%	0.72 [0.28, 1.85]
MuSK MG (Ehsan, 2015)	1	48	12	400	1.3%	0.69 [0.09, 5.41]
MuSK MG (Nikolic, 2014)	0	62	14	3984	0.7%	2.19 [0.13, 37.13]
Pemphigus (Torzecka, 2003)**	2	106	3	304	1.7%	1.93 [0.32, 11.71]
Pemphigus (Zhang, 2019)**	82	654	195	1002	70.2%	0.59 [0.45, 0.78]
PV (Párnická, 2013)	0	86	0	226		Not estimable
PV (Shams, 2008)	2	104	7	360	2.2%	0.99 [0.20, 4.83]
PV, Egypt (Haase, 2015)*	0	94	0	146		Not estimable
PV, German (Haase, 2015)*	0	92	1	148	0.5%	0.53 [0.02, 13.19]
TTP (Sakai, 2020)	16	104	142	1046	17.4%	1.16 [0.66, 2.03]
**Total (95% CI)**		**1394**		**8452**	**100.0%**	**0.70 [0.56, 0.89]**
Total events	108		501			

Heterogeneity: Tau² = 0.00; Chi² = 6.49, df = 7 (P = 0.48); I² = 0%

Test for overall effect: Z = 2.96 (P = 0.003)

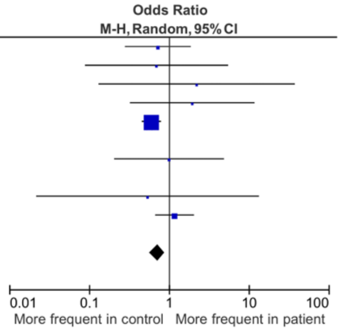

Figure S 10: Genotype (A) and allele (B) frequency of HLA-DRB1*09 in all diseases

A

DQB1*02

Genotype frequency

Study or Subgroup	Patients Events	Patients Total	Control Events	Control Total	Weight	Odds Ratio M-H, Random, 95% CI
Fogo selvagem (Moraes, 1991)	0	37	11	49	2.7%	0.04 [0.00, 0.78]
MuSK MG (Nikolic, 2014)	12	31	48	159	6.8%	1.46 [0.66, 3.24]
Pemphigus (Birol, 2002) ‡	1	33	26	100	4.0%	0.09 [0.01, 0.68]
Pemphigus (Koc, 2012) ‡	8	60	17	60	6.5%	0.39 [0.15, 0.99]
Pemphigus (Lee, 1998)**	1	30	21	100	4.0%	0.13 [0.02, 1.01]
Pemphigus (Zhang, 2019)**	21	327	58	501	7.4%	0.52 [0.31, 0.88]
PF (Martel, 2002)	6	30	28	64	6.3%	0.32 [0.12, 0.89]
PV (Dere, 2020)	2	30	14	30	4.9%	0.08 [0.02, 0.41]
PV (Gil, 2017)	9	102	134	594	7.0%	0.33 [0.16, 0.68]
PV (Lombardi, 1996)	3	33	35	102	5.8%	0.19 [0.05, 0.67]
PV (Orouji, 2014)	7	54	23	85	6.6%	0.40 [0.16, 1.01]
PV (Priyadarshini, 2018)	4	50	11	50	5.8%	0.31 [0.09, 1.05]
PV (Tunca, 2010)	3	25	37	113	5.7%	0.28 [0.08, 1.00]
TTP (Al Haddad, 2019)	14	30	2	30	4.9%	12.25 [2.46, 60.91]
TTP (Coppo, 2010)	19	60	68	172	7.2%	0.71 [0.38, 1.32]
TTP (John, 2011)	26	50	19	174	7.0%	8.84 [4.25, 18.36]
TTP (Scully, 2010)	16	50	86	200	7.1%	0.62 [0.32, 1.20]
**Total (95% CI)**		**1032**		**2583**	**100.0%**	**0.50 [0.28, 0.89]**
Total events	152		638			

Heterogeneity: $Tau^2 = 1.13$; $Chi^2 = 96.95$, df = 16 ($P < 0.00001$); $I^2 = 83\%$

Test for overall effect: Z = 2.34 (P = 0.02)

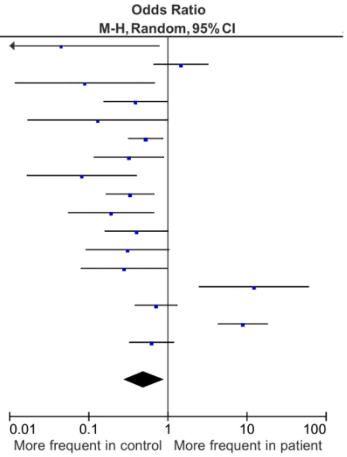

B

Allele frequency

Study or Subgroup	Patients Events	Patients Total	Control Events	Control Total	Weight	Odds Ratio M-H, Random, 95% CI
CIDP (Piccinelli, 2019) ‡	8	48	73	432	8.5%	0.98 [0.44, 2.19]
CIDP, NF155 (Ogata, 2020)	0	44	1	836	1.0%	6.26 [0.25, 155.82]
MuSK MG (Ehsan, 2015)	8	48	76	400	8.5%	0.85 [0.38, 1.90]
MuSK MG (Nikolic, 2014)	12	62	54	318	9.6%	1.17 [0.59, 2.35]
Pemphigus (Zhang, 2019)**	21	654	60	1002	12.0%	0.52 [0.31, 0.86]
PV (Orouji, 2014)	8	108	28	170	8.2%	0.41 [0.18, 0.93]
PV (Párnická, 2013)	8	86	44	226	8.5%	0.42 [0.19, 0.94]
PV (Shams, 2008)	3	104	69	360	5.4%	0.13 [0.04, 0.41]
PV (Zivanovic, 2016)	12	144	54	318	10.1%	0.44 [0.23, 0.86]
PV, Europe (Delgado, 1997)	2	38	45	496	4.0%	0.56 [0.13, 2.39]
PV, Indo-Asians (Saha, 2010)*	7	114	10	118	6.6%	0.71 [0.26, 1.92]
PV, Italians (Carcassi, 1996)	0	32	163	812	1.3%	0.06 [0.00, 1.00]
PV, Sardinians (Carcassi, 1996)	3	32	53	182	5.1%	0.25 [0.07, 0.86]
PV, White Europeans (Saha, 2010)*	19	192	51	200	11.2%	0.32 [0.18, 0.57]
**Total (95% CI)**		**1706**		**5870**	**100.0%**	**0.51 [0.36, 0.71]**
Total events	111		781			

Heterogeneity: $Tau^2 = 0.18$; $Chi^2 = 25.33$, df = 13 ($P = 0.02$); $I^2 = 49\%$

Test for overall effect: Z = 3.99 (P < 0.0001)

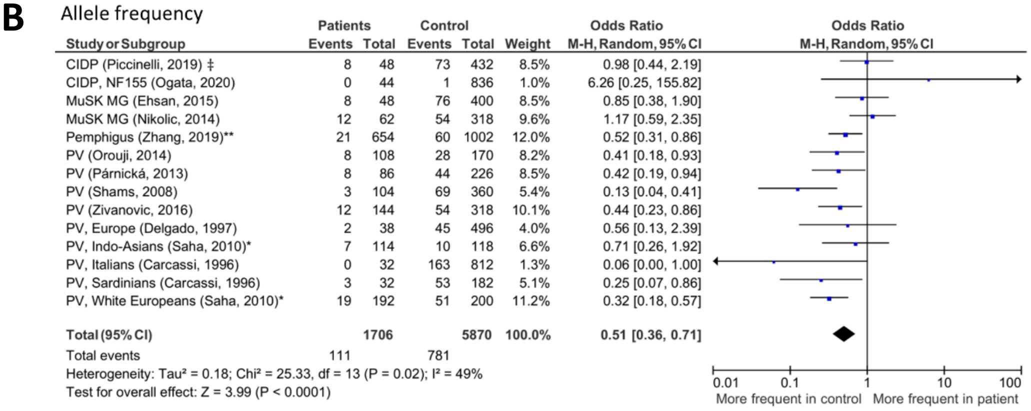

Figure S 11: Genotype (A) and allele (B) frequency of HLA-DQB1*02 in all diseases

A

DQB1*06

Genotype frequency

Study or Subgroup	Patients Events	Patients Total	Control Events	Control Total	Weight	Odds Ratio M-H, Random, 95% CI
Fogo selvagem (Moraes, 1991)	3	38	15	41	3.9%	0.15 [0.04, 0.57]
MuSK MG (Kanai, 2016)	6	14	57	100	4.8%	0.57 [0.18, 1.75]
MuSK MG (Nikolic, 2014)	4	31	43	159	4.9%	0.40 [0.13, 1.21]
MuSK MG (Niks, 2006)	5	23	453	2440	5.5%	1.22 [0.45, 3.30]
Pemphigus (Koc, 2012) ‡	6	60	17	60	5.4%	0.28 [0.10, 0.77]
Pemphigus (Lee, 1998)**	20	30	41	100	6.3%	2.88 [1.22, 6.78]
Pemphigus (Zhang, 2019)**	69	327	179	501	9.9%	0.48 [0.35, 0.66]
PV (Dere, 2020)	5	30	14	30	4.5%	0.23 [0.07, 0.76]
PV (Gil, 2017)	14	102	131	594	8.1%	0.56 [0.31, 1.02]
PV (Lombardi, 1996)	5	33	22	102	5.1%	0.65 [0.22, 1.88]
PV (Orouji, 2014)	7	54	18	85	5.7%	0.55 [0.21, 1.43]
PV (Priyadarshini, 2018)	8	50	19	50	5.8%	0.31 [0.12, 0.80]
PV (Tunca, 2010)	4	25	40	113	4.8%	0.35 [0.11, 1.08]
TTP (Al Haddad, 2019)	2	30	3	30	2.4%	0.64 [0.10, 4.15]
TTP (Coppo, 2010)	22	60	74	172	8.0%	0.77 [0.42, 1.40]
TTP (John, 2011)	15	52	28	174	7.2%	2.11 [1.03, 4.36]
TTP (Scully, 2010)	19	50	92	200	7.8%	0.72 [0.38, 1.36]
**Total (95% CI)**		**1009**		**4951**	**100.0%**	**0.61 [0.44, 0.84]**
Total events	214		1246			

Heterogeneity: Tau² = 0.26; Chi² = 41.37, df = 16 (P = 0.0005); I² = 61%

Test for overall effect: Z = 2.98 (P = 0.003)

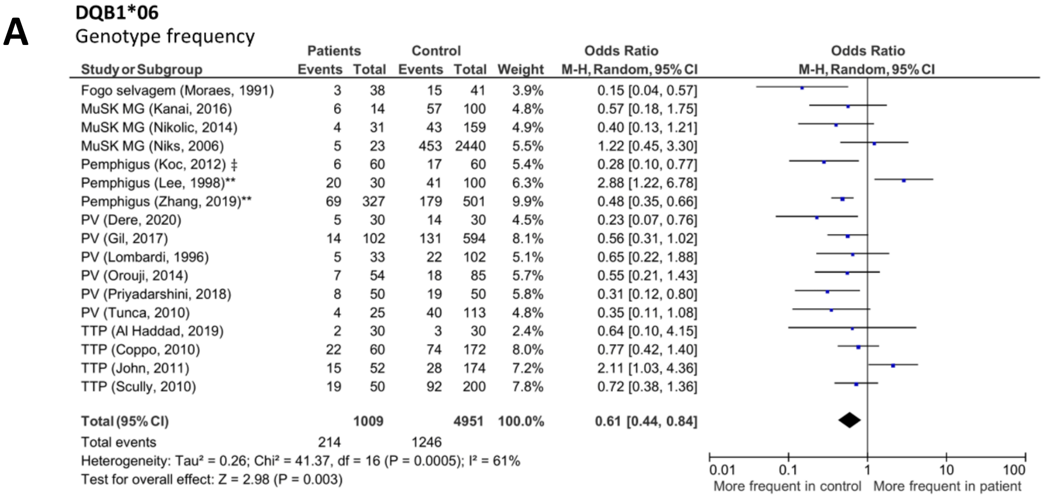

B

Allele frequency

Study or Subgroup	Patients Events	Patients Total	Control Events	Control Total	Weight	Odds Ratio M-H, Random, 95% CI
CIDP (Piccinelli, 2019) ‡	8	48	63	432	6.6%	1.17 [0.52, 2.62]
CIDP, NF155 (Ogata, 2020)	28	44	168	836	7.2%	6.96 [3.68, 13.16]
MuSK MG (Ehsan, 2015)	6	48	70	400	6.2%	0.67 [0.28, 1.65]
MuSK MG (Nikolic, 2014)	4	62	46	318	5.6%	0.41 [0.14, 1.18]
Pemphigus (Brochado, 2016) **	26	336	489	3184	8.0%	0.46 [0.31, 0.70]
Pemphigus (Zhang, 2019)**	75	654	231	1002	8.3%	0.43 [0.33, 0.57]
PF (Abida, 2009)	18	180	106	540	7.6%	0.45 [0.27, 0.77]
PV (Orouji, 2014)	8	108	18	170	6.3%	0.68 [0.28, 1.61]
PV (Párnická, 2013)	3	86	54	226	5.1%	0.12 [0.03, 0.38]
PV (Shams, 2008)	11	104	73	360	7.1%	0.47 [0.24, 0.91]
PV (Zivanovic, 2016)	14	144	46	318	7.2%	0.64 [0.34, 1.20]
PV, Europe (Delgado, 1997)	2	38	89	496	4.3%	0.25 [0.06, 1.07]
PV, Indo-Asians (Saha, 2010)*	5	114	21	118	5.8%	0.21 [0.08, 0.58]
PV, White Europeans (Saha, 2010)*	10	192	24	200	6.7%	0.40 [0.19, 0.87]
TTP (Sakai, 2020)	37	104	330	1046	7.9%	1.20 [0.79, 1.83]
**Total (95% CI)**		**2262**		**9646**	**100.0%**	**0.59 [0.38, 0.90]**
Total events	255		1828			

Heterogeneity: Tau² = 0.55; Chi² = 93.06, df = 14 (P < 0.00001); I² = 85%

Test for overall effect: Z = 2.46 (P = 0.01)

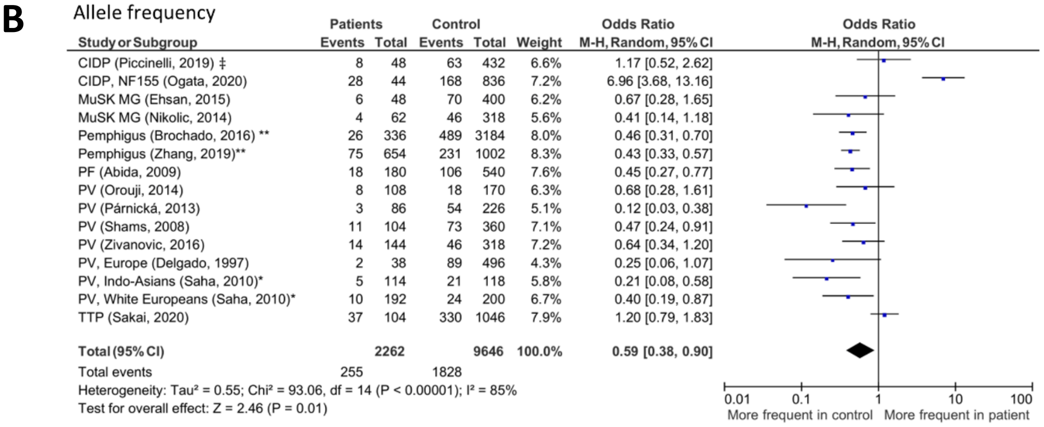

Figure S 12: Genotype (A) and allele (B) frequency of HLA-DQB1*06 in all diseases

A **DRB1*14:01**

Genotype frequency

Study or Subgroup	Patient Events	Patient Total	Control Events	Control Total	Weight	Odds Ratio M-H, Random, 95% CI
Fogo selvagem (Pavoni, 2003)	11	128	51	402	13.5%	0.65 [0.33, 1.28]
Pemphigus (Miyagawa, 1997)**	6	16	36	525	12.5%	8.15 [2.80, 23.69]
PF (Martel, 2002)	4	31	7	84	11.7%	1.63 [0.44, 6.01]
PV (Glorio, 1999)	11	30	7	199	12.5%	15.88 [5.51, 45.76]
PV (Glorio, 2002)	15	47	7	199	12.8%	12.86 [4.86, 33.99]
PV (Gonzalez-Escribano, 1998)	7	26	8	200	12.3%	8.84 [2.89, 27.06]
PV (Lombardi, 1996)	18	33	6	102	12.5%	19.20 [6.57, 56.10]
PV (Yamashina, 1998)	5	17	19	525	12.2%	11.10 [3.55, 34.67]
**Total (95% CI)**		**328**		**2236**	**100.0%**	**6.44 [2.28, 18.17]**
Total events	77		141			

Heterogeneity: Tau² = 1.95; Chi² = 57.81, df = 7 (P < 0.00001); I² = 88%
Test for overall effect: Z = 3.52 (P = 0.0004)

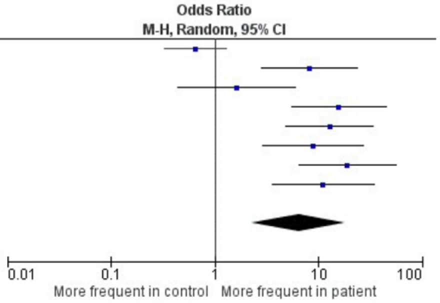

Allele frequency

Study or Subgroup	Patient Events	Patient Total	Control Events	Control Total	Weight	Odds Ratio M-H, Random, 95% CI
Pemphigus (Brochado, 2016) **	25	336	70	3184	20.8%	3.58 [2.23, 5.73]
PF, Indo-Asian (Saha, 2019)	0	20	0	118		Not estimable
PF, White British (Saha, 2019)	0	50	0	200		Not estimable
PV, Europe (Delgado, 1997)	14	38	21	496	16.1%	13.19 [5.98, 29.10]
PV, Indo-Asians (Saha, 2010)*	1	114	0	118	2.5%	3.13 [0.13, 77.69]
PV, Italians (Carcassi, 1996)	12	32	32	568	15.9%	10.05 [4.52, 22.36]
PV, Sardinians (Carcassi, 1996)	5	32	4	182	9.3%	8.24 [2.08, 32.62]
PV, White Europeans (Saha, 2010)*	2	192	0	200	2.7%	5.26 [0.25, 110.33]
PV (Párnická, 2013)	0	86	1	226	2.5%	0.87 [0.04, 21.54]
PV (Shams, 2008)	26	104	18	360	18.2%	6.33 [3.31, 12.12]
PV (Zivanovic, 2016)	6	144	7	3984	12.0%	24.70 [8.19, 74.47]
**Total (95% CI)**		**1148**		**9636**	**100.0%**	**7.65 [4.49, 13.02]**
Total events	91		153			

Heterogeneity: Tau² = 0.30; Chi² = 18.18, df = 8 (P = 0.02); I² = 56%
Test for overall effect: Z = 7.50 (P < 0.00001)

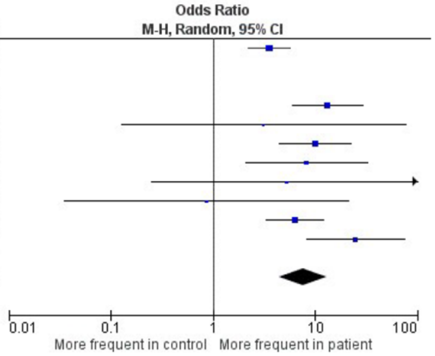

B **DRB1*14:04**

Allele frequency

Study or Subgroup	Patient Events	Patient Total	Control Events	Control Total	Weight	Odds Ratio M-H, Random, 95% CI
Pemphigus (Brochado, 2016) **	9	336	7	3184	22.8%	12.49 [4.62, 33.76]
PF, Indo-Asian (Saha, 2019)	8	20	8	118	20.5%	9.17 [2.91, 28.86]
PF, White British (Saha, 2019)	0	50	0	200		Not estimable
PV, Egypt (Haase, 2015)*	3	94	0	146	6.0%	11.21 [0.57, 219.48]
PV, German (Haase, 2015)*	1	92	0	148	5.3%	4.87 [0.20, 120.79]
PV, Indo-Asians (Saha, 2010)*	48	114	8	118	25.9%	10.00 [4.46, 22.44]
PV, White Europeans (Saha, 2010)*	9	192	0	200	6.5%	20.76 [1.20, 359.21]
PV (Párnická, 2013)	5	86	0	226	6.3%	30.57 [1.67, 559.00]
PV (Zivanovic, 2016)	19	144	0	3984	6.6%	1238.21 [74.34, 20622.95]
**Total (95% CI)**		**1128**		**8324**	**100.0%**	**15.50 [6.94, 34.63]**
Total events	102		23			

Heterogeneity: Tau² = 0.48; Chi² = 12.23, df = 7 (P = 0.09); I² = 43%
Test for overall effect: Z = 6.68 (P < 0.00001)

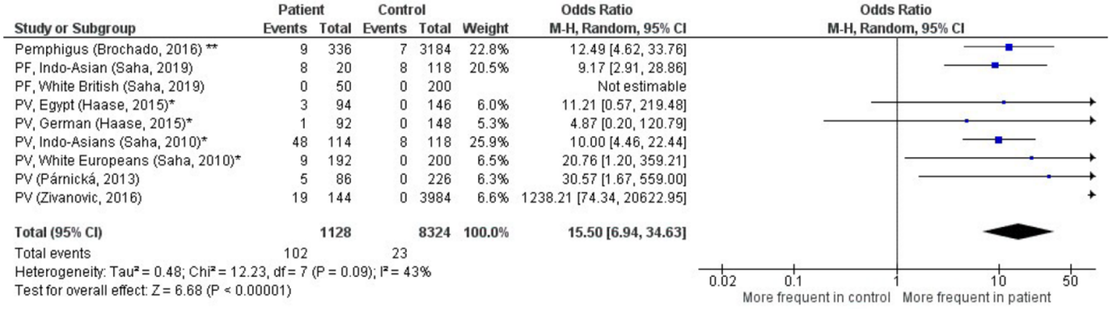

C **DRB1*14:54**

Allele frequency

Study or Subgroup	Patient Events	Patient Total	Control Events	Control Total	Weight	Odds Ratio M-H, Random, 95% CI
CIDP, NF155 (Ogata, 2020)	0	44	26	836	9.1%	0.34 [0.02, 5.73]
PF, Indo-Asian (Saha, 2019)	4	50	8	200	13.3%	2.09 [0.60, 7.23]
PF, White British (Saha, 2019)	0	20	5	118	8.9%	0.50 [0.03, 9.45]
PV, Indo-Asians (Saha, 2010)*	3	114	5	118	12.8%	0.61 [0.14, 2.62]
PV, White Europeans (Saha, 2010)*	32	192	8	200	14.3%	4.80 [2.15, 10.71]
PV (Párnická, 2013)	12	86	5	226	13.7%	7.17 [2.44, 21.02]
PV (Zivanovic, 2016)	19	144	12	3984	14.4%	50.31 [23.90, 105.91]
TTP (Sakai, 2020)	3	104	44	1046	13.5%	0.68 [0.21, 2.22]
**Total (95% CI)**		**754**		**6728**	**100.0%**	**2.41 [0.62, 9.42]**
Total events	73		113			

Heterogeneity: Tau² = 3.22; Chi² = 69.85, df = 7 (P < 0.00001); I² = 90%
Test for overall effect: Z = 1.27 (P = 0.20)

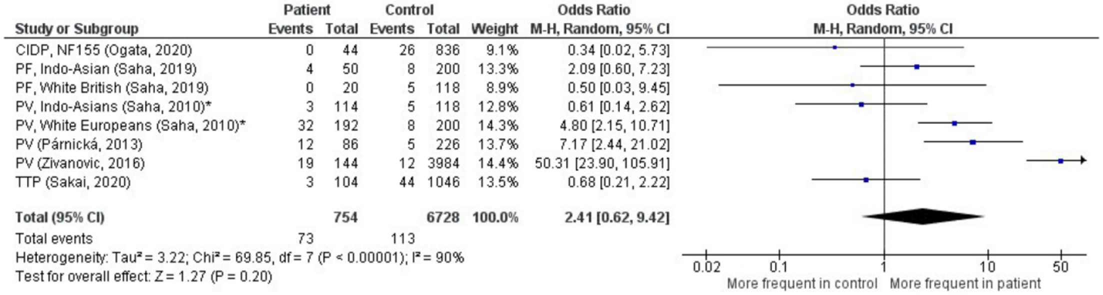

Figure S 13: Forest plots for allele and genotype frequency of HLA-DRB1*14:01 (A). Allele frequency for HLA-DRB1*14:04 (B) and HLA-DRB1*14:54 (C) for all studies.

DRB1*04:02

A Genotype frequency

Study or Subgroup	Patients Events	Patients Total	Control Events	Control Total	Weight	Odds Ratio M-H, Random, 95% CI
Fogo selvagem (Pavoni, 2003)	3	128	8	402	14.0%	1.18 [0.31, 4.52]
PF (Martel, 2002)	3	31	1	84	9.0%	8.89 [0.89, 89.00]
PV (Glorio, 1999)	13	30	6	199	15.5%	24.60 [8.29, 72.94]
PV (Glorio, 2002)	17	47	6	199	16.0%	18.23 [6.66, 49.90]
PV (Gonzalez-Escribano, 1998)	21	26	8	200	14.8%	100.80 [30.22, 336.26]
PV (Harfouch, 2014)	51	61	19	71	16.9%	13.96 [5.92, 32.91]
PV (Lombardi, 1996)	11	33	3	102	13.9%	16.50 [4.25, 64.13]
**Total (95% CI)**		**356**		**1257**	**100.0%**	**14.84 [5.83, 37.75]**
Total events	119		51			

Heterogeneity: Tau² = 1.15; Chi² = 24.93, df = 6 (P = 0.0004); I² = 76%
Test for overall effect: Z = 5.66 (P < 0.00001)

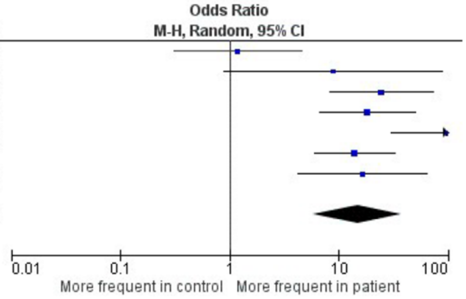

B Allele frequency

Study or Subgroup	Patients Events	Patients Total	Control Events	Control Total	Weight	Odds Ratio M-H, Random, 95% CI
Pemphigus (Brochado, 2016) **	46	336	65	3184	13.5%	7.61 [5.12, 11.31]
PF (Abida, 2009)	24	180	20	540	13.0%	4.00 [2.15, 7.43]
PV, Indo-Asians (Saha, 2010)*	16	114	1	118	8.3%	19.10 [2.49, 146.62]
PV, Italians (Carcassi, 1996)	7	32	8	568	11.6%	19.60 [6.59, 58.33]
PV, Sardinians (Carcassi, 1996)	13	32	3	182	10.7%	40.82 [10.67, 156.15]
PV, White Europeans (Saha, 2010)*	52	192	0	200	6.2%	149.84 [9.17, 2447.64]
PV (Párnická, 2013)	25	86	4	226	11.6%	22.75 [7.63, 67.85]
PV (Shams, 2008)	22	104	13	360	12.7%	7.16 [3.46, 14.81]
PV (Zivanovic, 2016)	43	144	6	3984	12.3%	282.27 [117.47, 678.27]
**Total (95% CI)**		**1220**		**9362**	**100.0%**	**21.36 [8.37, 54.47]**
Total events	248		120			

Heterogeneity: Tau² = 1.65; Chi² = 78.45, df = 8 (P < 0.00001); I² = 90%
Test for overall effect: Z = 6.41 (P < 0.00001)

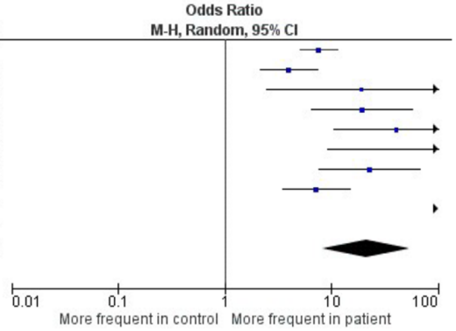

Figure S 14: Forest plots for allele (A) and genotype (B) frequency of HLA-DRB1*04:02 for all studies.

DQB1*05:01

A Allele frequency

Study or Subgroup	Patient Events	Patient Total	Control Events	Control Total	Weight	Odds Ratio M-H, Random, 95% CI
CIDP, NF155 (Ogata, 2020)	2	44	63	836	6.0%	0.58 [0.14, 2.47]
Pemphigus (Brochado, 2016) **	69	336	328	3184	13.2%	2.25 [1.69, 3.00]
PF, Indo-Asian (Saha, 2019)	1	20	10	118	3.6%	0.57 [0.07, 4.70]
PF, White British (Saha, 2019)	10	50	28	200	9.9%	1.54 [0.69, 3.42]
PV, Europe (Delgado, 1997)	5	38	59	496	8.6%	1.12 [0.42, 2.99]
PV, Indo-Asians (Saha, 2010)*	4	115	10	118	7.3%	0.39 [0.12, 1.28]
PV, White Europeans (Saha, 2010)*	12	192	28	200	10.6%	0.41 [0.20, 0.83]
PV (Párnická, 2013)	9	86	25	225	9.8%	0.94 [0.42, 2.09]
PV (Shams, 2008)	11	104	66	360	10.8%	0.53 [0.27, 1.04]
PV (Zivanovic, 2016)	11	114	30	318	10.4%	1.03 [0.50, 2.12]
TTP (Sakai, 2020)	7	104	46	1046	9.7%	1.57 [0.69, 3.57]
**Total (95% CI)**		**1203**		**7101**	**100.0%**	**0.92 [0.58, 1.46]**
Total events	141		693			

Heterogeneity: Tau² = 0.41; Chi² = 38.95, df = 10 (P < 0.0001); I² = 74%
Test for overall effect: Z = 0.36 (P = 0.72)

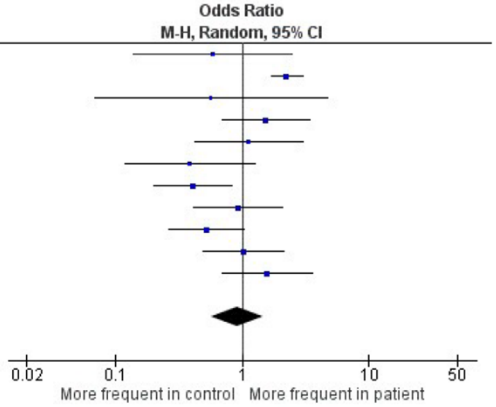

Figure S 15: Forest plots for allele frequency of (A) HLA-DQB1*05:01 and allele and genotype frequency of (B) HLA-DQB1*05:02 and (C) HLA-DQB1*05:03 for all studies.
(continued on next page)

B **DQB1*05:02**

Genotype frequency

Study or Subgroup	Patient Events	Patient Total	Control Events	Control Total	Weight	Odds Ratio M-H, Random, 95% CI
Pemphigus (Lee, 1998)**	2	30	6	100	19.5%	1.12 [0.21, 5.86]
PV (Gil, 2017)	1	102	18	594	16.5%	0.32 [0.04, 2.40]
PV (Lombardi, 1996)	3	33	10	102	22.2%	0.92 [0.24, 3.56]
PV (Yamashina, 1998)	3	17	11	525	22.0%	10.01 [2.51, 39.90]
TTP (John, 2011)	3	54	3	174	19.7%	3.35 [0.66, 17.12]
**Total (95% CI)**		**236**		**1495**	**100.0%**	**1.75 [0.52, 5.86]**
Total events	12		48			

Heterogeneity: $Tau^2 = 1.24$; $Chi^2 = 11.68$, df = 4 (P = 0.02); $I^2 = 66\%$
Test for overall effect: Z = 0.90 (P = 0.37)

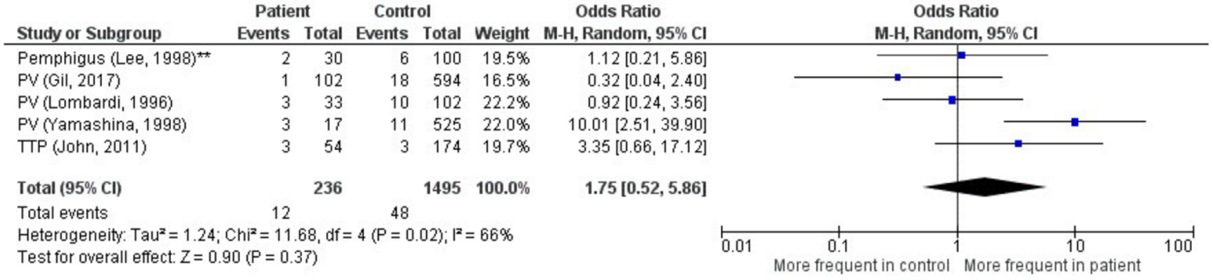

Allele frequency

Study or Subgroup	Patient Events	Patient Total	Control Events	Control Total	Weight	Odds Ratio M-H, Random, 95% CI
CIDP, NF155 (Ogata, 2020)	0	44	17	836	10.8%	0.53 [0.03, 8.89]
PF, White British (Saha, 2019)	5	50	2	200	16.1%	11.00 [2.07, 58.52]
PV (Párnická, 2013)	1	86	17	225	14.3%	0.14 [0.02, 1.10]
PV (Shams, 2008)	19	104	9	360	20.0%	8.72 [3.81, 19.95]
PV (Zivanovic, 2016)	10	144	27	318	20.3%	0.80 [0.38, 1.71]
TTP (Sakai, 2020)	3	104	34	1046	18.4%	0.88 [0.27, 2.93]
**Total (95% CI)**		**532**		**2985**	**100.0%**	**1.50 [0.40, 5.57]**
Total events	38		106			

Heterogeneity: $Tau^2 = 2.06$; $Chi^2 = 31.58$, df = 5 (P < 0.00001); $I^2 = 84\%$
Test for overall effect: Z = 0.61 (P = 0.54)

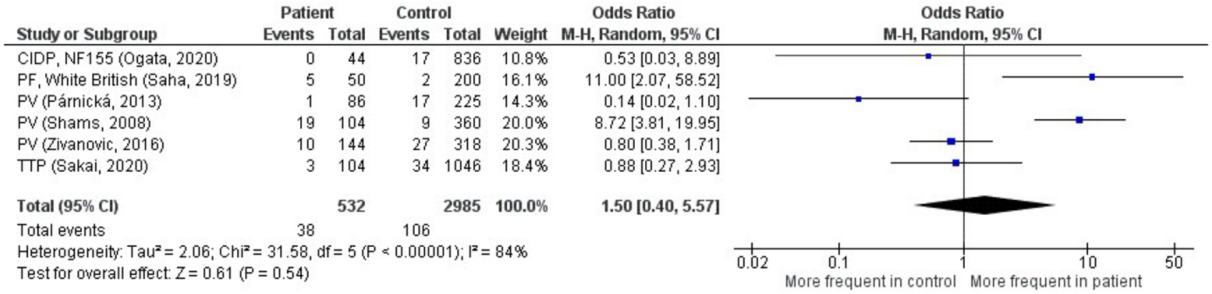

C **DQB1*05:03**

Genotype frequency

Study or Subgroup	Patient Events	Patient Total	Control Events	Control Total	Weight	Odds Ratio M-H, Random, 95% CI
Pemphigus (Lee, 1998)**	8	30	10	100	14.3%	3.27 [1.16, 9.26]
PF (Martel, 2002)	8	30	8	64	13.6%	2.55 [0.85, 7.62]
PV (Gil, 2017)	13	102	21	594	19.3%	3.99 [1.93, 8.24]
PV (Glorio, 1999)	11	30	10	199	15.2%	10.94 [4.12, 29.09]
PV (Glorio, 2002)	14	47	10	199	16.5%	8.02 [3.29, 19.56]
PV (Lombardi, 1996)	18	33	6	102	13.9%	19.20 [6.57, 56.10]
TTP (John, 2011)	2	54	3	174	7.1%	2.19 [0.36, 13.48]
**Total (95% CI)**		**326**		**1432**	**100.0%**	**5.69 [3.26, 9.93]**
Total events	74		68			

Heterogeneity: $Tau^2 = 0.28$; $Chi^2 = 12.37$, df = 6 (P = 0.05); $I^2 = 51\%$
Test for overall effect: Z = 6.13 (P < 0.00001)

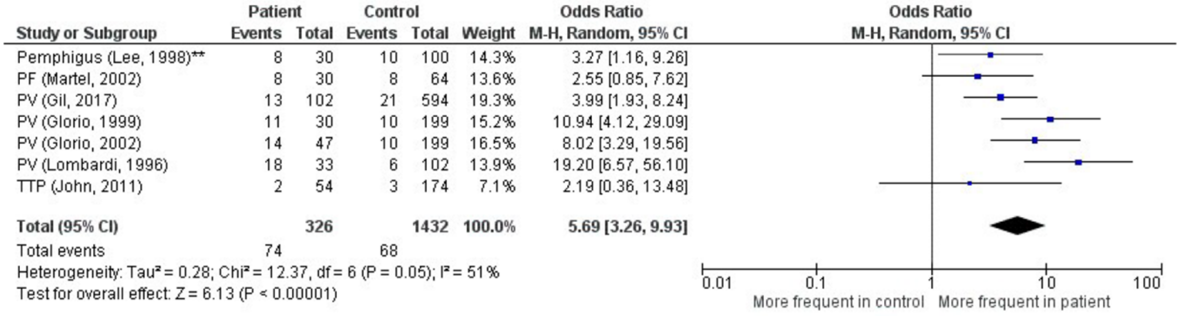

Allele frequency

Study or Subgroup	Patient Events	Patient Total	Control Events	Control Total	Weight	Odds Ratio M-H, Random, 95% CI
CIDP, NF155 (Ogata, 2020)	0	44	30	836	3.4%	0.30 [0.02, 4.94]
Pemphigus (Brochado, 2016) **	12	336	69	3184	10.1%	1.67 [0.90, 3.12]
PF, Indo-Asian (Saha, 2019)	9	20	15	118	8.6%	5.62 [2.00, 15.80]
PF, White British (Saha, 2019)	4	50	8	200	7.8%	2.09 [0.60, 7.23]
PV, Europe (Delgado, 1997)	14	38	20	496	9.5%	13.88 [6.26, 30.79]
PV, Indo-Asians (Saha, 2010)*	54	114	15	118	10.0%	6.18 [3.21, 11.89]
PV, Italians (Carcassi, 1996)	12	32	15	812	9.2%	31.88 [13.23, 76.80]
PV, Sardinians (Carcassi, 1996)	5	32	4	182	7.3%	8.24 [2.08, 32.62]
PV, White Europeans (Saha, 2010)*	43	192	8	200	9.6%	6.93 [3.16, 15.18]
PV (Párnická, 2013)	19	86	6	225	8.9%	10.35 [3.97, 26.97]
PV (Zivanovic, 2016)	38	144	19	318	10.2%	5.64 [3.12, 10.21]
TTP (Sakai, 2020)	1	104	56	1046	5.2%	0.17 [0.02, 1.25]
**Total (95% CI)**		**1192**		**7735**	**100.0%**	**4.96 [2.66, 9.24]**
Total events	211		265			

Heterogeneity: $Tau^2 = 0.89$; $Chi^2 = 58.69$, df = 11 (P < 0.00001); $I^2 = 81\%$
Test for overall effect: Z = 5.05 (P < 0.00001)

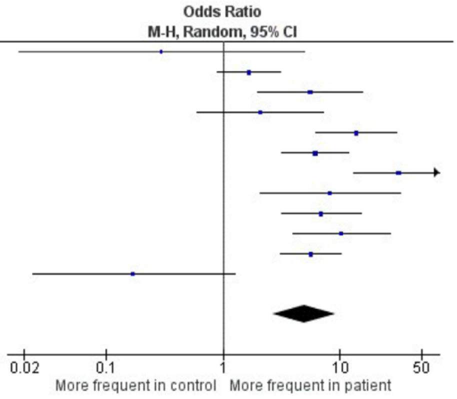

Figure S 15 continued

DRB1*14

Genotype frequency

A **Brazil**

Study or Subgroup	Patient Events	Patient Total	Control Events	Control Total	Weight	Odds Ratio M-H, Random, 95% CI
Fogo selvagem (Cerna, 1993)	8	10	38	74	25.7%	3.79 [0.75, 19.06]
Fogo selvagem (Pavoni, 2003)	22	128	100	402	37.7%	0.63 [0.38, 1.05]
PV (Gil, 2017)	16	102	30	594	36.6%	3.50 [1.83, 6.69]
**Total (95% CI)**		**240**		**1070**	**100.0%**	**1.87 [0.47, 7.43]**
Total events	46		168			

Heterogeneity: Tau² = 1.25; Chi² = 18.70, df = 2 (P < 0.0001); I² = 89%

Test for overall effect: Z = 0.89 (P = 0.38)

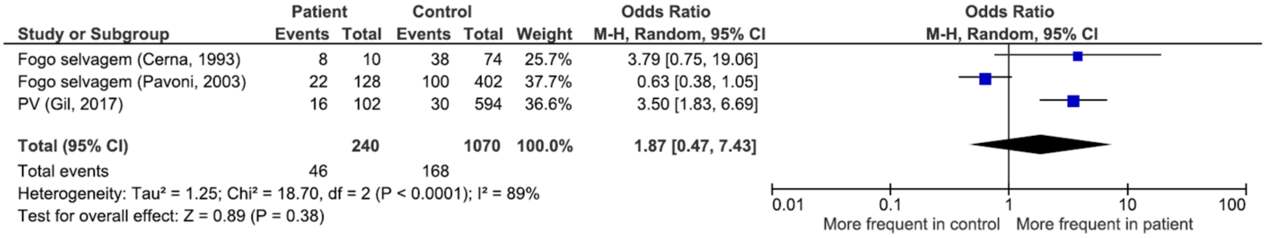

B **Japan**

Study or Subgroup	Patient Events	Patient Total	Control Events	Control Total	Weight	Odds Ratio M-H, Random, 95% CI
MuSK MG (Kanai, 2016)	8	14	18	100	28.1%	6.07 [1.88, 19.67]
Pemphigus (Miyagawa, 1997)**	6	16	36	525	34.0%	8.15 [2.80, 23.69]
PV (Yamashina, 1998)	9	17	36	525	37.9%	15.28 [5.56, 41.99]
**Total (95% CI)**		**47**		**1150**	**100.0%**	**9.52 [5.11, 17.75]**
Total events	23		90			

Heterogeneity: Tau² = 0.00; Chi² = 1.52, df = 2 (P = 0.47); I² = 0%

Test for overall effect: Z = 7.10 (P < 0.00001)

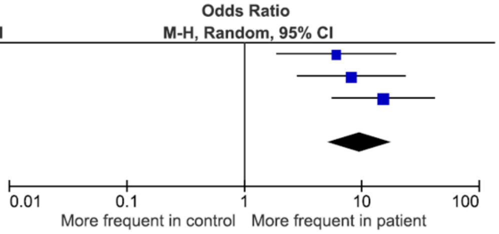

C **Turkey**

Study or Subgroup	Patient Events	Patient Total	Control Events	Control Total	Weight	Odds Ratio M-H, Random, 95% CI
MuSK MG (Alahgholi-Hajibehzad, 2013)	14	48	29	250	30.5%	3.14 [1.51, 6.53]
Pemphigus (Birol, 2002) ‡	10	33	15	100	19.2%	2.46 [0.98, 6.20]
Pemphigus (Koc, 2012) ‡	28	60	9	60	21.6%	4.96 [2.07, 11.85]
PV (Dere, 2020)	15	30	8	30	14.1%	2.75 [0.93, 8.10]
PV (Tunca, 2010)	8	25	10	113	14.6%	4.85 [1.68, 14.02]
**Total (95% CI)**		**196**		**553**	**100.0%**	**3.46 [2.31, 5.18]**
Total events	75		71			

Heterogeneity: Tau² = 0.00; Chi² = 1.80, df = 4 (P = 0.77); I² = 0%

Test for overall effect: Z = 6.00 (P < 0.00001)

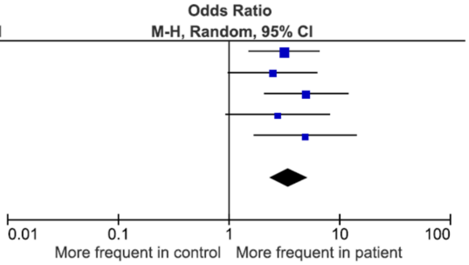

Figure S 16: **Within ancestry analysis**: Forest plots for genotype frequency of HLA-DRB1*14 for all studies from Brazil (A), Japan (B) and Turkey (C).

DQB1*05

Genotype frequency

A **Japan**

Study or Subgroup	Patient Events	Patient Total	Control Events	Control Total	Weight	Odds Ratio M-H, Random, 95% CI
MuSK MG (Kanai, 2016)	11	14	30	100	23.9%	8.56 [2.23, 32.88]
Pemphigus (Miyagawa, 1997)**	5	16	25	525	33.8%	9.09 [2.93, 28.17]
PV (Yamashina, 1998)	9	17	36	525	42.3%	15.28 [5.56, 41.99]
**Total (95% CI)**		**47**		**1150**	**100.0%**	**11.16 [5.78, 21.55]**
Total events	25		91			

Heterogeneity: Tau² = 0.00; Chi² = 0.67, df = 2 (P = 0.72); I² = 0%

Test for overall effect: Z = 7.19 (P < 0.00001)

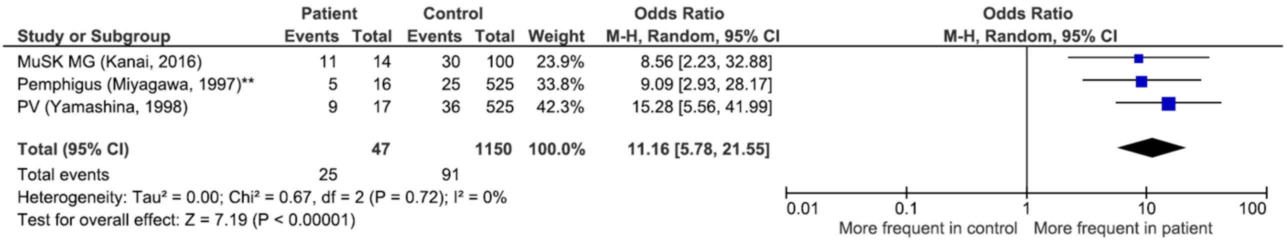

B **Turkey**

Study or Subgroup	Patient Events	Patient Total	Control Events	Control Total	Weight	Odds Ratio M-H, Random, 95% CI
MuSK MG (Alahgholi-Hajibehzad, 2013)	35	48	90	250	29.8%	4.79 [2.41, 9.51]
Pemphigus (Koc, 2012) ‡	38	60	21	60	28.4%	3.21 [1.52, 6.77]
PV (Dere, 2020)	17	30	3	30	16.5%	11.77 [2.92, 47.46]
PV (Tunca, 2010)	10	25	38	113	25.3%	1.32 [0.54, 3.20]
**Total (95% CI)**		**163**		**453**	**100.0%**	**3.57 [1.71, 7.46]**
Total events	100		152			

Heterogeneity: Tau² = 0.35; Chi² = 8.40, df = 3 (P = 0.04); I² = 64%

Test for overall effect: Z = 3.39 (P = 0.0007)

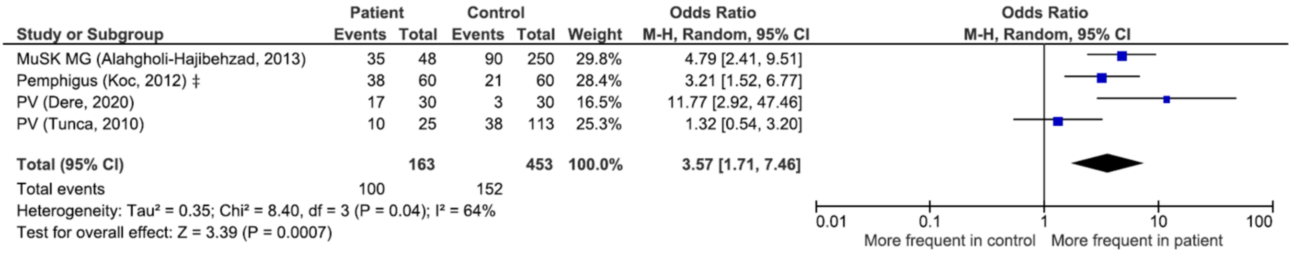

Figure S 17: **Within ancestry analysis**: Forest plots for genotype frequency of HLA-DQB1*05 for all studies from Japan (A) and Turkey (B).

DRB1*04

Genotype frequency

A **Brazil**

Study or Subgroup	Patients Events	Patients Total	Control Events	Control Total	Weight	Odds Ratio M-H, Random, 95% CI
Fogo selvagem (Cerna, 1993)	6	10	10	74	8.0%	9.60 [2.30, 40.11]
Fogo selvagem (Pavoni, 2003)	49	128	76	402	49.6%	2.66 [1.72, 4.11]
PV (Gil, 2017)	29	102	66	594	42.4%	3.18 [1.93, 5.24]
**Total (95% CI)**		**240**		**1070**	**100.0%**	**3.18 [2.09, 4.84]**
Total events	84		152			

Heterogeneity: Tau² = 0.04; Chi² = 2.88, df = 2 (P = 0.24); I² = 30%

Test for overall effect: Z = 5.39 (P < 0.00001)

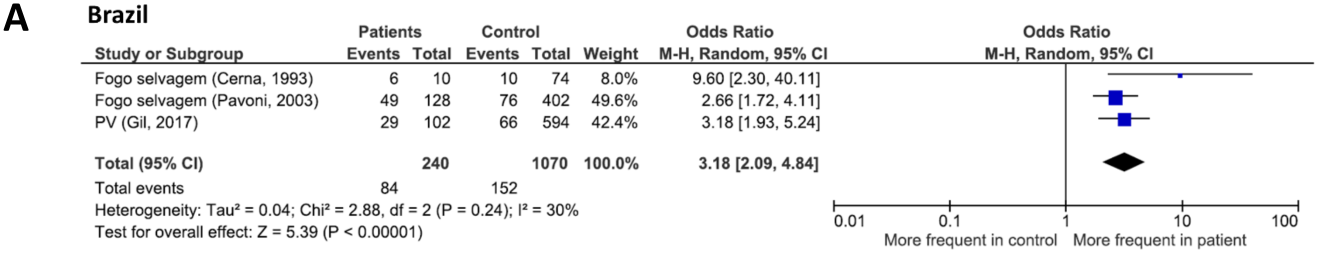

B **Japan**

Study or Subgroup	Patients Events	Patients Total	Control Events	Control Total	Weight	Odds Ratio M-H, Random, 95% CI
MuSK MG (Kanai, 2016)	4	14	44	100	32.5%	0.51 [0.15, 1.73]
Pemphigus (Miyagawa, 1997)**	7	16	35	525	33.7%	10.89 [3.83, 30.98]
PV (Yamashina, 1998)	7	17	35	525	33.8%	9.80 [3.52, 27.31]
**Total (95% CI)**		**47**		**1150**	**100.0%**	**3.88 [0.54, 27.80]**
Total events	18		114			

Heterogeneity: Tau² = 2.71; Chi² = 19.40, df = 2 (P < 0.0001); I² = 90%

Test for overall effect: Z = 1.35 (P = 0.18)

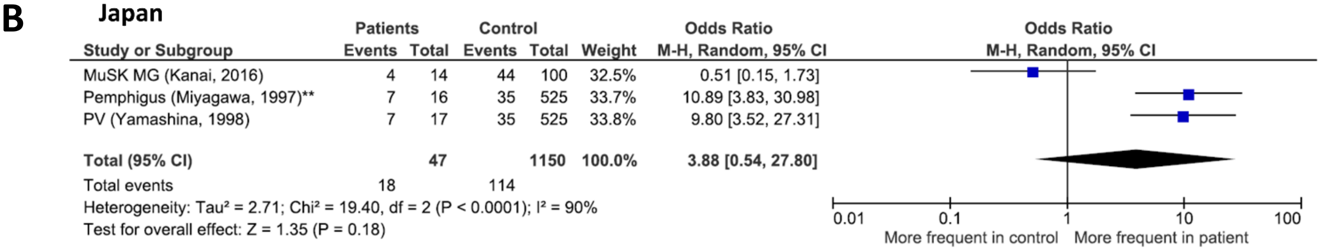

C **Turkey**

Study or Subgroup	Patients Events	Patients Total	Control Events	Control Total	Weight	Odds Ratio M-H, Random, 95% CI
Pemphigus (Birol, 2002) ‡	25	33	29	100	37.1%	7.65 [3.09, 18.93]
Pemphigus (Koc, 2012) ‡	39	60	11	60	43.0%	8.27 [3.56, 19.20]
PV (Dere, 2020)	25	30	9	30	19.9%	11.67 [3.38, 40.22]
**Total (95% CI)**		**123**		**190**	**100.0%**	**8.60 [4.95, 14.94]**
Total events	89		49			

Heterogeneity: Tau² = 0.00; Chi² = 0.31, df = 2 (P = 0.86); I² = 0%

Test for overall effect: Z = 7.64 (P < 0.00001)

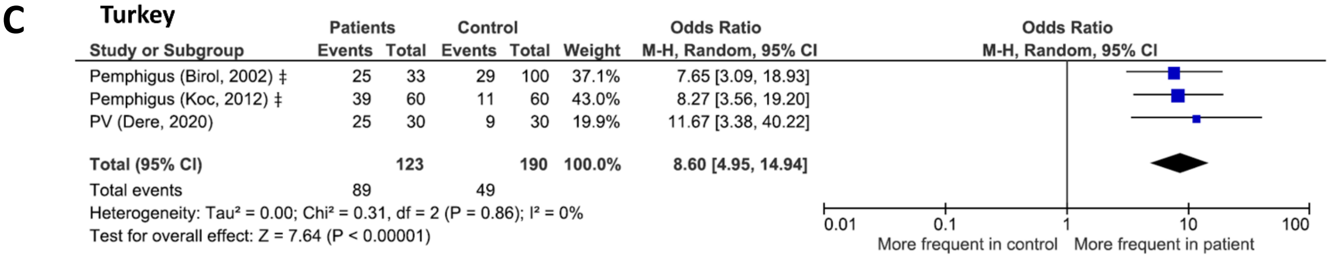

DRB1*04

Allele frequency

D **Brazil**

Study or Subgroup	Patients Events	Patients Total	Control Events	Control Total	Weight	Odds Ratio M-H, Random, 95% CI
Fogo selvagem (de Sena Nogueira Maehara, 2017)	33	84	87	956	35.0%	6.46 [3.96, 10.55]
Pemphigus (Brochado, 2016) **	46	336	65	3184	43.6%	7.61 [5.12, 11.31]
PF (de Sena Nogueira Maehara, 2017)	13	34	133	894	21.3%	3.54 [1.73, 7.25]
**Total (95% CI)**		**454**		**5034**	**100.0%**	**6.10 [4.15, 8.98]**
Total events	92		285			

Heterogeneity: Tau² = 0.05; Chi² = 3.38, df = 2 (P = 0.18); I² = 41%

Test for overall effect: Z = 9.20 (P < 0.00001)

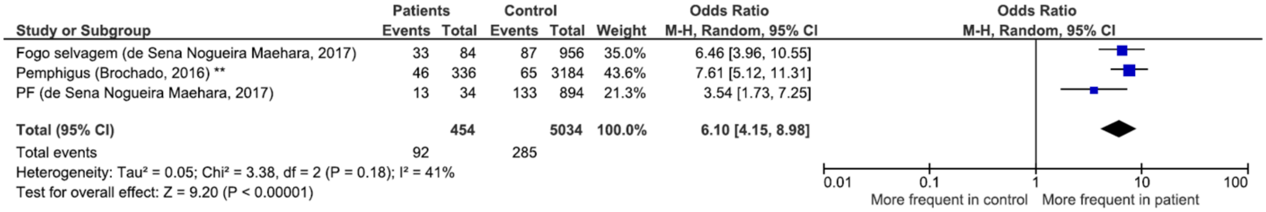

Figure S 18: **Within ancestry analysis**: Forest plots for genotype frequency of HLA-DRB1*04 for all studies from Brazil (A), Japan (B) and Turkey (C) and allele frequency for all studies from Brazil (D).

DRB1*01

Genotype frequency

Study or Subgroup	Patients Events	Patients Total	Control Events	Control Total	Weight	Odds Ratio M-H, Random, 95% CI
Fogo selvagem (Moraes, 1991)	22	37	10	49	5.2%	5.72 [2.20, 14.87]
Fogo selvagem (Pavoni, 2003)	78	128	70	402	5.6%	7.40 [4.77, 11.47]
MuSK MG (Kanai, 2016)	1	14	15	100	3.9%	0.44 [0.05, 3.58]
MuSK MG (Nikolic, 2014)	3	31	386	1992	5.0%	0.45 [0.13, 1.47]
MuSK MG (Niks, 2006)	9	23	14	2440	5.2%	111.40 [41.45, 299.36]
Pemphigus (Lee, 1998)**	19	30	12	100	5.2%	12.67 [4.87, 32.97]
Pemphigus (Zhang, 2019)**	9	327	26	501	5.4%	0.52 [0.24, 1.12]
PF (Martel, 2002)	7	31	16	84	5.2%	1.24 [0.45, 3.38]
PV (Dere, 2020)	1	30	1	30	3.2%	1.00 [0.06, 16.76]
PV (Gil, 2017)	2	102	61	594	4.7%	0.17 [0.04, 0.73]
PV (Gonzalez-Escribano, 1998)	1	26	43	200	4.0%	0.15 [0.02, 1.11]
PV (Harfouch, 2014)	5	91	33	270	5.2%	0.42 [0.16, 1.10]
PV (Khan, 2015)	0	28	0	150		Not estimable
PV (Lombardi, 1996)	2	33	13	102	4.6%	0.44 [0.09, 2.07]
PV (Priyadarshini, 2018)	0	50	3	50	3.0%	0.13 [0.01, 2.67]
PV (Thomas, 1998)	1	26	43	200	4.0%	0.15 [0.02, 1.11]
PV (Tunca, 2010)	3	25	16	113	4.8%	0.83 [0.22, 3.09]
TTP (Al Haddad, 2019)	8	30	2	30	4.5%	5.09 [0.98, 26.43]
TTP (Coppo, 2010)	11	61	32	172	5.4%	0.96 [0.45, 2.05]
TTP (John, 2011)	7	54	2395	11407	5.3%	0.56 [0.25, 1.24]
TTP (Scully, 2010)	9	50	38	200	5.3%	0.94 [0.42, 2.09]
TTP (Sinkovits, 2017)	8	75	38	204	5.3%	0.52 [0.23, 1.18]
**Total (95% CI)**		**1302**		**19390**	**100.0%**	**1.10 [0.52, 2.33]**
Total events	206		3267			

Heterogeneity: Tau² = 2.57; Chi² = 216.32, df = 20 (P < 0.00001); I² = 91%

Test for overall effect: Z = 0.25 (P = 0.81)

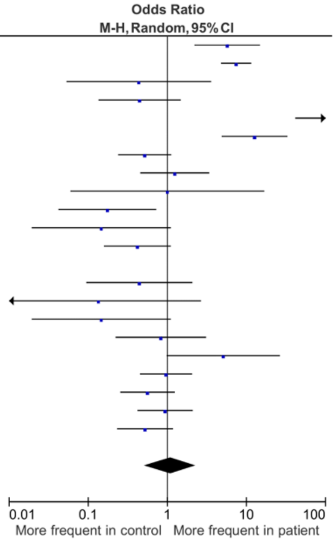

Allele frequency

Study or Subgroup	Patients Events	Patients Total	Control Events	Control Total	Weight	Odds Ratio M-H, Random, 95% CI
CIDP (Piccinelli, 2019) ‡	5	48	48	432	6.4%	0.93 [0.35, 2.46]
CIDP, NF155 (Ogata, 2020)	2	44	57	836	4.9%	0.65 [0.15, 2.76]
MuSK MG (Ehsan, 2015)	4	48	22	400	5.9%	1.56 [0.51, 4.74]
MuSK MG (Nikolic, 2014)	2	62	410	3984	5.0%	0.29 [0.07, 1.19]
Pemphigus (Brochado, 2016) **	69	336	263	3184	8.3%	2.87 [2.14, 3.85]
Pemphigus (Torzecka, 2003)**	15	106	31	304	7.4%	1.45 [0.75, 2.81]
Pemphigus (Zhang, 2019)**	9	654	31	1002	7.1%	0.44 [0.21, 0.92]
PF, Indo-Asian (Saha, 2019)	8	50	24	200	6.8%	1.40 [0.59, 3.33]
PF, White British (Saha, 2019)	0	20	4	118	2.1%	0.62 [0.03, 11.97]
PV (Párnická, 2013)	6	86	23	226	6.5%	0.66 [0.26, 1.69]
PV (Rangel-Gamboa, 2015) ‡	13	86	10	198	6.8%	3.35 [1.41, 7.97]
PV (Shams, 2008)	2	104	24	360	4.9%	0.27 [0.06, 1.18]
PV (Zivanovic, 2016)	7	144	410	3984	7.1%	0.45 [0.21, 0.96]
PV, Egypt (Haase, 2015)*	1	94	7	146	3.3%	0.21 [0.03, 1.76]
PV, Europe (Delgado, 1997)	0	38	1	26	1.8%	0.22 [0.01, 5.63]
PV, German (Haase, 2015)*	1	92	15	148	3.4%	0.10 [0.01, 0.75]
PV, Pakistan (Delgado, 1997) †	3	38	54	496	5.6%	0.70 [0.21, 2.36]
TTP (Sakai, 2020)	6	104	40	1046	6.7%	1.54 [0.64, 3.72]
**Total (95% CI)**		**2154**		**17090**	**100.0%**	**0.81 [0.50, 1.32]**
Total events	153		1474			

Heterogeneity: Tau² = 0.72; Chi² = 75.91, df = 17 (P < 0.00001); I² = 78%

Test for overall effect: Z = 0.83 (P = 0.41)

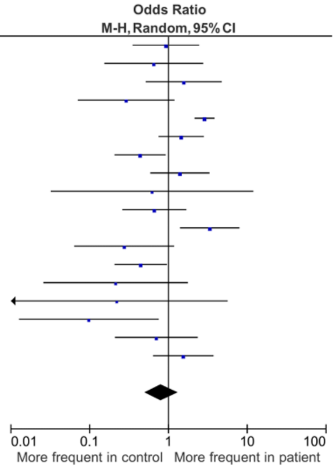

Figure S 19: Allele and genotype frequency of HLA-DRB1*01 in all diseases

DRB1*08

Genotype frequency

Study or Subgroup	Patients Events	Patients Total	Control Events	Control Total	Weight	Odds Ratio M-H, Random, 95% CI
Fogo selvagem (Cerna, 1993)	1	10	34	74	3.0%	0.13 [0.02, 1.08]
Fogo selvagem (Pavoni, 2003)	6	128	62	402	5.9%	0.27 [0.11, 0.64]
MuSK MG (Kanai, 2016)	0	14	28	100	2.0%	0.09 [0.01, 1.52]
MuSK MG (Nikolic, 2014)	3	31	123	1992	5.0%	1.63 [0.49, 5.43]
Pemphigus (Lee, 1998)**	7	30	20	100	5.6%	1.22 [0.46, 3.24]
Pemphigus (Zhang, 2019)**	28	327	86	501	7.0%	0.45 [0.29, 0.71]
PF (Martel, 2002)	0	31	2	84	1.8%	0.52 [0.02, 11.22]
PV (Dere, 2020)	0	30	2	30	1.8%	0.19 [0.01, 4.06]
PV (Gil, 2017)	18	102	40	594	6.6%	2.97 [1.63, 5.42]
PV (Glorio, 1999)	9	30	29	199	5.9%	2.51 [1.05, 6.02]
PV (Glorio, 2002)	14	47	29	199	6.3%	2.49 [1.19, 5.21]
PV (Gonzalez-Escribano, 1998)	2	26	10	200	4.0%	1.58 [0.33, 7.66]
PV (Harfouch, 2014)	7	91	9	270	5.5%	2.42 [0.87, 6.69]
PV (Khan, 2015)	2	28	7	150	3.9%	1.57 [0.31, 7.99]
PV (Lombardi, 1996)	7	33	3	102	4.4%	8.88 [2.15, 36.75]
PV (Priyadarshini, 2018)	4	50	1	50	2.8%	4.26 [0.46, 39.54]
PV (Thomas, 1998)	2	26	10	200	4.0%	1.58 [0.33, 7.66]
PV (Tunca, 2010)	1	25	4	113	2.8%	1.14 [0.12, 10.62]
TTP (Al Haddad, 2019)	5	30	1	30	2.8%	5.80 [0.63, 53.01]
TTP (Coppo, 2010)	4	61	11	172	5.0%	1.03 [0.31, 3.35]
TTP (John, 2011)	1	54	730	11407	3.2%	0.28 [0.04, 2.00]
TTP (Scully, 2010)	6	50	8	200	5.3%	3.27 [1.08, 9.91]
TTP (Sinkovits, 2017)	5	75	18	204	5.5%	0.74 [0.26, 2.06]
**Total (95% CI)**		**1329**		**17373**	**100.0%**	**1.27 [0.79, 2.02]**
Total events	132		1267			

Heterogeneity: $Tau^2 = 0.76$; $Chi^2 = 75.62$, df = 22 (P < 0.00001); $I^2 = 71\%$

Test for overall effect: Z = 0.99 (P = 0.32)

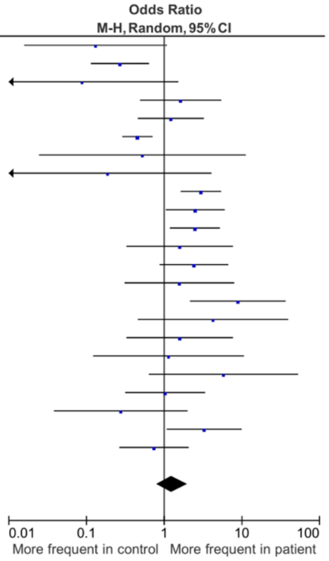

Allele frequency

Study or Subgroup	Patients Events	Patients Total	Control Events	Control Total	Weight	Odds Ratio M-H, Random, 95% CI
CIDP (Piccinelli, 2019) ‡	0	48	19	432	3.1%	0.22 [0.01, 3.68]
CIDP, NF155 (Ogata, 2020)	6	44	95	836	8.2%	1.23 [0.51, 2.99]
MuSK MG (Ehsan, 2015)	0	48	6	400	3.0%	0.63 [0.03, 11.28]
MuSK MG (Nikolic, 2014)	3	62	123	3984	7.2%	1.60 [0.49, 5.16]
Pemphigus (Brochado, 2016) **	15	336	42	3184	9.1%	3.50 [1.92, 6.37]
Pemphigus (Torzecka, 2003)**	3	106	7	304	6.5%	1.24 [0.31, 4.87]
Pemphigus (Zhang, 2019)**	28	654	97	1002	9.5%	0.42 [0.27, 0.64]
PV (Párnická, 2013)	2	86	7	226	5.8%	0.74 [0.15, 3.66]
PV (Rangel-Gamboa, 2015) ‡	10	86	33	198	8.6%	0.66 [0.31, 1.40]
PV (Shams, 2008)	2	104	8	360	5.9%	0.86 [0.18, 4.13]
PV, Egypt (Haase, 2015)*	13	94	2	146	6.1%	11.56 [2.54, 52.49]
PV, Europe (Delgado, 1997)	1	38	8	496	4.4%	1.65 [0.20, 13.54]
PV, German (Haase, 2015)*	2	92	5	148	5.6%	0.64 [0.12, 3.35]
PV, Italians (Carcassi, 1996)	3	32	2	568	5.1%	29.28 [4.71, 182.08]
PV, Sardinians (Carcassi, 1996)	0	32	1	182	2.5%	1.86 [0.07, 46.70]
TTP (Sakai, 2020)	22	104	102	1046	9.3%	2.48 [1.49, 4.15]
**Total (95% CI)**		**1966**		**13512**	**100.0%**	**1.48 [0.82, 2.68]**
Total events	110		557			

Heterogeneity: $Tau^2 = 0.92$; $Chi^2 = 69.89$, df = 15 (P < 0.00001); $I^2 = 79\%$

Test for overall effect: Z = 1.29 (P = 0.20)

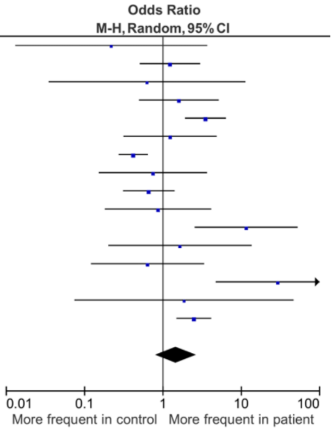

Figure S 20: Allele and genotype frequency of HLA-DRB1*08 in all diseases

DRB1*10

Genotype frequency

Study or Subgroup	Patients Events	Patients Total	Control Events	Control Total	Weight	Odds Ratio M-H, Random, 95% CI
Fogo selvagem (Pavoni, 2003)	5	128	17	402	12.6%	0.92 [0.33, 2.55]
MuSK MG (Nikolic, 2014)	0	31	44	1992	2.2%	0.70 [0.04, 11.54]
Pemphigus (Lee, 1998)**	8	30	7	100	11.0%	4.83 [1.58, 14.74]
Pemphigus (Zhang, 2019)**	9	327	8	501	13.6%	1.74 [0.67, 4.57]
PF (Martel, 2002)	7	31	16	84	12.8%	1.24 [0.45, 3.38]
PV (Gil, 2017)	3	102	13	594	9.0%	1.35 [0.38, 4.84]
PV (Gonzalez-Escribano, 1998)	0	26	4	200	2.0%	0.82 [0.04, 15.74]
PV (Harfouch, 2014)	3	91	12	270	8.9%	0.73 [0.20, 2.66]
PV (Khan, 2015)	0	28	27	150	2.2%	0.08 [0.00, 1.33]
PV (Priyadarshini, 2018)	2	50	5	50	5.6%	0.38 [0.07, 2.03]
PV (Thomas, 1998)	0	26	4	200	2.0%	0.82 [0.04, 15.74]
PV (Tunca, 2010)	0	25	5	113	2.1%	0.39 [0.02, 7.22]
TTP (Al Haddad, 2019)	2	30	3	30	4.7%	0.64 [0.10, 4.15]
TTP (Coppo, 2010)	3	61	1	172	3.3%	8.84 [0.90, 86.71]
TTP (Scully, 2010)	0	50	3	200	2.0%	0.56 [0.03, 10.99]
TTP (Sinkovits, 2017)	2	75	5	204	5.8%	1.09 [0.21, 5.74]
**Total (95% CI)**		**1111**		**5262**	**100.0%**	**1.18 [0.76, 1.81]**
Total events	44		174			

Heterogeneity: Tau² = 0.12; Chi² = 17.79, df = 15 (P = 0.27); I² = 16%

Test for overall effect: Z = 0.74 (P = 0.46)

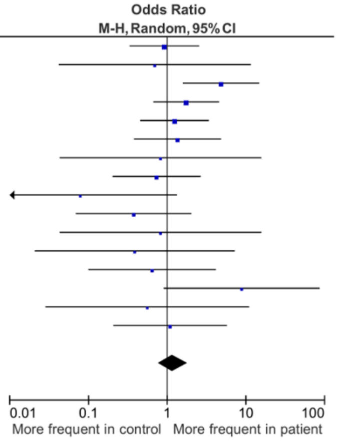

Allele frequency

Study or Subgroup	Patients Events	Patients Total	Control Events	Control Total	Weight	Odds Ratio M-H, Random, 95% CI
CIDP (Piccinelli, 2019) ‡	2	48	0	432	5.8%	46.51 [2.20, 983.35]
CIDP, NF155 (Ogata, 2020)	0	44	6	836	6.3%	1.44 [0.08, 25.89]
MuSK MG (Ehsan, 2015)	1	48	16	400	10.8%	0.51 [0.07, 3.94]
MuSK MG (Nikolic, 2014)	0	62	44	3984	6.7%	0.71 [0.04, 11.63]
Pemphigus (Torzecka, 2003)**	0	106	7	304	6.4%	0.19 [0.01, 3.29]
Pemphigus (Zhang, 2019)**	9	654	8	1002	23.9%	1.73 [0.67, 4.52]
PV (Párnická, 2013)	3	86	2	226	12.7%	4.05 [0.66, 24.66]
PV (Shams, 2008)	2	104	12	360	15.8%	0.57 [0.13, 2.58]
PV, Egypt (Haase, 2015)*	0	94	2	146	5.8%	0.31 [0.01, 6.44]
PV, German (Haase, 2015)*	0	92	2	148	5.8%	0.32 [0.02, 6.67]
**Total (95% CI)**		**1338**		**7838**	**100.0%**	**1.14 [0.51, 2.53]**
Total events	17		99			

Heterogeneity: Tau² = 0.46; Chi² = 12.86, df = 9 (P = 0.17); I² = 30%

Test for overall effect: Z = 0.31 (P = 0.76)

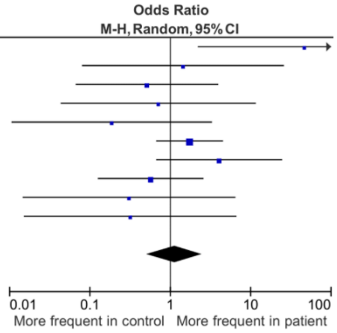

Figure S 21: Allele and genotype frequency of HLA-DRB1*10 in all diseases

DRB1*11

Genotype frequency

Study or Subgroup	Patient Events	Patient Total	Control Events	Control Total	Weight	Odds Ratio M-H, Random, 95% CI
Fogo selvagem (Pavoni, 2003)	3	128	88	402	4.4%	0.09 [0.03, 0.28]
MuSK MG (Kanai, 2016)	2	14	5	100	3.5%	3.17 [0.55, 18.16]
MuSK MG (Nikolic, 2014)	7	31	617	1992	4.9%	0.65 [0.28, 1.52]
Pemphigus (Birol, 2002) ‡	6	33	40	100	4.7%	0.33 [0.13, 0.88]
Pemphigus (Koc, 2012) ‡	5	60	30	60	4.6%	0.09 [0.03, 0.26]
Pemphigus (Lee, 1998)**	0	30	4	100	2.1%	0.35 [0.02, 6.72]
Pemphigus (Zhang, 2019)**	31	327	81	501	5.4%	0.54 [0.35, 0.84]
PF (Martel, 2002)	3	31	14	84	4.1%	0.54 [0.14, 2.01]
PV (Dere, 2020)	7	30	13	30	4.5%	0.40 [0.13, 1.21]
PV (Gil, 2017)	11	102	52	594	5.1%	1.26 [0.63, 2.51]
PV (Gonzalez-Escribano, 1998)	7	26	44	200	4.8%	1.31 [0.52, 3.31]
PV (Harfouch, 2014)	18	91	136	270	5.2%	0.24 [0.14, 0.43]
PV (Khan, 2015)	2	28	28	150	3.9%	0.34 [0.08, 1.50]
PV (Lombardi, 1996)	11	33	54	102	4.9%	0.44 [0.20, 1.01]
PV (Priyadarshini, 2018)	2	50	9	50	3.7%	0.19 [0.04, 0.93]
PV (Thomas, 1998)	7	26	44	200	4.8%	1.31 [0.52, 3.31]
PV (Tunca, 2010)	6	25	41	113	4.7%	0.55 [0.21, 1.50]
TTP (Al Haddad, 2019)	23	30	25	30	4.2%	0.66 [0.18, 2.36]
TTP (Coppo, 2010)	38	61	39	172	5.2%	5.63 [3.00, 10.57]
TTP (John, 2011)	26	54	2681	11407	5.3%	3.02 [1.77, 5.16]
TTP (Scully, 2010)	22	50	24	200	5.1%	5.76 [2.85, 11.63]
TTP (Sinkovits, 2017)	42	75	59	204	5.2%	3.13 [1.81, 5.41]
**Total (95% CI)**		**1335**		**17061**	**100.0%**	**0.74 [0.43, 1.26]**
Total events	279		4128			

Heterogeneity: Tau² = 1.34; Chi² = 182.18, df = 21 (P < 0.00001); I² = 88%

Test for overall effect: Z = 1.11 (P = 0.27)

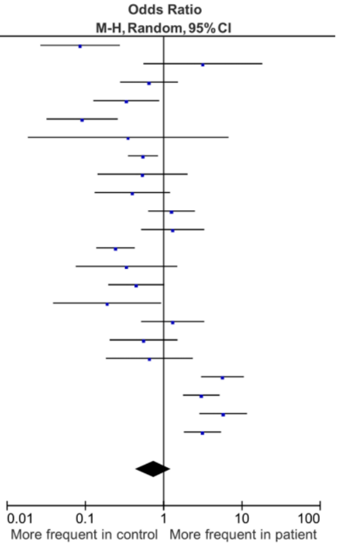

Allele frequency

Study or Subgroup	Patient Events	Patient Total	Control Events	Control Total	Weight	Odds Ratio M-H, Random, 95% CI
CIDP (Piccinelli, 2019) ‡	15	48	111	432	7.2%	1.31 [0.69, 2.51]
CIDP, NF155 (Ogata, 2020)	2	44	23	836	3.2%	1.68 [0.38, 7.38]
MuSK MG (Ehsan, 2015)	5	48	100	400	5.4%	0.35 [0.13, 0.91]
MuSK MG (Nikolic, 2014)	7	62	637	3984	6.3%	0.67 [0.30, 1.48]
Pemphigus (Brochado, 2016) **	10	336	258	3184	7.3%	0.35 [0.18, 0.66]
Pemphigus (Torzecka, 2003)**	14	106	42	304	7.2%	0.95 [0.50, 1.82]
Pemphigus (Zhang, 2019)**	7	144	86	1002	6.3%	0.54 [0.25, 1.20]
PF (Abida, 2009)	4	180	73	540	5.0%	0.15 [0.05, 0.40]
PV (Párnická, 2013)	9	86	35	226	6.4%	0.64 [0.29, 1.39]
PV (Rangel-Gamboa, 2015) ‡	2	86	20	198	3.2%	0.21 [0.05, 0.93]
PV (Shams, 2008)	14	104	89	360	7.5%	0.47 [0.26, 0.87]
PV (Zivanovic, 2016)	10	144	673	3984	7.2%	0.37 [0.19, 0.70]
PV, Egypt (Haase, 2015)*	3	94	18	146	4.0%	0.23 [0.07, 0.82]
PV, Europe (Delgado, 1997)	0	38	2	26	1.0%	0.13 [0.01, 2.76]
PV, German (Haase, 2015)*	13	92	11	148	6.0%	2.05 [0.88, 4.79]
TTP (Sakai, 2020)	5	104	38	1046	5.4%	1.34 [0.52, 3.48]
TTP, African patients (Martino, 2016)	6	52	201	1326	5.9%	0.73 [0.31, 1.73]
TTP, Caribbean patients (Martino, 2016)	6	48	32	200	5.5%	0.75 [0.29, 1.91]
**Total (95% CI)**		**1816**		**18342**	**100.0%**	**0.59 [0.43, 0.82]**
Total events	132		2449			

Heterogeneity: Tau² = 0.26; Chi² = 41.23, df = 17 (P = 0.0009); I² = 59%

Test for overall effect: Z = 3.19 (P = 0.001)

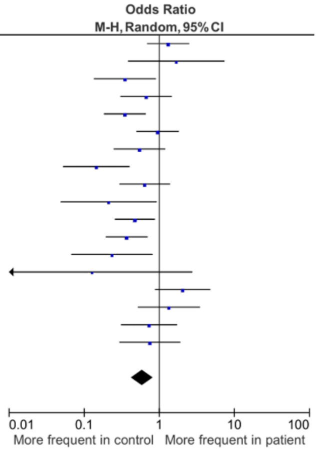

Figure S 22: Allele and genotype frequency of HLA-DRB1*11 in all diseases

DRB1*12

Genotype frequency

Study or Subgroup	Patients Events	Patients Total	Control Events	Control Total	Weight	Odds Ratio M-H, Random, 95% CI
Fogo selvagem (Pavoni, 2003)	7	128	8	402	9.6%	2.85 [1.01, 8.02]
MuSK MG (Kanai, 2016)	1	14	7	100	3.1%	1.02 [0.12, 8.99]
MuSK MG (Nikolic, 2014)	2	31	76	1992	6.0%	1.74 [0.41, 7.42]
Pemphigus (Lee, 1998)**	0	30	12	100	1.9%	0.12 [0.01, 2.02]
Pemphigus (Zhang, 2019)**	84	327	102	501	21.9%	1.35 [0.97, 1.88]
PF (Martel, 2002)	2	31	1	84	2.5%	5.72 [0.50, 65.50]
PV (Dere, 2020)	1	30	1	30	1.9%	1.00 [0.06, 16.76]
PV (Gil, 2017)	1	102	16	594	3.4%	0.36 [0.05, 2.73]
PV (Harfouch, 2014)	0	91	6	270	1.8%	0.22 [0.01, 3.99]
PV (Khan, 2015)	6	28	23	150	9.9%	1.51 [0.55, 4.12]
PV (Priyadarshini, 2018)	3	50	10	50	6.6%	0.26 [0.07, 0.99]
PV (Thomas, 1998)	0	26	7	200	1.8%	0.49 [0.03, 8.77]
PV (Tunca, 2010)	0	25	3	113	1.7%	0.62 [0.03, 12.36]
TTP (Al Haddad, 2019)	3	30	0	30	1.7%	7.76 [0.38, 157.14]
TTP (Coppo, 2010)	2	61	2	172	3.6%	2.88 [0.40, 20.92]
TTP (John, 2011)	2	54	422	11407	6.2%	1.00 [0.24, 4.12]
TTP (Scully, 2010)	5	50	4	200	6.6%	5.44 [1.41, 21.09]
TTP (Sinkovits, 2017)	7	75	9	204	9.7%	2.23 [0.80, 6.22]
**Total (95% CI)**		**1183**		**16599**	**100.0%**	**1.39 [0.93, 2.09]**
Total events	126		709			

Heterogeneity: Tau² = 0.17; Chi² = 23.48, df = 17 (P = 0.13); I² = 28%

Test for overall effect: Z = 1.61 (P = 0.11)

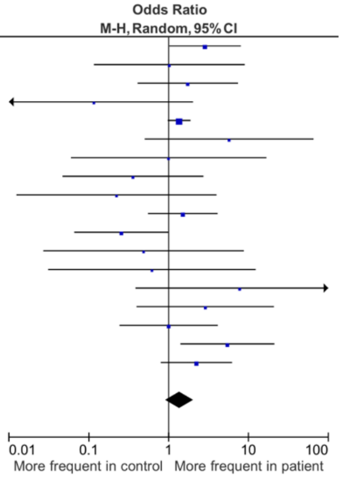

Allele frequency

Study or Subgroup	Patients Events	Patients Total	Control Events	Control Total	Weight	Odds Ratio M-H, Random, 95% CI
CIDP, NF155 (Ogata, 2020)	3	44	48	836	4.4%	1.20 [0.36, 4.02]
MuSK MG (Ehsan, 2015)	1	48	6	400	1.4%	1.40 [0.16, 11.86]
MuSK MG (Nikolic, 2014)	2	62	76	3984	3.1%	1.71 [0.41, 7.14]
Pemphigus (Torzecka, 2003)**	0	106	14	304	0.8%	0.09 [0.01, 1.59]
Pemphigus (Zhang, 2019)**	87	654	126	1002	74.8%	1.07 [0.80, 1.43]
PV (Párnická, 2013)	0	86	3	226	0.7%	0.37 [0.02, 7.22]
PV (Shams, 2008)	0	104	3	360	0.7%	0.49 [0.03, 9.54]
PV (Zivanovic, 2016)	1	144	76	3984	1.6%	0.36 [0.05, 2.60]
PV, Egypt (Haase, 2015)*	0	94	1	146	0.6%	0.51 [0.02, 12.73]
PV, Europe (Delgado, 1997)	1	38	12	496	1.5%	1.09 [0.14, 8.61]
PV, German (Haase, 2015)*	0	92	4	148	0.7%	0.17 [0.01, 3.26]
TTP (Sakai, 2020)	7	104	48	1046	9.5%	1.50 [0.66, 3.41]
**Total (95% CI)**		**1576**		**12932**	**100.0%**	**1.05 [0.82, 1.36]**
Total events	102		417			

Heterogeneity: Tau² = 0.00; Chi² = 7.92, df = 11 (P = 0.72); I² = 0%

Test for overall effect: Z = 0.41 (P = 0.68)

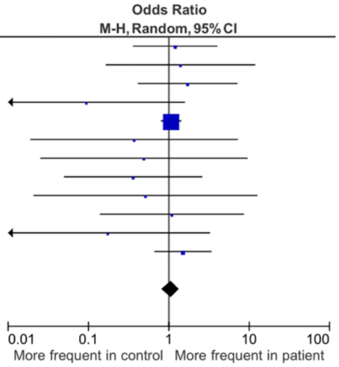

Figure S 23: Allele and genotype frequency of HLA-DRB1*12 in all diseases

DRB1*15

Genotype frequency

Study or Subgroup	Patient Events	Patient Total	Control Events	Control Total	Weight	Odds Ratio M-H, Random, 95% CI
CIDP, NF155 (Martinez-Martinez, 2017)	10	13	160	941	4.6%	16.27 [4.43, 59.78]
Fogo selvagem (Moraes, 1991)	2	38	11	46	3.8%	0.18 [0.04, 0.86]
Fogo selvagem (Pavoni, 2003)	18	128	97	402	7.3%	0.51 [0.30, 0.89]
MuSK MG (Kanai, 2016)	6	14	32	100	5.1%	1.59 [0.51, 4.98]
MuSK MG (Nikolic, 2014)	4	31	371	1992	5.4%	0.65 [0.23, 1.86]
Pemphigus (Lee, 1998)**	3	30	23	100	4.7%	0.37 [0.10, 1.34]
Pemphigus (Zhang, 2019)**	56	327	139	501	7.8%	0.54 [0.38, 0.76]
PF (Martel, 2002)	2	31	16	84	3.9%	0.29 [0.06, 1.36]
PV (Dere, 2020)	5	30	8	30	4.7%	0.55 [0.16, 1.93]
PV (Gil, 2017)	3	102	61	594	5.0%	0.26 [0.08, 0.86]
PV (Glorio, 1999)	1	30	39	199	2.8%	0.14 [0.02, 1.07]
PV (Glorio, 2002)	1	47	39	199	2.8%	0.09 [0.01, 0.67]
PV (Khan, 2015)	8	28	59	150	6.0%	0.62 [0.26, 1.49]
PV (Lombardi, 1996)	3	33	10	102	4.4%	0.92 [0.24, 3.56]
PV (Priyadarshini, 2018)	7	50	16	50	5.6%	0.35 [0.13, 0.94]
PV (Tunca, 2010)	2	25	20	113	3.9%	0.40 [0.09, 1.86]
TTP (Al Haddad, 2019)	3	30	0	30	1.6%	7.76 [0.38, 157.14]
TTP (Coppo, 2010)	16	61	33	172	6.8%	1.50 [0.75, 2.97]
TTP (Scully, 2010)	15	50	54	200	6.8%	1.16 [0.59, 2.29]
TTP (Sinkovits, 2017)	21	75	29	204	6.9%	2.35 [1.24, 4.45]
**Total (95% CI)**		**1173**		**6209**	**100.0%**	**0.71 [0.47, 1.07]**
Total events	186		1217			

Heterogeneity: Tau² = 0.54; Chi² = 68.20, df = 19 (P < 0.00001); I² = 72%

Test for overall effect: Z = 1.63 (P = 0.10)

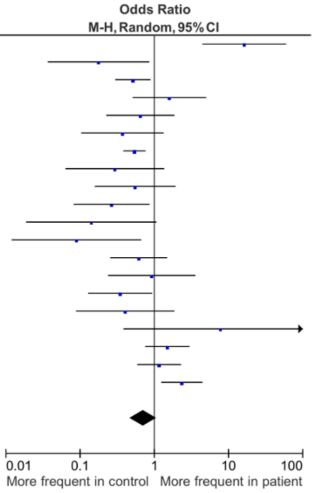

Allele frequency

Study or Subgroup	Patient Events	Patient Total	Control Events	Control Total	Weight	Odds Ratio M-H, Random, 95% CI
CIDP (Piccinelli, 2019) ‡	2	48	24	432	4.9%	0.74 [0.17, 3.23]
CIDP, NF155 (Ogata, 2020)	23	44	137	836	7.8%	5.59 [3.01, 10.38]
MuSK MG (Ehsan, 2015)	6	48	48	400	6.8%	1.05 [0.42, 2.59]
MuSK MG (Nikolic, 2014)	4	62	392	3984	6.4%	0.63 [0.23, 1.75]
Pemphigus (Torzecka, 2003)**	7	106	51	304	7.1%	0.35 [0.15, 0.80]
Pemphigus (Zhang, 2019)**	58	654	178	1002	8.6%	0.45 [0.33, 0.62]
PF (Abida, 2009)	8	180	56	540	7.3%	0.40 [0.19, 0.86]
PV (Párnická, 2013)	2	86	29	226	4.9%	0.16 [0.04, 0.69]
PV (Shams, 2008)	4	104	47	360	6.3%	0.27 [0.09, 0.76]
PV (Zivanovic, 2016)	5	144	392	3984	6.8%	0.33 [0.13, 0.81]
PV, Egypt (Haase, 2015)*	2	94	17	146	4.8%	0.16 [0.04, 0.73]
PV, German (Haase, 2015)*	6	92	18	148	6.6%	0.50 [0.19, 1.32]
PV, Indo-Asians (Saha, 2010)*	5	114	25	118	6.5%	0.17 [0.06, 0.46]
PV, White Europeans (Saha, 2010)*	8	192	25	200	7.1%	0.30 [0.13, 0.69]
TTP (Sakai, 2020)	16	104	212	1046	8.0%	0.72 [0.41, 1.24]
**Total (95% CI)**		**2072**		**13726**	**100.0%**	**0.48 [0.30, 0.79]**
Total events	156		1651			

Heterogeneity: Tau² = 0.69; Chi² = 76.54, df = 14 (P < 0.00001); I² = 82%

Test for overall effect: Z = 2.93 (P = 0.003)

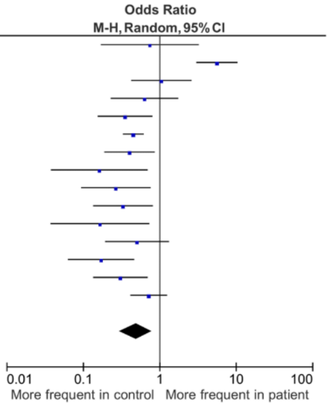

Figure S 24: Allele and genotype frequency of HLA-DRB1*15 in all diseases

DRB1*16

Genotype frequency

Study or Subgroup	Patients Events	Patients Total	Control Events	Control Total	Weight	Odds Ratio M-H, Random, 95% CI
Fogo selvagem (Cerna, 1993)	4	10	38	74	5.5%	0.63 [0.16, 2.42]
Fogo selvagem (Moraes, 1991)	8	37	2	49	4.7%	6.48 [1.29, 32.66]
Fogo selvagem (Pavoni, 2003)	21	128	39	402	8.3%	1.83 [1.03, 3.24]
MuSK MG (Alahgholi-Hajibehzad, 2013)	18	48	27	250	7.9%	4.96 [2.44, 10.06]
MuSK MG (Kanai, 2016)	2	14	2	100	3.6%	8.17 [1.05, 63.41]
MuSK MG (Nikolic, 2014)	17	31	406	1992	7.8%	4.74 [2.32, 9.70]
MuSK MG (Niks, 2006)	2	23	43	2440	5.1%	5.31 [1.21, 23.36]
Pemphigus (Lee, 1998)**	1	30	2	100	2.9%	1.69 [0.15, 19.31]
Pemphigus (Zhang, 2019)**	0	327	7	501	2.3%	0.10 [0.01, 1.77]
PF (Martel, 2002)	4	31	3	84	4.8%	4.00 [0.84, 19.02]
PV (Gil, 2017)	2	102	19	594	5.1%	0.61 [0.14, 2.64]
PV (Harfouch, 2014)	3	91	23	270	5.9%	0.37 [0.11, 1.25]
PV (Khan, 2015)	0	28	4	150	2.2%	0.57 [0.03, 10.90]
PV (Lombardi, 1996)	3	33	8	102	5.4%	1.18 [0.29, 4.71]
PV (Priyadarshini, 2018)	1	50	0	50	1.9%	3.06 [0.12, 76.95]
PV (Tunca, 2010)	0	25	25	113	2.3%	0.07 [0.00, 1.16]
TTP (Al Haddad, 2019)	10	30	8	30	6.4%	1.38 [0.45, 4.17]
TTP (Coppo, 2010)	3	61	11	172	5.6%	0.76 [0.20, 2.81]
TTP (Scully, 2010)	3	50	5	200	5.1%	2.49 [0.57, 10.79]
TTP (Sinkovits, 2017)	7	75	26	204	7.2%	0.70 [0.29, 1.70]
**Total (95% CI)**		**1224**		**7877**	**100.0%**	**1.56 [0.95, 2.56]**
Total events	109		698			

Heterogeneity: Tau² = 0.68; Chi² = 52.70, df = 19 (P < 0.0001); I² = 64%

Test for overall effect: Z = 1.77 (P = 0.08)

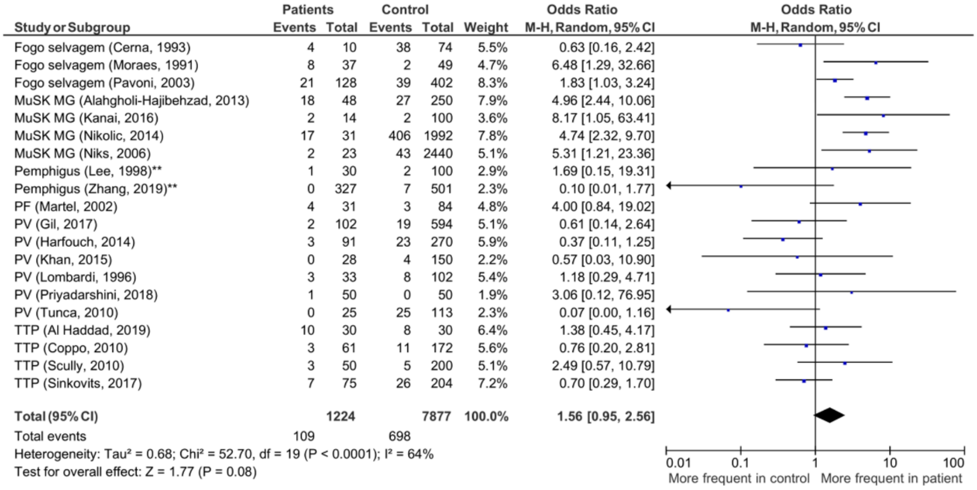

Allele frequency

Study or Subgroup	Patients Events	Patients Total	Control Events	Control Total	Weight	Odds Ratio M-H, Random, 95% CI
CIDP (Piccinelli, 2019) ‡	2	48	19	432	11.0%	0.95 [0.21, 4.19]
CIDP, NF155 (Ogata, 2020)	0	44	2	836	5.6%	3.75 [0.18, 79.30]
Fogo selvagem (de Sena Nogueira Maehara, 2017)	10	84	36	956	14.3%	3.45 [1.65, 7.23]
MuSK MG (Nikolic, 2014)	18	62	433	3984	14.9%	3.35 [1.92, 5.86]
Pemphigus (Zhang, 2019)**	0	654	8	1002	6.1%	0.09 [0.01, 1.55]
PV (Párnická, 2013)	1	86	14	226	8.7%	0.18 [0.02, 1.38]
PV (Rangel-Gamboa, 2015) ‡	2	86	5	198	10.3%	0.92 [0.17, 4.83]
PV (Shams, 2008)	1	104	16	360	8.8%	0.21 [0.03, 1.59]
PV (Zivanovic, 2016)	8	144	433	3984	14.3%	0.48 [0.23, 0.99]
PV, Egypt (Haase, 2015)*	0	94	0	146		Not estimable
PV, German (Haase, 2015)*	0	92	4	148	5.9%	0.17 [0.01, 3.26]
**Total (95% CI)**		**1498**		**12272**	**100.0%**	**0.80 [0.32, 1.97]**
Total events	42		970			

Heterogeneity: Tau² = 1.35; Chi² = 42.94, df = 9 (P < 0.00001); I² = 79%

Test for overall effect: Z = 0.49 (P = 0.62)

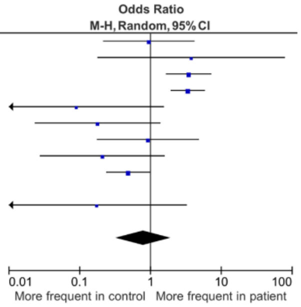

Figure S 25: Allele and genotype frequency of HLA-DRB1*16 in all diseases

DQB1*04

Genotype frequency

Study or Subgroup	Patients Events	Patients Total	Control Events	Control Total	Weight	Odds Ratio M-H, Random, 95% CI
Fogo selvagem (Cerna, 1993)	1	10	34	74	4.8%	0.13 [0.02, 1.08]
MuSK MG (Kanai, 2016)	2	14	37	100	6.8%	0.28 [0.06, 1.34]
MuSK MG (Nikolic, 2014)	3	31	28	159	8.2%	0.50 [0.14, 1.76]
Pemphigus (Birol, 2002) ‡	3	33	1	100	4.3%	9.90 [0.99, 98.72]
Pemphigus (Lee, 1998)**	8	30	25	100	9.9%	1.09 [0.43, 2.76]
Pemphigus (Zhang, 2019)**	15	327	57	501	11.7%	0.37 [0.21, 0.67]
PV (Dere, 2020)	0	30	1	30	2.6%	0.32 [0.01, 8.24]
PV (Gil, 2017)	8	102	46	594	10.7%	1.01 [0.46, 2.22]
PV (Lombardi, 1996)	4	33	1	102	4.5%	13.93 [1.50, 129.54]
PV (Orouji, 2014)	0	54	2	85	2.8%	0.31 [0.01, 6.51]
PV (Priyadarshini, 2018)	3	50	1	50	4.3%	3.13 [0.31, 31.14]
PV (Tunca, 2010)	2	25	7	113	6.5%	1.32 [0.26, 6.75]
TTP (Al Haddad, 2019)	0	30	1	30	2.6%	0.32 [0.01, 8.24]
TTP (Coppo, 2010)	3	60	7	172	7.6%	1.24 [0.31, 4.96]
TTP (John, 2011)	1	54	3	174	4.3%	1.08 [0.11, 10.56]
TTP (Scully, 2010)	6	50	5	200	8.3%	5.32 [1.55, 18.21]
**Total (95% CI)**		**933**		**2584**	**100.0%**	**1.01 [0.56, 1.80]**
Total events	59		256			

Heterogeneity: Tau² = 0.66; Chi² = 35.56, df = 15 (P = 0.002); I² = 58%

Test for overall effect: Z = 0.02 (P = 0.99)

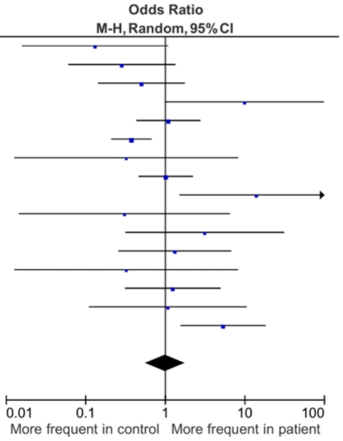

Allele frequency

Study or Subgroup	Patients Events	Patients Total	Control Events	Control Total	Weight	Odds Ratio M-H, Random, 95% CI
CIDP (Piccinelli, 2019) ‡	0	48	16	432	4.3%	0.26 [0.02, 4.41]
CIDP, NF155 (Ogata, 2020)	1	44	148	836	7.5%	0.11 [0.01, 0.79]
MuSK MG (Nikolic, 2014)	3	62	7	318	11.9%	2.26 [0.57, 8.99]
Pemphigus (Zhang, 2019)**	16	654	65	1002	21.9%	0.36 [0.21, 0.63]
PV (Orouji, 2014)	0	108	2	170	3.8%	0.31 [0.01, 6.53]
PV (Párnická, 2013)	3	86	6	226	11.6%	1.33 [0.32, 5.42]
PV (Shams, 2008)	2	104	6	360	9.9%	1.16 [0.23, 5.82]
PV (Zivanovic, 2016)	1	144	7	318	6.9%	0.31 [0.04, 2.55]
TTP (Sakai, 2020)	19	104	174	1046	22.3%	1.12 [0.66, 1.89]
**Total (95% CI)**		**1354**		**4708**	**100.0%**	**0.67 [0.35, 1.27]**
Total events	45		431			

Heterogeneity: Tau² = 0.41; Chi² = 17.98, df = 8 (P = 0.02); I² = 56%

Test for overall effect: Z = 1.23 (P = 0.22)

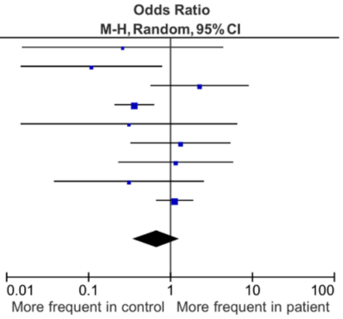

Figure S 26: Allele and genotype frequency of HLA-DQB1*04 in all diseases

DRB1*16-DQB1*05

n

Study or Subgroup	Patients Events	Patients Total	Control Events	Control Total	Weight	Odds Ratio M-H, Random, 95% CI
MuSK MG (Alahgholi-Hajibehzad, 2013)	16	48	27	250	40.4%	4.13 [2.01, 8.49]
MuSK MG (Kanai, 2016)	2	14	2	100	21.9%	8.17 [1.05, 63.41]
TTP (Sinkovits, 2017)	7	75	18	162	37.6%	0.82 [0.33, 2.07]
**Total (95% CI)**		**137**		**512**	**100.0%**	**2.61 [0.69, 9.87]**
Total events	25		47			

Heterogeneity: Tau² = 1.00; Chi² = 8.93, df = 2 (P = 0.01); I² = 78%

Test for overall effect: Z = 1.42 (P = 0.16)

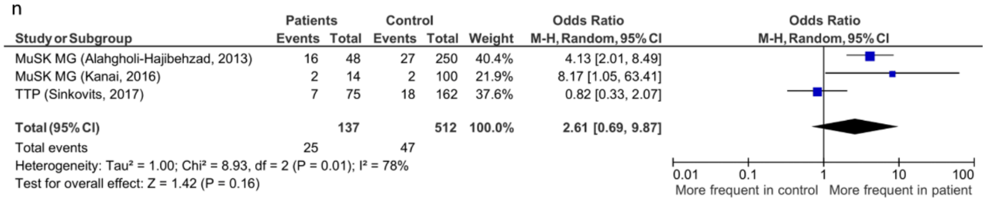

2n

Study or Subgroup	Patients Events	Patients Total	Control Events	Control Total	Weight	Odds Ratio M-H, Random, 95% CI
MuSK MG (Ehsan, 2015)	11	48	26	400	18.9%	4.28 [1.96, 9.35]
MuSK MG (Nikolic, 2014)	18	62	39	318	19.9%	2.93 [1.54, 5.56]
MuSK MG (Niks, 2006)	2	46	7	642	12.4%	4.12 [0.83, 20.44]
Pemphigus (Brochado, 2016) **	14	336	85	3184	20.4%	1.59 [0.89, 2.82]
PV (Shams, 2008)	1	104	16	360	9.7%	0.21 [0.03, 1.59]
PV (Zivanovic, 2016)	8	144	39	318	18.8%	0.42 [0.19, 0.93]
**Total (95% CI)**		**740**		**5222**	**100.0%**	**1.56 [0.67, 3.59]**
Total events	54		212			

Heterogeneity: Tau² = 0.81; Chi² = 27.04, df = 5 (P < 0.0001); I² = 82%

Test for overall effect: Z = 1.04 (P = 0.30)

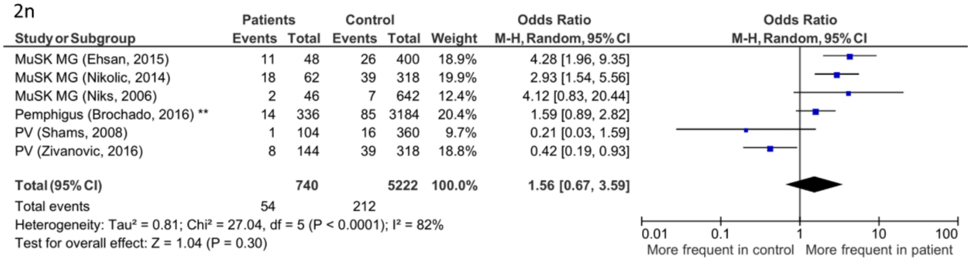

Figure S 27: Allele and genotype frequency of HLA-DRB1*16-DQB1*05 in all diseases

DRB1*15-DQB1*06

n

Study or Subgroup	Patients Events	Patients Total	Control Events	Control Total	Weight	Odds Ratio M-H, Random, 95% CI
CIDP, NF155 (Martinez-Martinez, 2017)	8	13	5	35	25.6%	9.60 [2.22, 41.52]
PV (Mobini, 1997)	0	38	10	57	17.4%	0.06 [0.00, 1.03]
PV (Priyadarshini, 2018)	5	50	15	50	27.6%	0.26 [0.09, 0.78]
TTP (Sinkovits, 2017)	19	75	19	162	29.4%	2.55 [1.26, 5.18]
**Total (95% CI)**		**176**		**304**	**100.0%**	**0.99 [0.16, 6.06]**
Total events	32		49			

Heterogeneity: Tau² = 2.78; Chi² = 24.02, df = 3 (P < 0.0001); I² = 88%

Test for overall effect: Z = 0.01 (P = 0.99)

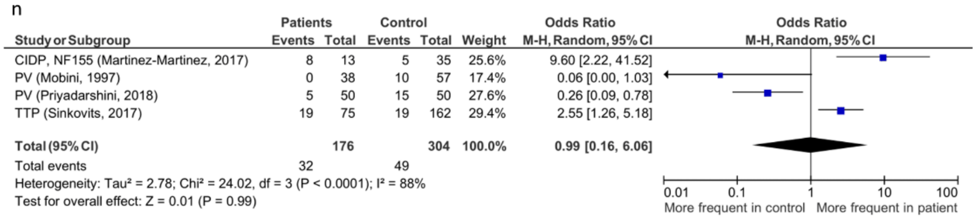

2n

Study or Subgroup	Patients Events	Patients Total	Control Events	Control Total	Weight	Odds Ratio M-H, Random, 95% CI
CIDP (Piccinelli, 2019) ‡	1	48	20	432	8.6%	0.44 [0.06, 3.34]
CIDP, NF155 (Ogata, 2020)	23	44	135	836	16.9%	5.69 [3.06, 10.57]
MuSK MG (Ehsan, 2015)	5	48	44	400	14.7%	0.94 [0.35, 2.50]
MuSK MG (Nikolic, 2014)	4	62	19	318	13.8%	1.09 [0.36, 3.31]
Pemphigus (Brochado, 2016) **	20	336	282	3184	17.6%	0.65 [0.41, 1.04]
PV (Shams, 2008)	4	104	17	360	13.8%	0.81 [0.27, 2.45]
PV (Zivanovic, 2016)	5	144	19	318	14.5%	0.57 [0.21, 1.55]
**Total (95% CI)**		**786**		**5848**	**100.0%**	**1.04 [0.46, 2.33]**
Total events	62		536			

Heterogeneity: Tau² = 0.92; Chi² = 35.05, df = 6 (P < 0.00001); I² = 83%

Test for overall effect: Z = 0.09 (P = 0.93)

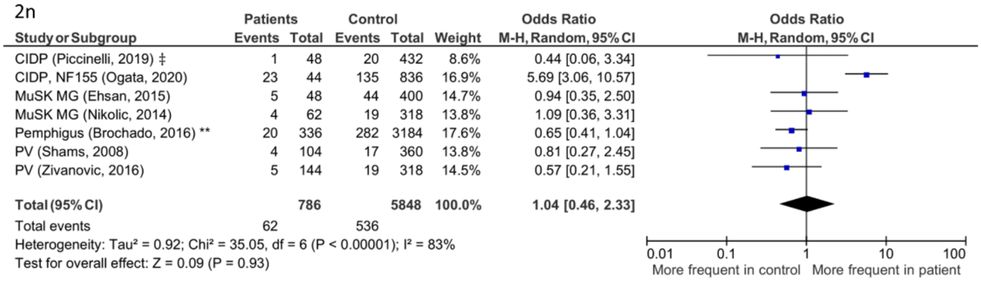

Figure S 28: Allele and genotype frequency of HLA-DRB1*15-DQB1*16 in all diseases

DRB1*01

Genotype frequency

Study or Subgroup	Patients Events	Patients Total	Control Events	Control Total	Weight	Odds Ratio M-H, Random, 95% CI
MuSK MG (Kanai, 2016)	1	14	15	100	10.3%	0.44 [0.05, 3.58]
MuSK MG (Nikolic, 2014)	3	31	386	1992	12.5%	0.45 [0.13, 1.47]
MuSK MG (Niks, 2006)	9	23	14	2440	12.9%	111.40 [41.45, 299.36]
TTP (Al Haddad, 2019)	8	30	2	30	11.4%	5.09 [0.98, 26.43]
TTP (Coppo, 2010)	11	61	32	172	13.3%	0.96 [0.45, 2.05]
TTP (John, 2011)	7	54	2395	11407	13.2%	0.56 [0.25, 1.24]
TTP (Scully, 2010)	9	50	38	200	13.2%	0.94 [0.42, 2.09]
TTP (Sinkovits, 2017)	8	75	38	204	13.2%	0.52 [0.23, 1.18]
**Total (95% CI)**		**338**		**16545**	**100.0%**	**1.54 [0.41, 5.76]**
Total events	56		2920			

Heterogeneity: Tau² = 3.26; Chi² = 98.76, df = 7 (P < 0.00001); I² = 93%
Test for overall effect: Z = 0.64 (P = 0.52)

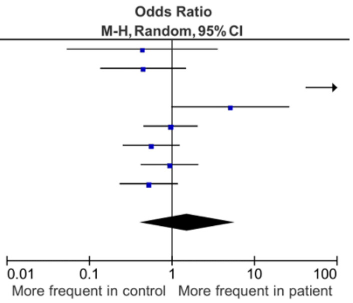

Allele frequency

Study or Subgroup	Patients Events	Patients Total	Control Events	Control Total	Weight	Odds Ratio M-H, Random, 95% CI
CIDP (Piccinelli, 2019) ‡	5	48	48	432	24.6%	0.93 [0.35, 2.46]
CIDP, NF155 (Ogata, 2020)	2	44	57	836	13.2%	0.65 [0.15, 2.76]
MuSK MG (Ehsan, 2015)	4	48	22	400	20.2%	1.56 [0.51, 4.74]
MuSK MG (Nikolic, 2014)	2	62	410	3984	13.7%	0.29 [0.07, 1.19]
TTP (Sakai, 2020)	6	104	40	1046	28.2%	1.54 [0.64, 3.72]
**Total (95% CI)**		**306**		**6698**	**100.0%**	**0.97 [0.55, 1.71]**
Total events	19		577			

Heterogeneity: Tau² = 0.10; Chi² = 5.19, df = 4 (P = 0.27); I² = 23%
Test for overall effect: Z = 0.11 (P = 0.91)

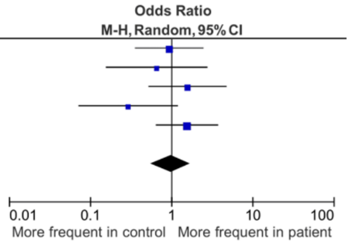

Figure S 29: Allele and genotype frequency of HLA-DRB1*01 in MuSK MG, TTP and CIDP

DRB1*03

Genotype frequency

Study or Subgroup	Patients Events	Patients Total	Control Events	Control Total	Weight	Odds Ratio M-H, Random, 95% CI
MuSK MG (Alahgholi-Hajibehzad, 2013)	1	48	44	250	5.9%	0.10 [0.01, 0.74]
MuSK MG (Nikolic, 2014)	5	31	418	1992	12.7%	0.72 [0.28, 1.90]
MuSK MG (Niks, 2006)	2	23	21	2440	8.4%	10.97 [2.42, 49.79]
TTP (Al Haddad, 2019)	18	30	8	30	11.6%	4.13 [1.39, 12.27]
TTP (Coppo, 2010)	10	61	32	172	14.5%	0.86 [0.39, 1.87]
TTP (John, 2011)	15	54	2281	11407	16.2%	1.54 [0.85, 2.80]
TTP (Scully, 2010)	11	50	50	200	14.8%	0.85 [0.40, 1.78]
TTP (Sinkovits, 2017)	18	75	41	204	15.9%	1.26 [0.67, 2.36]
**Total (95% CI)**		**372**		**16695**	**100.0%**	**1.28 [0.72, 2.29]**
Total events	80		2895			

Heterogeneity: Tau² = 0.45; Chi² = 23.34, df = 7 (P = 0.001); I² = 70%

Test for overall effect: Z = 0.84 (P = 0.40)

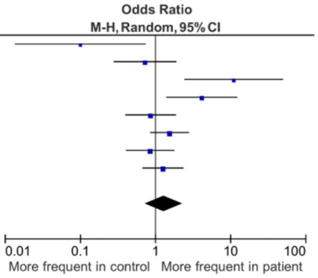

Allele frequency

Study or Subgroup	Patients Events	Patients Total	Control Events	Control Total	Weight	Odds Ratio M-H, Random, 95% CI
CIDP (Piccinelli, 2019) ‡	6	48	17	432	32.5%	3.49 [1.30, 9.32]
CIDP, NF155 (Ogata, 2020)	0	44	1	836	12.8%	6.26 [0.25, 155.82]
MuSK MG (Ehsan, 2015)	1	48	40	400	21.6%	0.19 [0.03, 1.43]
MuSK MG (Nikolic, 2014)	5	62	433	3984	33.1%	0.72 [0.29, 1.80]
**Total (95% CI)**		**202**		**5652**	**100.0%**	**1.19 [0.29, 4.87]**
Total events	12		491			

Heterogeneity: Tau² = 1.34; Chi² = 11.08, df = 3 (P = 0.01); I² = 73%

Test for overall effect: Z = 0.24 (P = 0.81)

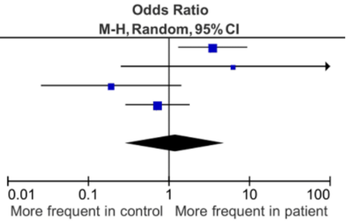

Figure S 30: Allele and genotype frequency of HLA-DRB1*03 in MuSK MG, TTP and CIDP

DRB1*04

Genotype frequency

Study or Subgroup	Patients Events	Patients Total	Control Events	Control Total	Weight	Odds Ratio M-H, Random, 95% CI
MuSK MG (Kanai, 2016)	4	14	44	100	8.9%	0.51 [0.15, 1.73]
MuSK MG (Nikolic, 2014)	2	31	339	1992	6.5%	0.34 [0.08, 1.42]
TTP (Al Haddad, 2019)	10	30	16	30	12.3%	0.44 [0.15, 1.24]
TTP (Coppo, 2010)	6	61	48	172	16.3%	0.28 [0.11, 0.70]
TTP (John, 2011)	7	54	2395	11407	21.2%	0.56 [0.25, 1.24]
TTP (Scully, 2010)	5	50	70	200	14.3%	0.21 [0.08, 0.54]
TTP (Sinkovits, 2017)	8	75	43	204	20.6%	0.45 [0.20, 1.00]
**Total (95% CI)**		**315**		**14105**	**100.0%**	**0.39 [0.27, 0.56]**
Total events	42		2955			

Heterogeneity: Tau² = 0.00; Chi² = 3.35, df = 6 (P = 0.76); I² = 0%

Test for overall effect: Z = 5.10 (P < 0.00001)

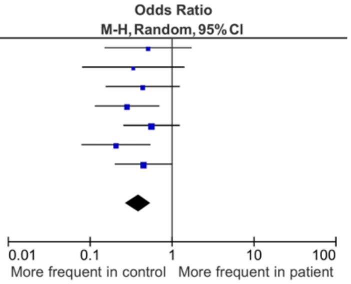

Allele frequency

Study or Subgroup	Patients Events	Patients Total	Control Events	Control Total	Weight	Odds Ratio M-H, Random, 95% CI
CIDP (Piccinelli, 2019) ‡	4	48	61	432	17.7%	0.55 [0.19, 1.59]
CIDP, NF155 (Ogata, 2020)	1	44	199	836	7.2%	0.07 [0.01, 0.54]
MuSK MG (Ehsan, 2015)	2	48	42	400	11.8%	0.37 [0.09, 1.58]
MuSK MG (Nikolic, 2014)	2	62	358	3984	12.2%	0.34 [0.08, 1.39]
TTP (Sakai, 2020)	23	104	248	1046	32.6%	0.91 [0.56, 1.48]
TTP, African patients (Martino, 2016)	1	52	36	1326	7.1%	0.70 [0.09, 5.23]
TTP, Caribbean patients (Martino, 2016)	2	48	22	200	11.4%	0.35 [0.08, 1.55]
**Total (95% CI)**		**406**		**8224**	**100.0%**	**0.49 [0.27, 0.88]**
Total events	35		966			

Heterogeneity: Tau² = 0.21; Chi² = 9.52, df = 6 (P = 0.15); I² = 37%

Test for overall effect: Z = 2.39 (P = 0.02)

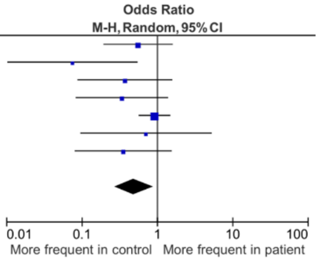

Figure S 31: Allele and genotype frequency of HLA-DRB1*04 in MuSK MG, TTP, CIDP

DRB1*07

Genotype frequency

Study or Subgroup	Patients Events	Patients Total	Control Events	Control Total	Weight	Odds Ratio M-H, Random, 95% CI
MuSK MG (Nikolic, 2014)	7	31	276	1992	16.2%	1.81 [0.77, 4.25]
TTP (Al Haddad, 2019)	7	30	9	30	12.2%	0.71 [0.22, 2.25]
TTP (Coppo, 2010)	12	61	44	172	18.3%	0.71 [0.35, 1.46]
TTP (John, 2011)	15	54	2692	11407	20.3%	1.25 [0.69, 2.26]
TTP (Scully, 2010)	7	50	59	200	16.1%	0.39 [0.17, 0.91]
TTP (Sinkovits, 2017)	8	75	55	204	17.0%	0.32 [0.15, 0.72]
**Total (95% CI)**		**301**		**14005**	**100.0%**	**0.74 [0.43, 1.26]**
Total events	56		3135			

Heterogeneity: Tau² = 0.28; Chi² = 13.68, df = 5 (P = 0.02); I² = 63%

Test for overall effect: Z = 1.11 (P = 0.27)

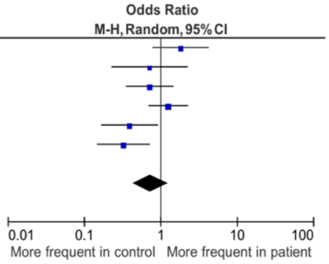

Allele frequency

Study or Subgroup	Patients Events	Patients Total	Control Events	Control Total	Weight	Odds Ratio M-H, Random, 95% CI
CIDP (Piccinelli, 2019) ‡	3	48	57	432	26.8%	0.44 [0.13, 1.46]
MuSK MG (Ehsan, 2015)	5	48	26	400	32.6%	1.67 [0.61, 4.58]
MuSK MG (Nikolic, 2014)	7	62	284	3984	40.6%	1.66 [0.75, 3.67]
**Total (95% CI)**		**158**		**4816**	**100.0%**	**1.16 [0.52, 2.59]**
Total events	15		367			

Heterogeneity: Tau² = 0.24; Chi² = 3.92, df = 2 (P = 0.14); I² = 49%

Test for overall effect: Z = 0.37 (P = 0.71)

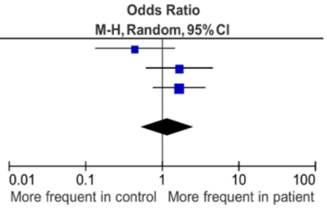

Figure S 32: Allele and genotype frequency of HLA-DRB1*07 in MuSK MG, TTP, CIDP

DRB1*08

Genotype frequency

Study or Subgroup	Patients Events	Patients Total	Control Events	Control Total	Weight	Odds Ratio M-H, Random, 95% CI
MuSK MG (Kanai, 2016)	0	14	28	100	6.1%	0.09 [0.01, 1.52]
MuSK MG (Nikolic, 2014)	3	31	123	1992	17.7%	1.63 [0.49, 5.43]
TTP (Al Haddad, 2019)	5	30	1	30	9.0%	5.80 [0.63, 53.01]
TTP (Coppo, 2010)	4	61	11	172	17.9%	1.03 [0.31, 3.35]
TTP (John, 2011)	1	54	730	11407	10.4%	0.28 [0.04, 2.00]
TTP (Scully, 2010)	6	50	8	200	18.9%	3.27 [1.08, 9.91]
TTP (Sinkovits, 2017)	5	75	18	204	19.9%	0.74 [0.26, 2.06]
**Total (95% CI)**		**315**		**14105**	**100.0%**	**1.14 [0.51, 2.51]**
Total events	24		919			

Heterogeneity: Tau² = 0.55; Chi² = 12.40, df = 6 (P = 0.05); I² = 52%

Test for overall effect: Z = 0.32 (P = 0.75)

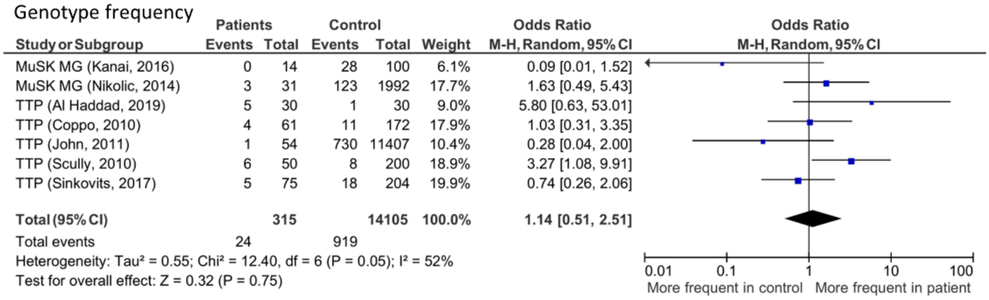

Allele frequency

Study or Subgroup	Patients Events	Patients Total	Control Events	Control Total	Weight	Odds Ratio M-H, Random, 95% CI
CIDP (Piccinelli, 2019) ‡	0	48	19	432	3.6%	0.22 [0.01, 3.68]
CIDP, NF155 (Ogata, 2020)	6	44	95	836	26.4%	1.23 [0.51, 2.99]
MuSK MG (Ehsan, 2015)	0	48	6	400	3.4%	0.63 [0.03, 11.28]
MuSK MG (Nikolic, 2014)	3	62	123	3984	17.3%	1.60 [0.49, 5.16]
TTP (Sakai, 2020)	22	104	102	1046	49.4%	2.48 [1.49, 4.15]
**Total (95% CI)**		**306**		**6698**	**100.0%**	**1.67 [0.97, 2.88]**
Total events	31		345			

Heterogeneity: Tau² = 0.09; Chi² = 5.11, df = 4 (P = 0.28); I² = 22%

Test for overall effect: Z = 1.85 (P = 0.06)

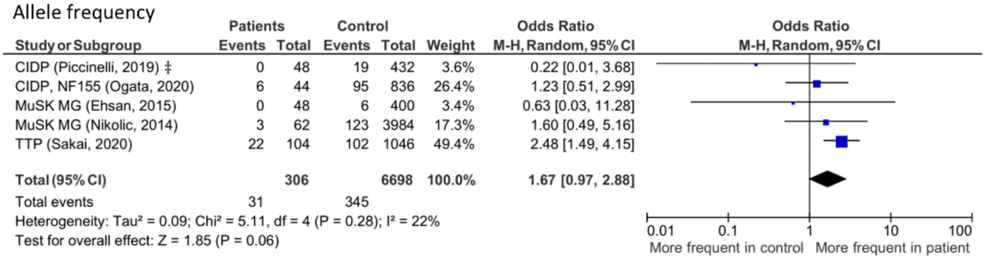

Figure S 33: Allele and genotype frequency of HLA-DRB1*08 in MuSK MG, TTP, CIDP

DRB1*09

Genotype frequency

Study or Subgroup	Patients Events	Patients Total	Control Events	Control Total	Weight	Odds Ratio M-H, Random, 95% CI
MuSK MG (Kanai, 2016)	3	14	23	100	32.5%	0.91 [0.23, 3.55]
MuSK MG (Nikolic, 2014)	0	31	14	1992	7.4%	2.17 [0.13, 37.11]
TTP (Al Haddad, 2019)	2	30	0	30	6.3%	5.35 [0.25, 116.31]
TTP (Coppo, 2010)	2	61	3	172	18.3%	1.91 [0.31, 11.71]
TTP (John, 2011)	1	54	217	11407	15.3%	0.97 [0.13, 7.07]
TTP (Scully, 2010)	1	50	6	200	13.1%	0.66 [0.08, 5.61]
TTP (Sinkovits, 2017)	0	75	4	204	7.0%	0.30 [0.02, 5.55]
**Total (95% CI)**		**315**		**14105**	**100.0%**	**1.11 [0.51, 2.42]**
Total events	9		267			

Heterogeneity: Tau² = 0.00; Chi² = 2.68, df = 6 (P = 0.85); I² = 0%

Test for overall effect: Z = 0.27 (P = 0.78)

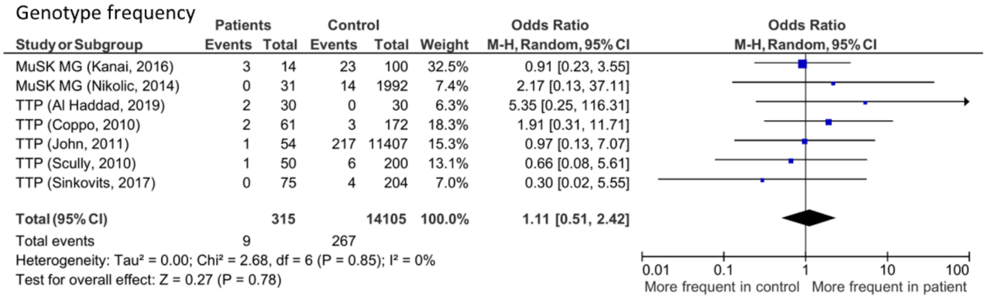

Allele frequency

Study or Subgroup	Patients Events	Patients Total	Control Events	Control Total	Weight	Odds Ratio M-H, Random, 95% CI
CIDP, NF155 (Ogata, 2020)	5	44	127	836	23.9%	0.72 [0.28, 1.85]
MuSK MG (Ehsan, 2015)	1	48	12	400	5.1%	0.69 [0.09, 5.41]
MuSK MG (Nikolic, 2014)	0	62	14	3984	2.7%	2.19 [0.13, 37.13]
TTP (Sakai, 2020)	16	104	142	1046	68.4%	1.16 [0.66, 2.03]
**Total (95% CI)**		**258**		**6266**	**100.0%**	**1.02 [0.64, 1.63]**
Total events	22		295			

Heterogeneity: Tau² = 0.00; Chi² = 1.16, df = 3 (P = 0.76); I² = 0%

Test for overall effect: Z = 0.09 (P = 0.92)

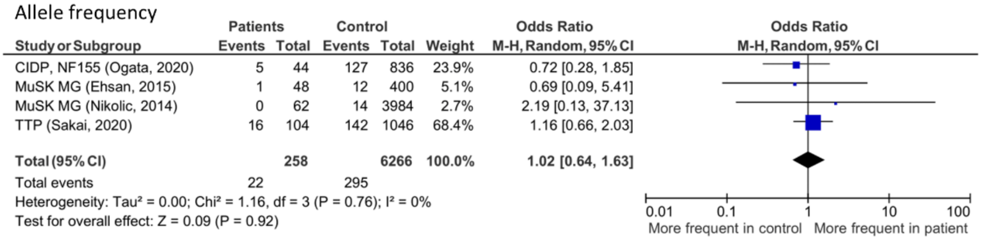

Figure S 34: Allele and genotype frequency of HLA-DRB1*09 in MuSK MG, TTP, CIDP

DRB1*10

Genotype frequency

Study or Subgroup	Patients Events	Patients Total	Control Events	Control Total	Weight	Odds Ratio M-H, Random, 95% CI
MuSK MG (Nikolic, 2014)	0	31	44	1992	11.7%	0.70 [0.04, 11.54]
TTP (Al Haddad, 2019)	2	30	3	30	26.6%	0.64 [0.10, 4.15]
TTP (Coppo, 2010)	3	61	1	172	17.8%	8.84 [0.90, 86.71]
TTP (Scully, 2010)	0	50	3	200	10.4%	0.56 [0.03, 10.99]
TTP (Sinkovits, 2017)	2	75	5	204	33.5%	1.09 [0.21, 5.74]
**Total (95% CI)**		**247**		**2598**	**100.0%**	**1.22 [0.46, 3.18]**
Total events	7		56			

Heterogeneity: Tau² = 0.00; Chi² = 3.78, df = 4 (P = 0.44); I² = 0%

Test for overall effect: Z = 0.40 (P = 0.69)

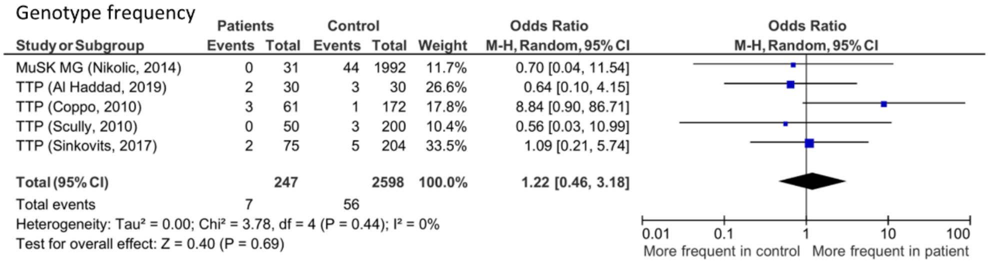

Allele frequency

Study or Subgroup	Patients Events	Patients Total	Control Events	Control Total	Weight	Odds Ratio M-H, Random, 95% CI
CIDP (Piccinelli, 2019) ‡	2	48	0	432	21.8%	46.51 [2.20, 983.35]
CIDP, NF155 (Ogata, 2020)	0	44	6	836	23.1%	1.44 [0.08, 25.89]
MuSK MG (Ehsan, 2015)	1	48	16	400	31.3%	0.51 [0.07, 3.94]
MuSK MG (Nikolic, 2014)	0	62	44	3984	23.9%	0.71 [0.04, 11.63]
**Total (95% CI)**		**202**		**5652**	**100.0%**	**1.87 [0.28, 12.69]**
Total events	3		66			

Heterogeneity: Tau² = 1.96; Chi² = 6.21, df = 3 (P = 0.10); I² = 52%

Test for overall effect: Z = 0.64 (P = 0.52)

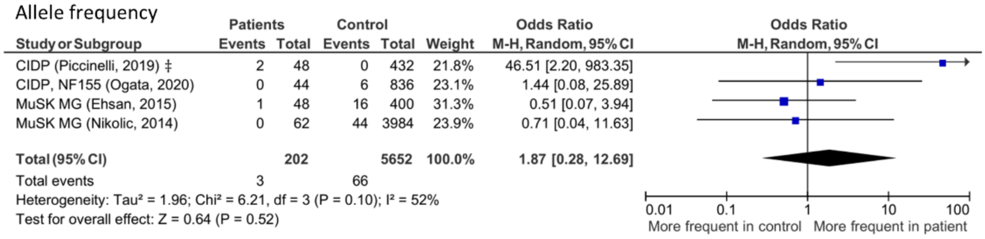

Figure S 35: Allele and genotype frequency of HLA-DRB1*10 in MuSK MG, TTP, CIDP

DRB1*11

Genotype frequency

Study or Subgroup	Patient Events	Patient Total	Control Events	Control Total	Weight	Odds Ratio M-H, Random, 95% CI
MuSK MG (Kanai, 2016)	2	14	5	100	7.4%	3.17 [0.55, 18.16]
MuSK MG (Nikolic, 2014)	7	31	617	1992	14.5%	0.65 [0.28, 1.52]
TTP (Al Haddad, 2019)	23	30	25	30	10.5%	0.66 [0.18, 2.36]
TTP (Coppo, 2010)	38	61	39	172	16.7%	5.63 [3.00, 10.57]
TTP (John, 2011)	26	54	2681	11407	17.5%	3.02 [1.77, 5.16]
TTP (Scully, 2010)	22	50	24	200	15.9%	5.76 [2.85, 11.63]
TTP (Sinkovits, 2017)	42	75	59	204	17.4%	3.13 [1.81, 5.41]
**Total (95% CI)**		**315**		**14105**	**100.0%**	**2.56 [1.41, 4.65]**
Total events	160		3450			

Heterogeneity: Tau² = 0.45; Chi² = 25.33, df = 6 (P = 0.0003); I² = 76%

Test for overall effect: Z = 3.08 (P = 0.002)

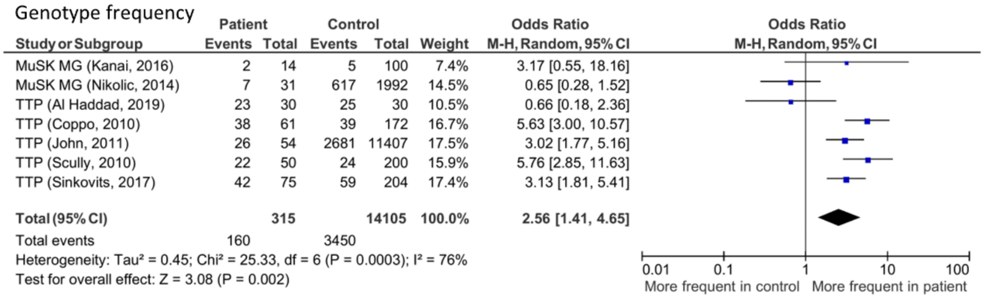

Allele frequency

Study or Subgroup	Patient Events	Patient Total	Control Events	Control Total	Weight	Odds Ratio M-H, Random, 95% CI
CIDP (Piccinelli, 2019) ‡	15	48	111	432	23.1%	1.31 [0.69, 2.51]
CIDP, NF155 (Ogata, 2020)	2	44	23	836	5.9%	1.68 [0.38, 7.38]
MuSK MG (Ehsan, 2015)	5	48	100	400	12.8%	0.35 [0.13, 0.91]
MuSK MG (Nikolic, 2014)	7	62	637	3984	17.2%	0.67 [0.30, 1.48]
TTP (Sakai, 2020)	5	104	38	1046	12.8%	1.34 [0.52, 3.48]
TTP, African patients (Martino, 2016)	6	52	201	1326	15.0%	0.73 [0.31, 1.73]
TTP, Caribbean patients (Martino, 2016)	6	48	32	200	13.2%	0.75 [0.29, 1.91]
**Total (95% CI)**		**406**		**8224**	**100.0%**	**0.85 [0.59, 1.24]**
Total events	46		1142			

Heterogeneity: Tau² = 0.05; Chi² = 7.46, df = 6 (P = 0.28); I² = 20%

Test for overall effect: Z = 0.83 (P = 0.41)

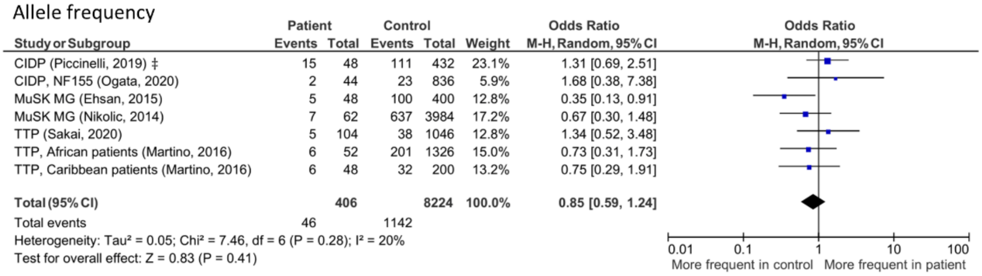

Figure S 36: Allele and genotype frequency of HLA-DRB1*11 in MuSK MG, TTP and CIDP

DRB1*12

Genotype frequency

Study or Subgroup	Patients Events	Patients Total	Control Events	Control Total	Weight	Odds Ratio M-H, Random, 95% CI
MuSK MG (Kanai, 2016)	1	14	7	100	6.9%	1.02 [0.12, 8.99]
MuSK MG (Nikolic, 2014)	2	31	76	1992	15.6%	1.74 [0.41, 7.42]
TTP (Al Haddad, 2019)	3	30	0	30	3.6%	7.76 [0.38, 157.14]
TTP (Coppo, 2010)	2	61	2	172	8.4%	2.88 [0.40, 20.92]
TTP (John, 2011)	2	54	422	11407	16.4%	1.00 [0.24, 4.12]
TTP (Scully, 2010)	5	50	4	200	17.9%	5.44 [1.41, 21.09]
TTP (Sinkovits, 2017)	7	75	9	204	31.2%	2.23 [0.80, 6.22]
**Total (95% CI)**		**315**		**14105**	**100.0%**	**2.24 [1.26, 3.96]**
Total events	22		520			

Heterogeneity: Tau² = 0.00; Chi² = 4.23, df = 6 (P = 0.65); I² = 0%

Test for overall effect: Z = 2.75 (P = 0.006)

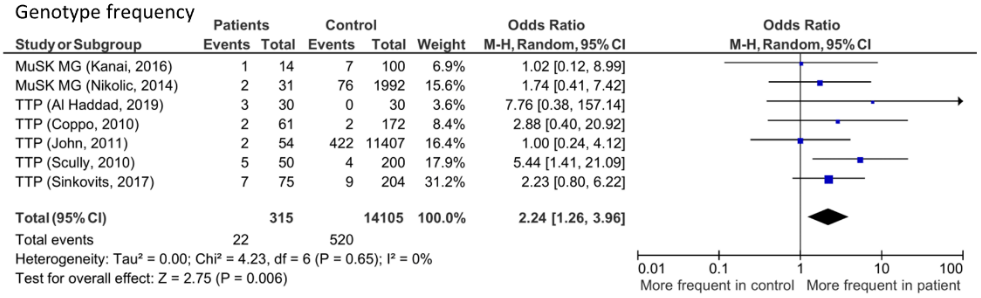

Allele frequency

Study or Subgroup	Patients Events	Patients Total	Control Events	Control Total	Weight	Odds Ratio M-H, Random, 95% CI
CIDP, NF155 (Ogata, 2020)	3	44	48	836	23.8%	1.20 [0.36, 4.02]
MuSK MG (Ehsan, 2015)	1	48	6	400	7.6%	1.40 [0.16, 11.86]
MuSK MG (Nikolic, 2014)	2	62	76	3984	17.0%	1.71 [0.41, 7.14]
TTP (Sakai, 2020)	7	104	48	1046	51.6%	1.50 [0.66, 3.41]
**Total (95% CI)**		**258**		**6266**	**100.0%**	**1.45 [0.80, 2.61]**
Total events	13		178			

Heterogeneity: Tau² = 0.00; Chi² = 0.15, df = 3 (P = 0.98); I² = 0%

Test for overall effect: Z = 1.23 (P = 0.22)

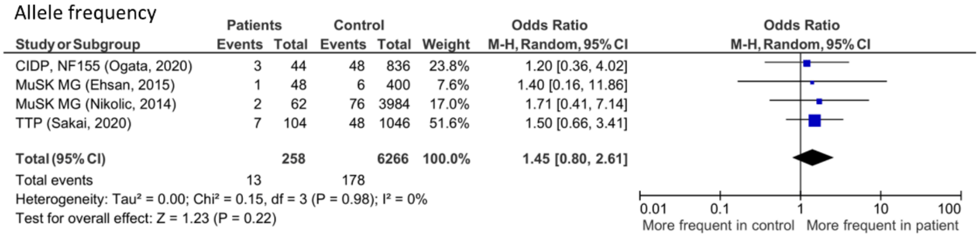

Figure S 37: Allele and genotype frequency of HLA-DRB1*12 in MuSK MG, TTP and CIDP

DRB1*15

Genotype frequency

Study or Subgroup	Patient Events	Patient Total	Control Events	Control Total	Weight	Odds Ratio M-H, Random, 95% CI
CIDP, NF155 (Martinez-Martinez, 2017)	10	13	160	941	11.9%	16.27 [4.43, 59.78]
MuSK MG (Kanai, 2016)	6	14	32	100	13.5%	1.59 [0.51, 4.98]
MuSK MG (Nikolic, 2014)	4	31	371	1992	14.3%	0.65 [0.23, 1.86]
TTP (Al Haddad, 2019)	3	30	0	30	3.7%	7.76 [0.38, 157.14]
TTP (Coppo, 2010)	16	61	33	172	18.7%	1.50 [0.75, 2.97]
TTP (Scully, 2010)	15	50	54	200	18.7%	1.16 [0.59, 2.29]
TTP (Sinkovits, 2017)	21	75	29	204	19.2%	2.35 [1.24, 4.45]
**Total (95% CI)**		**274**		**3639**	**100.0%**	**1.96 [1.04, 3.69]**
Total events	75		679			

Heterogeneity: Tau² = 0.43; Chi² = 18.07, df = 6 (P = 0.006); I² = 67%

Test for overall effect: Z = 2.09 (P = 0.04)

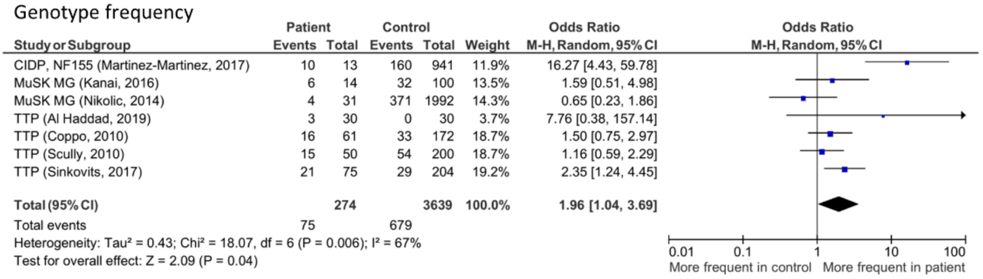

Allele frequency

Study or Subgroup	Patient Events	Patient Total	Control Events	Control Total	Weight	Odds Ratio M-H, Random, 95% CI
CIDP (Piccinelli, 2019) ‡	2	48	24	432	15.8%	0.74 [0.17, 3.23]
CIDP, NF155 (Ogata, 2020)	23	44	137	836	22.2%	5.59 [3.01, 10.38]
MuSK MG (Ehsan, 2015)	6	48	48	400	20.2%	1.05 [0.42, 2.59]
MuSK MG (Nikolic, 2014)	4	62	392	3984	19.3%	0.63 [0.23, 1.75]
TTP (Sakai, 2020)	16	104	212	1046	22.6%	0.72 [0.41, 1.24]
**Total (95% CI)**		**306**		**6698**	**100.0%**	**1.20 [0.45, 3.21]**
Total events	51		813			

Heterogeneity: Tau² = 1.05; Chi² = 28.74, df = 4 (P < 0.00001); I² = 86%

Test for overall effect: Z = 0.36 (P = 0.72)

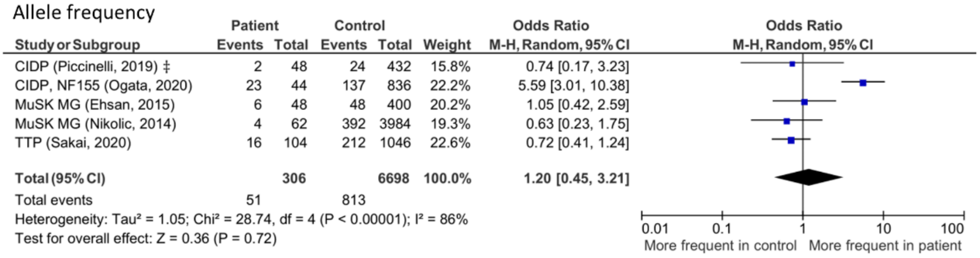

Figure S 38: Allele and genotype frequency of HLA-DRB1*15 in MuSK MG, TTP and CIDP

DRB1*16

Genotype frequency

Study or Subgroup	Patients Events	Patients Total	Control Events	Control Total	Weight	Odds Ratio M-H, Random, 95% CI
MuSK MG (Alahgholi-Hajibehzad, 2013)	18	48	27	250	16.4%	4.96 [2.44, 10.06]
MuSK MG (Kanai, 2016)	2	14	2	100	7.1%	8.17 [1.05, 63.41]
MuSK MG (Nikolic, 2014)	17	31	406	1992	16.4%	4.74 [2.32, 9.70]
MuSK MG (Niks, 2006)	2	23	43	2440	10.3%	5.31 [1.21, 23.36]
TTP (Al Haddad, 2019)	10	30	8	30	13.0%	1.38 [0.45, 4.17]
TTP (Coppo, 2010)	3	61	11	172	11.5%	0.76 [0.20, 2.81]
TTP (Scully, 2010)	3	50	5	200	10.4%	2.49 [0.57, 10.79]
TTP (Sinkovits, 2017)	7	75	26	204	15.0%	0.70 [0.29, 1.70]
**Total (95% CI)**		**332**		**5388**	**100.0%**	**2.43 [1.23, 4.80]**
Total events	62		528			

Heterogeneity: Tau² = 0.60; Chi² = 21.71, df = 7 (P = 0.003); I² = 68%

Test for overall effect: Z = 2.57 (P = 0.01)

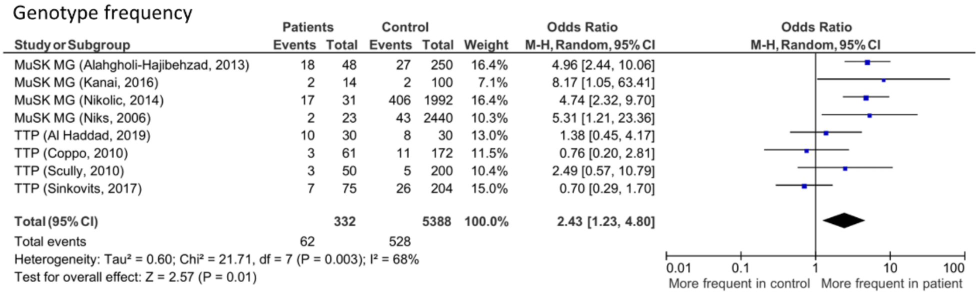

Allele frequency

Study or Subgroup	Patients Events	Patients Total	Control Events	Control Total	Weight	Odds Ratio M-H, Random, 95% CI
CIDP (Piccinelli, 2019) ‡	2	48	19	432	22.2%	0.95 [0.21, 4.19]
CIDP, NF155 (Ogata, 2020)	0	44	2	836	6.2%	3.75 [0.18, 79.30]
MuSK MG (Nikolic, 2014)	18	62	433	3984	71.5%	3.35 [1.92, 5.86]
**Total (95% CI)**		**154**		**5252**	**100.0%**	**2.55 [1.16, 5.58]**
Total events	20		454			

Heterogeneity: Tau² = 0.14; Chi² = 2.54, df = 2 (P = 0.28); I² = 21%

Test for overall effect: Z = 2.34 (P = 0.02)

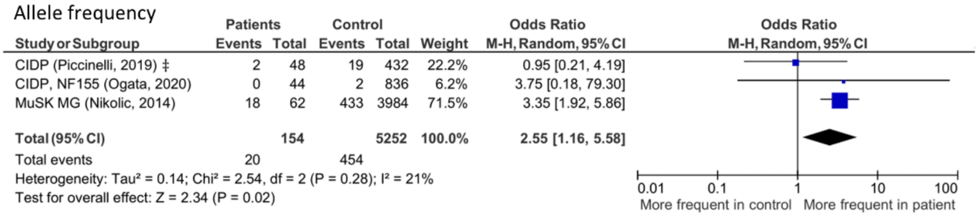

Figure S 39: Allele and genotype frequency of HLA-DRB1*16 in MuSK MG, TTP and CIDP

DQB1*02

Genotype frequency

Study or Subgroup	Patients Events	Patients Total	Control Events	Control Total	Weight	Odds Ratio M-H, Random, 95% CI
MuSK MG (Nikolic, 2014)	12	31	48	159	20.6%	1.46 [0.66, 3.24]
TTP (Al Haddad, 2019)	14	30	2	30	15.8%	12.25 [2.46, 60.91]
TTP (Coppo, 2010)	19	60	68	172	21.4%	0.71 [0.38, 1.32]
TTP (John, 2011)	26	50	19	174	20.9%	8.84 [4.25, 18.36]
TTP (Scully, 2010)	16	50	86	200	21.3%	0.62 [0.32, 1.20]
**Total (95% CI)**		**221**		**735**	**100.0%**	**2.13 [0.68, 6.64]**
Total events	87		223			

Heterogeneity: Tau² = 1.47; Chi² = 41.58, df = 4 (P < 0.00001); I² = 90%
Test for overall effect: Z = 1.30 (P = 0.19)

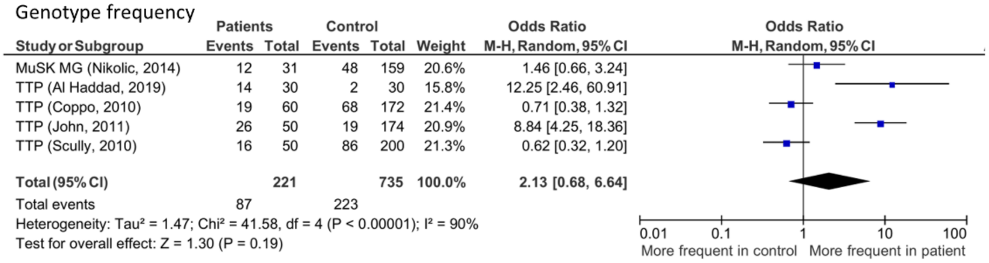

Allele frequency

Study or Subgroup	Patients Events	Patients Total	Control Events	Control Total	Weight	Odds Ratio M-H, Random, 95% CI
CIDP (Piccinelli, 2019) ‡	8	48	73	432	29.5%	0.98 [0.44, 2.19]
CIDP, NF155 (Ogata, 2020)	0	44	1	836	1.8%	6.26 [0.25, 155.82]
MuSK MG (Ehsan, 2015)	8	48	76	400	29.6%	0.85 [0.38, 1.90]
MuSK MG (Nikolic, 2014)	12	62	54	318	39.1%	1.17 [0.59, 2.35]
**Total (95% CI)**		**202**		**1986**	**100.0%**	**1.04 [0.68, 1.61]**
Total events	28		204			

Heterogeneity: Tau² = 0.00; Chi² = 1.58, df = 3 (P = 0.67); I² = 0%
Test for overall effect: Z = 0.20 (P = 0.84)

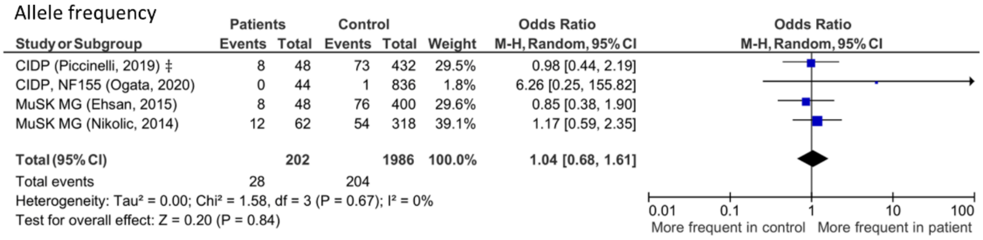

Figure S 40: Allele and genotype frequency of HLA-DQB1*02 in MuSK MG, TTP and CIDP

DQB1*03

Genotype frequency

Study or Subgroup	Patients Events	Patients Total	Control Events	Control Total	Weight	Odds Ratio M-H, Random, 95% CI
MuSK MG (Kanai, 2016)	9	14	51	100	14.9%	1.73 [0.54, 5.52]
MuSK MG (Nikolic, 2014)	9	21	87	159	17.1%	0.62 [0.25, 1.56]
TTP (Al Haddad, 2019)	28	30	26	30	10.2%	2.15 [0.36, 12.76]
TTP (Coppo, 2010)	46	60	92	172	19.4%	2.86 [1.46, 5.58]
TTP (John, 2011)	29	47	29	174	19.0%	8.06 [3.96, 16.39]
TTP (Scully, 2010)	36	50	129	200	19.3%	1.42 [0.72, 2.80]
**Total (95% CI)**		**222**		**835**	**100.0%**	**2.11 [0.98, 4.53]**
Total events	157		414			

Heterogeneity: Tau² = 0.67; Chi² = 22.37, df = 5 (P = 0.0004); I² = 78%
Test for overall effect: Z = 1.92 (P = 0.06)

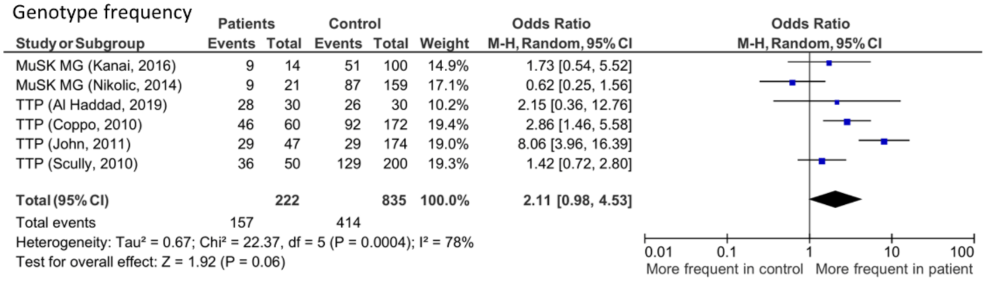

Allele frequency

Study or Subgroup	Patients Events	Patients Total	Control Events	Control Total	Weight	Odds Ratio M-H, Random, 95% CI
CIDP (Piccinelli, 2019) ‡	20	48	196	432	14.8%	0.86 [0.47, 1.57]
CIDP, NF155 (Ogata, 2020)	13	33	309	836	13.8%	1.11 [0.54, 2.26]
MuSK MG (Ehsan, 2015)	8	48	54	400	12.8%	1.28 [0.57, 2.88]
MuSK MG (Nikolic, 2014)	11	62	106	318	14.0%	0.43 [0.22, 0.86]
TTP (Sakai, 2020)	37	104	378	1046	16.4%	0.98 [0.64, 1.49]
TTP, African patients (Martino, 2016)	17	52	82	874	14.6%	4.69 [2.52, 8.74]
TTP, Caribbean patients (Martino, 2016)	12	48	50	200	13.6%	1.00 [0.48, 2.07]
**Total (95% CI)**		**395**		**4106**	**100.0%**	**1.14 [0.67, 1.93]**
Total events	118		1175			

Heterogeneity: Tau² = 0.40; Chi² = 29.39, df = 6 (P < 0.0001); I² = 80%
Test for overall effect: Z = 0.47 (P = 0.64)

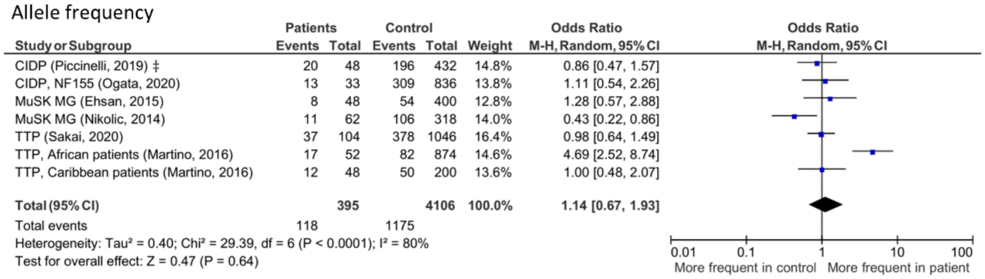

Figure S 41: Allele and genotype frequency of HLA-DQB1*03 in MuSK MG, TTP and CIDP

DQB1*04

Genotype frequency

Study or Subgroup	Patients Events	Patients Total	Control Events	Control Total	Weight	Odds Ratio M-H, Random, 95% CI
MuSK MG (Kanai, 2016)	2	14	37	100	18.1%	0.28 [0.06, 1.34]
MuSK MG (Nikolic, 2014)	3	31	28	159	21.1%	0.50 [0.14, 1.76]
TTP (Al Haddad, 2019)	0	30	1	30	7.5%	0.32 [0.01, 8.24]
TTP (Coppo, 2010)	3	60	7	172	19.8%	1.24 [0.31, 4.96]
TTP (John, 2011)	1	54	3	174	12.1%	1.08 [0.11, 10.56]
TTP (Scully, 2010)	6	50	5	200	21.4%	5.32 [1.55, 18.21]
**Total (95% CI)**		**239**		**835**	**100.0%**	**0.95 [0.35, 2.63]**
Total events	15		81			

Heterogeneity: Tau² = 0.86; Chi² = 11.54, df = 5 (P = 0.04); I² = 57%

Test for overall effect: Z = 0.09 (P = 0.93)

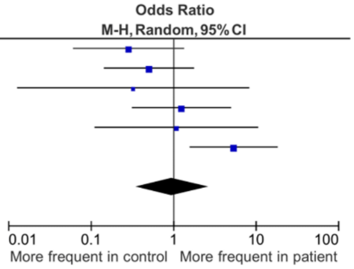

Allele frequency

Study or Subgroup	Patients Events	Patients Total	Control Events	Control Total	Weight	Odds Ratio M-H, Random, 95% CI
CIDP (Piccinelli, 2019) ‡	0	48	16	432	12.9%	0.26 [0.02, 4.41]
CIDP, NF155 (Ogata, 2020)	1	44	148	836	19.9%	0.11 [0.01, 0.79]
MuSK MG (Nikolic, 2014)	3	62	7	318	27.6%	2.26 [0.57, 8.99]
TTP (Sakai, 2020)	19	104	174	1046	39.6%	1.12 [0.66, 1.89]
**Total (95% CI)**		**258**		**2632**	**100.0%**	**0.71 [0.21, 2.38]**
Total events	23		345			

Heterogeneity: Tau² = 0.90; Chi² = 8.35, df = 3 (P = 0.04); I² = 64%

Test for overall effect: Z = 0.56 (P = 0.58)

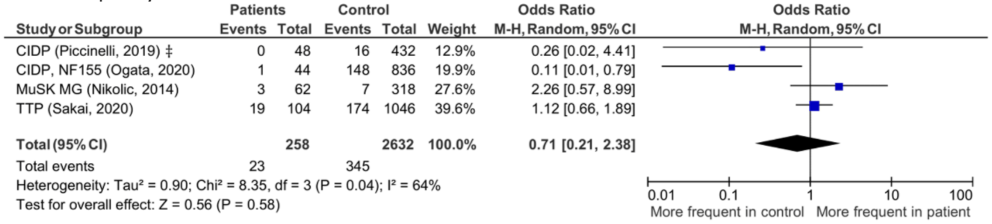

Figure S 42: Allele and genotype frequency of HLA-DQB1*04 in MuSK MG, TTP and CIDP

DQB1*06

Genotype frequency

Study or Subgroup	Patients Events	Patients Total	Control Events	Control Total	Weight	Odds Ratio M-H, Random, 95% CI
MuSK MG (Kanai, 2016)	6	14	57	100	10.3%	0.57 [0.18, 1.75]
MuSK MG (Nikolic, 2014)	4	31	43	159	10.6%	0.40 [0.13, 1.21]
MuSK MG (Niks, 2006)	5	23	453	2440	12.4%	1.22 [0.45, 3.30]
TTP (Al Haddad, 2019)	2	30	3	30	4.5%	0.64 [0.10, 4.15]
TTP (Coppo, 2010)	22	60	74	172	22.3%	0.77 [0.42, 1.40]
TTP (John, 2011)	15	52	28	174	18.6%	2.11 [1.03, 4.36]
TTP (Scully, 2010)	19	50	92	200	21.3%	0.72 [0.38, 1.36]
**Total (95% CI)**		**260**		**3275**	**100.0%**	**0.87 [0.57, 1.32]**
Total events	73		750			

Heterogeneity: Tau² = 0.11; Chi² = 9.31, df = 6 (P = 0.16); I² = 36%

Test for overall effect: Z = 0.67 (P = 0.51)

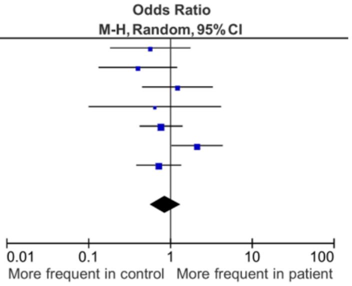

Allele frequency

Study or Subgroup	Patients Events	Patients Total	Control Events	Control Total	Weight	Odds Ratio M-H, Random, 95% CI
CIDP (Piccinelli, 2019) ‡	8	48	63	432	19.8%	1.17 [0.52, 2.62]
CIDP, NF155 (Ogata, 2020)	28	44	168	836	21.0%	6.96 [3.68, 13.16]
MuSK MG (Ehsan, 2015)	6	48	70	400	19.1%	0.67 [0.28, 1.65]
MuSK MG (Nikolic, 2014)	4	62	46	318	17.8%	0.41 [0.14, 1.18]
TTP (Sakai, 2020)	37	104	330	1046	22.3%	1.20 [0.79, 1.83]
**Total (95% CI)**		**306**		**3032**	**100.0%**	**1.28 [0.51, 3.17]**
Total events	83		677			

Heterogeneity: Tau² = 0.92; Chi² = 32.80, df = 4 (P < 0.00001); I² = 88%

Test for overall effect: Z = 0.53 (P = 0.60)

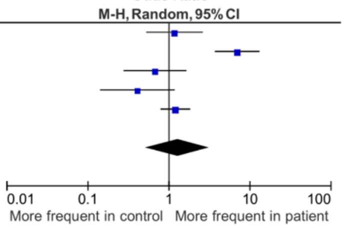

Figure S 43: Allele and genotype frequency of HLA-DQB1*06 in MuSK MG, TTP and CIDP

DRB1*16-DQB1*05

n

Study or Subgroup	Patients Events	Patients Total	Control Events	Control Total	Weight	Odds Ratio M-H, Random, 95% CI
MuSK MG (Alahgholi-Hajibehzad, 2013)	16	48	27	250	40.4%	4.13 [2.01, 8.49]
MuSK MG (Kanai, 2016)	2	14	2	100	21.9%	8.17 [1.05, 63.41]
TTP (Sinkovits, 2017)	7	75	18	162	37.6%	0.82 [0.33, 2.07]
**Total (95% CI)**		**137**		**512**	**100.0%**	**2.61 [0.69, 9.87]**
Total events	25		47			

Heterogeneity: Tau² = 1.00; Chi² = 8.93, df = 2 (P = 0.01); I² = 78%

Test for overall effect: Z = 1.42 (P = 0.16)

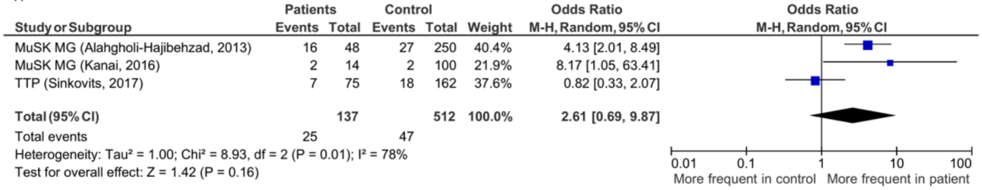

2n

Study or Subgroup	Patients Events	Patients Total	Control Events	Control Total	Weight	Odds Ratio M-H, Random, 95% CI
MuSK MG (Ehsan, 2015)	11	48	26	400	36.8%	4.28 [1.96, 9.35]
MuSK MG (Nikolic, 2014)	18	62	39	318	54.4%	2.93 [1.54, 5.56]
MuSK MG (Niks, 2006)	2	46	7	642	8.8%	4.12 [0.83, 20.44]
**Total (95% CI)**		**156**		**1360**	**100.0%**	**3.47 [2.16, 5.57]**
Total events	31		72			

Heterogeneity: Tau² = 0.00; Chi² = 0.59, df = 2 (P = 0.74); I² = 0%

Test for overall effect: Z = 5.14 (P < 0.00001)

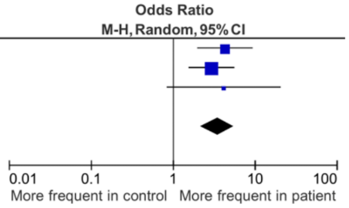

Figure S 44: Allele and genotype frequency of HLA-DRB1*16-DQB1*05 in MuSK MG, TTP, CIDP

DRB1*15-DQB1*06

n

Study or Subgroup	Patients Events	Patients Total	Control Events	Control Total	Weight	Odds Ratio M-H, Random, 95% CI
CIDP, NF155 (Martinez-Martinez, 2017)	8	13	5	35	37.8%	9.60 [2.22, 41.52]
TTP (Sinkovits, 2017)	19	75	19	162	62.2%	2.55 [1.26, 5.18]
**Total (95% CI)**		**88**		**197**	**100.0%**	**4.21 [1.20, 14.83]**
Total events	27		24			

Heterogeneity: Tau² = 0.53; Chi² = 2.55, df = 1 (P = 0.11); I² = 61%
Test for overall effect: Z = 2.24 (P = 0.03)

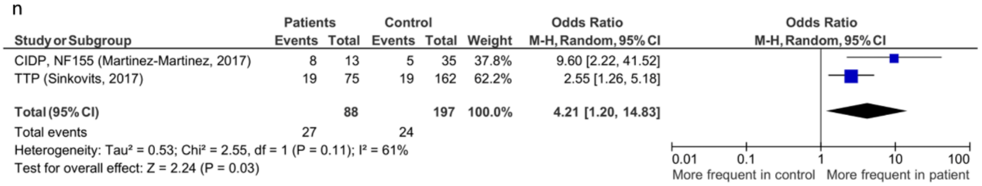

2n

Study or Subgroup	Patients Events	Patients Total	Control Events	Control Total	Weight	Odds Ratio M-H, Random, 95% CI
CIDP (Piccinelli, 2019) ‡	1	48	20	432	17.1%	0.44 [0.06, 3.34]
CIDP, NF155 (Ogata, 2020)	23	44	135	836	30.2%	5.69 [3.06, 10.57]
MuSK MG (Ehsan, 2015)	5	48	44	400	27.0%	0.94 [0.35, 2.50]
MuSK MG (Nikolic, 2014)	4	62	19	318	25.7%	1.09 [0.36, 3.31]
**Total (95% CI)**		**202**		**1986**	**100.0%**	**1.47 [0.44, 4.98]**
Total events	33		218			

Heterogeneity: Tau² = 1.18; Chi² = 16.05, df = 3 (P = 0.001); I² = 81%
Test for overall effect: Z = 0.62 (P = 0.53)

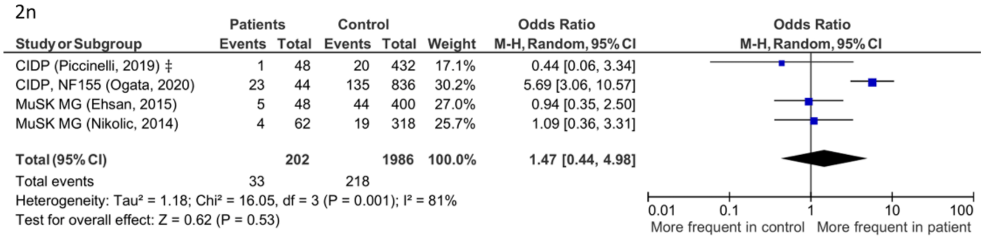

Figure S 45: Allele and genotype frequency of HLA-DRB1*15-DQB1*06 in MuSK MG, TTP, CIDP

DRB1*01

Genotype frequency

Study or Subgroup	Patients Events	Patients Total	Control Events	Control Total	Weight	Odds Ratio M-H, Random, 95% CI
Fogo selvagem (Moraes, 1991)	22	37	10	49	8.7%	5.72 [2.20, 14.87]
Fogo selvagem (Pavoni, 2003)	78	128	70	402	9.3%	7.40 [4.77, 11.47]
Pemphigus (Lee, 1998)**	19	30	12	100	8.7%	12.67 [4.87, 32.97]
Pemphigus (Zhang, 2019)**	9	327	26	501	9.0%	0.52 [0.24, 1.12]
PF (Martel, 2002)	7	31	16	84	8.6%	1.24 [0.45, 3.38]
PV (Dere, 2020)	1	30	1	30	5.2%	1.00 [0.06, 16.76]
PV (Gil, 2017)	2	102	61	594	7.9%	0.17 [0.04, 0.73]
PV (Gonzalez-Escribano, 1998)	1	26	43	200	6.7%	0.15 [0.02, 1.11]
PV (Harfouch, 2014)	5	91	33	270	8.7%	0.42 [0.16, 1.10]
PV (Khan, 2015)	0	28	0	150		Not estimable
PV (Lombardi, 1996)	2	33	13	102	7.6%	0.44 [0.09, 2.07]
PV (Priyadarshini, 2018)	0	50	3	50	4.9%	0.13 [0.01, 2.67]
PV (Thomas, 1998)	1	26	43	200	6.7%	0.15 [0.02, 1.11]
PV (Tunca, 2010)	3	25	16	113	8.1%	0.83 [0.22, 3.09]
**Total (95% CI)**		**964**		**2845**	**100.0%**	**0.88 [0.34, 2.28]**
Total events	150		347			

Heterogeneity: Tau² = 2.47; Chi² = 113.47, df = 12 (P < 0.00001); I² = 89%
Test for overall effect: Z = 0.26 (P = 0.79)

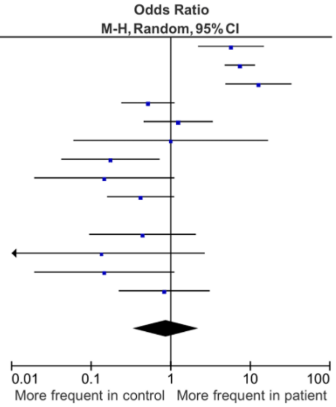

Allele frequency

Study or Subgroup	Patients Events	Patients Total	Control Events	Control Total	Weight	Odds Ratio M-H, Random, 95% CI
Pemphigus (Brochado, 2016) **	69	336	263	3184	11.2%	2.87 [2.14, 3.85]
Pemphigus (Torzecka, 2003)**	15	106	31	304	10.1%	1.45 [0.75, 2.81]
Pemphigus (Zhang, 2019)**	9	654	31	1002	9.8%	0.44 [0.21, 0.92]
PF, Indo-Asian (Saha, 2019)	8	50	24	200	9.4%	1.40 [0.59, 3.33]
PF, White British (Saha, 2019)	0	20	4	118	3.2%	0.62 [0.03, 11.97]
PV (Párnická, 2013)	6	86	23	226	9.1%	0.66 [0.26, 1.69]
PV (Rangel-Gamboa, 2015) ‡	13	86	10	198	9.4%	3.35 [1.41, 7.97]
PV (Shams, 2008)	2	104	24	360	7.1%	0.27 [0.06, 1.18]
PV (Zivanovic, 2016)	7	144	410	3984	9.8%	0.45 [0.21, 0.96]
PV, Egypt (Haase, 2015)*	1	94	7	146	5.0%	0.21 [0.03, 1.76]
PV, Europe (Delgado, 1997)	0	38	1	26	2.8%	0.22 [0.01, 5.63]
PV, German (Haase, 2015)*	1	92	15	148	5.2%	0.10 [0.01, 0.75]
PV, Pakistan (Delgado, 1997) †	3	38	54	496	8.0%	0.70 [0.21, 2.36]
**Total (95% CI)**		**1848**		**10392**	**100.0%**	**0.76 [0.41, 1.42]**
Total events	134		897			

Heterogeneity: Tau² = 0.89; Chi² = 68.27, df = 12 (P < 0.00001); I² = 82%
Test for overall effect: Z = 0.85 (P = 0.39)

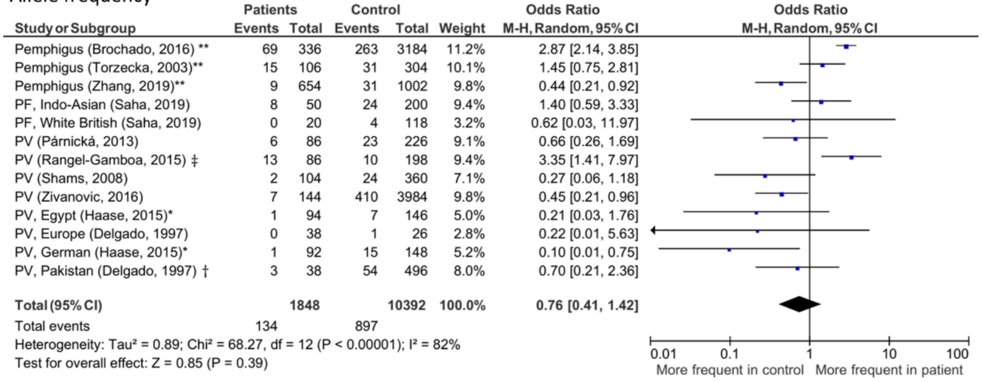

Figure S 46: Allele and genotype frequency of HLA-DRB1*01 in pemphigus

DRB1*03

Genotype frequency

Study or Subgroup	Patients Events	Patients Total	Control Events	Control Total	Weight	Odds Ratio M-H, Random, 95% CI
Fogo selvagem (Pavoni, 2003)	14	128	71	402	28.1%	0.57 [0.31, 1.06]
Pemphigus (Lee, 1998)**	0	30	5	100	1.2%	0.28 [0.02, 5.30]
Pemphigus (Zhang, 2019)**	4	327	39	501	9.7%	0.15 [0.05, 0.41]
PF (Martel, 2002)	2	31	18	84	4.5%	0.25 [0.06, 1.16]
PV (Dere, 2020)	1	30	8	30	2.3%	0.09 [0.01, 0.82]
PV (Gil, 2017)	4	102	66	594	9.9%	0.33 [0.12, 0.92]
PV (Glorio, 1999)	2	30	42	199	4.8%	0.27 [0.06, 1.17]
PV (Glorio, 2002)	3	47	42	199	7.1%	0.25 [0.08, 0.86]
PV (Gonzalez-Escribano, 1998)	1	26	42	200	2.6%	0.15 [0.02, 1.14]
PV (Harfouch, 2014)	2	91	40	270	5.1%	0.13 [0.03, 0.55]
PV (Khan, 2015)	4	28	31	150	8.2%	0.64 [0.21, 1.98]
PV (Lombardi, 1996)	4	33	15	102	7.5%	0.80 [0.25, 2.60]
PV (Priyadarshini, 2018)	1	50	2	50	1.8%	0.49 [0.04, 5.58]
PV (Thomas, 1998)	1	26	42	200	2.6%	0.15 [0.02, 1.14]
PV (Tunca, 2010)	2	25	26	113	4.6%	0.29 [0.06, 1.32]
**Total (95% CI)**		**1004**		**3194**	**100.0%**	**0.34 [0.25, 0.47]**
Total events	45		489			

Heterogeneity: Tau² = 0.00; Chi² = 13.92, df = 14 (P = 0.46); I² = 0%
Test for overall effect: Z = 6.47 (P < 0.00001)

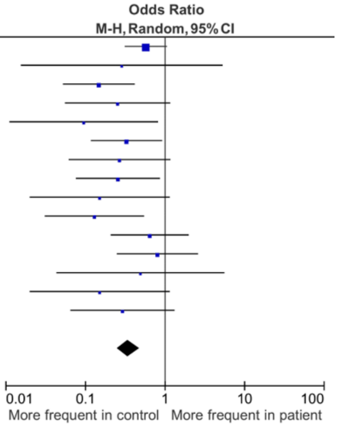

Allele frequency

Study or Subgroup	Patients Events	Patients Total	Control Events	Control Total	Weight	Odds Ratio M-H, Random, 95% CI
Pemphigus (Torzecka, 2003)**	11	106	31	304	10.3%	1.02 [0.49, 2.11]
Pemphigus (Zhang, 2019)**	4	654	39	1002	9.2%	0.15 [0.05, 0.43]
PF (Abida, 2009)	46	180	79	540	11.1%	2.00 [1.33, 3.02]
PV (Párnická, 2013)	4	86	18	226	9.0%	0.56 [0.19, 1.72]
PV (Shams, 2008)	2	104	32	360	7.8%	0.20 [0.05, 0.85]
PV (Zivanovic, 2016)	6	144	433	3984	10.0%	0.36 [0.16, 0.81]
PV, Egypt (Haase, 2015)*	7	94	18	146	9.7%	0.57 [0.23, 1.43]
PV, German (Haase, 2015)*	4	92	21	148	9.0%	0.27 [0.09, 0.83]
PV, Indo-Asians (Saha, 2010)*	1	114	17	118	5.9%	0.05 [0.01, 0.40]
PV, Italians (Carcassi, 1996)	0	32	59	568	4.1%	0.13 [0.01, 2.18]
PV, Sardinians (Carcassi, 1996)	0	32	45	182	4.0%	0.05 [0.00, 0.77]
PV, White Europeans (Saha, 2010)*	8	192	29	200	10.0%	0.26 [0.11, 0.58]
**Total (95% CI)**		**1830**		**7778**	**100.0%**	**0.35 [0.17, 0.70]**
Total events	93		821			

Heterogeneity: Tau² = 1.11; Chi² = 64.33, df = 11 (P < 0.00001); I² = 83%
Test for overall effect: Z = 2.96 (P = 0.003)

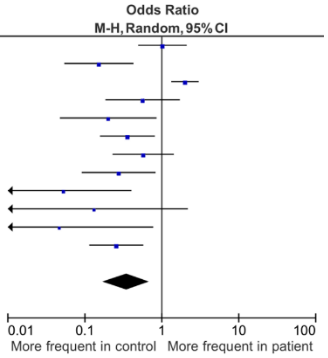

Figure S 47: Allele and genotype frequency of HLA-DRB1*03 in pemphigus

DRB1*04

Genotype frequency

Study or Subgroup	Patients Events	Patients Total	Control Events	Control Total	Weight	Odds Ratio M-H, Random, 95% CI
Fogo selvagem (Cerna, 1993)	6	10	10	74	2.9%	9.60 [2.30, 40.11]
Fogo selvagem (Pavoni, 2003)	49	128	76	402	7.2%	2.66 [1.72, 4.11]
Pemphigus (Birol, 2002) ‡	25	33	29	100	4.8%	7.65 [3.09, 18.93]
Pemphigus (Koc, 2012) ‡	39	60	11	60	5.1%	8.27 [3.56, 19.20]
Pemphigus (Lee, 1998)**	19	30	46	100	5.1%	2.03 [0.88, 4.70]
Pemphigus (Miyagawa, 1997)**	7	16	35	525	4.1%	10.89 [3.83, 30.98]
Pemphigus (Zhang, 2019)**	114	327	96	501	7.7%	2.26 [1.64, 3.10]
PF (Martel, 2002)	19	31	27	84	5.0%	3.34 [1.42, 7.86]
PV (Dere, 2020)	25	30	9	30	3.4%	11.67 [3.38, 40.22]
PV (Gil, 2017)	29	102	66	594	6.9%	3.18 [1.93, 5.24]
PV (Glorio, 1999)	16	30	47	199	5.3%	3.70 [1.68, 8.13]
PV (Glorio, 2002)	28	47	45	199	5.9%	5.04 [2.58, 9.86]
PV (Gonzalez-Escribano, 1998)	23	26	45	200	3.4%	26.41 [7.58, 91.99]
PV (Harfouch, 2014)	61	91	71	270	6.8%	5.70 [3.41, 9.53]
PV (Khan, 2015)	14	28	31	150	5.1%	3.84 [1.66, 8.89]
PV (Lombardi, 1996)	11	33	15	102	4.7%	2.90 [1.17, 7.19]
PV (Priyadarshini, 2018)	13	50	8	50	4.4%	1.84 [0.69, 4.94]
PV (Thomas, 1998)	23	26	45	200	3.4%	26.41 [7.58, 91.99]
PV (Tunca, 2010)	17	25	35	113	4.6%	4.74 [1.87, 12.00]
PV (Yamashina, 1998)	7	17	35	525	4.2%	9.80 [3.52, 27.31]
**Total (95% CI)**		**1140**		**4478**	**100.0%**	**4.86 [3.61, 6.54]**
Total events	545		782			

Heterogeneity: Tau² = 0.27; Chi² = 57.38, df = 19 (P < 0.00001); I² = 67%

Test for overall effect: Z = 10.42 (P < 0.00001)

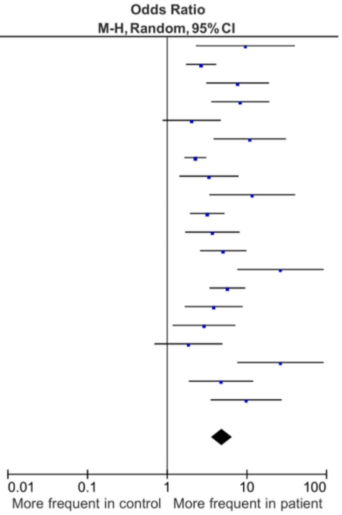

Allele frequency

Study or Subgroup	Patients Events	Patients Total	Control Events	Control Total	Weight	Odds Ratio M-H, Random, 95% CI
Fogo selvagem (de Sena Nogueira Maehara, 2017)	33	84	87	956	6.3%	6.46 [3.96, 10.55]
Pemphigus (Brochado, 2016) **	46	336	65	3184	6.7%	7.61 [5.12, 11.31]
Pemphigus (Torzecka, 2003)**	32	106	38	304	6.1%	3.03 [1.77, 5.17]
Pemphigus (Zhang, 2019)**	127	654	111	1002	7.2%	1.93 [1.47, 2.55]
PF (Abida, 2009)	57	180	79	540	6.7%	2.70 [1.82, 4.01]
PF (de Sena Nogueira Maehara, 2017)	13	34	133	894	5.2%	3.54 [1.73, 7.25]
PF, Indo-Asian (Saha, 2019)	2	20	7	118	2.2%	1.76 [0.34, 9.16]
PF, White British (Saha, 2019)	22	50	29	200	5.4%	4.63 [2.34, 9.17]
PV (Párnická, 2013)	32	86	21	226	5.6%	5.78 [3.09, 10.83]
PV (Rangel-Gamboa, 2015) ‡	27	86	47	198	5.9%	1.47 [0.84, 2.58]
PV (Shams, 2008)	38	104	33	360	6.1%	5.71 [3.34, 9.75]
PV (Zivanovic, 2016)	46	144	358	3984	6.8%	4.75 [3.29, 6.86]
PV, Egypt (Haase, 2015)*	42	94	27	146	5.8%	3.56 [1.99, 6.38]
PV, Europe (Delgado, 1997)	4	38	0	26	0.8%	6.91 [0.36, 134.11]
PV, German (Haase, 2015)*	35	92	26	148	5.8%	2.88 [1.59, 5.23]
PV, Indo-Asians (Saha, 2010)*	23	114	8	118	4.6%	3.48 [1.48, 8.14]
PV, Italians (Carcassi, 1996)	7	32	8	568	3.6%	19.60 [6.59, 58.33]
PV, Sardinians (Carcassi, 1996)	13	32	3	182	2.9%	40.82 [10.67, 156.15]
PV, White Europeans (Saha, 2010)*	71	192	29	200	6.3%	3.46 [2.12, 5.65]
**Total (95% CI)**		**2478**		**13354**	**100.0%**	**4.18 [3.14, 5.56]**
Total events	670		1109			

Heterogeneity: Tau² = 0.28; Chi² = 82.97, df = 18 (P < 0.00001); I² = 78%

Test for overall effect: Z = 9.80 (P < 0.00001)

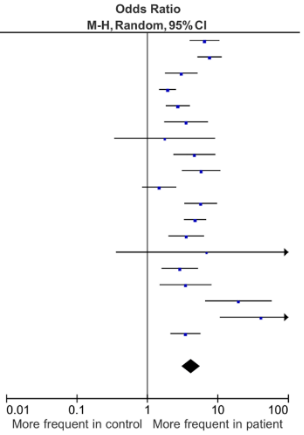

Figure S 48: Allele and genotype frequency of HLA-DRB1*04 in pemphigus

DRB1*07

Genotype frequency

Study or Subgroup	Patients Events	Patients Total	Control Events	Control Total	Weight	Odds Ratio M-H, Random, 95% CI
Fogo selvagem (Pavoni, 2003)	3	128	83	402	7.7%	0.09 [0.03, 0.30]
Pemphigus (Lee, 1998)**	0	30	12	100	2.0%	0.12 [0.01, 2.02]
Pemphigus (Zhang, 2019)**	17	327	26	501	13.1%	1.00 [0.53, 1.88]
PF (Martel, 2002)	5	31	24	84	8.5%	0.48 [0.17, 1.40]
PV (Dere, 2020)	1	30	5	30	3.1%	0.17 [0.02, 1.58]
PV (Gil, 2017)	8	102	80	594	11.5%	0.55 [0.26, 1.17]
PV (Gonzalez-Escribano, 1998)	4	26	72	200	8.2%	0.32 [0.11, 0.97]
PV (Harfouch, 2014)	6	91	55	270	10.3%	0.28 [0.11, 0.66]
PV (Khan, 2015)	4	28	46	150	8.1%	0.38 [0.12, 1.15]
PV (Lombardi, 1996)	1	33	29	102	3.5%	0.08 [0.01, 0.60]
PV (Priyadarshini, 2018)	8	50	14	50	9.3%	0.49 [0.18, 1.30]
PV (Thomas, 1998)	4	26	72	200	8.2%	0.32 [0.11, 0.97]
PV (Tunca, 2010)	3	25	14	113	6.6%	0.96 [0.26, 3.65]
**Total (95% CI)**		**927**		**2796**	**100.0%**	**0.38 [0.25, 0.58]**
Total events	64		532			

Heterogeneity: Tau² = 0.26; Chi² = 22.41, df = 12 (P = 0.03); I² = 46%

Test for overall effect: Z = 4.45 (P < 0.00001)

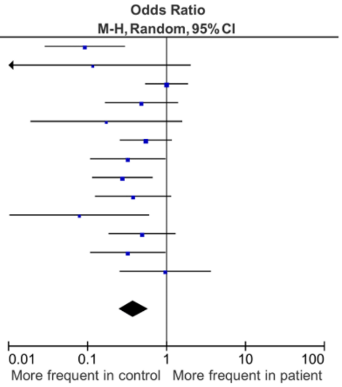

Allele frequency

Study or Subgroup	Patients Events	Patients Total	Control Events	Control Total	Weight	Odds Ratio M-H, Random, 95% CI
Fogo selvagem (de Sena Nogueira Maehara, 2017)	2	84	128	956	4.2%	0.16 [0.04, 0.65]
Pemphigus (Brochado, 2016) **	11	336	342	3184	13.2%	0.28 [0.15, 0.52]
Pemphigus (Torzecka, 2003)**	4	106	44	304	6.8%	0.23 [0.08, 0.66]
Pemphigus (Zhang, 2019)**	17	654	26	1002	13.1%	1.00 [0.54, 1.86]
PV (Párnická, 2013)	4	86	33	226	6.6%	0.29 [0.10, 0.83]
PV (Rangel-Gamboa, 2015) ‡	4	86	22	198	6.3%	0.39 [0.13, 1.17]
PV (Shams, 2008)	2	104	26	360	4.0%	0.25 [0.06, 1.08]
PV (Zivanovic, 2016)	7	144	284	3984	10.3%	0.67 [0.31, 1.44]
PV, Egypt (Haase, 2015)*	4	94	17	146	6.1%	0.34 [0.11, 1.04]
PV, Europe (Delgado, 1997)	1	38	1	26	1.2%	0.68 [0.04, 11.31]
PV, German (Haase, 2015)*	7	92	13	148	7.7%	0.86 [0.33, 2.23]
PV, Indo-Asians (Saha, 2010)*	8	114	14	118	8.3%	0.56 [0.23, 1.39]
PV, White Europeans (Saha, 2010)*	14	192	29	200	12.0%	0.46 [0.24, 0.91]
**Total (95% CI)**		**2130**		**10852**	**100.0%**	**0.45 [0.32, 0.61]**
Total events	85		979			

Heterogeneity: Tau² = 0.10; Chi² = 17.55, df = 12 (P = 0.13); I² = 32%

Test for overall effect: Z = 4.99 (P < 0.00001)

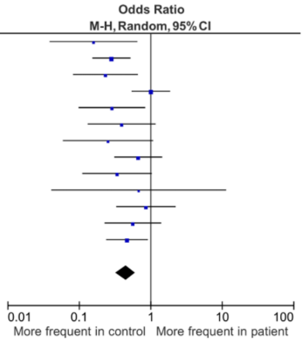

Figure S 49: Allele and genotype frequency of HLA-DRB1*07 in pemphigus

DRB1*08

Genotype frequency

Study or Subgroup	Patients Events	Patients Total	Control Events	Control Total	Weight	Odds Ratio M-H, Random, 95% CI
Fogo selvagem (Cerna, 1993)	1	10	34	74	4.3%	0.13 [0.02, 1.08]
Fogo selvagem (Pavoni, 2003)	6	128	62	402	8.2%	0.27 [0.11, 0.64]
Pemphigus (Lee, 1998)**	7	30	20	100	7.8%	1.22 [0.46, 3.24]
Pemphigus (Zhang, 2019)**	28	327	86	501	9.4%	0.45 [0.29, 0.71]
PF (Martel, 2002)	0	31	2	84	2.7%	0.52 [0.02, 11.22]
PV (Dere, 2020)	0	30	2	30	2.6%	0.19 [0.01, 4.06]
PV (Gil, 2017)	18	102	40	594	9.0%	2.97 [1.63, 5.42]
PV (Glorio, 1999)	9	30	29	199	8.2%	2.51 [1.05, 6.02]
PV (Glorio, 2002)	14	47	29	199	8.6%	2.49 [1.19, 5.21]
PV (Gonzalez-Escribano, 1998)	2	26	10	200	5.8%	1.58 [0.33, 7.66]
PV (Harfouch, 2014)	7	91	9	270	7.7%	2.42 [0.87, 6.69]
PV (Khan, 2015)	2	28	7	150	5.6%	1.57 [0.31, 7.99]
PV (Lombardi, 1996)	7	33	3	102	6.3%	8.88 [2.15, 36.75]
PV (Priyadarshini, 2018)	4	50	1	50	4.1%	4.26 [0.46, 39.54]
PV (Thomas, 1998)	2	26	10	200	5.8%	1.58 [0.33, 7.66]
PV (Tunca, 2010)	1	25	4	113	4.0%	1.14 [0.12, 10.62]
**Total (95% CI)**		**1014**		**3268**	**100.0%**	**1.33 [0.74, 2.38]**
Total events	108		348			

Heterogeneity: Tau² = 0.88; Chi² = 63.24, df = 15 (P < 0.00001); I² = 76%
Test for overall effect: Z = 0.95 (P = 0.34)

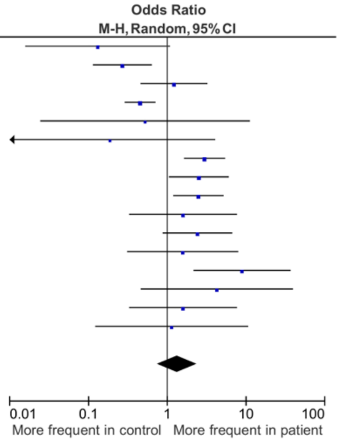

Allele frequency

Study or Subgroup	Patients Events	Patients Total	Control Events	Control Total	Weight	Odds Ratio M-H, Random, 95% CI
Pemphigus (Brochado, 2016) **	15	336	42	3184	12.2%	3.50 [1.92, 6.37]
Pemphigus (Torzecka, 2003)**	3	106	7	304	9.5%	1.24 [0.31, 4.87]
Pemphigus (Zhang, 2019)**	28	654	97	1002	12.6%	0.42 [0.27, 0.64]
PV (Párnická, 2013)	2	86	7	226	8.7%	0.74 [0.15, 3.66]
PV (Rangel-Gamboa, 2015) ‡	10	86	33	198	11.7%	0.66 [0.31, 1.40]
PV (Shams, 2008)	2	104	8	360	8.8%	0.86 [0.18, 4.13]
PV, Egypt (Haase, 2015)*	13	94	2	146	9.0%	11.56 [2.54, 52.49]
PV, Europe (Delgado, 1997)	1	38	8	496	7.0%	1.65 [0.20, 13.54]
PV, German (Haase, 2015)*	2	92	5	148	8.5%	0.64 [0.12, 3.35]
PV, Italians (Carcassi, 1996)	3	32	2	568	7.9%	29.28 [4.71, 182.08]
PV, Sardinians (Carcassi, 1996)	0	32	1	182	4.3%	1.86 [0.07, 46.70]
**Total (95% CI)**		**1660**		**6814**	**100.0%**	**1.62 [0.72, 3.67]**
Total events	79		212			

Heterogeneity: Tau² = 1.33; Chi² = 58.12, df = 10 (P < 0.00001); I² = 83%
Test for overall effect: Z = 1.16 (P = 0.24)

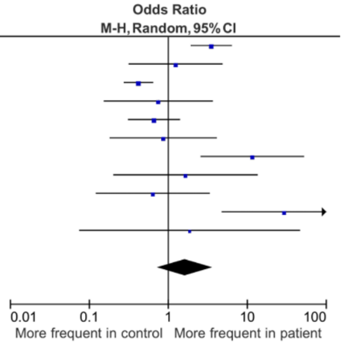

Figure S 50: Allele and genotype frequency of HLA-DRB1*08 in pemphigus

DRB1*09

Genotype frequency

Study or Subgroup	Patients Events	Patients Total	Control Events	Control Total	Weight	Odds Ratio M-H, Random, 95% CI
Fogo selvagem (Pavoni, 2003)	1	128	11	402	2.0%	0.28 [0.04, 2.19]
Pemphigus (Lee, 1998)**	0	30	20	100	1.1%	0.06 [0.00, 1.10]
Pemphigus (Zhang, 2019)**	79	327	175	501	88.0%	0.59 [0.43, 0.81]
PF (Martel, 2002)	1	31	1	84	1.1%	2.77 [0.17, 45.64]
PV (Gil, 2017)	1	102	9	594	2.0%	0.64 [0.08, 5.13]
PV (Gonzalez-Escribano, 1998)	0	26	6	200	1.0%	0.56 [0.03, 10.31]
PV (Harfouch, 2014)	0	91	2	270	0.9%	0.59 [0.03, 12.34]
PV (Khan, 2015)	0	28	6	150	1.0%	0.39 [0.02, 7.12]
PV (Priyadarshini, 2018)	0	50	1	50	0.8%	0.33 [0.01, 8.21]
PV (Thomas, 1998)	0	26	6	200	1.0%	0.56 [0.03, 10.31]
PV (Tunca, 2010)	0	25	4	113	1.0%	0.48 [0.02, 9.15]
**Total (95% CI)**		**864**		**2664**	**100.0%**	**0.57 [0.43, 0.77]**
Total events	82		241			

Heterogeneity: Tau² = 0.00; Chi² = 4.35, df = 10 (P = 0.93); I² = 0%
Test for overall effect: Z = 3.71 (P = 0.0002)

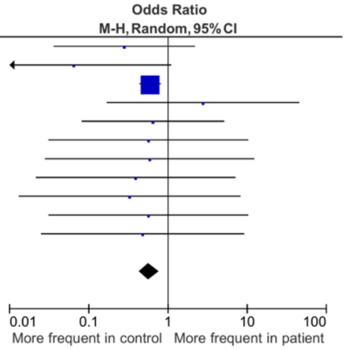

Allele frequency

Study or Subgroup	Patients Events	Patients Total	Control Events	Control Total	Weight	Odds Ratio M-H, Random, 95% CI
Pemphigus (Torzecka, 2003)**	2	106	3	304	2.3%	1.93 [0.32, 11.71]
Pemphigus (Zhang, 2019)**	82	654	195	1002	94.1%	0.59 [0.45, 0.78]
PV (Párnická, 2013)	0	86	0	226		Not estimable
PV (Shams, 2008)	2	104	7	360	2.9%	0.99 [0.20, 4.83]
PV, Egypt (Haase, 2015)*	0	94	0	146		Not estimable
PV, German (Haase, 2015)*	0	92	1	148	0.7%	0.53 [0.02, 13.19]
**Total (95% CI)**		**1136**		**2186**	**100.0%**	**0.62 [0.47, 0.81]**
Total events	86		206			

Heterogeneity: Tau² = 0.00; Chi² = 1.96, df = 3 (P = 0.58); I² = 0%
Test for overall effect: Z = 3.48 (P = 0.0005)

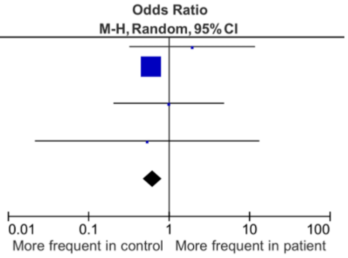

Figure S 51: Allele and genotype frequency of HLA-DRB1*09 in pemphigus

DRB1*10

Genotype frequency

Study or Subgroup	Patients Events	Patients Total	Control Events	Control Total	Weight	Odds Ratio M-H, Random, 95% CI
Fogo selvagem (Pavoni, 2003)	5	128	17	402	14.7%	0.92 [0.33, 2.55]
Pemphigus (Lee, 1998)**	8	30	7	100	13.2%	4.83 [1.58, 14.74]
Pemphigus (Zhang, 2019)**	9	327	8	501	15.7%	1.74 [0.67, 4.57]
PF (Martel, 2002)	7	31	16	84	15.0%	1.24 [0.45, 3.38]
PV (Gil, 2017)	3	102	13	594	11.2%	1.35 [0.38, 4.84]
PV (Gonzalez-Escribano, 1998)	0	26	4	200	2.9%	0.82 [0.04, 15.74]
PV (Harfouch, 2014)	3	91	12	270	11.0%	0.73 [0.20, 2.66]
PV (Khan, 2015)	0	28	27	150	3.1%	0.08 [0.00, 1.33]
PV (Priyadarshini, 2018)	2	50	5	50	7.4%	0.38 [0.07, 2.03]
PV (Thomas, 1998)	0	26	4	200	2.9%	0.82 [0.04, 15.74]
PV (Tunca, 2010)	0	25	5	113	2.9%	0.39 [0.02, 7.22]
**Total (95% CI)**		**864**		**2664**	**100.0%**	**1.13 [0.67, 1.91]**
Total events	37		118			

Heterogeneity: Tau² = 0.21; Chi² = 14.13, df = 10 (P = 0.17); I² = 29%
Test for overall effect: Z = 0.47 (P = 0.64)

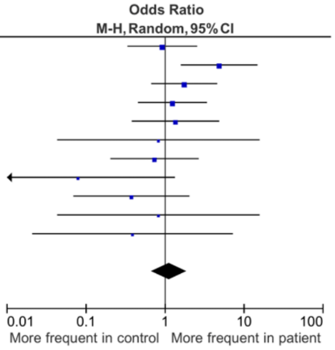

Allele frequency

Study or Subgroup	Patients Events	Patients Total	Control Events	Control Total	Weight	Odds Ratio M-H, Random, 95% CI
Pemphigus (Torzecka, 2003)**	0	106	7	304	8.1%	0.19 [0.01, 3.29]
Pemphigus (Zhang, 2019)**	9	654	8	1002	37.8%	1.73 [0.67, 4.52]
PV (Párnická, 2013)	3	86	2	226	17.3%	4.05 [0.66, 24.66]
PV (Shams, 2008)	2	104	12	360	22.3%	0.57 [0.13, 2.58]
PV, Egypt (Haase, 2015)*	0	94	2	146	7.3%	0.31 [0.01, 6.44]
PV, German (Haase, 2015)*	0	92	2	148	7.3%	0.32 [0.02, 6.67]
**Total (95% CI)**		**1136**		**2186**	**100.0%**	**1.02 [0.43, 2.43]**
Total events	14		33			

Heterogeneity: Tau² = 0.28; Chi² = 6.58, df = 5 (P = 0.25); I² = 24%
Test for overall effect: Z = 0.04 (P = 0.97)

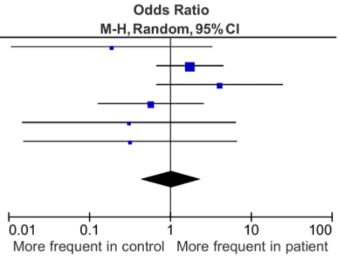

Figure S 52: Allele and genotype frequency of HLA-DRB1*10 in pemphigus

DRB1*11

Genotype frequency

Study or Subgroup	Patient Events	Patient Total	Control Events	Control Total	Weight	Odds Ratio M-H, Random, 95% CI
Fogo selvagem (Pavoni, 2003)	3	128	88	402	6.1%	0.09 [0.03, 0.28]
Pemphigus (Birol, 2002) ‡	6	33	40	100	7.1%	0.33 [0.13, 0.88]
Pemphigus (Koc, 2012) ‡	5	60	30	60	6.7%	0.09 [0.03, 0.26]
Pemphigus (Lee, 1998)**	0	30	4	100	1.8%	0.35 [0.02, 6.72]
Pemphigus (Zhang, 2019)**	31	327	81	501	9.9%	0.54 [0.35, 0.84]
PF (Martel, 2002)	3	31	14	84	5.4%	0.54 [0.14, 2.01]
PV (Dere, 2020)	7	30	13	30	6.4%	0.40 [0.13, 1.21]
PV (Gil, 2017)	11	102	52	594	8.6%	1.26 [0.63, 2.51]
PV (Gonzalez-Escribano, 1998)	7	26	44	200	7.3%	1.31 [0.52, 3.31]
PV (Harfouch, 2014)	18	91	136	270	9.3%	0.24 [0.14, 0.43]
PV (Khan, 2015)	2	28	28	150	4.7%	0.34 [0.08, 1.50]
PV (Lombardi, 1996)	11	33	54	102	7.9%	0.44 [0.20, 1.01]
PV (Priyadarshini, 2018)	2	50	9	50	4.4%	0.19 [0.04, 0.93]
PV (Thomas, 1998)	7	26	44	200	7.3%	1.31 [0.52, 3.31]
PV (Tunca, 2010)	6	25	41	113	7.0%	0.55 [0.21, 1.50]
**Total (95% CI)**		**1020**		**2956**	**100.0%**	**0.42 [0.27, 0.65]**
Total events	119		678			

Heterogeneity: Tau² = 0.43; Chi² = 43.57, df = 14 (P < 0.0001); I² = 68%
Test for overall effect: Z = 3.93 (P < 0.0001)

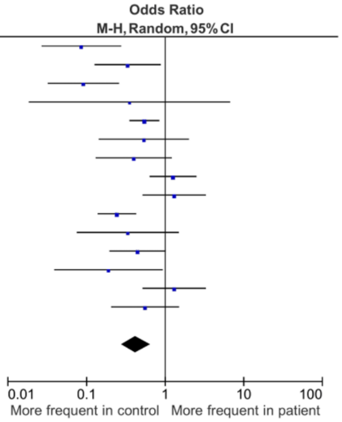

Allele frequency

Study or Subgroup	Patient Events	Patient Total	Control Events	Control Total	Weight	Odds Ratio M-H, Random, 95% CI
Pemphigus (Brochado, 2016) **	10	336	258	3184	11.8%	0.35 [0.18, 0.66]
Pemphigus (Torzecka, 2003)**	14	106	42	304	11.7%	0.95 [0.50, 1.82]
Pemphigus (Zhang, 2019)**	7	144	86	1002	10.3%	0.54 [0.25, 1.20]
PF (Abida, 2009)	4	180	73	540	8.3%	0.15 [0.05, 0.40]
PV (Párnická, 2013)	9	86	35	226	10.4%	0.64 [0.29, 1.39]
PV (Rangel-Gamboa, 2015) ‡	2	86	20	198	5.5%	0.21 [0.05, 0.93]
PV (Shams, 2008)	14	104	89	360	12.0%	0.47 [0.26, 0.87]
PV (Zivanovic, 2016)	10	144	673	3984	11.7%	0.37 [0.19, 0.70]
PV, Egypt (Haase, 2015)*	3	94	18	146	6.7%	0.23 [0.07, 0.82]
PV, Europe (Delgado, 1997)	0	38	2	26	1.7%	0.13 [0.01, 2.76]
PV, German (Haase, 2015)*	13	92	11	148	9.8%	2.05 [0.88, 4.79]
**Total (95% CI)**		**1410**		**10118**	**100.0%**	**0.47 [0.31, 0.72]**
Total events	86		1307			

Heterogeneity: Tau² = 0.29; Chi² = 26.61, df = 10 (P = 0.003); I² = 62%
Test for overall effect: Z = 3.48 (P = 0.0005)

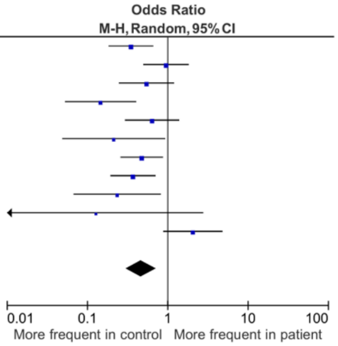

Figure S 53: Allele and genotype frequency of HLA-DRB1*11 in pemphigus

DRB1*12

Genotype frequency

Study or Subgroup	Patients Events	Patients Total	Control Events	Control Total	Weight	Odds Ratio M-H, Random, 95% CI
Fogo selvagem (Pavoni, 2003)	7	128	8	402	15.5%	2.85 [1.01, 8.02]
Pemphigus (Lee, 1998)**	0	30	12	100	3.6%	0.12 [0.01, 2.02]
Pemphigus (Zhang, 2019)**	84	327	102	501	28.5%	1.35 [0.97, 1.88]
PF (Martel, 2002)	2	31	1	84	4.7%	5.72 [0.50, 65.50]
PV (Dere, 2020)	1	30	1	30	3.7%	1.00 [0.06, 16.76]
PV (Gil, 2017)	1	102	16	594	6.4%	0.36 [0.05, 2.73]
PV (Harfouch, 2014)	0	91	6	270	3.5%	0.22 [0.01, 3.99]
PV (Khan, 2015)	6	28	23	150	16.0%	1.51 [0.55, 4.12]
PV (Priyadarshini, 2018)	3	50	10	50	11.4%	0.26 [0.07, 0.99]
PV (Thomas, 1998)	0	26	7	200	3.5%	0.49 [0.03, 8.77]
PV (Tunca, 2010)	0	25	3	113	3.3%	0.62 [0.03, 12.36]
**Total (95% CI)**		**868**		**2494**	**100.0%**	**1.00 [0.57, 1.78]**
Total events	104		189			

Heterogeneity: Tau² = 0.27; Chi² = 16.08, df = 10 (P = 0.10); I² = 38%
Test for overall effect: Z = 0.02 (P = 0.99)

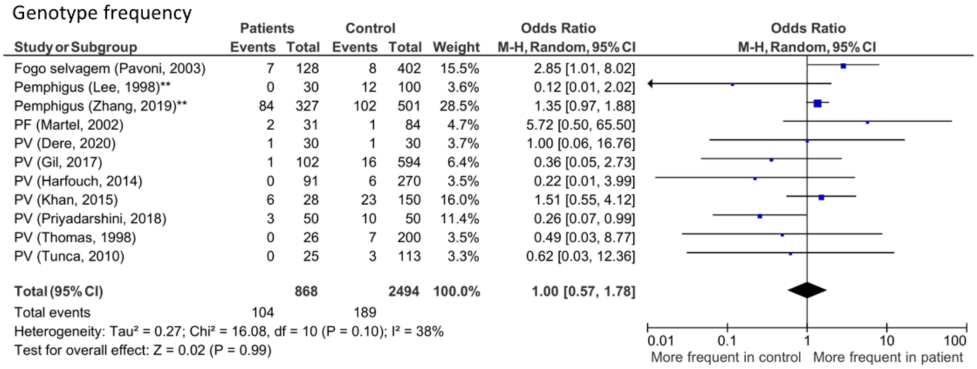

Allele frequency

Study or Subgroup	Patients Events	Patients Total	Control Events	Control Total	Weight	Odds Ratio M-H, Random, 95% CI
Pemphigus (Torzecka, 2003)**	0	106	14	304	1.0%	0.09 [0.01, 1.59]
Pemphigus (Zhang, 2019)**	87	654	126	1002	91.7%	1.07 [0.80, 1.43]
PV (Párnická, 2013)	0	86	3	226	0.9%	0.37 [0.02, 7.22]
PV (Shams, 2008)	0	104	3	360	0.9%	0.49 [0.03, 9.54]
PV (Zivanovic, 2016)	1	144	76	3984	2.0%	0.36 [0.05, 2.60]
PV, Egypt (Haase, 2015)*	0	94	1	146	0.8%	0.51 [0.02, 12.73]
PV, Europe (Delgado, 1997)	1	38	12	496	1.8%	1.09 [0.14, 8.61]
PV, German (Haase, 2015)*	0	92	4	148	0.9%	0.17 [0.01, 3.26]
**Total (95% CI)**		**1318**		**6666**	**100.0%**	**0.98 [0.74, 1.30]**
Total events	89		239			

Heterogeneity: Tau² = 0.00; Chi² = 6.34, df = 7 (P = 0.50); I² = 0%
Test for overall effect: Z = 0.13 (P = 0.89)

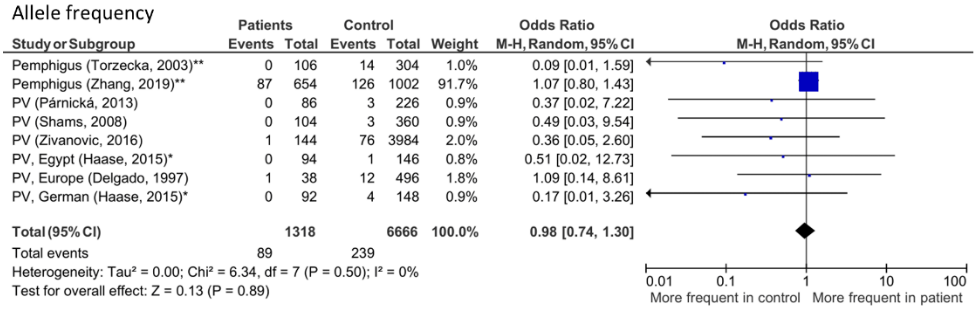

Figure S 54: Allele and genotype frequency of HLA-DRB1*12 in pemphigus

DRB1*13

Genotype frequency

Study or Subgroup	Patients Events	Patients Total	Control Events	Control Total	Weight	Odds Ratio M-H, Random, 95% CI
Fogo selvagem (Pavoni, 2003)	22	128	90	402	13.8%	0.72 [0.43, 1.20]
Pemphigus (Lee, 1998)**	0	30	18	100	2.4%	0.07 [0.00, 1.25]
Pemphigus (Zhang, 2019)**	25	327	36	501	13.6%	1.07 [0.63, 1.82]
PF (Martel, 2002)	6	31	18	84	9.3%	0.88 [0.31, 2.47]
PV (Dere, 2020)	2	30	6	30	5.4%	0.29 [0.05, 1.55]
PV (Gil, 2017)	4	102	81	594	9.4%	0.26 [0.09, 0.72]
PV (Gonzalez-Escribano, 1998)	0	26	55	200	2.5%	0.05 [0.00, 0.83]
PV (Harfouch, 2014)	5	91	54	270	10.0%	0.23 [0.09, 0.60]
PV (Khan, 2015)	6	28	23	150	9.5%	1.51 [0.55, 4.12]
PV (Lombardi, 1996)	2	33	14	102	6.1%	0.41 [0.09, 1.89]
PV (Priyadarshini, 2018)	2	50	6	50	5.6%	0.31 [0.06, 1.59]
PV (Thomas, 1998)	0	26	55	200	2.5%	0.05 [0.00, 0.83]
PV (Tunca, 2010)	7	25	30	113	9.8%	1.08 [0.41, 2.83]
**Total (95% CI)**		**927**		**2796**	**100.0%**	**0.51 [0.31, 0.82]**
Total events	81		486			

Heterogeneity: Tau² = 0.37; Chi² = 28.36, df = 12 (P = 0.005); I² = 58%
Test for overall effect: Z = 2.76 (P = 0.006)

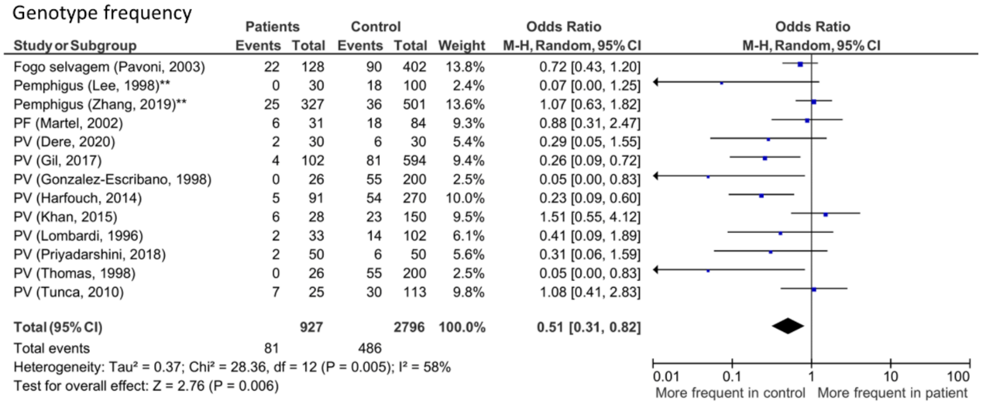

Allele frequency

Study or Subgroup	Patients Events	Patients Total	Control Events	Control Total	Weight	Odds Ratio M-H, Random, 95% CI
Pemphigus (Brochado, 2016) **	7	336	274	3184	10.2%	0.23 [0.11, 0.48]
Pemphigus (Torzecka, 2003)**	7	196	30	304	9.0%	0.34 [0.15, 0.79]
Pemphigus (Zhang, 2019)**	25	654	47	1002	15.0%	0.81 [0.49, 1.33]
PF (Abida, 2009)	10	180	74	540	11.4%	0.37 [0.19, 0.73]
PV (Párnická, 2013)	4	86	35	226	6.6%	0.27 [0.09, 0.77]
PV (Rangel-Gamboa, 2015) ‡	3	86	10	198	4.8%	0.68 [0.18, 2.53]
PV (Shams, 2008)	9	104	35	360	10.0%	0.88 [0.41, 1.89]
PV (Zivanovic, 2016)	10	144	527	3984	12.0%	0.49 [0.26, 0.94]
PV, Egypt (Haase, 2015)*	8	94	35	146	9.3%	0.30 [0.13, 0.67]
PV, Europe (Delgado, 1997)	3	38	54	496	5.4%	0.70 [0.21, 2.36]
PV, German (Haase, 2015)*	4	92	25	148	6.4%	0.22 [0.08, 0.67]
**Total (95% CI)**		**2010**		**10588**	**100.0%**	**0.44 [0.32, 0.60]**
Total events	90		1146			

Heterogeneity: Tau² = 0.12; Chi² = 16.98, df = 10 (P = 0.07); I² = 41%
Test for overall effect: Z = 5.02 (P < 0.00001)

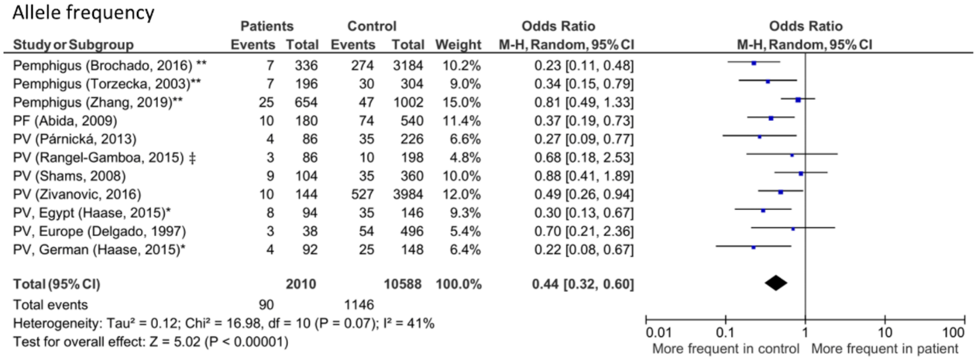

Figure S 55: Allele and genotype frequency of HLA-DRB1*13 in pemphigus

DRB1*14

Genotype frequency

Study or Subgroup	Patient Events	Patient Total	Control Events	Control Total	Weight	Odds Ratio M-H, Random, 95% CI
Fogo selvagem (Cerna, 1993)	8	10	38	74	3.9%	3.79 [0.75, 19.06]
Fogo selvagem (Pavoni, 2003)	22	128	100	402	6.0%	0.63 [0.38, 1.05]
Pemphigus (Birol, 2002) ‡	10	33	15	100	5.3%	2.46 [0.98, 6.20]
Pemphigus (Koc, 2012) ‡	28	60	9	60	5.4%	4.96 [2.07, 11.85]
Pemphigus (Lee, 1998)**	0	30	13	100	2.1%	0.11 [0.01, 1.84]
Pemphigus (Miyagawa, 1997)**	6	16	36	525	5.0%	8.15 [2.80, 23.69]
Pemphigus (Zhang, 2019)**	179	327	46	501	6.1%	11.96 [8.24, 17.38]
PF (Martel, 2002)	8	31	9	84	5.0%	2.90 [1.00, 8.37]
PV (Dere, 2020)	15	30	8	30	5.0%	2.75 [0.93, 8.10]
PV (Gil, 2017)	16	102	30	594	5.8%	3.50 [1.83, 6.69]
PV (Glorio, 1999)	12	30	20	199	5.4%	5.97 [2.51, 14.16]
PV (Glorio, 2002)	17	47	20	199	5.6%	5.07 [2.39, 10.77]
PV (Gonzalez-Escribano, 1998)	8	26	8	200	4.9%	10.67 [3.58, 31.81]
PV (Harfouch, 2014)	51	91	30	270	5.9%	10.20 [5.82, 17.89]
PV (Khan, 2015)	2	28	21	150	4.1%	0.47 [0.10, 2.14]
PV (Lombardi, 1996)	19	33	9	102	5.2%	14.02 [5.31, 37.07]
PV (Priyadarshini, 2018)	47	50	18	50	4.5%	27.85 [7.57, 102.42]
PV (Thomas, 1998)	8	26	8	200	4.9%	10.67 [3.58, 31.81]
PV (Tunca, 2010)	8	25	10	113	5.0%	4.85 [1.68, 14.02]
PV (Yamashina, 1998)	9	17	36	525	5.1%	15.28 [5.56, 41.99]
**Total (95% CI)**		**1140**		**4478**	**100.0%**	**4.81 [2.88, 8.05]**
Total events	473		484			

Heterogeneity: Tau² = 1.09; Chi² = 134.89, df = 19 (P < 0.00001); I² = 86%

Test for overall effect: Z = 5.99 (P < 0.00001)

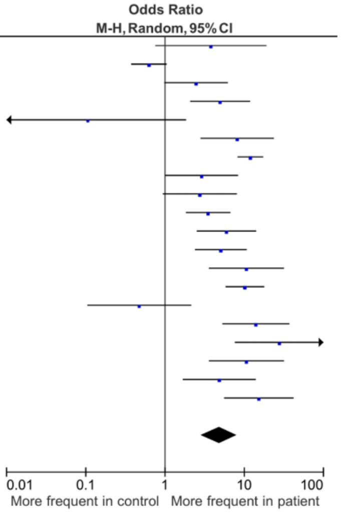

Allele frequency

Study or Subgroup	Patient Events	Patient Total	Control Events	Control Total	Weight	Odds Ratio M-H, Random, 95% CI
Pemphigus (Brochado, 2016) **	33	336	77	3184	12.8%	4.39 [2.87, 6.72]
Pemphigus (Torzecka, 2003)**	11	106	6	304	3.7%	5.75 [2.07, 15.97]
Pemphigus (Zhang, 2019)**	177	654	50	1002	16.1%	7.07 [5.07, 9.85]
PF, Indo-Asian (Saha, 2019)	9	20	14	118	3.5%	6.08 [2.14, 17.24]
PF, White British (Saha, 2019)	4	50	8	200	2.6%	2.09 [0.60, 7.23]
PV (Párnická, 2013)	19	86	6	226	4.1%	10.40 [3.99, 27.09]
PV (Rangel-Gamboa, 2015) ‡	20	86	21	198	7.1%	2.55 [1.30, 5.01]
PV (Shams, 2008)	26	104	18	360	7.5%	6.33 [3.31, 12.12]
PV (Zivanovic, 2016)	44	144	216	3984	14.3%	7.68 [5.25, 11.23]
PV, Egypt (Haase, 2015)*	14	94	2	146	1.8%	12.60 [2.79, 56.84]
PV, Europe (Delgado, 1997)	21	38	7	26	3.3%	3.35 [1.14, 9.84]
PV, German (Haase, 2015)*	20	92	3	148	2.6%	13.43 [3.86, 46.67]
PV, Indo-Asians (Saha, 2010)*	53	114	14	118	7.2%	6.45 [3.31, 12.59]
PV, Italians (Carcassi, 1996)	12	32	32	568	5.5%	10.05 [4.52, 22.36]
PV, Sardinians (Carcassi, 1996)	5	32	4	182	2.1%	8.24 [2.08, 32.62]
PV, White Europeans (Saha, 2010)*	44	192	8	200	5.7%	7.14 [3.26, 15.62]
**Total (95% CI)**		**2180**		**10964**	**100.0%**	**6.14 [4.98, 7.58]**
Total events	512		486			

Heterogeneity: Tau² = 0.04; Chi² = 20.36, df = 15 (P = 0.16); I² = 26%

Test for overall effect: Z = 16.93 (P < 0.00001)

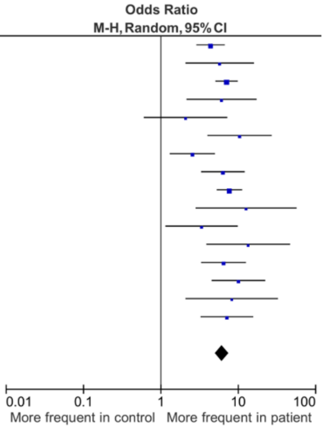

Figure S 56: Allele and genotype frequency of HLA-DRB1*14 in pemphigus

DRB1*15

Genotype frequency

Study or Subgroup	Patient Events	Patient Total	Control Events	Control Total	Weight	Odds Ratio M-H, Random, 95% CI
Fogo selvagem (Moraes, 1991)	2	38	11	46	2.2%	0.18 [0.04, 0.86]
Fogo selvagem (Pavoni, 2003)	18	128	97	402	18.3%	0.51 [0.30, 0.89]
Pemphigus (Lee, 1998)**	3	30	23	100	3.4%	0.37 [0.10, 1.34]
Pemphigus (Zhang, 2019)**	56	327	139	501	45.5%	0.54 [0.38, 0.76]
PF (Martel, 2002)	2	31	16	84	2.3%	0.29 [0.06, 1.36]
PV (Dere, 2020)	5	30	8	30	3.5%	0.55 [0.16, 1.93]
PV (Gil, 2017)	3	102	61	594	4.0%	0.26 [0.08, 0.86]
PV (Glorio, 1999)	1	30	39	199	1.3%	0.14 [0.02, 1.07]
PV (Glorio, 2002)	1	47	39	199	1.4%	0.09 [0.01, 0.67]
PV (Khan, 2015)	8	28	59	150	7.1%	0.62 [0.26, 1.49]
PV (Lombardi, 1996)	3	33	10	102	3.0%	0.92 [0.24, 3.56]
PV (Priyadarshini, 2018)	7	50	16	50	5.6%	0.35 [0.13, 0.94]
PV (Tunca, 2010)	2	25	20	113	2.4%	0.40 [0.09, 1.86]
**Total (95% CI)**		**899**		**2570**	**100.0%**	**0.47 [0.37, 0.59]**
Total events	111		538			

Heterogeneity: Tau² = 0.00; Chi² = 9.60, df = 12 (P = 0.65); I² = 0%

Test for overall effect: Z = 6.30 (P < 0.00001)

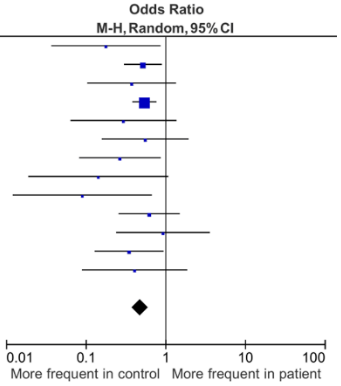

Allele frequency

Study or Subgroup	Patient Events	Patient Total	Control Events	Control Total	Weight	Odds Ratio M-H, Random, 95% CI
Pemphigus (Torzecka, 2003)**	7	106	51	304	7.4%	0.35 [0.15, 0.80]
Pemphigus (Zhang, 2019)**	58	654	178	1002	50.7%	0.45 [0.33, 0.62]
PF (Abida, 2009)	8	180	56	540	8.7%	0.40 [0.19, 0.86]
PV (Párnická, 2013)	2	86	29	226	2.4%	0.16 [0.04, 0.69]
PV (Shams, 2008)	4	104	47	360	4.6%	0.27 [0.09, 0.76]
PV (Zivanovic, 2016)	5	144	392	3984	6.2%	0.33 [0.13, 0.81]
PV, Egypt (Haase, 2015)*	2	94	17	146	2.3%	0.16 [0.04, 0.73]
PV, German (Haase, 2015)*	6	92	18	148	5.4%	0.50 [0.19, 1.32]
PV, Indo-Asians (Saha, 2010)*	5	114	25	118	5.0%	0.17 [0.06, 0.46]
PV, White Europeans (Saha, 2010)*	8	192	25	200	7.4%	0.30 [0.13, 0.69]
**Total (95% CI)**		**1766**		**7028**	**100.0%**	**0.37 [0.30, 0.47]**
Total events	105		838			

Heterogeneity: Tau² = 0.00; Chi² = 7.37, df = 9 (P = 0.60); I² = 0%

Test for overall effect: Z = 8.65 (P < 0.00001)

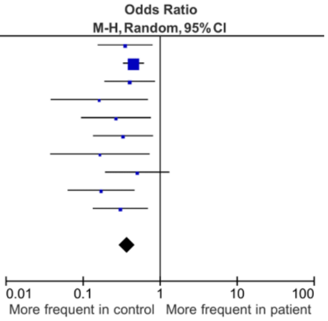

Figure S 57: Allele and genotype frequency of HLA-DRB1*15 in pemphigus

DRB1*16

Genotype frequency

Study or Subgroup	Patients Events	Patients Total	Control Events	Control Total	Weight	Odds Ratio M-H, Random, 95% CI
Fogo selvagem (Cerna, 1993)	4	10	38	74	10.8%	0.63 [0.16, 2.42]
Fogo selvagem (Moraes, 1991)	8	37	2	49	9.1%	6.48 [1.29, 32.66]
Fogo selvagem (Pavoni, 2003)	21	128	39	402	17.0%	1.83 [1.03, 3.24]
Pemphigus (Lee, 1998)**	1	30	2	100	5.4%	1.69 [0.15, 19.31]
Pemphigus (Zhang, 2019)**	0	327	7	501	4.2%	0.10 [0.01, 1.77]
PF (Martel, 2002)	4	31	3	84	9.4%	4.00 [0.84, 19.02]
PV (Gil, 2017)	2	102	19	594	10.0%	0.61 [0.14, 2.64]
PV (Harfouch, 2014)	3	91	23	270	11.7%	0.37 [0.11, 1.25]
PV (Khan, 2015)	0	28	4	150	4.0%	0.57 [0.03, 10.90]
PV (Lombardi, 1996)	3	33	8	102	10.5%	1.18 [0.29, 4.71]
PV (Priyadarshini, 2018)	1	50	0	50	3.5%	3.06 [0.12, 76.95]
PV (Tunca, 2010)	0	25	25	113	4.3%	0.07 [0.00, 1.16]
**Total (95% CI)**		**892**		**2489**	**100.0%**	**1.03 [0.53, 2.01]**
Total events	47		170			

Heterogeneity: Tau² = 0.59; Chi² = 22.45, df = 11 (P = 0.02); I² = 51%

Test for overall effect: Z = 0.10 (P = 0.92)

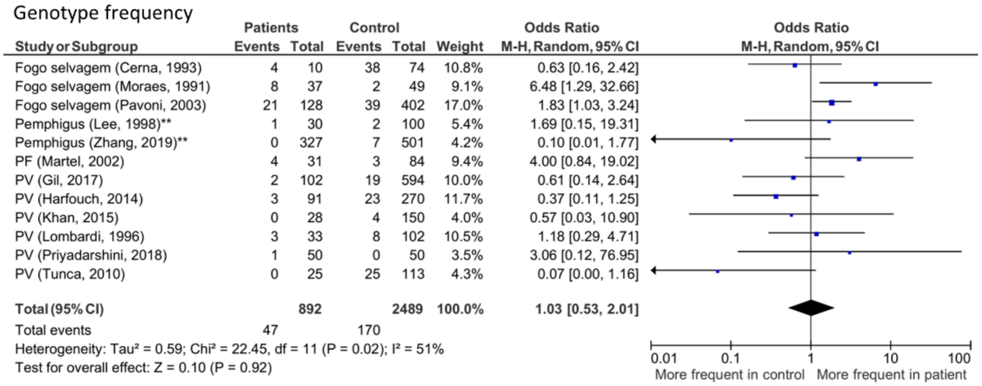

Allele frequency

Study or Subgroup	Patients Events	Patients Total	Control Events	Control Total	Weight	Odds Ratio M-H, Random, 95% CI
Fogo selvagem (de Sena Nogueira Maehara, 2017)	10	84	36	956	20.1%	3.45 [1.65, 7.23]
Pemphigus (Zhang, 2019)**	0	654	8	1002	9.4%	0.09 [0.01, 1.55]
PV (Párnická, 2013)	1	86	14	226	13.0%	0.18 [0.02, 1.38]
PV (Rangel-Gamboa, 2015) ‡	2	86	5	198	15.1%	0.92 [0.17, 4.83]
PV (Shams, 2008)	1	104	16	360	13.1%	0.21 [0.03, 1.59]
PV (Zivanovic, 2016)	8	144	433	3984	20.2%	0.48 [0.23, 0.99]
PV, Egypt (Haase, 2015)*	0	94	0	146		Not estimable
PV, German (Haase, 2015)*	0	92	4	148	9.1%	0.17 [0.01, 3.26]
**Total (95% CI)**		**1344**		**7020**	**100.0%**	**0.48 [0.15, 1.54]**
Total events	22		516			

Heterogeneity: Tau² = 1.60; Chi² = 26.39, df = 6 (P = 0.0002); I² = 77%

Test for overall effect: Z = 1.23 (P = 0.22)

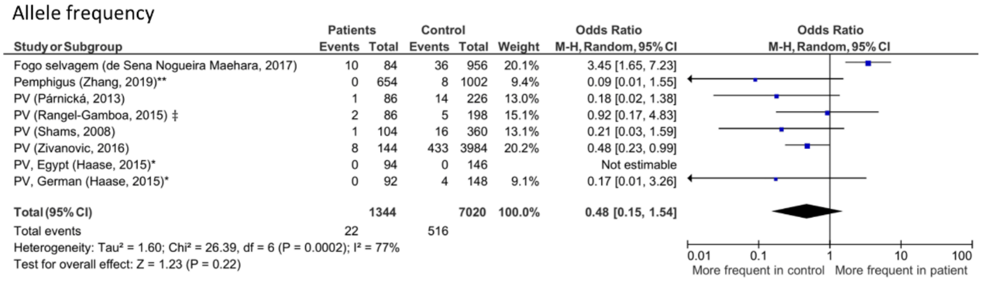

Figure S 58: Allele and genotype frequency of HLA-DRB1*16 in pemphigus

DQB1*02

Genotype frequency

Study or Subgroup	Patients Events	Patients Total	Control Events	Control Total	Weight	Odds Ratio M-H, Random, 95% CI
Fogo selvagem (Moraes, 1991)	0	37	11	49	1.1%	0.04 [0.00, 0.78]
Pemphigus (Birol, 2002) ‡	1	33	26	100	2.2%	0.09 [0.01, 0.68]
Pemphigus (Koc, 2012) ‡	8	60	17	60	10.0%	0.39 [0.15, 0.99]
Pemphigus (Lee, 1998)**	1	30	21	100	2.2%	0.13 [0.02, 1.01]
Pemphigus (Zhang, 2019)**	21	327	58	501	28.4%	0.52 [0.31, 0.88]
PF (Martel, 2002)	6	30	28	64	8.5%	0.32 [0.12, 0.89]
PV (Dere, 2020)	2	30	14	30	3.5%	0.08 [0.02, 0.41]
PV (Gil, 2017)	9	102	134	594	16.6%	0.33 [0.16, 0.68]
PV (Lombardi, 1996)	3	33	35	102	5.7%	0.19 [0.05, 0.67]
PV (Orouji, 2014)	7	54	23	85	10.2%	0.40 [0.16, 1.01]
PV (Priyadarshini, 2018)	4	50	11	50	6.0%	0.31 [0.09, 1.05]
PV (Tunca, 2010)	3	25	37	113	5.6%	0.28 [0.08, 1.00]
**Total (95% CI)**		**811**		**1848**	**100.0%**	**0.33 [0.24, 0.45]**
Total events	65		415			

Heterogeneity: Tau² = 0.01; Chi² = 11.57, df = 11 (P = 0.40); I² = 5%

Test for overall effect: Z = 7.11 (P < 0.00001)

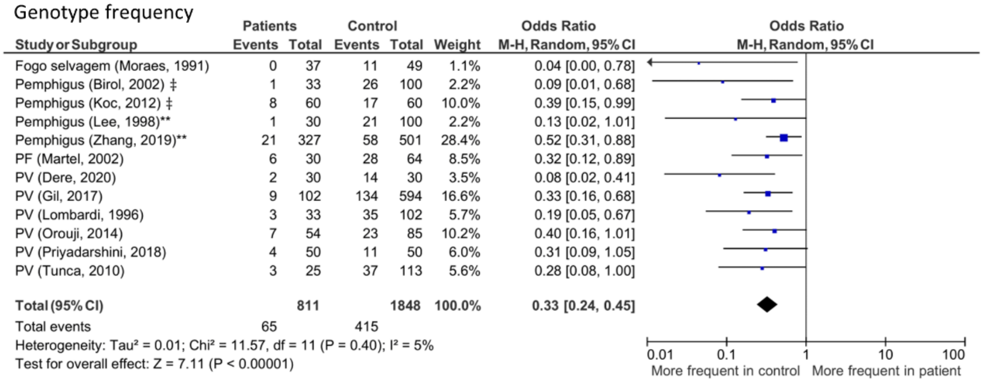

Allele frequency

Study or Subgroup	Patients Events	Patients Total	Control Events	Control Total	Weight	Odds Ratio M-H, Random, 95% CI
Pemphigus (Zhang, 2019)**	21	654	60	1002	24.1%	0.52 [0.31, 0.86]
PV (Orouji, 2014)	8	108	28	170	9.9%	0.41 [0.18, 0.93]
PV (Párnická, 2013)	8	86	44	226	10.6%	0.42 [0.19, 0.94]
PV (Shams, 2008)	3	104	69	360	5.0%	0.13 [0.04, 0.41]
PV (Zivanovic, 2016)	12	144	54	318	15.1%	0.44 [0.23, 0.86]
PV, Europe (Delgado, 1997)	2	38	45	496	3.3%	0.56 [0.13, 2.39]
PV, Indo-Asians (Saha, 2010)*	7	114	10	118	6.9%	0.71 [0.26, 1.92]
PV, Italians (Carcassi, 1996)	0	32	163	812	0.9%	0.06 [0.00, 1.00]
PV, Sardinians (Carcassi, 1996)	3	32	53	182	4.6%	0.25 [0.07, 0.86]
PV, White Europeans (Saha, 2010)*	19	192	51	200	19.6%	0.32 [0.18, 0.57]
**Total (95% CI)**		**1504**		**3884**	**100.0%**	**0.40 [0.31, 0.52]**
Total events	83		577			

Heterogeneity: Tau² = 0.01; Chi² = 9.54, df = 9 (P = 0.39); I² = 6%

Test for overall effect: Z = 6.72 (P < 0.00001)

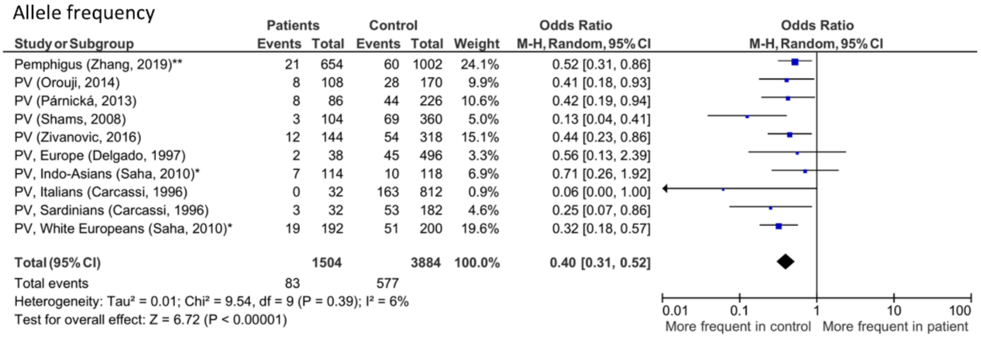

Figure S 59: Allele and genotype frequency of HLA-DQB1*02 in pemphigus

DQB1*03

Genotype frequency

Study or Subgroup	Patients Events	Patients Total	Control Events	Control Total	Weight	Odds Ratio M-H, Random, 95% CI
Fogo selvagem (Cerna, 1993)	8	10	63	74	5.3%	0.70 [0.13, 3.73]
Pemphigus (Lee, 1998)**	17	30	64	100	8.0%	0.74 [0.32, 1.69]
Pemphigus (Miyagawa, 1997)**	8	16	90	525	7.4%	4.83 [1.77, 13.22]
Pemphigus (Zhang, 2019)**	241	327	365	501	9.4%	1.04 [0.76, 1.43]
PF (Martel, 2002)	13	30	13	64	7.6%	3.00 [1.17, 7.72]
PV (Dere, 2020)	29	30	22	30	4.1%	10.55 [1.23, 90.66]
PV (Gil, 2017)	51	102	159	594	9.1%	2.74 [1.78, 4.20]
PV (Glorio, 1999)	15	30	18	199	7.9%	10.06 [4.24, 23.86]
PV (Glorio, 2002)	25	47	18	199	8.3%	11.43 [5.40, 24.20]
PV (Lombardi, 1996)	28	33	68	102	7.3%	2.80 [0.99, 7.90]
PV (Orouji, 2014)	54	54	75	85	2.9%	15.16 [0.87, 264.26]
PV (Priyadarshini, 2018)	14	50	25	50	8.0%	0.39 [0.17, 0.89]
PV (Tunca, 2010)	21	25	71	113	7.0%	3.11 [1.00, 9.66]
PV (Yamashina, 1998)	9	17	90	525	7.5%	5.44 [2.04, 14.47]
**Total (95% CI)**		**801**		**3161**	**100.0%**	**2.77 [1.56, 4.92]**
Total events	533		1141			

Heterogeneity: Tau² = 0.89; Chi² = 87.57, df = 13 (P < 0.00001); I² = 85%

Test for overall effect: Z = 3.47 (P = 0.0005)

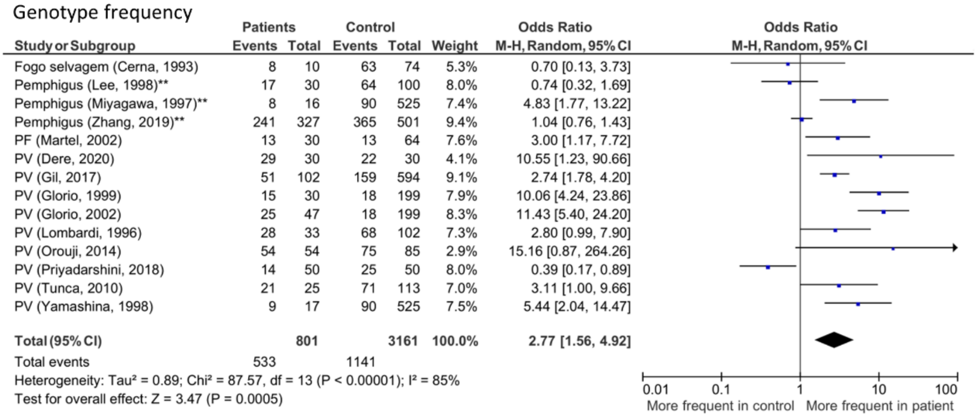

Allele frequency

Study or Subgroup	Patients Events	Patients Total	Control Events	Control Total	Weight	Odds Ratio M-H, Random, 95% CI
Pemphigus (Brochado, 2016) **	123	336	923	3184	9.2%	1.41 [1.12, 1.79]
Pemphigus (Zhang, 2019)**	309	654	529	1002	9.3%	0.80 [0.66, 0.98]
PF (Abida, 2009)	53	180	171	540	8.7%	0.90 [0.62, 1.30]
PF, Indo-Asian (Saha, 2019)	2	20	8	118	3.2%	1.53 [0.30, 7.78]
PF, White British (Saha, 2019)	18	50	18	200	6.7%	5.69 [2.68, 12.08]
PV (Orouji, 2014)	82	108	105	170	7.9%	1.95 [1.14, 3.35]
PV (Párnická, 2013)	45	86	74	226	8.0%	2.25 [1.36, 3.74]
PV (Shams, 2008)	58	104	138	360	8.4%	2.03 [1.30, 3.15]
PV (Zivanovic, 2016)	58	144	106	318	8.5%	1.35 [0.90, 2.03]
PV, Europe (Delgado, 1997)	4	38	6	26	3.9%	0.39 [0.10, 1.56]
PV, Indo-Asians (Saha, 2010)*	20	114	10	118	6.4%	2.30 [1.02, 5.15]
PV, Italians (Carcassi, 1996)	7	32	37	812	5.9%	5.86 [2.38, 14.44]
PV, Sardinians (Carcassi, 1996)	13	32	9	182	5.6%	13.15 [4.97, 34.80]
PV, White Europeans (Saha, 2010)*	66	192	31	200	8.1%	2.86 [1.76, 4.64]
**Total (95% CI)**		**2090**		**7456**	**100.0%**	**1.99 [1.39, 2.83]**
Total events	858		2165			

Heterogeneity: Tau² = 0.34; Chi² = 99.95, df = 13 (P < 0.00001); I² = 87%

Test for overall effect: Z = 3.78 (P = 0.0002)

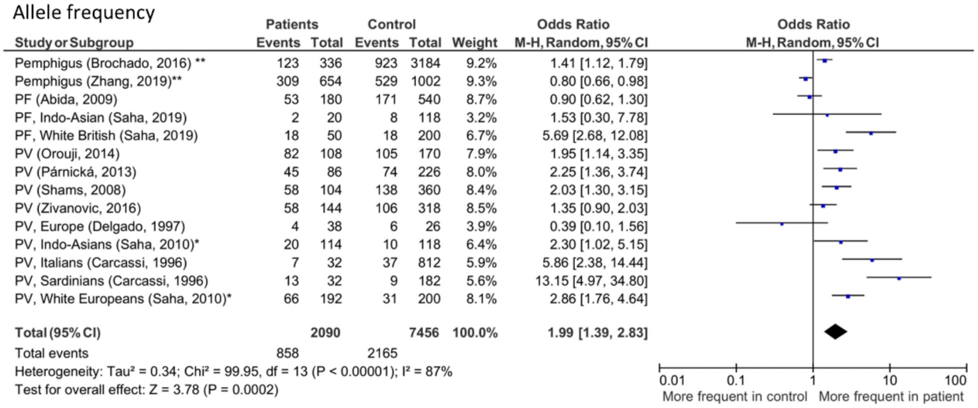

Figure S 60: Allele and genotype frequency of HLA-DQB1*03 in pemphigus

DQB1*04

Genotype frequency

Study or Subgroup	Patients Events	Patients Total	Control Events	Control Total	Weight	Odds Ratio M-H, Random, 95% CI
Fogo selvagem (Cerna, 1993)	1	10	34	74	7.8%	0.13 [0.02, 1.08]
Pemphigus (Birol, 2002) ‡	3	33	1	100	7.0%	9.90 [0.99, 98.72]
Pemphigus (Lee, 1998)**	8	30	25	100	15.8%	1.09 [0.43, 2.76]
Pemphigus (Zhang, 2019)**	15	327	57	501	18.5%	0.37 [0.21, 0.67]
PV (Dere, 2020)	0	30	1	30	4.3%	0.32 [0.01, 8.24]
PV (Gil, 2017)	8	102	46	594	17.0%	1.01 [0.46, 2.22]
PV (Lombardi, 1996)	4	33	1	102	7.3%	13.93 [1.50, 129.54]
PV (Orouji, 2014)	0	54	2	85	4.7%	0.31 [0.01, 6.51]
PV (Priyadarshini, 2018)	3	50	1	50	7.0%	3.13 [0.31, 31.14]
PV (Tunca, 2010)	2	25	7	113	10.5%	1.32 [0.26, 6.75]
**Total (95% CI)**		**694**		**1749**	**100.0%**	**1.04 [0.49, 2.19]**
Total events	44		175			

Heterogeneity: Tau² = 0.70; Chi² = 23.18, df = 9 (P = 0.006); I² = 61%

Test for overall effect: Z = 0.09 (P = 0.93)

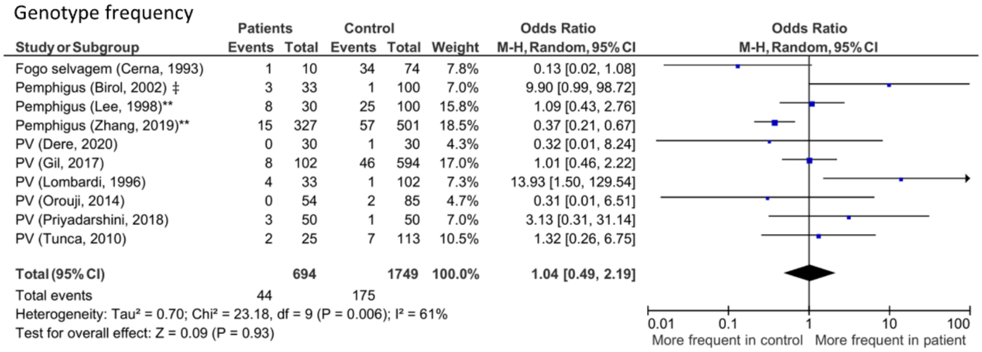

Allele frequency

Study or Subgroup	Patients Events	Patients Total	Control Events	Control Total	Weight	Odds Ratio M-H, Random, 95% CI
Pemphigus (Zhang, 2019)**	16	654	65	1002	64.0%	0.36 [0.21, 0.63]
PV (Orouji, 2014)	0	108	2	170	3.3%	0.31 [0.01, 6.53]
PV (Párnická, 2013)	3	86	6	226	14.5%	1.33 [0.32, 5.42]
PV (Shams, 2008)	2	104	6	360	11.3%	1.16 [0.23, 5.82]
PV (Zivanovic, 2016)	1	144	7	318	6.8%	0.31 [0.04, 2.55]
**Total (95% CI)**		**1096**		**2076**	**100.0%**	**0.49 [0.28, 0.86]**
Total events	22		86			

Heterogeneity: Tau² = 0.05; Chi² = 4.36, df = 4 (P = 0.36); I² = 8%

Test for overall effect: Z = 2.49 (P = 0.01)

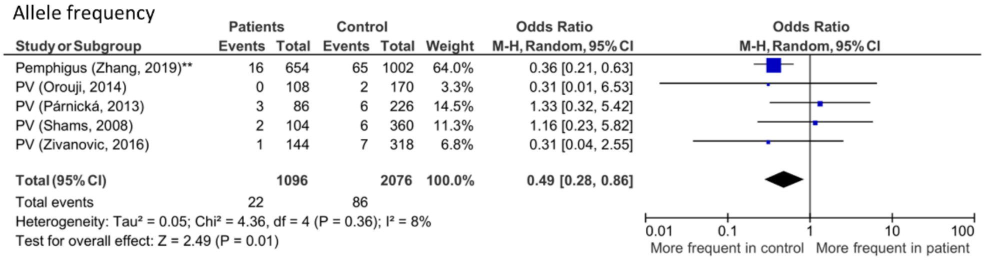

Figure S 61: Allele and genotype frequency of HLA-DQB1*04 in pemphigus

DQB1*05

Genotype frequency

Study or Subgroup	Patient Events	Patient Total	Control Events	Control Total	Weight	Odds Ratio M-H, Random, 95% CI
Pemphigus (Koc, 2012) ‡	38	60	21	60	7.7%	3.21 [1.52, 6.77]
Pemphigus (Lee, 1998)**	14	30	43	100	7.5%	1.16 [0.51, 2.63]
Pemphigus (Miyagawa, 1997)**	5	16	25	525	6.4%	9.09 [2.93, 28.17]
Pemphigus (Zhang, 2019)**	200	327	99	501	8.9%	6.39 [4.68, 8.74]
PF (Martel, 2002)	8	30	8	64	6.6%	2.55 [0.85, 7.62]
PV (Dere, 2020)	17	30	3	30	5.6%	11.77 [2.92, 47.46]
PV (Gil, 2017)	20	102	124	594	8.4%	0.92 [0.55, 1.57]
PV (Glorio, 1999)	11	30	10	199	7.0%	10.94 [4.12, 29.09]
PV (Glorio, 2002)	14	47	10	199	7.3%	8.02 [3.29, 19.56]
PV (Lombardi, 1996)	23	33	31	102	7.4%	5.27 [2.24, 12.37]
PV (Orouji, 2014)	9	54	17	170	7.3%	1.80 [0.75, 4.31]
PV (Priyadarshini, 2018)	47	50	24	50	5.9%	16.97 [4.66, 61.80]
PV (Tunca, 2010)	10	25	38	113	7.3%	1.32 [0.54, 3.20]
PV (Yamashina, 1998)	9	17	36	525	6.9%	15.28 [5.56, 41.99]
**Total (95% CI)**		**851**		**3232**	**100.0%**	**4.30 [2.53, 7.28]**
Total events	425		489			

Heterogeneity: Tau² = 0.79; Chi² = 80.26, df = 13 (P < 0.00001); I² = 84%

Test for overall effect: Z = 5.42 (P < 0.00001)

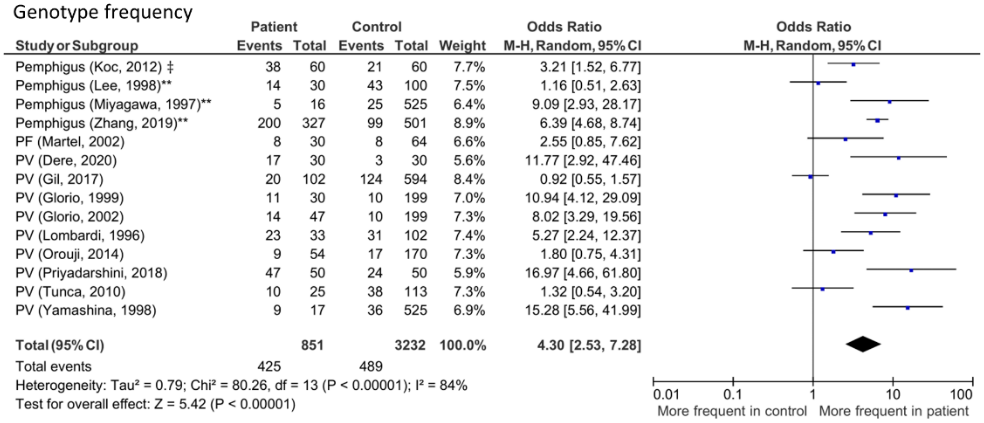

Allele frequency

Study or Subgroup	Patient Events	Patient Total	Control Events	Control Total	Weight	Odds Ratio M-H, Random, 95% CI
Pemphigus (Brochado, 2016) **	60	172	397	3184	9.3%	3.76 [2.70, 5.23]
Pemphigus (Zhang, 2019)**	223	654	117	1002	9.6%	3.91 [3.04, 5.03]
PF, Indo-Asian (Saha, 2019)	10	20	25	118	5.9%	3.72 [1.39, 9.93]
PF, White British (Saha, 2019)	19	50	41	200	7.6%	2.38 [1.22, 4.63]
PV (Orouji, 2014)	10	108	17	170	6.7%	0.92 [0.40, 2.09]
PV (Párnická, 2013)	29	86	48	226	8.3%	1.89 [1.09, 3.27]
PV (Shams, 2008)	30	104	75	360	8.6%	1.54 [0.94, 2.53]
PV (Zivanovic, 2016)	59	144	105	318	9.0%	1.41 [0.94, 2.11]
PV, Europe (Delgado, 1997)	19	38	79	496	7.5%	5.28 [2.67, 10.42]
PV, Indo-Asians (Saha, 2010)*	58	114	25	118	8.1%	3.85 [2.17, 6.84]
PV, Italians (Carcassi, 1996)	12	32	15	812	6.4%	31.88 [13.23, 76.80]
PV, Sardinians (Carcassi, 1996)	5	32	4	182	4.2%	8.24 [2.08, 32.62]
PV, White Europeans (Saha, 2010)*	55	192	36	200	8.6%	1.83 [1.13, 2.95]
**Total (95% CI)**		**1746**		**7386**	**100.0%**	**3.04 [2.10, 4.41]**
Total events	589		984			

Heterogeneity: Tau² = 0.36; Chi² = 75.49, df = 12 (P < 0.00001); I² = 84%

Test for overall effect: Z = 5.87 (P < 0.00001)

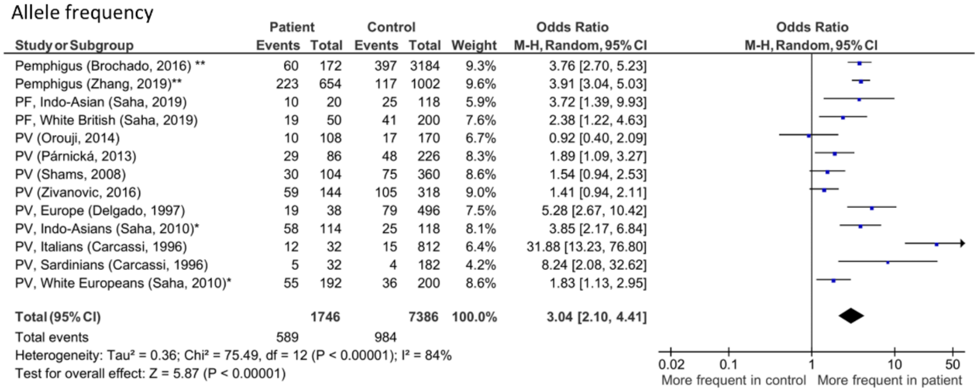

Figure S 62: Allele and genotype frequency of HLA-DQB1*05 in pemphigus

DQB1*06

Genotype frequency

Study or Subgroup	Patients Events	Patients Total	Control Events	Control Total	Weight	Odds Ratio M-H, Random, 95% CI
Fogo selvagem (Moraes, 1991)	3	38	15	41	6.6%	0.15 [0.04, 0.57]
Pemphigus (Koc, 2012) ‡	6	60	17	60	9.1%	0.28 [0.10, 0.77]
Pemphigus (Lee, 1998)**	20	30	41	100	10.6%	2.88 [1.22, 6.78]
Pemphigus (Zhang, 2019)**	69	327	179	501	16.5%	0.48 [0.35, 0.66]
PV (Dere, 2020)	5	30	14	30	7.6%	0.23 [0.07, 0.76]
PV (Gil, 2017)	14	102	131	594	13.5%	0.56 [0.31, 1.02]
PV (Lombardi, 1996)	5	33	22	102	8.7%	0.65 [0.22, 1.88]
PV (Orouji, 2014)	7	54	18	85	9.7%	0.55 [0.21, 1.43]
PV (Priyadarshini, 2018)	8	50	19	50	9.7%	0.31 [0.12, 0.80]
PV (Tunca, 2010)	4	25	40	113	8.1%	0.35 [0.11, 1.08]
**Total (95% CI)**		**749**		**1676**	**100.0%**	**0.48 [0.31, 0.74]**
Total events	141		496			

Heterogeneity: Tau² = 0.27; Chi² = 23.89, df = 9 (P = 0.004); I² = 62%

Test for overall effect: Z = 3.32 (P = 0.0009)

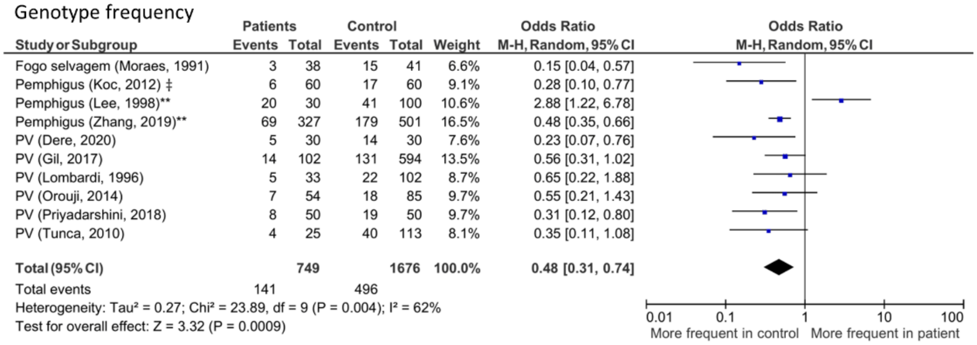

Allele frequency

Study or Subgroup	Patients Events	Patients Total	Control Events	Control Total	Weight	Odds Ratio M-H, Random, 95% CI
Pemphigus (Brochado, 2016) **	26	336	489	3184	18.8%	0.46 [0.31, 0.70]
Pemphigus (Zhang, 2019)**	75	654	231	1002	33.4%	0.43 [0.33, 0.57]
PF (Abida, 2009)	18	180	106	540	12.1%	0.45 [0.27, 0.77]
PV (Orouji, 2014)	8	108	18	170	4.9%	0.68 [0.28, 1.61]
PV (Párnická, 2013)	3	86	54	226	2.6%	0.12 [0.03, 0.38]
PV (Shams, 2008)	11	104	73	360	7.8%	0.47 [0.24, 0.91]
PV (Zivanovic, 2016)	14	144	46	318	8.8%	0.64 [0.34, 1.20]
PV, Europe (Delgado, 1997)	2	38	89	496	1.8%	0.25 [0.06, 1.07]
PV, Indo-Asians (Saha, 2010)*	5	114	21	118	3.6%	0.21 [0.08, 0.58]
PV, White Europeans (Saha, 2010)*	10	192	24	200	6.2%	0.40 [0.19, 0.87]
**Total (95% CI)**		**1956**		**6614**	**100.0%**	**0.43 [0.36, 0.53]**
Total events	172		1151			

Heterogeneity: Tau² = 0.01; Chi² = 9.89, df = 9 (P = 0.36); I² = 9%

Test for overall effect: Z = 8.32 (P < 0.00001)

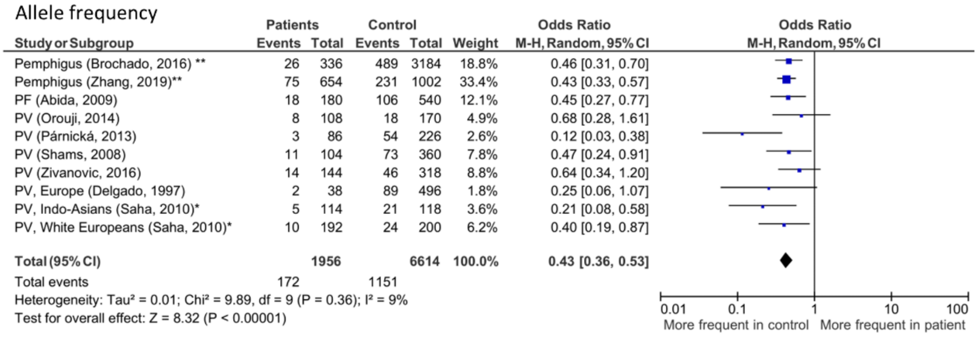

Figure S 63: Allele and genotype frequency of HLA-DQB1*06 in pemphigus

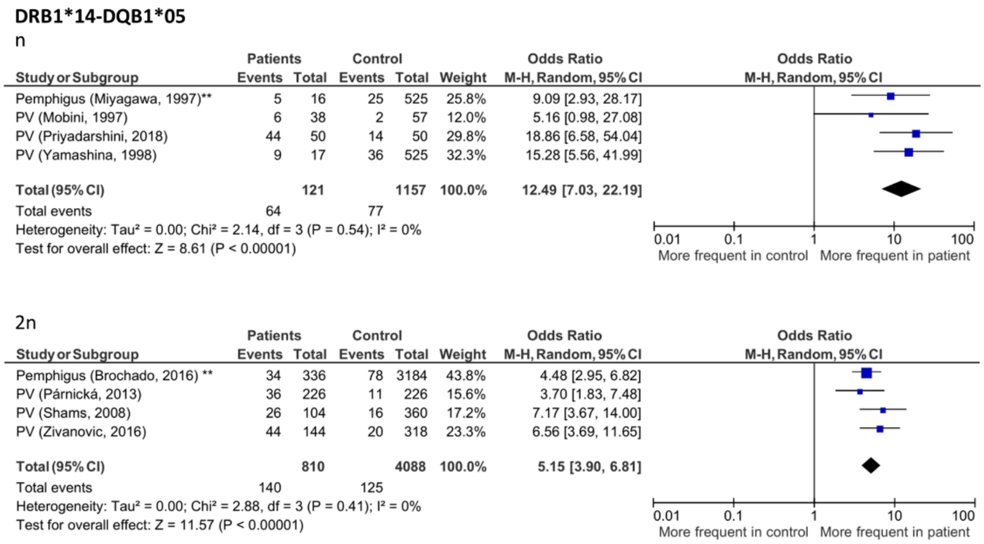

Figure S 64: Allele and genotype frequency of HLA-DRB1*14-DQB1*05 in pemphigus

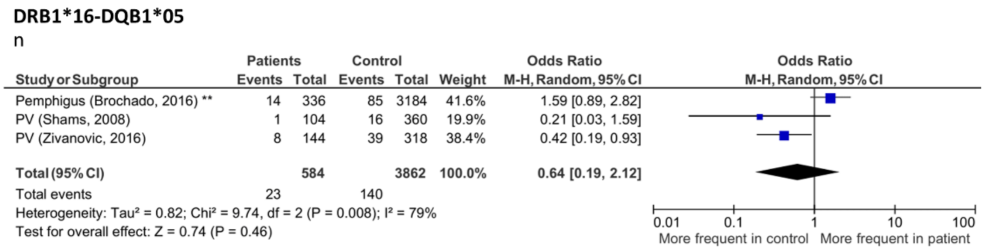

Figure S 65: Allele frequency of HLA-DRB1*16-DQB1*05 in pemphigus

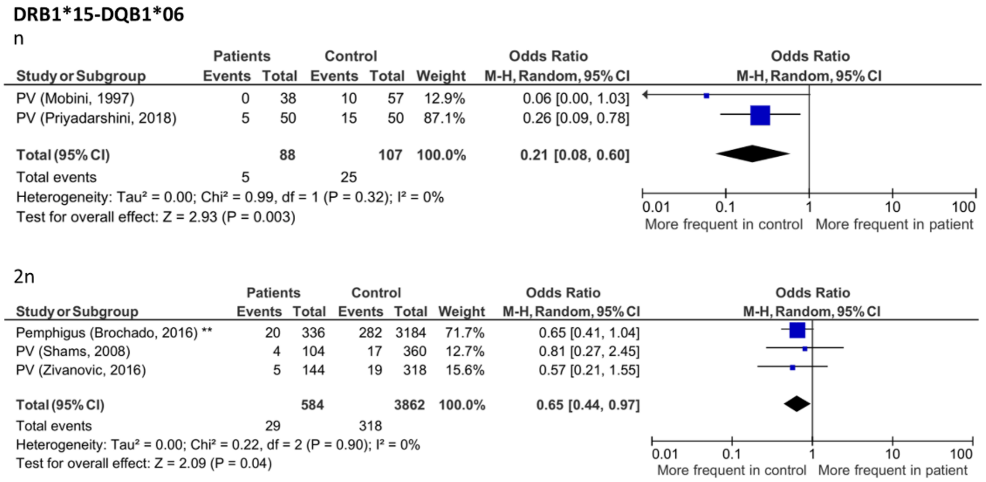

Figure S 66: Allele and genotype frequency of HLA-DRB1*15-DQB1*06 in pemphigus

DRB1*01

Genotype frequency

Study or Subgroup	Patients Events	Patients Total	Control Events	Control Total	Odds Ratio M-H, Random, 95% CI
TTP (Al Haddad, 2019)	8	30	2	30	5.09 [0.98, 26.43]
TTP (Coppo, 2010)	11	61	32	172	0.96 [0.45, 2.05]
TTP (John, 2011)	7	54	2395	11407	0.56 [0.25, 1.24]
TTP (Scully, 2010)	9	50	38	200	0.94 [0.42, 2.09]
TTP (Sinkovits, 2017)	8	75	38	204	0.52 [0.23, 1.18]

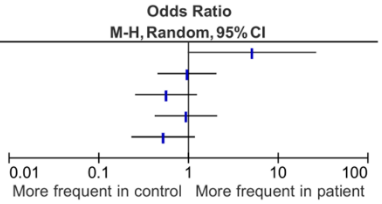

Allele frequency

Study or Subgroup	Patients Events	Patients Total	Control Events	Control Total	Odds Ratio M-H, Random, 95% CI
TTP (Sakai, 2020)	6	104	40	1046	1.54 [0.64, 3.72]

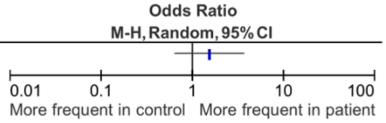

Figure S 67: Allele and genotype frequency of HLA-DRB1*01 in thrombotic thrombocytopenic purpura

DRB1*03

Genotype frequency

Study or Subgroup	Patients Events	Patients Total	Control Events	Control Total	Odds Ratio M-H, Random, 95% CI
TTP (Al Haddad, 2019)	18	30	8	30	4.13 [1.39, 12.27]
TTP (Coppo, 2010)	10	61	32	172	0.86 [0.39, 1.87]
TTP (John, 2011)	15	54	2281	11407	1.54 [0.85, 2.80]
TTP (Scully, 2010)	11	50	50	200	0.85 [0.40, 1.78]
TTP (Sinkovits, 2017)	18	75	41	204	1.26 [0.67, 2.36]

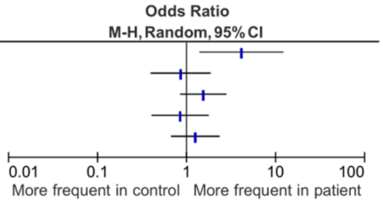

Figure S 68: Allele frequency of HLA-DRB1*03 in thrombotic thrombocytopenic purpura

DRB1*04

Genotype frequency

Study or Subgroup	Patients Events	Patients Total	Control Events	Control Total	Weight	Odds Ratio M-H, Random, 95% CI
TTP (Al Haddad, 2019)	10	30	16	30	14.5%	0.44 [0.15, 1.24]
TTP (Coppo, 2010)	6	61	48	172	19.3%	0.28 [0.11, 0.70]
TTP (John, 2011)	7	54	2395	11407	25.0%	0.56 [0.25, 1.24]
TTP (Scully, 2010)	5	50	70	200	16.9%	0.21 [0.08, 0.54]
TTP (Sinkovits, 2017)	8	75	43	204	24.3%	0.45 [0.20, 1.00]
**Total (95% CI)**		**270**		**12013**	**100.0%**	**0.38 [0.25, 0.56]**
Total events	36		2572			

Heterogeneity: Tau² = 0.00; Chi² = 3.10, df = 4 (P = 0.54); I² = 0%

Test for overall effect: Z = 4.78 (P < 0.00001)

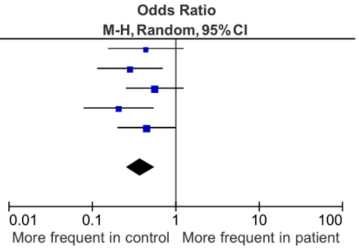

Allele frequency

Study or Subgroup	Patients Events	Patients Total	Control Events	Control Total	Odds Ratio M-H, Random, 95% CI
TTP (Sakai, 2020)	23	104	248	1046	0.91 [0.56, 1.48]
TTP, African patients (Martino, 2016)	1	52	36	1326	0.70 [0.09, 5.23]
TTP, Caribbean patients (Martino, 2016)	2	48	22	200	0.35 [0.08, 1.55]

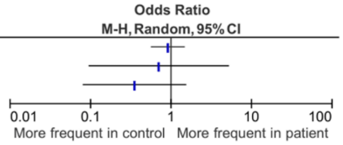

Figure S 69: Allele and genotype frequency of HLA-DRB1*04 in thrombotic thrombocytopenic purpura

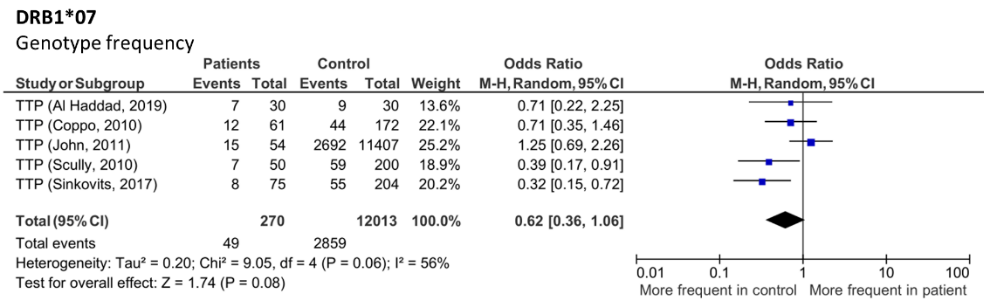

Figure S 70: Allele frequency of HLA-DRB1*07 in thrombotic thrombocytopenic purpura

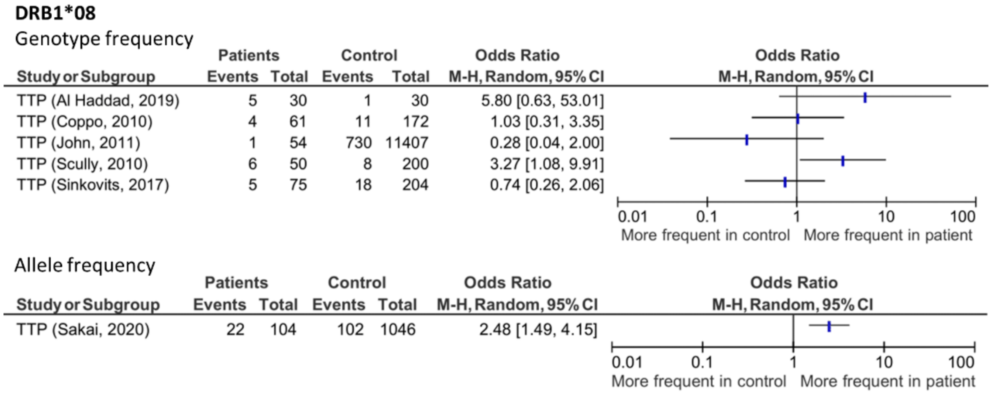

Figure S 71: Allele and genotype frequency of HLA-DRB1*08 in thrombotic thrombocytopenic purpura

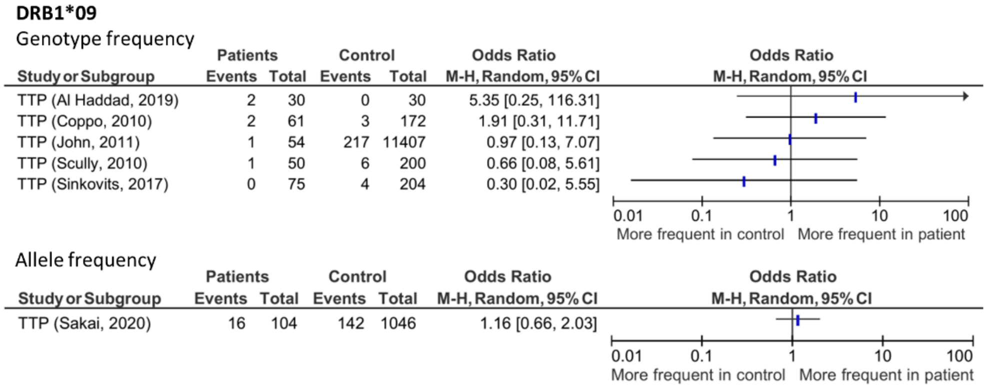

Figure S 72: Allele and genotype frequency of HLA-DRB1*09 in thrombotic thrombocytopenic purpura

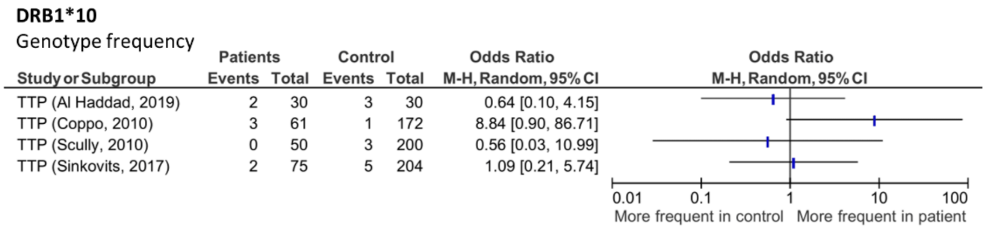

Figure S 73: Allele frequency of HLA-DRB1*10 in thrombotic thrombocytopenic purpura

DRB1*11

Genotype frequency

Study or Subgroup	Patient Events	Patient Total	Control Events	Control Total	Weight	Odds Ratio M-H, Random, 95% CI
TTP (Al Haddad, 2019)	23	30	25	30	10.6%	0.66 [0.18, 2.36]
TTP (Coppo, 2010)	38	61	39	172	21.7%	5.63 [3.00, 10.57]
TTP (John, 2011)	26	54	2681	11407	24.0%	3.02 [1.77, 5.16]
TTP (Scully, 2010)	22	50	24	200	20.1%	5.76 [2.85, 11.63]
TTP (Sinkovits, 2017)	42	75	59	204	23.7%	3.13 [1.81, 5.41]
**Total (95% CI)**		**270**		**12013**	**100.0%**	**3.38 [2.04, 5.60]**
Total events	151		2828			

Heterogeneity: Tau² = 0.20; Chi² = 11.14, df = 4 (P = 0.03); I² = 64%

Test for overall effect: Z = 4.72 (P < 0.00001)

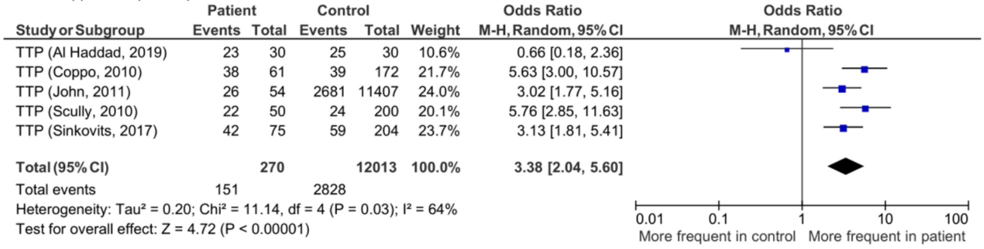

Allele frequency

Study or Subgroup	Patient Events	Patient Total	Control Events	Control Total	Odds Ratio M-H, Random, 95% CI
TTP (Sakai, 2020)	5	104	38	1046	1.34 [0.52, 3.48]
TTP, African patients (Martino, 2016)	6	52	201	1326	0.73 [0.31, 1.73]
TTP, Caribbean patients (Martino, 2016)	6	48	32	200	0.75 [0.29, 1.91]

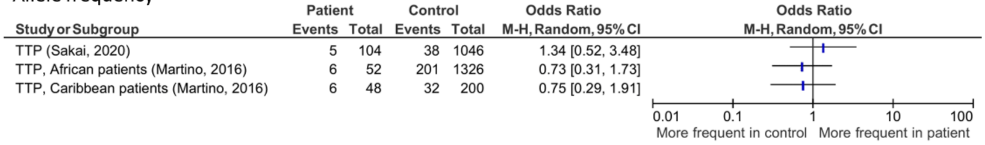

Figure S 74: Allele and genotype frequency of HLA-DRB1*11 in thrombotic thrombocytopenic purpura

DRB1*12

Genotype frequency

Study or Subgroup	Patients Events	Patients Total	Control Events	Control Total	Weight	Odds Ratio M-H, Random, 95% CI
TTP (Al Haddad, 2019)	3	30	0	30	4.7%	7.76 [0.38, 157.14]
TTP (Coppo, 2010)	2	61	2	172	10.8%	2.88 [0.40, 20.92]
TTP (John, 2011)	2	54	422	11407	21.1%	1.00 [0.24, 4.12]
TTP (Scully, 2010)	5	50	4	200	23.1%	5.44 [1.41, 21.09]
TTP (Sinkovits, 2017)	7	75	9	204	40.3%	2.23 [0.80, 6.22]
**Total (95% CI)**		**270**		**12013**	**100.0%**	**2.52 [1.32, 4.84]**
Total events	19		437			

Heterogeneity: Tau² = 0.00; Chi² = 3.49, df = 4 (P = 0.48); I² = 0%

Test for overall effect: Z = 2.79 (P = 0.005)

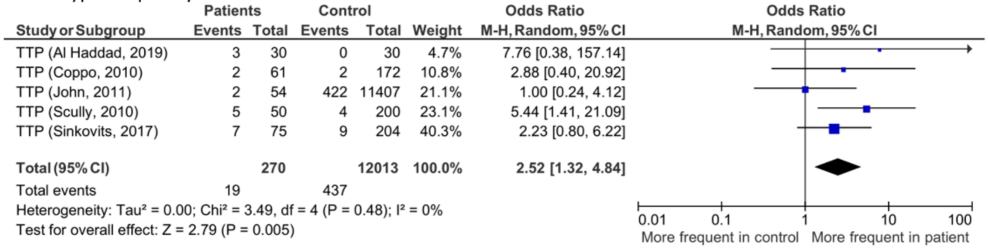

Allele frequency

Study or Subgroup	Patients Events	Patients Total	Control Events	Control Total	Odds Ratio M-H, Random, 95% CI
TTP (Sakai, 2020)	7	104	48	1046	1.50 [0.66, 3.41]

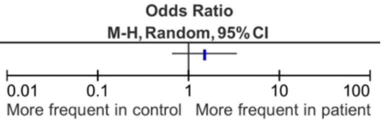

Figure S 75: Allele and genotype frequency of HLA-DRB1*12 in thrombotic thrombocytopenic purpura

DRB1*13

Genotype frequency

Study or Subgroup	Patients Events	Patients Total	Control Events	Control Total	Weight	Odds Ratio M-H, Random, 95% CI
TTP (Al Haddad, 2019)	8	30	12	30	13.0%	0.55 [0.18, 1.62]
TTP (Coppo, 2010)	9	61	55	172	25.5%	0.37 [0.17, 0.80]
TTP (John, 2011)	7	54	2749	11407	24.4%	0.47 [0.21, 1.04]
TTP (Scully, 2010)	6	50	43	200	18.3%	0.50 [0.20, 1.25]
TTP (Sinkovits, 2017)	6	75	39	204	18.8%	0.37 [0.15, 0.91]
**Total (95% CI)**		**270**		**12013**	**100.0%**	**0.43 [0.29, 0.64]**
Total events	36		2898			

Heterogeneity: Tau² = 0.00; Chi² = 0.59, df = 4 (P = 0.96); I² = 0%

Test for overall effect: Z = 4.17 (P < 0.0001)

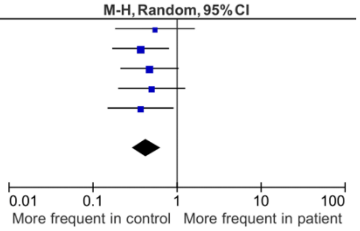

Allele frequency

Study or Subgroup	Patients Events	Patients Total	Control Events	Control Total	Odds Ratio M-H, Random, 95% CI
TTP (Sakai, 2020)	3	104	58	1046	0.51 [0.16, 1.64]

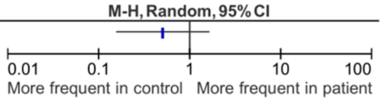

Figure S 76: Allele and genotype frequency of HLA-DRB1*13 in thrombotic thrombocytopenic purpura

DRB1*14

Genotype frequency

Study or Subgroup	Patient Events	Patient Total	Control Events	Control Total	Odds Ratio M-H, Random, 95% CI
TTP (Al Haddad, 2019)	14	30	0	30	53.61 [3.00, 956.98]
TTP (Coppo, 2010)	2	61	10	172	0.55 [0.12, 2.58]
TTP (John, 2011)	2	54	696	11407	0.59 [0.14, 2.44]
TTP (Scully, 2010)	2	50	7	200	1.15 [0.23, 5.71]
TTP (Sinkovits, 2017)	10	75	12	204	2.46 [1.02, 5.96]

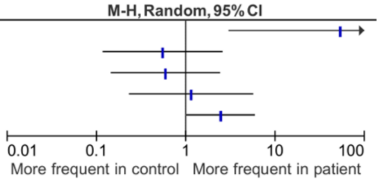

Allele frequency

Study or Subgroup	Patient Events	Patient Total	Control Events	Control Total	Odds Ratio M-H, Random, 95% CI
TTP (Sakai, 2020)	3	104	98	1046	0.29 [0.09, 0.92]

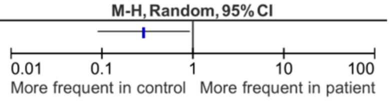

Figure S 77: Allele and genotype frequency of HLA-DRB1*14 in thrombotic thrombocytopenic purpura

DRB1*15

Genotype frequency

Study or Subgroup	Patient Events	Patient Total	Control Events	Control Total	Weight	Odds Ratio M-H, Random, 95% CI
TTP (Al Haddad, 2019)	3	30	0	30	1.8%	7.76 [0.38, 157.14]
TTP (Coppo, 2010)	16	61	33	172	31.3%	1.50 [0.75, 2.97]
TTP (Scully, 2010)	15	50	54	200	31.6%	1.16 [0.59, 2.29]
TTP (Sinkovits, 2017)	21	75	29	204	35.3%	2.35 [1.24, 4.45]
**Total (95% CI)**		**216**		**606**	**100.0%**	**1.67 [1.11, 2.51]**
Total events	55		116			

Heterogeneity: Tau² = 0.02; Chi² = 3.30, df = 3 (P = 0.35); I² = 9%

Test for overall effect: Z = 2.45 (P = 0.01)

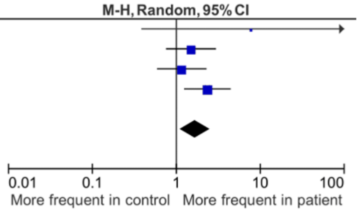

Allele frequency

Study or Subgroup	Patient Events	Patient Total	Control Events	Control Total	Odds Ratio M-H, Random, 95% CI
TTP (Sakai, 2020)	16	104	212	1046	0.72 [0.41, 1.24]

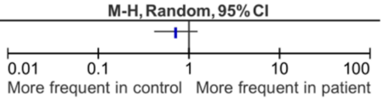

Figure S 78: Allele and genotype frequency of HLA-DRB1*15 in thrombotic thrombocytopenic purpura

DRB1*16

Genotype frequency

Study or Subgroup	Patients Events	Patients Total	Control Events	Control Total	Odds Ratio M-H, Random, 95% CI
TTP (Al Haddad, 2019)	10	30	8	30	1.38 [0.45, 4.17]
TTP (Coppo, 2010)	3	61	11	172	0.76 [0.20, 2.81]
TTP (Scully, 2010)	3	50	5	200	2.49 [0.57, 10.79]
TTP (Sinkovits, 2017)	7	75	26	204	0.70 [0.29, 1.70]

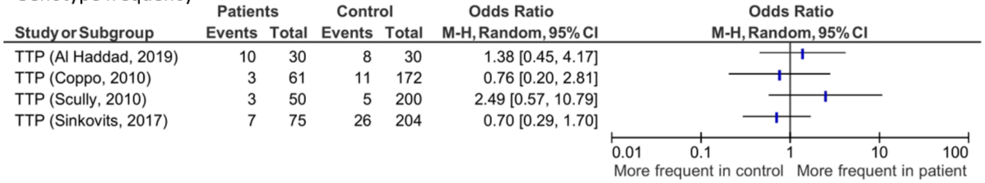

Figure S 79: Allele frequency of HLA-DRB1*16 in thrombotic thrombocytopenic purpura

DQB1*02

Genotype frequency

Study or Subgroup	Patients Events	Patients Total	Control Events	Control Total	Odds Ratio M-H, Random, 95% CI
TTP (Al Haddad, 2019)	14	30	2	30	12.25 [2.46, 60.91]
TTP (Coppo, 2010)	19	60	68	172	0.71 [0.38, 1.32]
TTP (John, 2011)	26	50	19	174	8.84 [4.25, 18.36]
TTP (Scully, 2010)	16	50	86	200	0.62 [0.32, 1.20]

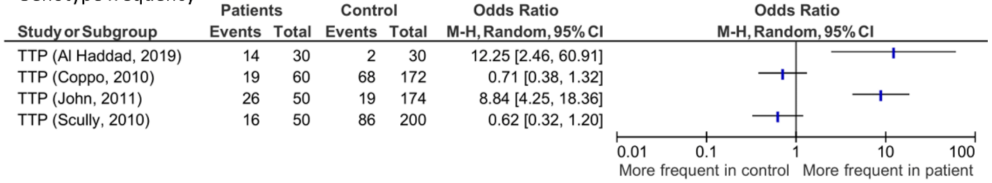

Figure S 80: Allele frequency of HLA-DQB1*02 in thrombotic thrombocytopenic purpura

DQB1*03

Genotype frequency

Study or Subgroup	Patients Events	Patients Total	Control Events	Control Total	Weight	Odds Ratio M-H, Random, 95% CI
TTP (Al Haddad, 2019)	28	30	26	30	13.8%	2.15 [0.36, 12.76]
TTP (Coppo, 2010)	46	60	92	172	29.0%	2.86 [1.46, 5.58]
TTP (John, 2011)	29	47	29	174	28.4%	8.06 [3.96, 16.39]
TTP (Scully, 2010)	36	50	129	200	28.8%	1.42 [0.72, 2.80]
**Total (95% CI)**		**187**		**576**	**100.0%**	**3.01 [1.30, 7.00]**
Total events	139		276			

Heterogeneity: Tau² = 0.52; Chi² = 12.27, df = 3 (P = 0.007); I² = 76%

Test for overall effect: Z = 2.56 (P = 0.01)

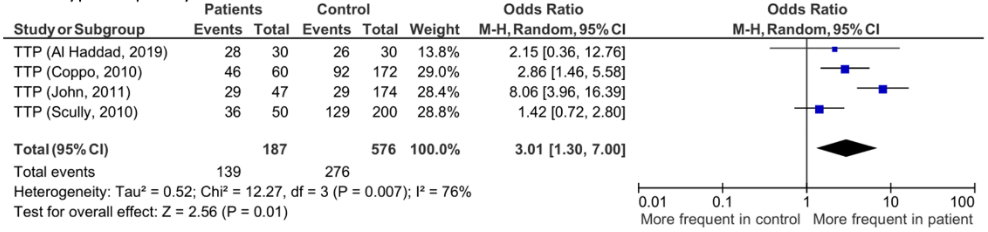

Allele frequency

Study or Subgroup	Patients Events	Patients Total	Control Events	Control Total	Odds Ratio M-H, Random, 95% CI
TTP (Sakai, 2020)	37	104	378	1046	0.98 [0.64, 1.49]
TTP, African patients (Martino, 2016)	17	52	82	874	4.69 [2.52, 8.74]
TTP, Caribbean patients (Martino, 2016)	12	48	50	200	1.00 [0.48, 2.07]

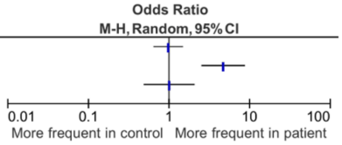

Figure S 81: Allele and genotype frequency of HLA-DQB1*03 in thrombotic thrombocytopenic purpura

DQB1*04

Genotype frequency

Study or Subgroup	Patients Events	Patients Total	Control Events	Control Total	Odds Ratio M-H, Random, 95% CI
TTP (Al Haddad, 2019)	0	30	1	30	0.32 [0.01, 8.24]
TTP (Coppo, 2010)	3	60	7	172	1.24 [0.31, 4.96]
TTP (John, 2011)	1	54	3	174	1.08 [0.11, 10.56]
TTP (Scully, 2010)	6	50	5	200	5.32 [1.55, 18.21]

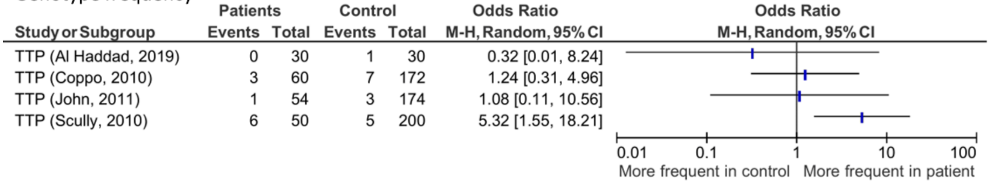

Allele frequency

Study or Subgroup	Patients Events	Patients Total	Control Events	Control Total	Odds Ratio M-H, Random, 95% CI
TTP (Sakai, 2020)	19	104	174	1046	1.12 [0.66, 1.89]

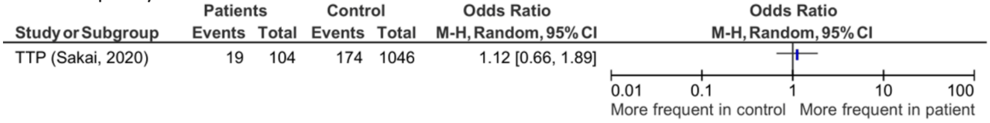

Figure S 82: Allele and genotype frequency of HLA-DQB1*04 in thrombotic thrombocytopenic purpura

DQB1*05

Genotype frequency

Study or Subgroup	Patient Events	Patient Total	Control Events	Control Total	Odds Ratio M-H, Random, 95% CI
TTP (Al Haddad, 2019)	0	30	3	30	0.13 [0.01, 2.61]
TTP (Coppo, 2010)	19	60	55	172	0.99 [0.52, 1.85]
TTP (John, 2011)	12	54	20	174	2.20 [1.00, 4.86]
TTP (Scully, 2010)	13	50	51	200	1.03 [0.51, 2.08]

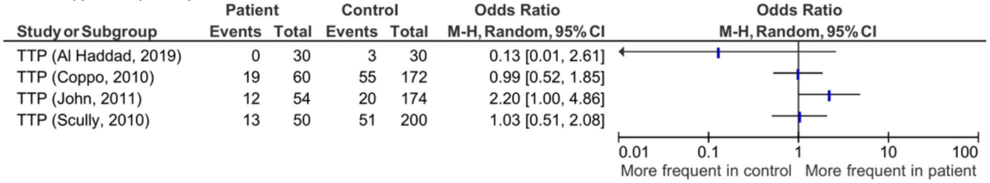

Allele frequency

Study or Subgroup	Patient Events	Patient Total	Control Events	Control Total	Odds Ratio M-H, Random, 95% CI
TTP (Sakai, 2020)	11	104	136	1046	0.79 [0.41, 1.52]

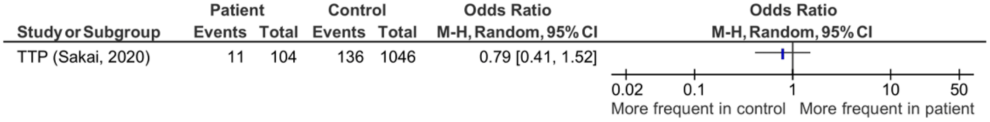

Figure S 83: Allele and genotype frequency of HLA-DQB1*05 in thrombotic thrombocytopenic purpura

DQB1*06

Genotype frequency

Study or Subgroup	Patients Events	Patients Total	Control Events	Control Total	Odds Ratio M-H, Random, 95% CI
TTP (Al Haddad, 2019)	2	30	3	30	0.64 [0.10, 4.15]
TTP (Coppo, 2010)	22	60	74	172	0.77 [0.42, 1.40]
TTP (John, 2011)	15	52	28	174	2.11 [1.03, 4.36]
TTP (Scully, 2010)	19	50	92	200	0.72 [0.38, 1.36]

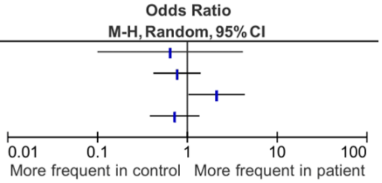

Allele frequency

Study or Subgroup	Patients Events	Patients Total	Control Events	Control Total	Odds Ratio M-H, Random, 95% CI
TTP (Sakai, 2020)	37	104	330	1046	1.20 [0.79, 1.83]

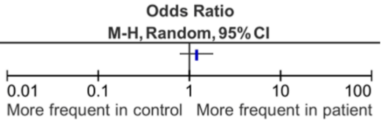

Figure S 84: Allele and genotype frequency of HLA-DQB1*06 in thrombotic thrombocytopenic purpura

DRB1*01

Genotype frequency

Study or Subgroup	Patients Events	Patients Total	Control Events	Control Total	Odds Ratio M-H, Random, 95% CI
MuSK MG (Kanai, 2016)	1	14	15	100	0.44 [0.05, 3.58]
MuSK MG (Nikolic, 2014)	3	31	386	1992	0.45 [0.13, 1.47]
MuSK MG (Niks, 2006)	9	23	14	2440	111.40 [41.45, 299.36]

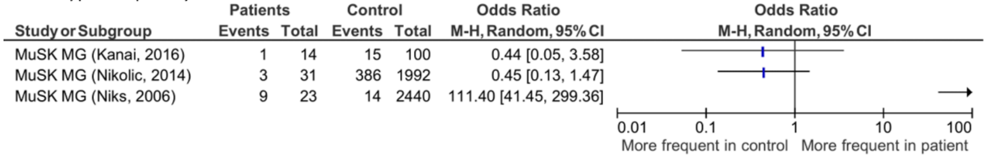

Allele frequency

Study or Subgroup	Patients Events	Patients Total	Control Events	Control Total	Odds Ratio M-H, Random, 95% CI
MuSK MG (Ehsan, 2015)	4	48	22	400	1.56 [0.51, 4.74]
MuSK MG (Nikolic, 2014)	2	62	410	3984	0.29 [0.07, 1.19]

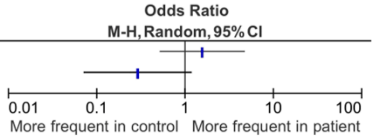

Figure S 85: Allele and genotype frequency of HLA-DRB1*01 in MuSK myasthenia gravis

DRB1*03

Genotype frequency

Study or Subgroup	Patients Events	Patients Total	Control Events	Control Total	Odds Ratio M-H, Random, 95% CI
MuSK MG (Alahgholi-Hajibehzad, 2013)	1	48	44	250	0.10 [0.01, 0.74]
MuSK MG (Nikolic, 2014)	5	31	418	1992	0.72 [0.28, 1.90]
MuSK MG (Niks, 2006)	2	23	21	2440	10.97 [2.42, 49.79]

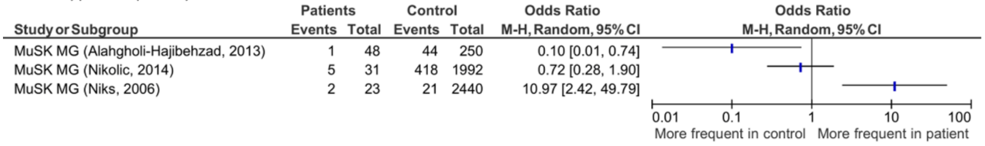

Allele frequency

Study or Subgroup	Patients Events	Patients Total	Control Events	Control Total	Odds Ratio M-H, Random, 95% CI
MuSK MG (Ehsan, 2015)	1	48	40	400	0.19 [0.03, 1.43]
MuSK MG (Nikolic, 2014)	5	62	433	3984	0.72 [0.29, 1.80]

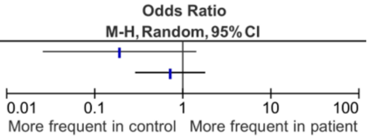

Figure S 86: Allele and genotype frequency of HLA-DRB1*03 in MuSK myasthenia gravis

DRB1*04

Genotype frequency

Study or Subgroup	Patients Events	Patients Total	Control Events	Control Total	Odds Ratio M-H, Random, 95% CI
MuSK MG (Kanai, 2016)	4	14	44	100	0.51 [0.15, 1.73]
MuSK MG (Nikolic, 2014)	2	31	339	1992	0.34 [0.08, 1.42]

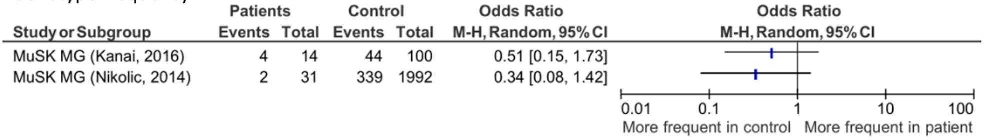

Allele frequency

Study or Subgroup	Patients Events	Patients Total	Control Events	Control Total	Odds Ratio M-H, Random, 95% CI
MuSK MG (Ehsan, 2015)	2	48	42	400	0.37 [0.09, 1.58]
MuSK MG (Nikolic, 2014)	2	62	358	3984	0.34 [0.08, 1.39]

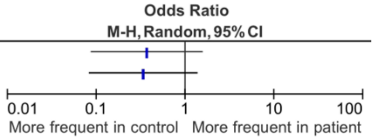

Figure S 87: Allele and genotype frequency of HLA-DRB1*04 in MuSK myasthenia gravis

DRB1*07

Genotype frequency

Study or Subgroup	Patients Events	Patients Total	Control Events	Control Total	Odds Ratio M-H, Random, 95% CI
MuSK MG (Nikolic, 2014)	7	31	276	1992	1.81 [0.77, 4.25]

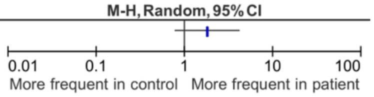

Allele frequency

Study or Subgroup	Patients Events	Patients Total	Control Events	Control Total	Odds Ratio M-H, Random, 95% CI
MuSK MG (Ehsan, 2015)	5	48	26	400	1.67 [0.61, 4.58]
MuSK MG (Nikolic, 2014)	7	62	284	3984	1.66 [0.75, 3.67]

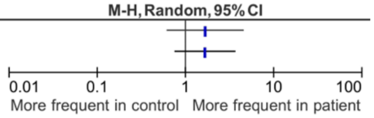

Figure S 88: Allele and genotype frequency of HLA-DRB1*07 in MuSK myasthenia gravis

DRB1*08

Genotype frequency

Study or Subgroup	Patients Events	Patients Total	Control Events	Control Total	Odds Ratio M-H, Random, 95% CI
MuSK MG (Kanai, 2016)	0	14	28	100	0.09 [0.01, 1.52]
MuSK MG (Nikolic, 2014)	3	31	123	1992	1.63 [0.49, 5.43]

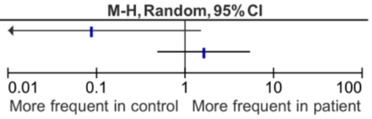

Allele frequency

Study or Subgroup	Patients Events	Patients Total	Control Events	Control Total	Odds Ratio M-H, Random, 95% CI
MuSK MG (Ehsan, 2015)	0	48	6	400	0.63 [0.03, 11.28]
MuSK MG (Nikolic, 2014)	3	62	123	3984	1.60 [0.49, 5.16]

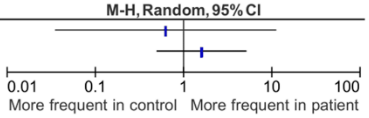

Figure S 89: Allele and genotype frequency of HLA-DRB1*08 in MuSK myasthenia gravis

DRB1*09

Genotype frequency

Study or Subgroup	Patients Events	Patients Total	Control Events	Control Total	Odds Ratio M-H, Random, 95% CI
MuSK MG (Kanai, 2016)	3	14	23	100	0.91 [0.23, 3.55]
MuSK MG (Nikolic, 2014)	0	31	14	1992	2.17 [0.13, 37.11]

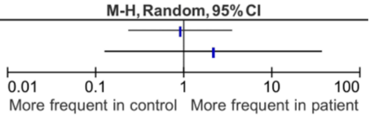

Allele frequency

Study or Subgroup	Patients Events	Patients Total	Control Events	Control Total	Odds Ratio M-H, Random, 95% CI
MuSK MG (Ehsan, 2015)	1	48	12	400	0.69 [0.09, 5.41]
MuSK MG (Nikolic, 2014)	0	62	14	3984	2.19 [0.13, 37.13]

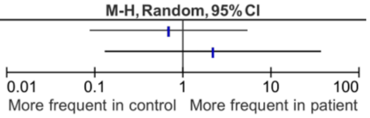

Figure S 90: Allele and genotype frequency of HLA-DRB1*09 in MuSK myasthenia gravis

DRB1*10

Genotype frequency

Study or Subgroup	Patients Events	Patients Total	Control Events	Control Total	Odds Ratio M-H, Random, 95% CI
MuSK MG (Nikolic, 2014)	0	31	44	1992	0.70 [0.04, 11.54]

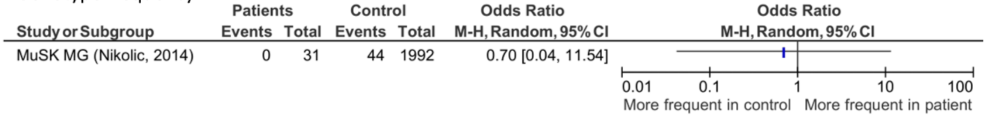

Allele frequency

Study or Subgroup	Patients Events	Patients Total	Control Events	Control Total	Odds Ratio M-H, Random, 95% CI
MuSK MG (Ehsan, 2015)	1	48	16	400	0.51 [0.07, 3.94]
MuSK MG (Nikolic, 2014)	0	62	44	3984	0.71 [0.04, 11.63]

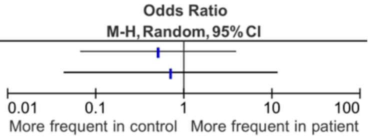

Figure S 91: Allele and genotype frequency of HLA-DRB1*10 in MuSK myasthenia gravis

DRB1*11

Genotype frequency

Study or Subgroup	Patient Events	Patient Total	Control Events	Control Total	Odds Ratio M-H, Random, 95% CI
MuSK MG (Kanai, 2016)	2	14	5	100	3.17 [0.55, 18.16]
MuSK MG (Nikolic, 2014)	7	31	617	1992	0.65 [0.28, 1.52]

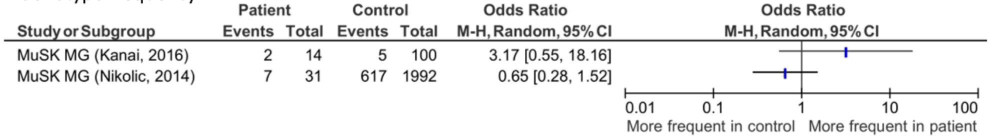

Allele frequency

Study or Subgroup	Patient Events	Patient Total	Control Events	Control Total	Odds Ratio M-H, Random, 95% CI
MuSK MG (Ehsan, 2015)	5	48	100	400	0.35 [0.13, 0.91]
MuSK MG (Nikolic, 2014)	7	62	637	3984	0.67 [0.30, 1.48]

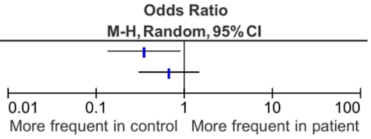

Figure S 92: Allele and genotype frequency of HLA-DRB1*11 in MuSK myasthenia gravis

DRB1*12

Genotype frequency

Study or Subgroup	Patients Events	Patients Total	Control Events	Control Total	Odds Ratio M-H, Random, 95% CI
MuSK MG (Kanai, 2016)	1	14	7	100	1.02 [0.12, 8.99]
MuSK MG (Nikolic, 2014)	2	31	76	1992	1.74 [0.41, 7.42]

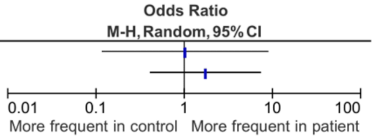

Allele frequency

Study or Subgroup	Patients Events	Patients Total	Control Events	Control Total	Odds Ratio M-H, Random, 95% CI
MuSK MG (Ehsan, 2015)	1	48	6	400	1.40 [0.16, 11.86]
MuSK MG (Nikolic, 2014)	2	62	76	3984	1.71 [0.41, 7.14]

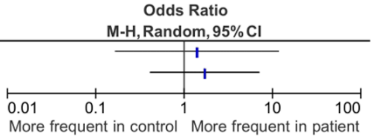

Figure S 93: Allele and genotype frequency of HLA-DRB1*12 in MuSK myasthenia gravis

DRB1*13

Genotype frequency

Study or Subgroup	Patients Events	Patients Total	Control Events	Control Total	Odds Ratio M-H, Random, 95% CI
MuSK MG (Kanai, 2016)	0	14	11	100	0.27 [0.01, 4.81]
MuSK MG (Nikolic, 2014)	0	31	495	1992	0.05 [0.00, 0.79]

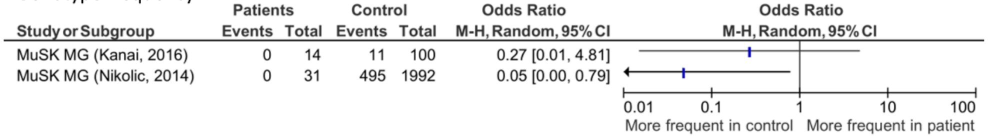

Allele frequency

Study or Subgroup	Patients Events	Patients Total	Control Events	Control Total	Odds Ratio M-H, Random, 95% CI
MuSK MG (Ehsan, 2015)	2	48	34	400	0.47 [0.11, 2.01]
MuSK MG (Nikolic, 2014)	0	62	527	3984	0.05 [0.00, 0.85]

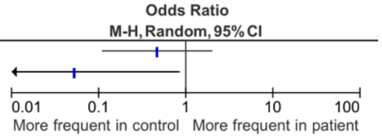

Figure S 94: Allele and genotype frequency of HLA-DRB1*13 in MuSK myasthenia gravis

DRB1*14

Genotype frequency

Study or Subgroup	Patient Events	Patient Total	Control Events	Control Total	Weight	Odds Ratio M-H, Random, 95% CI
MuSK MG (Alahgholi-Hajibehzad, 2013)	14	48	29	250	27.1%	3.14 [1.51, 6.53]
MuSK MG (Kanai, 2016)	8	14	18	100	20.5%	6.07 [1.88, 19.67]
MuSK MG (Nikolic, 2014)	11	31	214	1992	26.9%	4.57 [2.16, 9.67]
MuSK MG (Niks, 2006)	12	23	127	2440	25.5%	19.87 [8.60, 45.90]
**Total (95% CI)**		**116**		**4782**	**100.0%**	**6.36 [2.75, 14.75]**
Total events	45		388			

Heterogeneity: $Tau^2 = 0.54$; $Chi^2 = 11.69$, $df = 3$ ($P = 0.009$); $I^2 = 74\%$

Test for overall effect: $Z = 4.32$ ($P < 0.0001$)

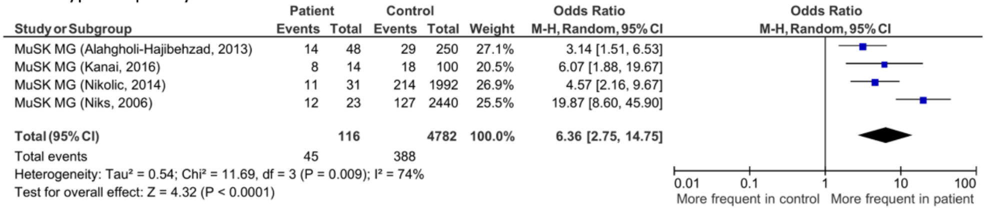

Allele frequency

Study or Subgroup	Patient Events	Patient Total	Control Events	Control Total	Odds Ratio M-H, Random, 95% CI
MuSK MG (Ehsan, 2015)	9	48	22	400	3.97 [1.71, 9.21]
MuSK MG (Nikolic, 2014)	11	62	216	3984	3.76 [1.93, 7.32]

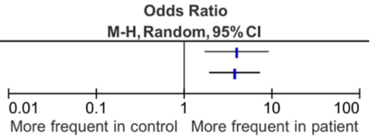

Figure S 95: Allele and genotype frequency of HLA-DRB1*14 in MuSK myasthenia gravis

DRB1*15

Genotype frequency

Study or Subgroup	Patient Events	Patient Total	Control Events	Control Total	Odds Ratio M-H, Random, 95% CI
MuSK MG (Kanai, 2016)	6	14	32	100	1.59 [0.51, 4.98]
MuSK MG (Nikolic, 2014)	4	31	371	1992	0.65 [0.23, 1.86]

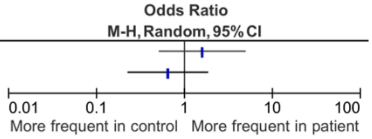

Allele frequency

Study or Subgroup	Patient Events	Patient Total	Control Events	Control Total	Odds Ratio M-H, Random, 95% CI
MuSK MG (Ehsan, 2015)	6	48	48	400	1.05 [0.42, 2.59]
MuSK MG (Nikolic, 2014)	4	62	392	3984	0.63 [0.23, 1.75]

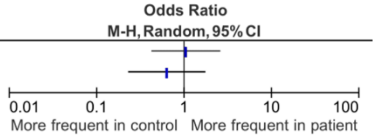

Figure S 96: Allele and genotype frequency of HLA-DRB1*15 in MuSK myasthenia gravis

DRB1*16

Genotype frequency

Study or Subgroup	Patients Events	Patients Total	Control Events	Control Total	Weight	Odds Ratio M-H, Random, 95% CI
MuSK MG (Alahgholi-Hajibehzad, 2013)	18	48	27	250	43.0%	4.96 [2.44, 10.06]
MuSK MG (Kanai, 2016)	2	14	2	100	5.1%	8.17 [1.05, 63.41]
MuSK MG (Nikolic, 2014)	17	31	406	1992	42.1%	4.74 [2.32, 9.70]
MuSK MG (Niks, 2006)	2	23	43	2440	9.8%	5.31 [1.21, 23.36]
**Total (95% CI)**		**116**		**4782**	**100.0%**	**5.03 [3.16, 7.99]**
Total events	39		478			

Heterogeneity: Tau² = 0.00; Chi² = 0.25, df = 3 (P = 0.97); I² = 0%

Test for overall effect: Z = 6.82 (P < 0.00001)

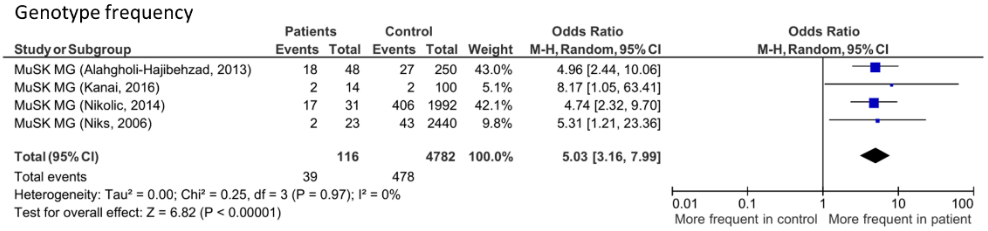

Allele frequency

Study or Subgroup	Patients Events	Patients Total	Control Events	Control Total	Odds Ratio M-H, Random, 95% CI
MuSK MG (Nikolic, 2014)	18	62	433	3984	3.35 [1.92, 5.86]

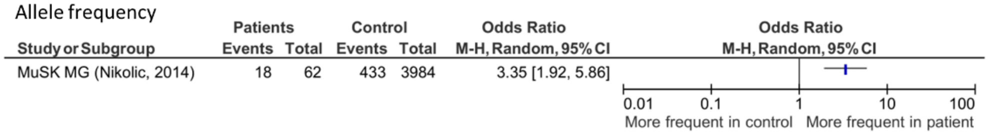

Figure S 97: Allele and genotype frequency of HLA-DRB1*16 in MuSK myasthenia gravis

DQB1*02

Genotype frequency

Study or Subgroup	Patients Events	Patients Total	Control Events	Control Total	Odds Ratio M-H, Random, 95% CI
MuSK MG (Nikolic, 2014)	12	31	48	159	1.46 [0.66, 3.24]

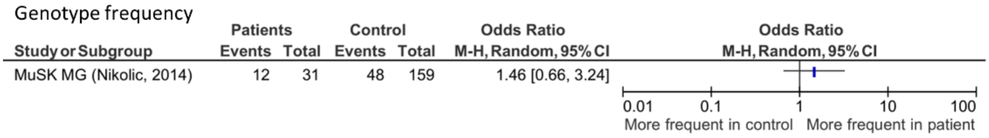

Allele frequency

Study or Subgroup	Patients Events	Patients Total	Control Events	Control Total	Odds Ratio M-H, Random, 95% CI
MuSK MG (Ehsan, 2015)	8	48	76	400	0.85 [0.38, 1.90]
MuSK MG (Nikolic, 2014)	12	62	54	318	1.17 [0.59, 2.35]

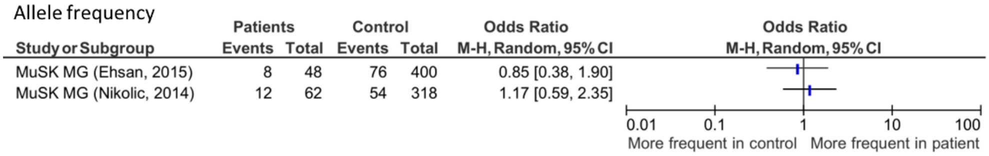

Figure S 98: Allele and genotype frequency of HLA-DQB1*02 in MuSK myasthenia gravis

DQB1*03

Genotype frequency

Study or Subgroup	Patients Events	Patients Total	Control Events	Control Total	Odds Ratio M-H, Random, 95% CI
MuSK MG (Kanai, 2016)	9	14	51	100	1.73 [0.54, 5.52]
MuSK MG (Nikolic, 2014)	9	21	87	159	0.62 [0.25, 1.56]

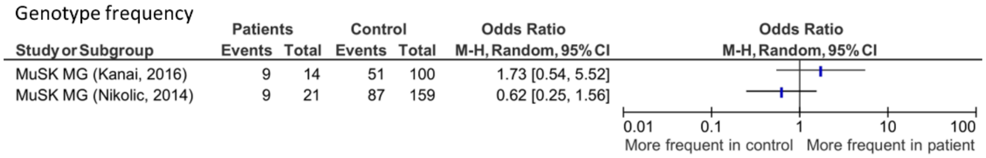

Allele frequency

Study or Subgroup	Patients Events	Patients Total	Control Events	Control Total	Odds Ratio M-H, Random, 95% CI
MuSK MG (Ehsan, 2015)	8	48	54	400	1.28 [0.57, 2.88]
MuSK MG (Nikolic, 2014)	11	62	106	318	0.43 [0.22, 0.86]

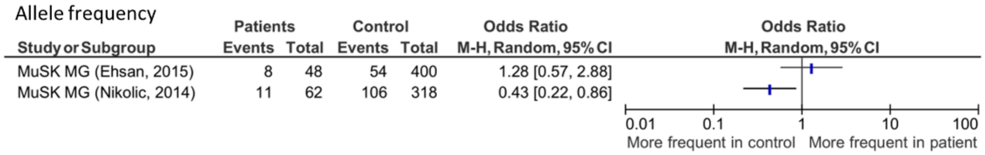

Figure S 99: Allele and genotype frequency of HLA-DQB1*03 in MuSK myasthenia gravis

DQB1*04

Genotype frequency

Study or Subgroup	Patients Events	Patients Total	Control Events	Control Total	Odds Ratio M-H, Random, 95% CI
MuSK MG (Kanai, 2016)	2	14	37	100	0.28 [0.06, 1.34]
MuSK MG (Nikolic, 2014)	3	31	28	159	0.50 [0.14, 1.76]

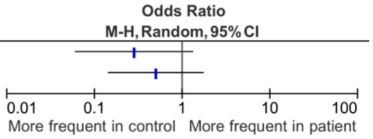

Allele frequency

Study or Subgroup	Patients Events	Patients Total	Control Events	Control Total	Odds Ratio M-H, Random, 95% CI
MuSK MG (Nikolic, 2014)	3	62	7	318	2.26 [0.57, 8.99]

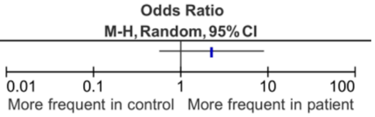

Figure S 100: Allele and genotype frequency of HLA-DQB1*04 in MuSK myasthenia gravis

DQB1*05

Genotype frequency

Study or Subgroup	Patient Events	Patient Total	Control Events	Control Total	Weight	Odds Ratio M-H, Random, 95% CI
MuSK MG (Alahgholi-Hajibehzad, 2013)	35	48	90	250	30.8%	4.79 [2.41, 9.51]
MuSK MG (Kanai, 2016)	11	14	30	100	19.3%	8.56 [2.23, 32.88]
MuSK MG (Nikolic, 2014)	26	31	87	159	24.9%	4.30 [1.57, 11.78]
MuSK MG (Niks, 2006)	18	23	300	2440	25.0%	25.68 [9.46, 69.68]
**Total (95% CI)**		**116**		**2949**	**100.0%**	**7.94 [3.44, 18.30]**
Total events	90		507			

Heterogeneity: Tau² = 0.47; Chi² = 8.75, df = 3 (P = 0.03); I² = 66%

Test for overall effect: Z = 4.86 (P < 0.00001)

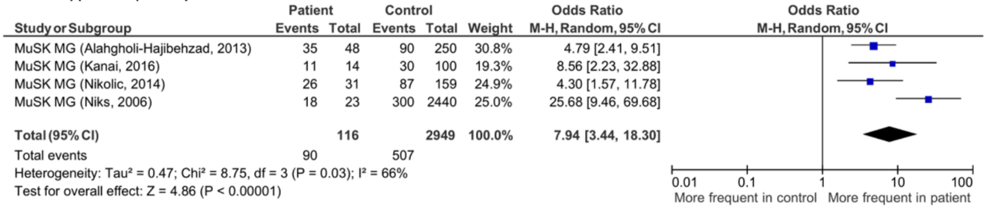

Allele frequency

Study or Subgroup	Patient Events	Patient Total	Control Events	Control Total	Odds Ratio M-H, Random, 95% CI
MuSK MG (Ehsan, 2015)	28	48	90	400	4.82 [2.59, 8.96]
MuSK MG (Nikolic, 2014)	32	62	105	318	2.16 [1.25, 3.75]

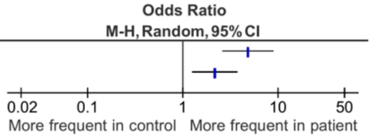

Figure S 101: Allele and genotype frequency of HLA-DQB1*05 in MuSK myasthenia gravis

DQB1*06

Genotype frequency

Study or Subgroup	Patients Events	Patients Total	Control Events	Control Total	Odds Ratio M-H, Random, 95% CI
MuSK MG (Kanai, 2016)	6	14	57	100	0.57 [0.18, 1.75]
MuSK MG (Nikolic, 2014)	4	31	43	159	0.40 [0.13, 1.21]
MuSK MG (Niks, 2006)	5	23	453	2440	1.22 [0.45, 3.30]

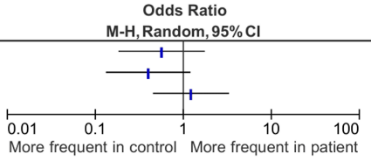

Allele frequency

Study or Subgroup	Patients Events	Patients Total	Control Events	Control Total	Odds Ratio M-H, Random, 95% CI
MuSK MG (Ehsan, 2015)	6	48	70	400	0.67 [0.28, 1.65]
MuSK MG (Nikolic, 2014)	4	62	46	318	0.41 [0.14, 1.18]

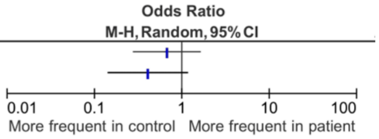

Figure S 102: Allele and genotype frequency of HLA-DRB1*06 in MuSK myasthenia gravis

DRB1*14-DQB1*05

n

Study or Subgroup	Patients Events	Patients Total	Control Events	Control Total	Odds Ratio M-H, Random, 95% CI
MuSK MG (Alahgholi-Hajibehzad, 2013)	13	48	28	250	2.94 [1.39, 6.22]
MuSK MG (Kanai, 2016)	6	14	13	100	5.02 [1.50, 16.80]

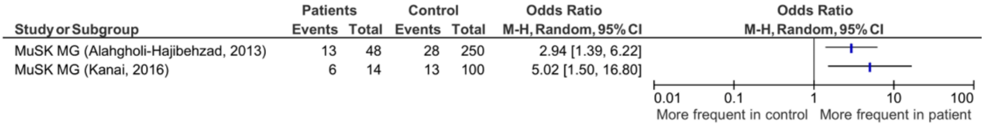

2n

Study or Subgroup	Patients Events	Patients Total	Control Events	Control Total	Weight	Odds Ratio M-H, Random, 95% CI
MuSK MG (Ehsan, 2015)	9	48	22	400	31.7%	3.97 [1.71, 9.21]
MuSK MG (Nikolic, 2014)	11	62	20	318	34.2%	3.21 [1.45, 7.11]
MuSK MG (Niks, 2006)	11	46	23	642	34.1%	8.46 [3.82, 18.73]
**Total (95% CI)**		**156**		**1360**	**100.0%**	**4.78 [2.65, 8.62]**
Total events	31		65			

Heterogeneity: Tau² = 0.10; Chi² = 3.19, df = 2 (P = 0.20); I² = 37%

Test for overall effect: Z = 5.19 (P < 0.00001)

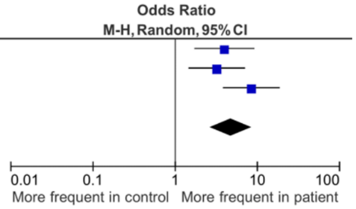

Figure S 103: Allele and genotype frequency of HLA-DRB1*14-DQB1*05 in MuSK myasthenia gravis

DRB1*16-DQB1*05

n

Study or Subgroup	Patients Events	Patients Total	Control Events	Control Total	Odds Ratio M-H, Random, 95% CI
MuSK MG (Alahgholi-Hajibehzad, 2013)	16	48	27	250	4.13 [2.01, 8.49]
MuSK MG (Kanai, 2016)	2	14	2	100	8.17 [1.05, 63.41]

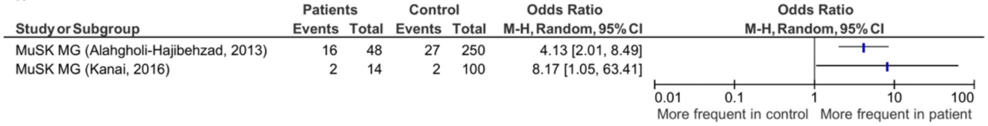

2n

Study or Subgroup	Patients Events	Patients Total	Control Events	Control Total	Weight	Odds Ratio M-H, Random, 95% CI
MuSK MG (Ehsan, 2015)	11	48	26	400	36.8%	4.28 [1.96, 9.35]
MuSK MG (Nikolic, 2014)	18	62	39	318	54.4%	2.93 [1.54, 5.56]
MuSK MG (Niks, 2006)	2	46	7	642	8.8%	4.12 [0.83, 20.44]
**Total (95% CI)**		**156**		**1360**	**100.0%**	**3.47 [2.16, 5.57]**
Total events	31		72			

Heterogeneity: Tau² = 0.00; Chi² = 0.59, df = 2 (P = 0.74); I² = 0%

Test for overall effect: Z = 5.14 (P < 0.00001)

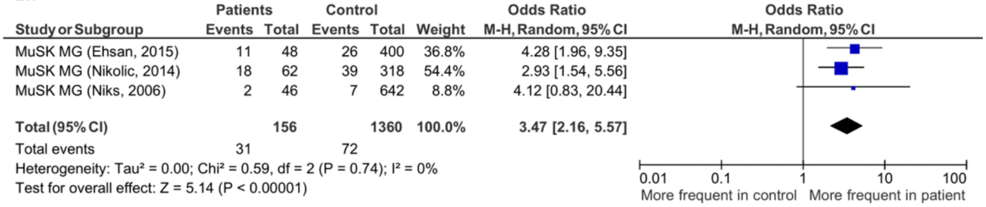

Figure S 104: Allele and genotype frequency of HLA-DRB1*16-DQB1*05 in MuSK myasthenia gravis

DRB1*15-DQB1*06

n

Study or Subgroup	Patients Events	Patients Total	Control Events	Control Total	Odds Ratio M-H, Random, 95% CI
MuSK MG (Ehsan, 2015)	5	48	44	400	0.94 [0.35, 2.50]
MuSK MG (Nikolic, 2014)	4	62	19	318	1.09 [0.36, 3.31]

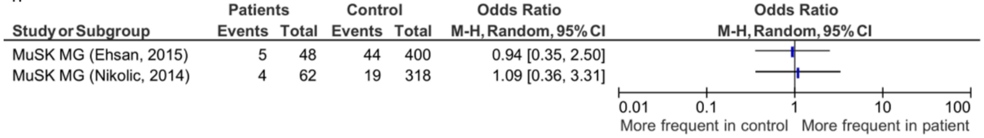

Figure S 105: Genotype frequency of HLA-DQB1*15-DQB1*06 in MuSK myasthenia gravis

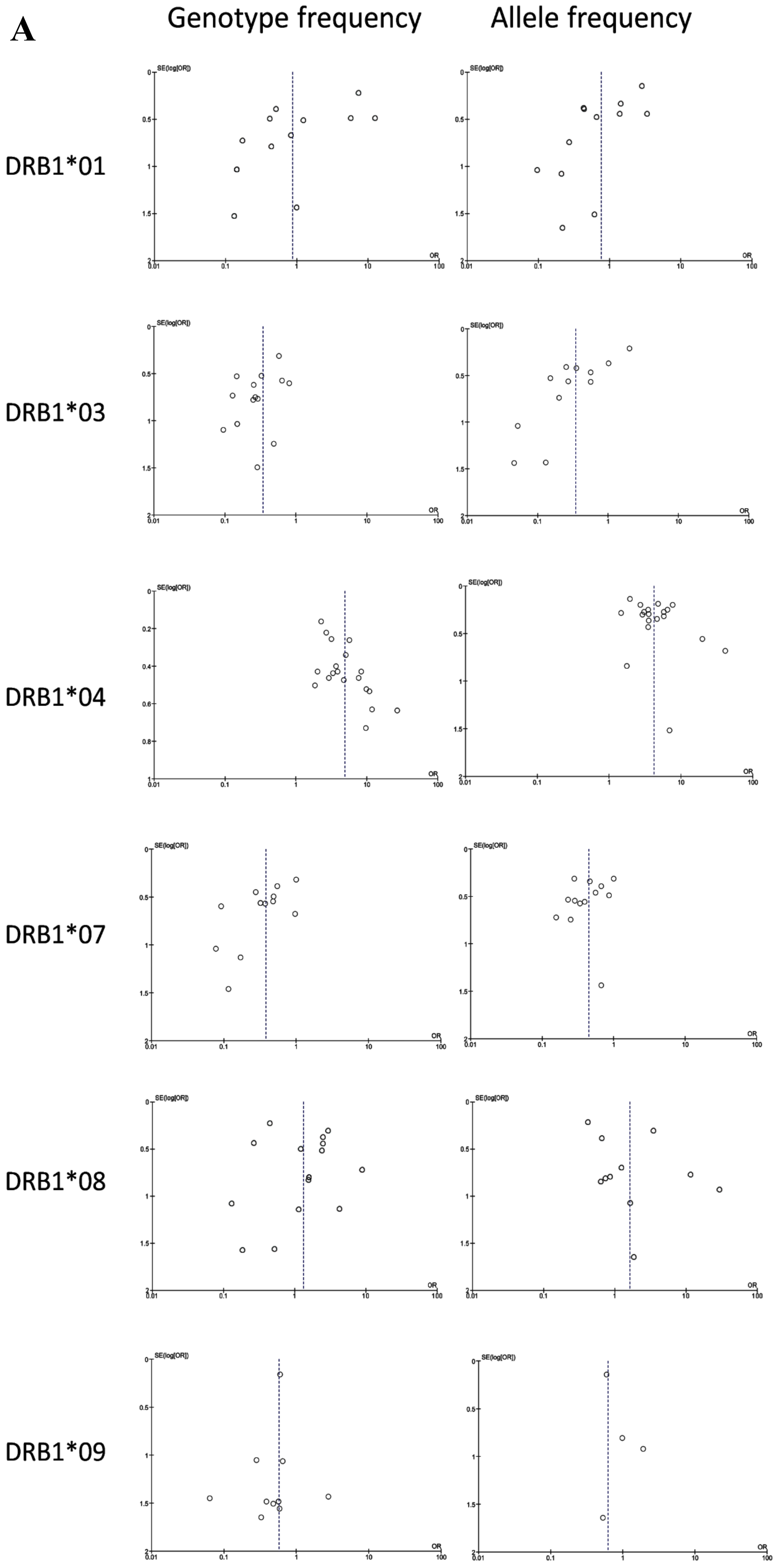

Figure S 106: continued on next page

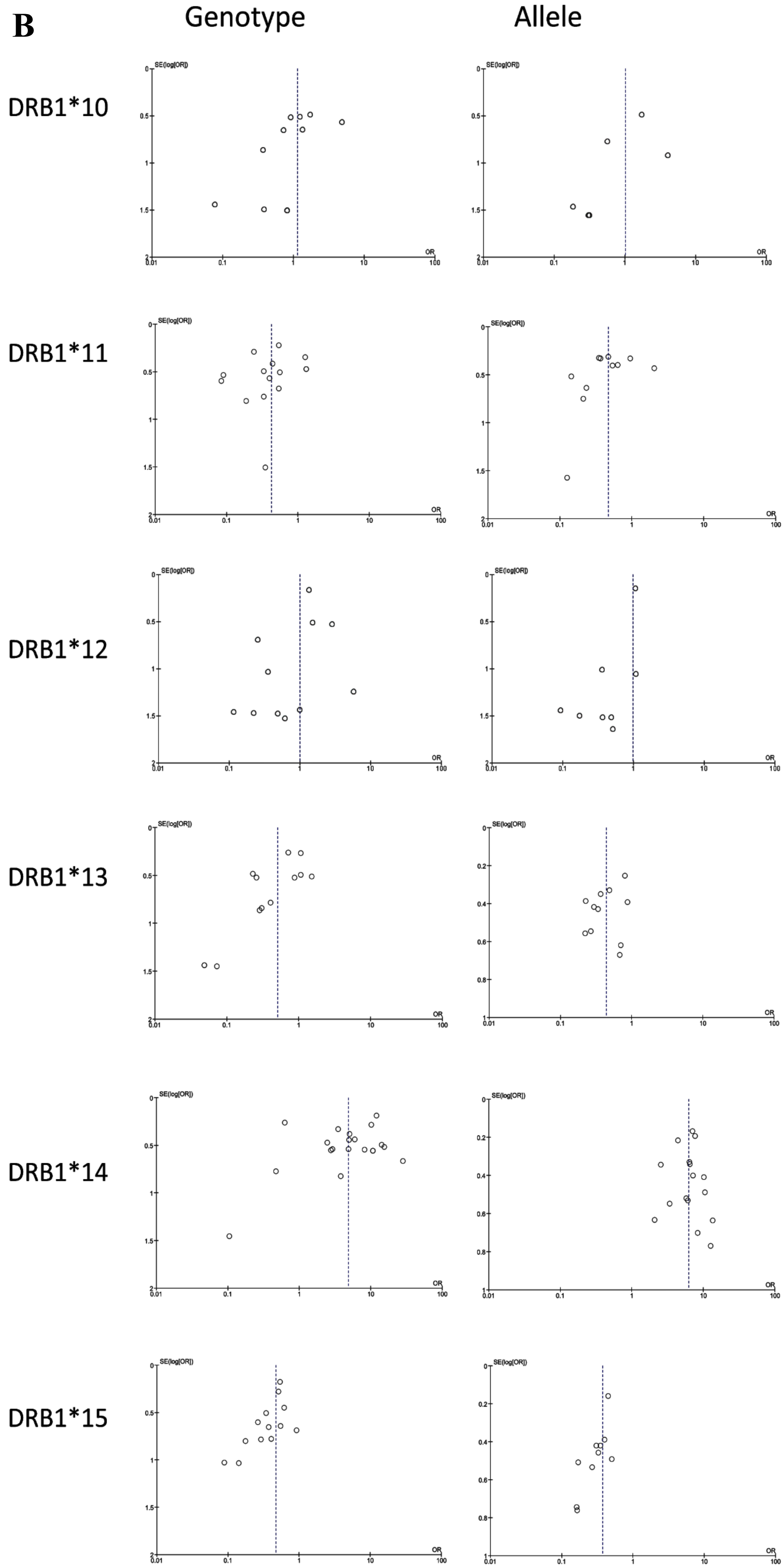

Figure S 106: Funnel plots for allele and genotype frequency of HLA-DRB1*01-DRB1*09 (A) and HLA-DRB1*10-DRB1*15 (B) in pemphigus

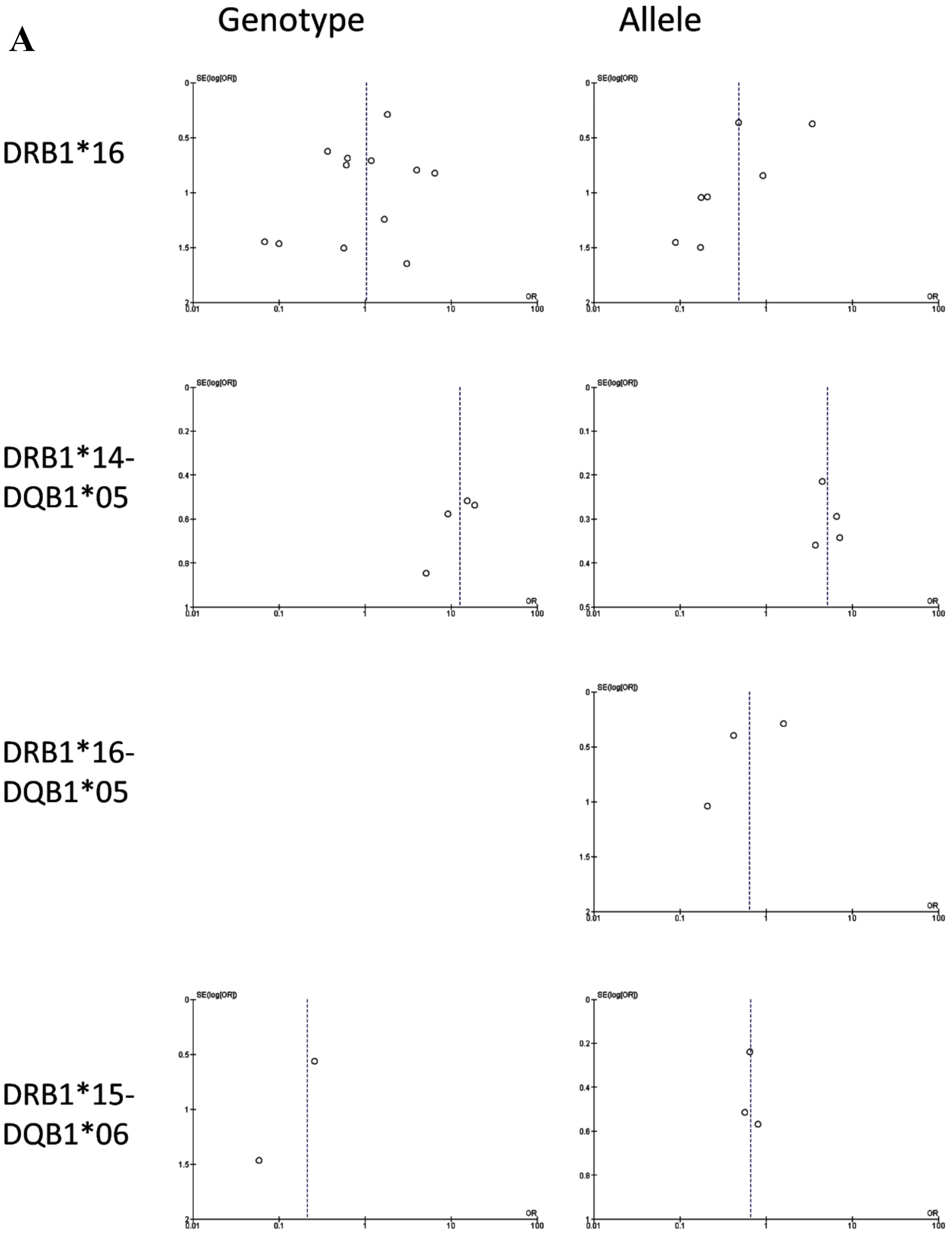

Figure S 107: continued on next page

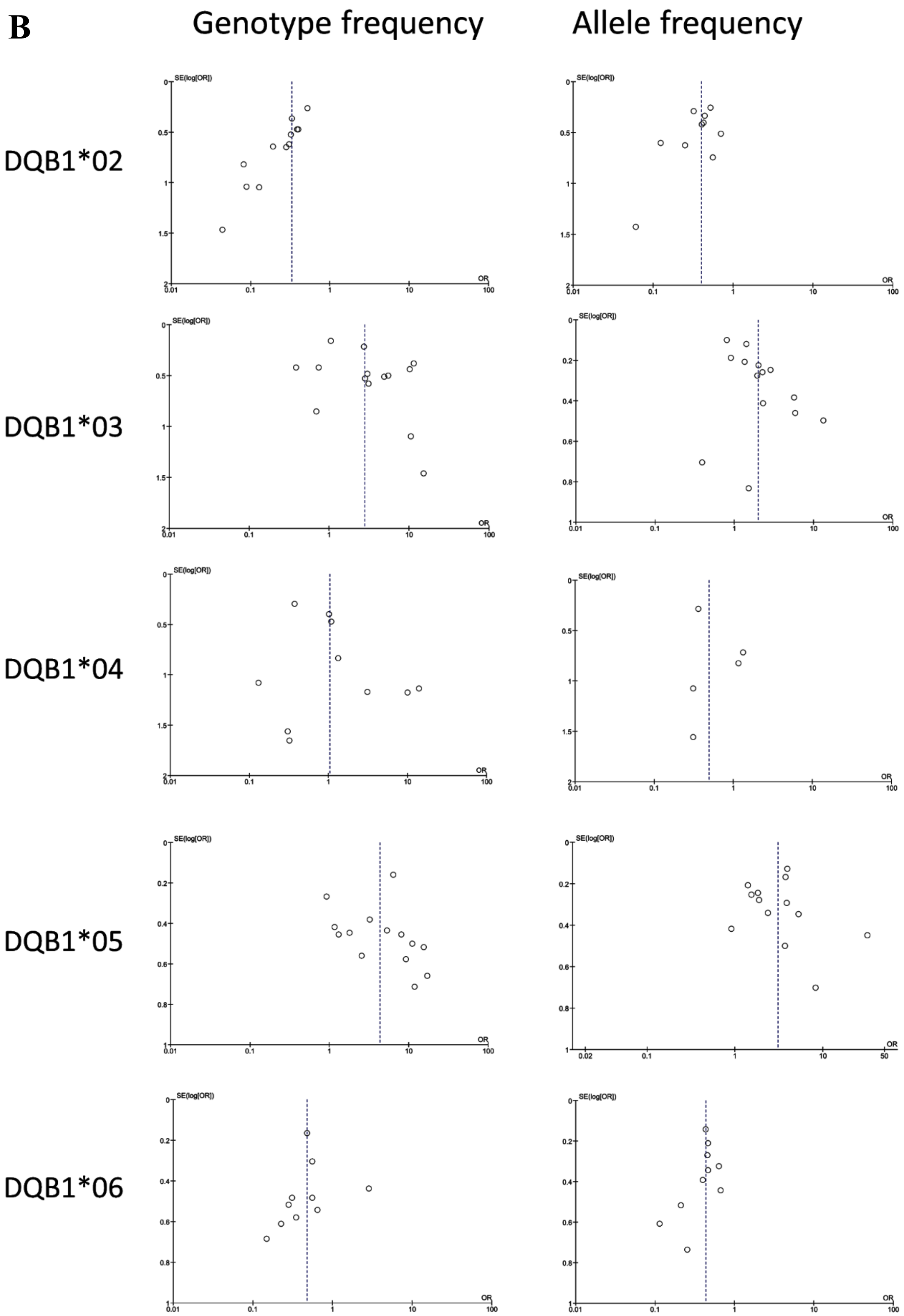

Figure S 107: Funnel plots for allele and genotype frequency of HLA-DRB1*16, HLA-DRB1*14-DQB1*05, HLA-DRB1*16-DQB1*05, HLA-DRB1*15-DQB1*06 (A) and HLA-DQB1*02-DQB1*06 (B) in pemphigus

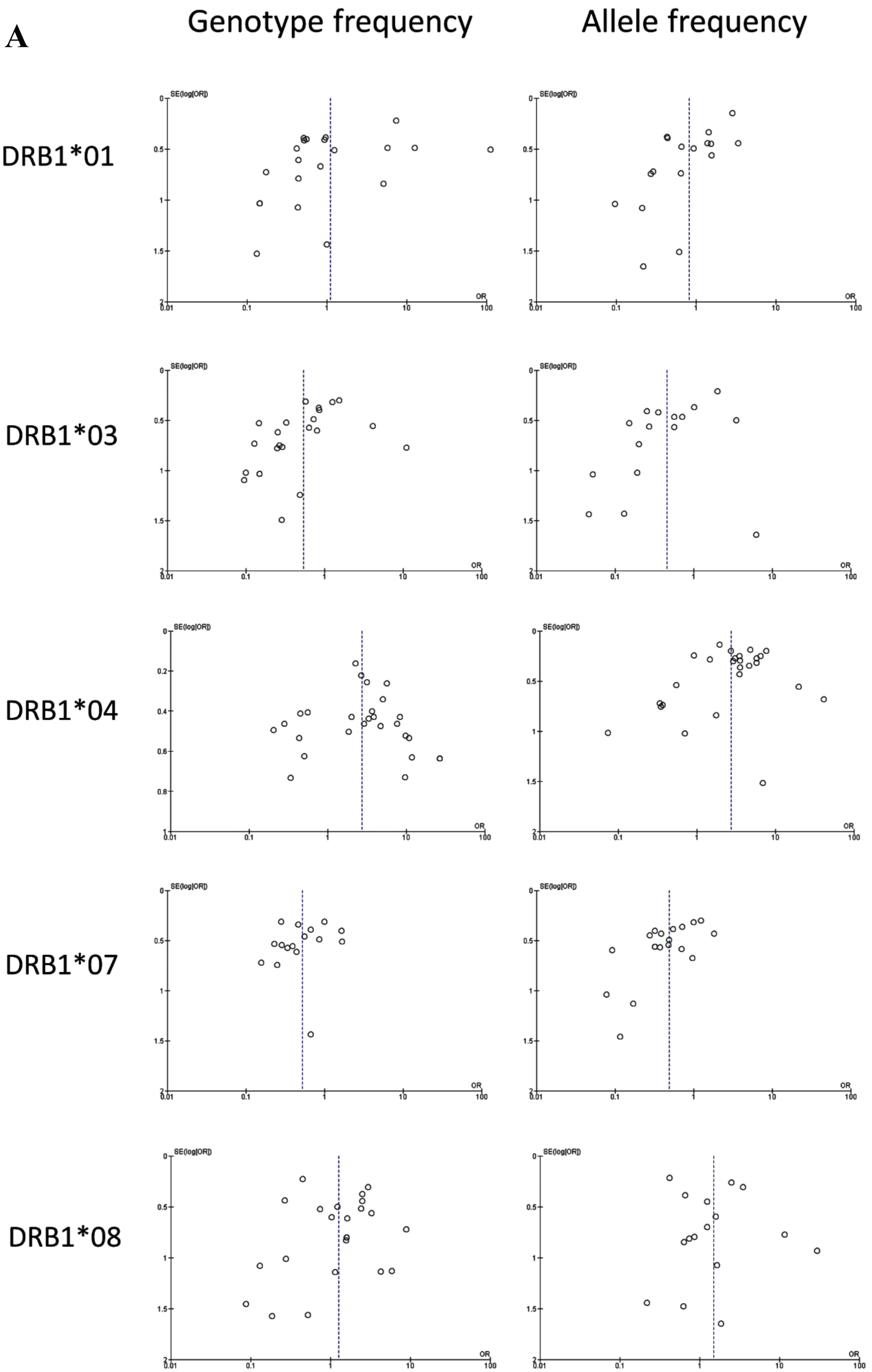

Figure S 108: continued on next page

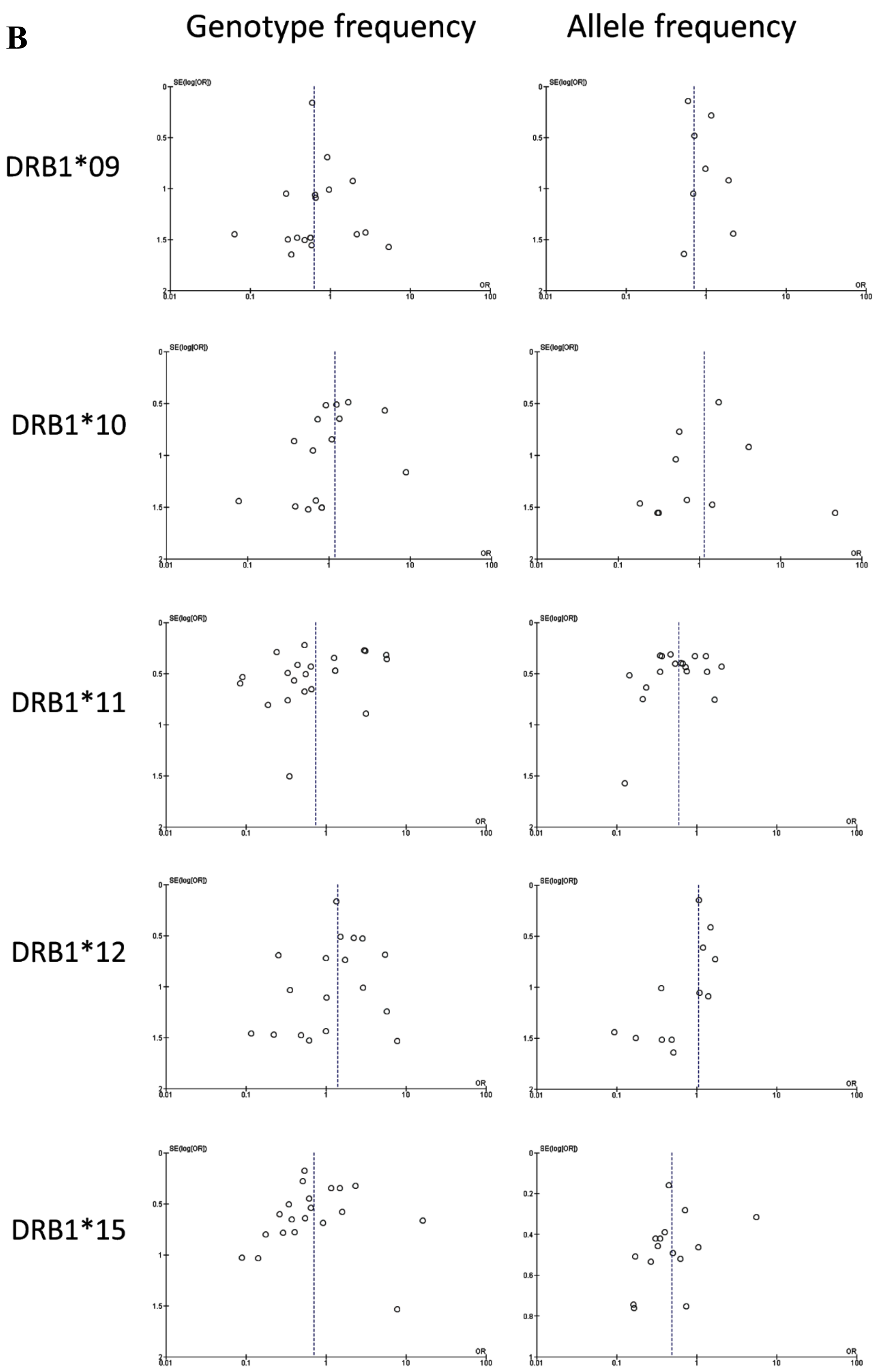

Figure S 108: Funnel plots for allele and genotype frequency of HLA-DRB1*01-DRB1*08 (A) and HLA-DRB1*09-DRB1*15 (B) in all diseases

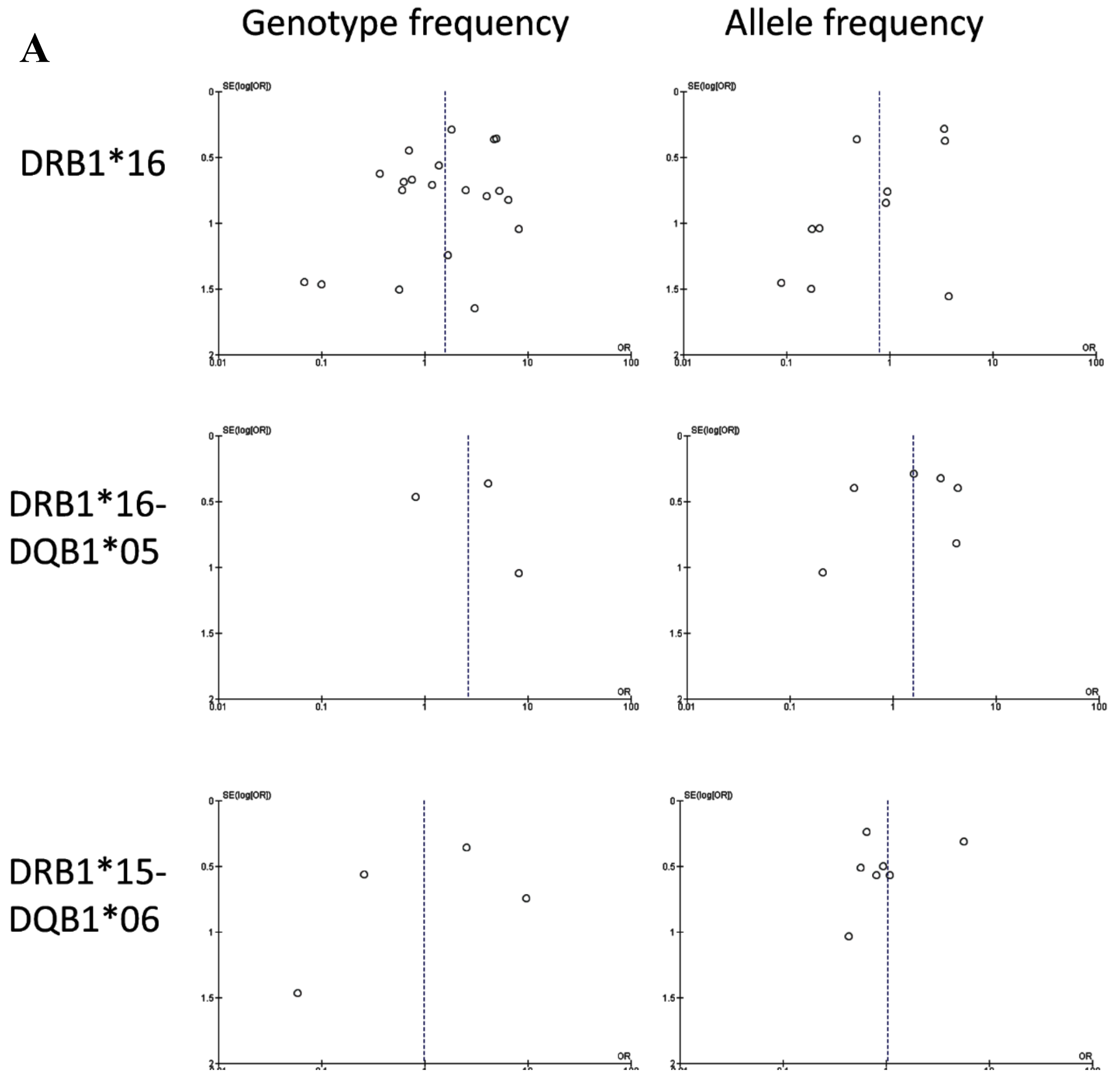

Figure S 109: continued on next page

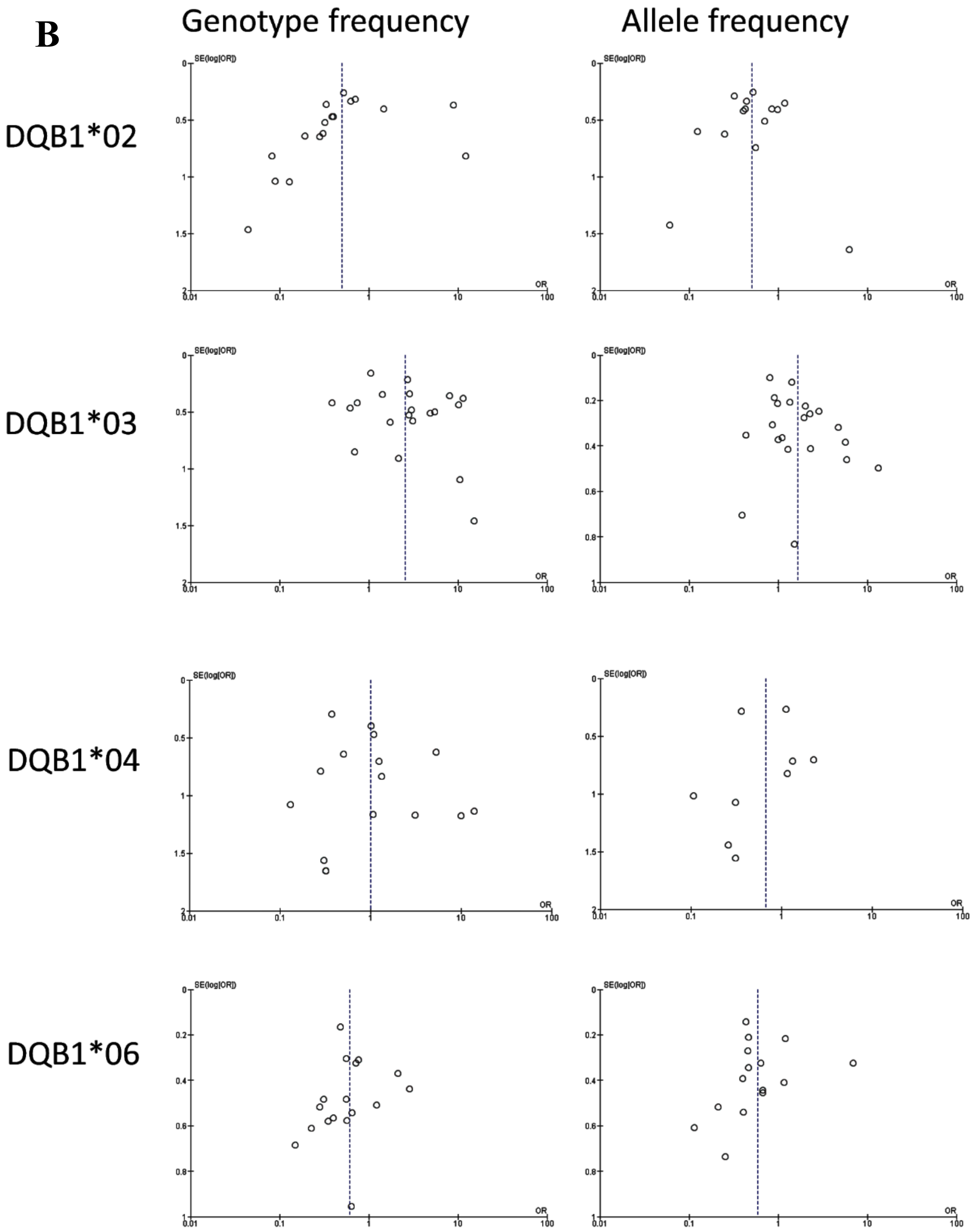

Figure S 109: Funnel plots for allele and genotype frequency of HLA-DRB1*16, HLA-DRB1*16-DQB1*05, HLA-DRB1*15-DQB1*06 (A) HLA-DQB1*02-DQB1*06 (B) in all diseases

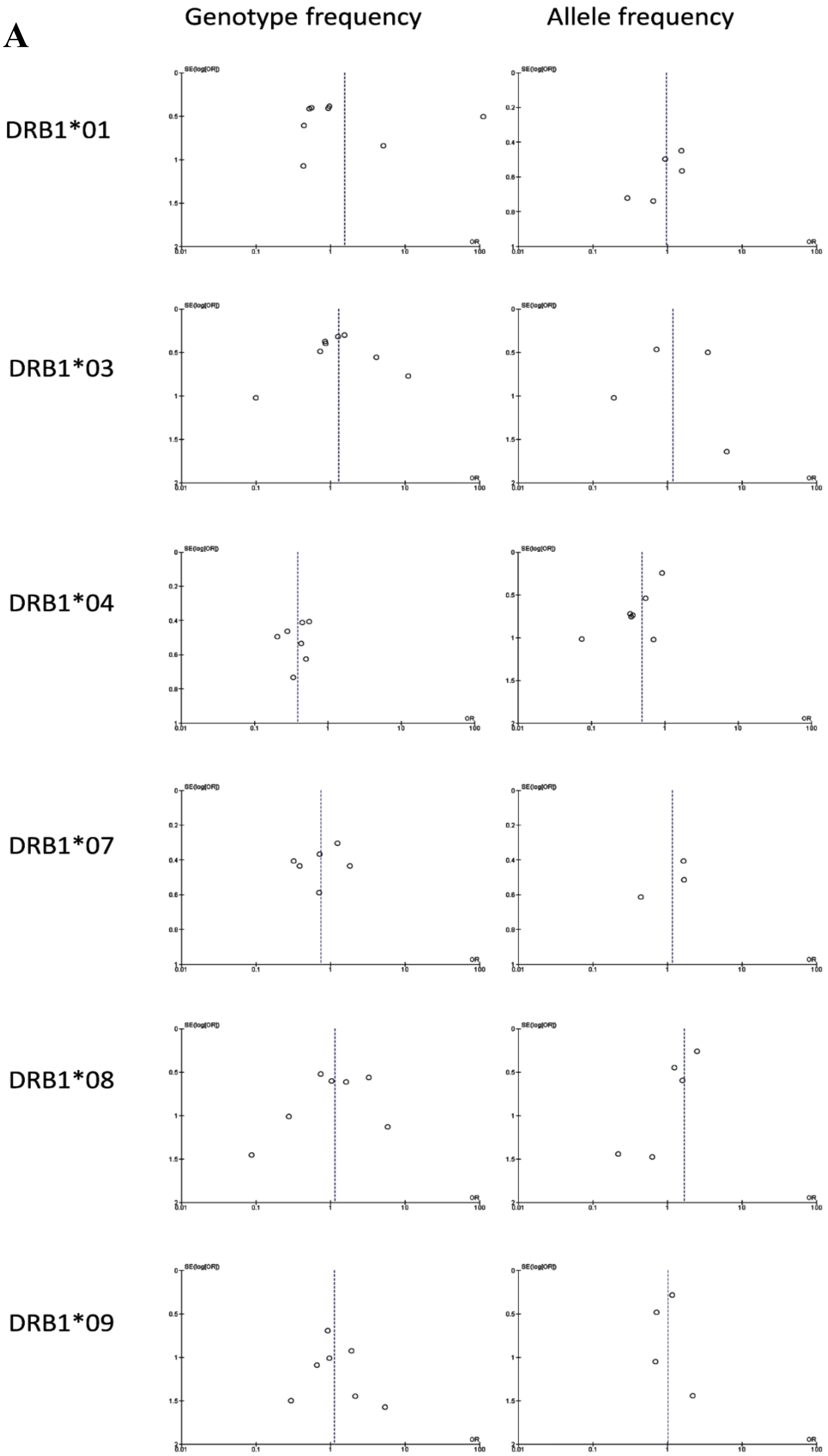

Figure S 110: continued on next page

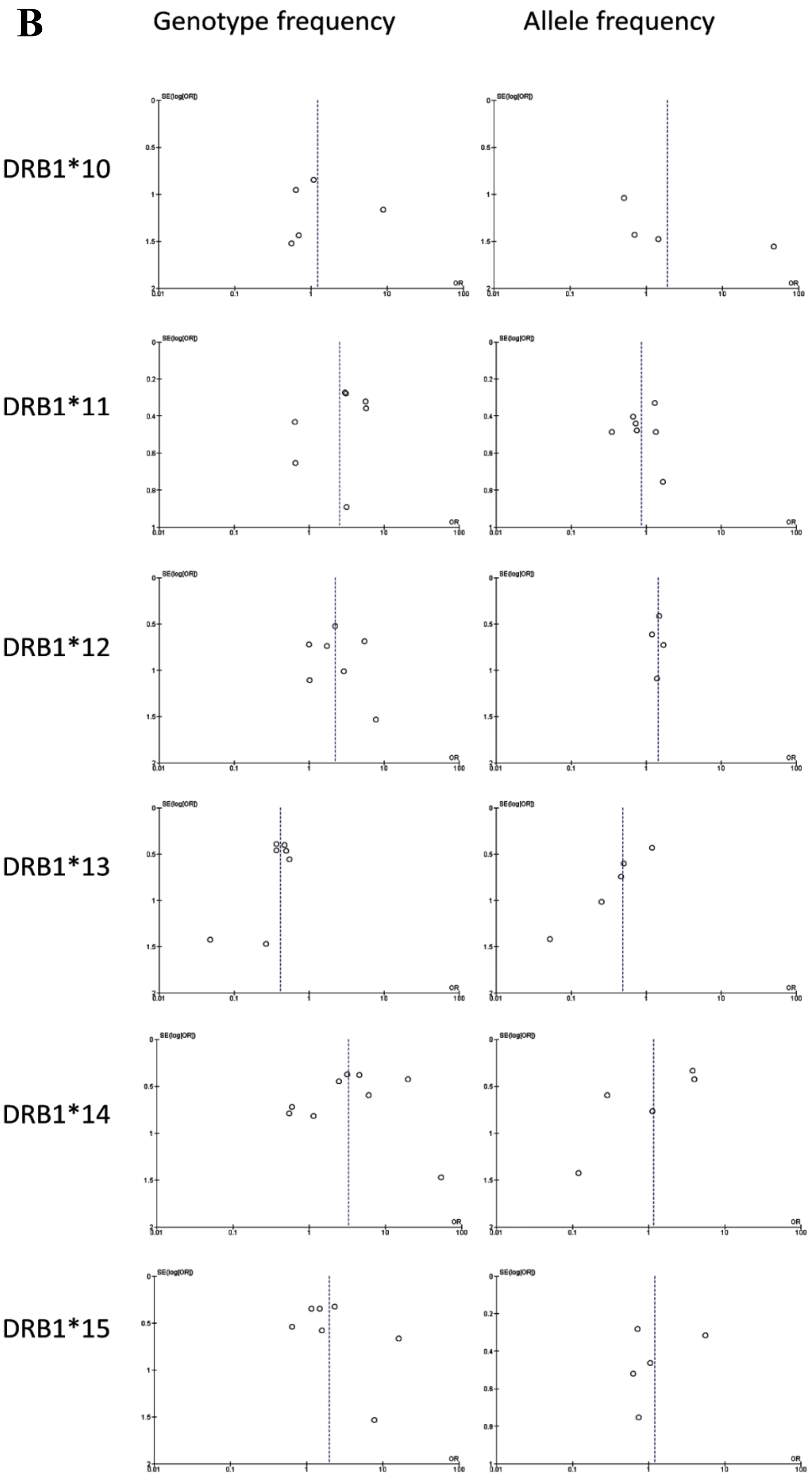

Figure S 110: Funnel plots for allele and genotype frequency of HLA-DRB1*01-DRB1*09 (A) and DRB1*10-DRB1*15 in MuSK MG, TTP and CIDP

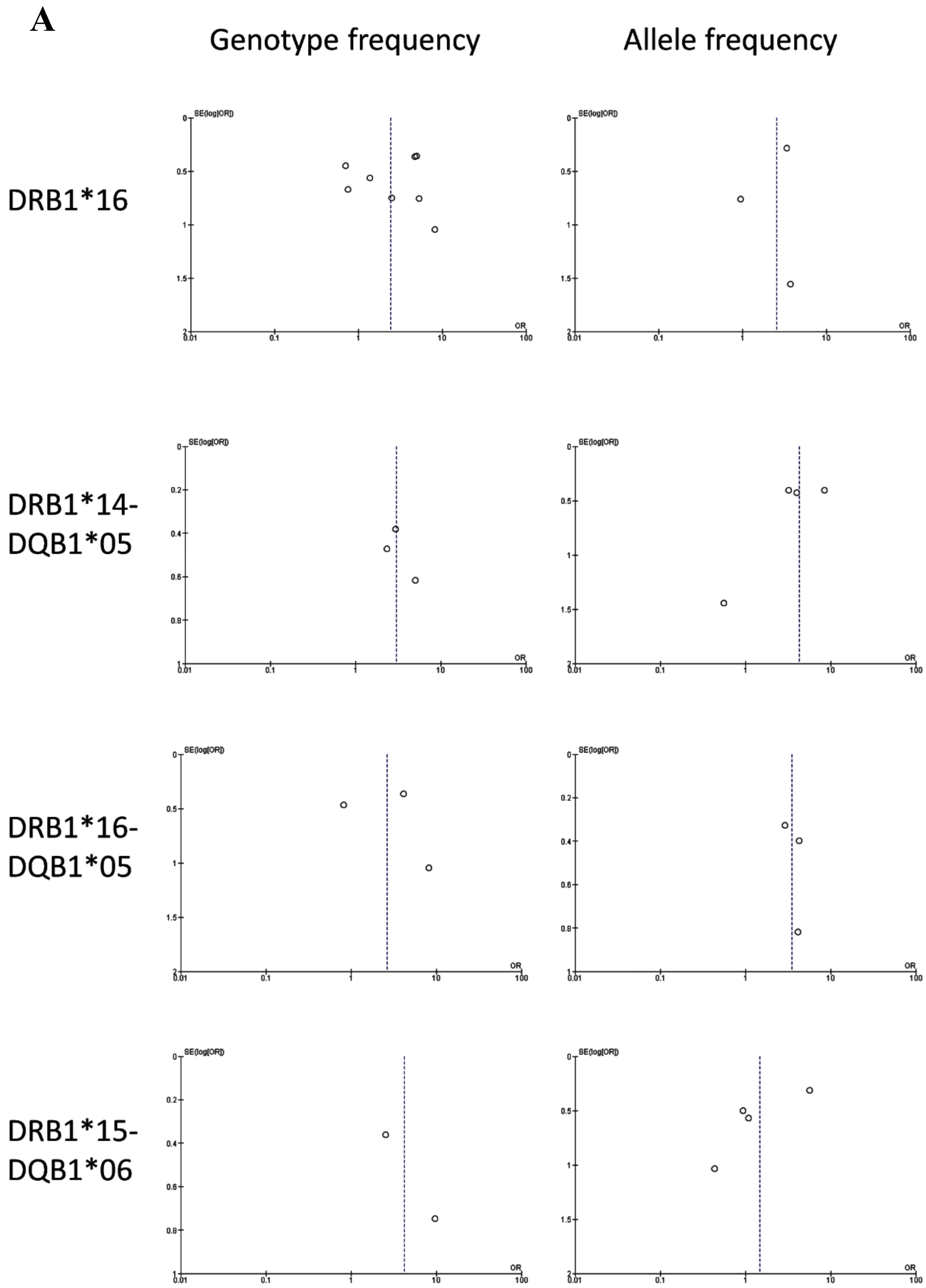

Figure S 111: continued on next page

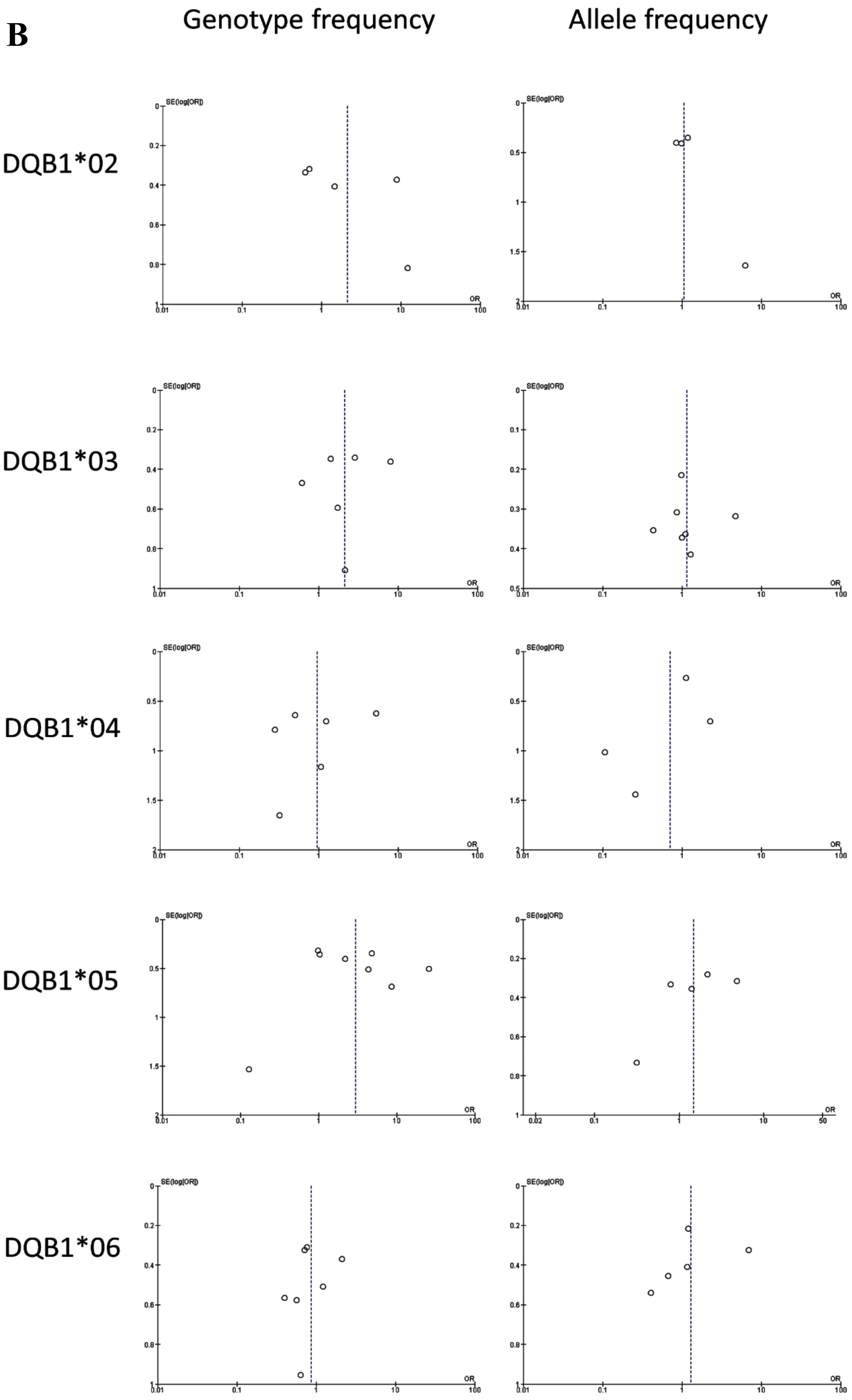

Figure S 111: Funnel plots for allele and genotype frequency of HLA-DRB1*16, HLA-DRB1*16-DQB1*05, HLA-DRB1*15-DQB1*06 (A) HLA-DQB1*02-DQB1*06 (B) in MuSK MG, TTP and CIDP

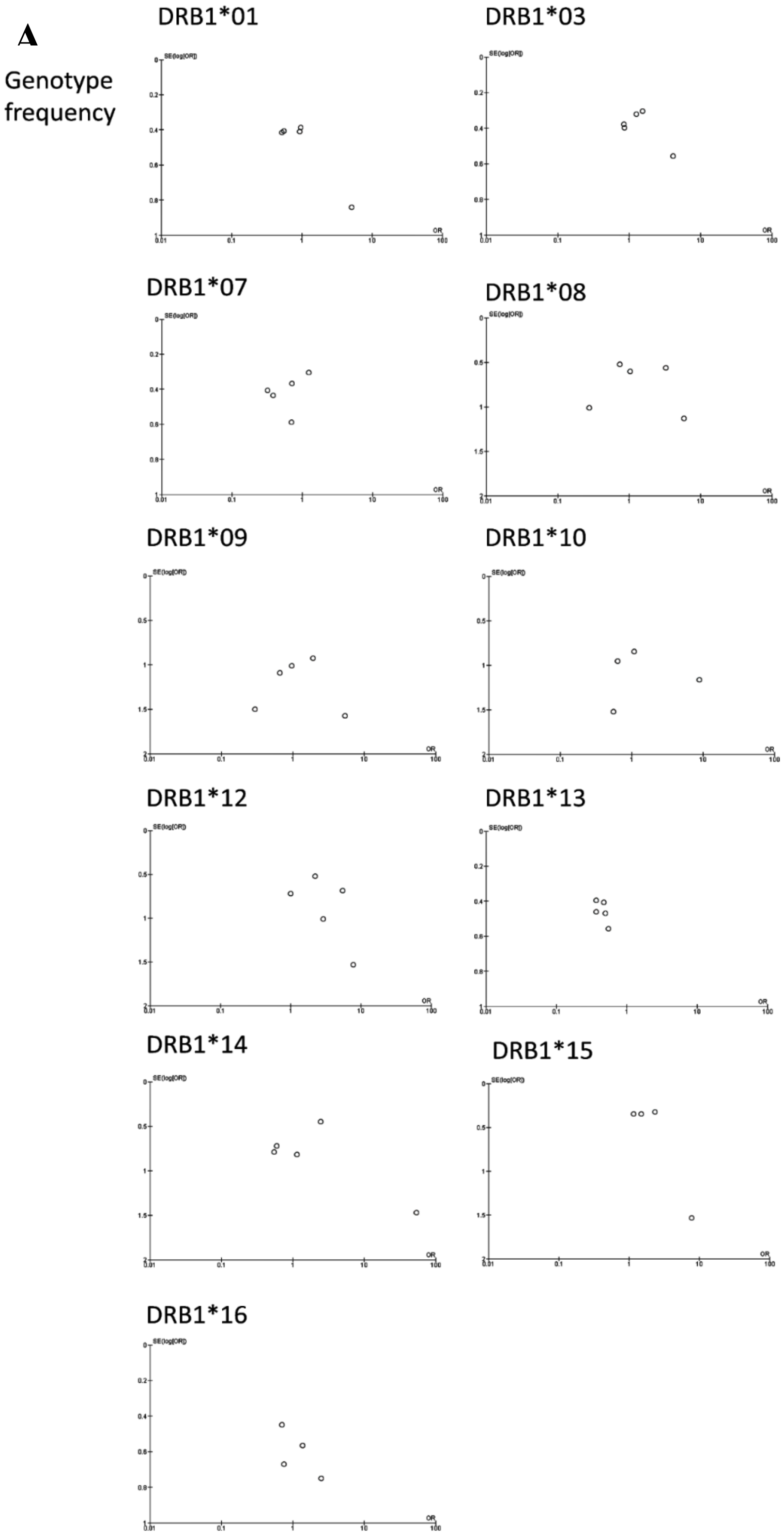

Figure S 112: continued on next page

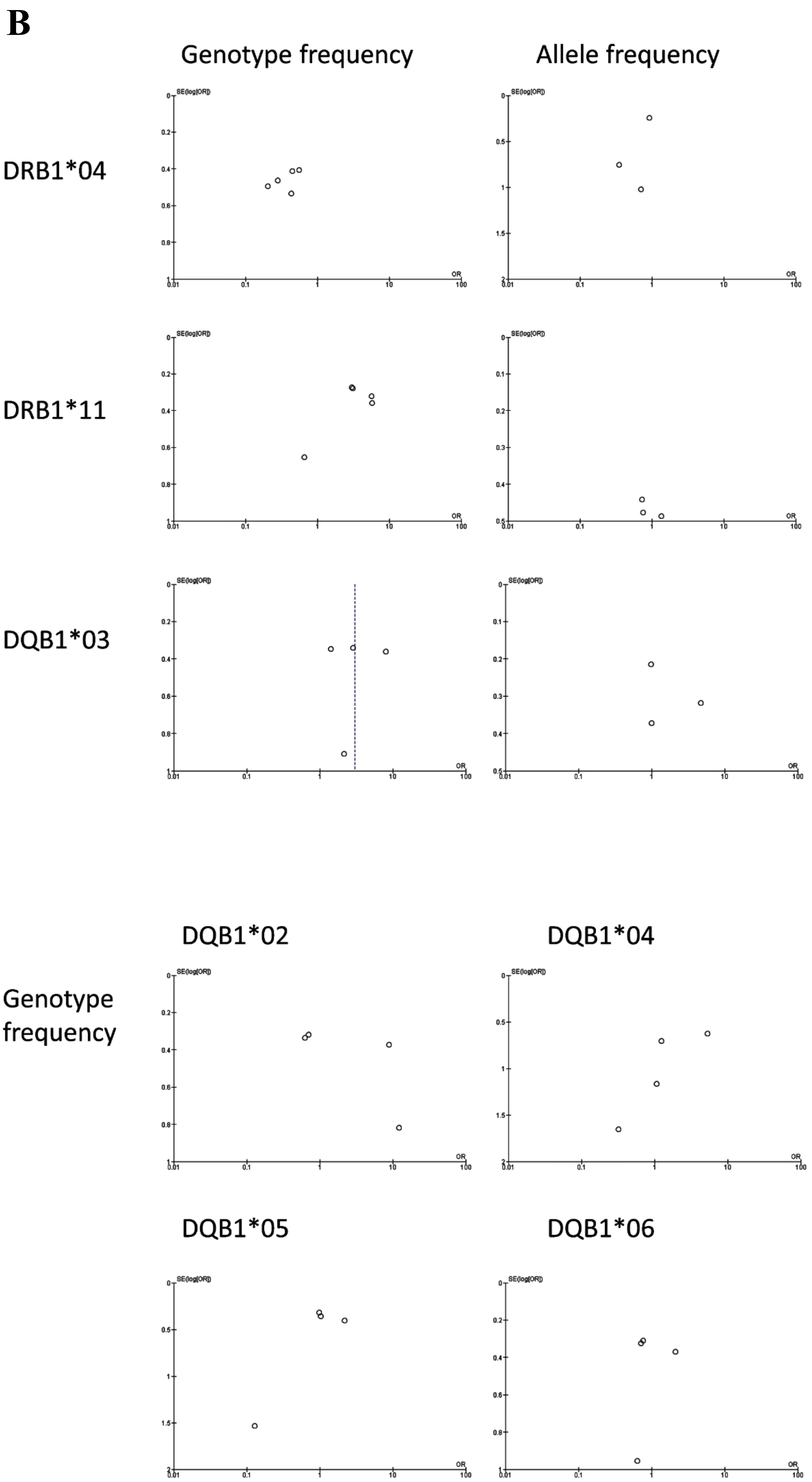

Figure S 112: Funnel plots for genotype frequency of HLA-DRB1*01-DRB1*16 (A) and HLA-DQB1*02-*06 (B). Allele and genotype frequency for HLA-DRB1*04, *11 and DQB1*03 (B) in thrombotic thrombocytopenic purpura

Protocol for a HuGE association review and meta-analysis

Cover sheet:
Are distinct alleles of the HLA-DRB1/DQB1 gene loci associated with class I IgG4 autoimmune diseases: a systematic association review and meta-analysis

Background:
The human leucocyte antigen (HLA) genes are expressed on antigen-presenting cells and epithelial cells in the thymus, and they code for the MHC class I and II cell surface receptors that are essential for presenting antigen to T-cells. MHC class II molecules (HLA-DR, HLA-DQ, and HLA-DP) present peptides to T helper cells. They are comprised of two different protein chains, α and *β*, that are non-covalently associated. HLA-DRB1 is the gene that codes for the *β1* -chain of the DR isotype and HLA-DQB1 codes for the *β1* -chain of the DQ isotype of the MHC class II cell surface receptor. HLA genes, especially the HLA-DRB1 gene, are highly polymorphic. In 2015, 1883 different alleles for HLA-DRB1 have been reported (Arango et al., 2017). The highest polymorphism is in the β1 domain of HLA-DRB1 which is in contact with the peptides that are presented to T-cells. The gene products of distinct HLA alleles will present distinct subsets of peptides (Pritchard et al., 2015). HLA genes are considered as a major contributor to genetic susceptibility to develop autoimmune diseases, and specifically HLA-DR has been linked to presentation of autoantigens to developing T-cells in the thymus and is associated with a range of autoimmune diseases, e.g. rheumatoid arthritis, diabetes mellitus type I, multiple sclerosis or systemic lupus erythematosus (Alvarez et al., 2015). It is thought that the presence of distinct HLA alleles may contribute to an aberrant presentation of self-peptide to autoreactive T helper cells in the thymus, thereby providing one of the first steps in the pathogenesis of autoimmune diseases (Arango et al., 2017).
Therefore, observing an association of specific alleles with distinct autoimmune diseases are important as they may foster our understanding of disease aetiology and pathogenesis and may present a diagnostic soft marker.

Rationale
IgG4 autoimmune diseases (IgG4-AID are a new group of autoimmune diseases first collectively described in 2015 (Huijbers et al., 2015) that are caused by pathogenic IgG4 autoantibodies targeting different antigens and sharing important mechanistic and therapeutic commonalities (Koneczny, 2018) (table 1). They affect one of four different organs (1. The peripheral and central nervous system, 2. skin and mucosa, 3. the kidneys and 4. the vascular system) and were previously not recognized to be related, but they share (1) IgG4 as predominant antibody subclass, and (2) the same pathogenic mechanism, which is an IgG4-mediated blocking of protein-protein interaction instead of complement mediated injury of target-organs (Koneczny, 2018, Koneczny et al., 2013), (3) they also share low disease prevalences of less than 5 per 10,000 (Koneczny, 2020, Autoimmun. Rev. in press). In MuSK MG and pemphigus vulgaris, there is a geographical decline of cases with degrees of latitude with high prevalence in the south and lower prevalence in the north, at least in Europe (Vodo et al., 2018, Koneczny et al., 2014). It is not clear what causes this distribution, but it is possible that genetic factors, such as the frequency of distinct HLA alleles, may contribute to this pattern (Hong et al., 2018).
IgG4-AID are classified by the degree of evidence for IgG4 pathogenicity (Koneczny, 2018), with IgG4 being proven as directly pathogenic in class I IgG4-AID. Class II and class III IgG4-AID are considered as potential IgG4-AID, but the evidence to date is insufficient and does not allow to include or exclude class II/III in the group of validated class I IgG4-AID. The six known class I IgG4-AID are 1.MuSK myasthenia gravis (antibodies to MuSK), 2. pemphigus vulgaris (antibodies

to desmoglein 3), 3.pemphigus foliaceus (antibodies to desmoglein 1 and/or 3), 4. chronic inflammatory demyelinating polyneuropathy (CIDP) with antibodies against Contactin-1, 5. CIDP with antibodies against Neurofascin 155 and 6. thrombotic thrombocytopenic purpura with antibodies against ADAMTS13 (Koneczny, 2020, Autoimm. Rev. in press).
A narrative review indicated a frequent genetic association of the individual IgG4-AID with a recurrent list of HLA supertypes (HLA-DRB1*04,11,14,15 and DQB1*05, Table 1), which suggests a shared immunopathogenesis (Koneczny, manuscript in preparation). However, it was a narrative review and not a systematic review with meta-analysis, which would provide stronger evidence of an association. To date, no systematic review or association review has been conducted to analyse whether IgG4-AID are overall genetically associated with the same HLA alleles. In this association review with meta-analysis, we aim to investigate if there is an overall association between class I IgG4-AID and HLA gene loci following the HuGENet™ HuGE Review Handbook, version 1.0.

Primary objective: Are individual class I IgG4-AID associated with distinct variants of the HLA gene loci?

Secondary objective: Are class I IgG4-AID collectively associated with distinct variants of the HLA gene loci?

Table 1: Hypothesis: HLA-associations of IgG4 autoimmune diseases. (N/A= no data available).Colours indicate organ predilection: green = PNS/CNS, yellow=skin/mucosa, red= vascular, blue=kidney.

Disease	Antigen/ Organ	HLA association	DRB1*14	DR4/ DRB1*04	DRB1*15	DQB1*05	DRB1*11	Protective alleles/ No association	References
Class I IgG4 autoimmune diseases									
MuSK-MG	/ PNS MuSK	HLA DQB1*05, DRB1*14, DRB1*16 , HLADQB*03 DQB1*0502, DQB1*0301/0313, DQA1*0101 (Iran)	x			x		DQA1* alleles (except Iran) HLA-DRB1*03 (Turkey) DRB1*13 (Serbia)	(Saruhan-Direskeneli et al., 2016, Ehsan et al., 2015, Bartoccioni et al., 2009, Alahgholi-Hajibehzad et al., 2013, Nikolic et al., 2015, Kanai et al., 2016, Cebi et al., 2019, Hong et al., 2018, Niks et al., 2006)
Pemphigus foliaceus, fogo selvagem (and p. vulgaris of mucocutanous type)	Desmoglein 1 /Skin / mucosa	PF: HLA-DRB1*04, HLA-DRB1*14, DRB1*1404 DQB1*0503, HLA-A10<br><br>FS: DRB1*01<br><br>DRB1*0404, *1402, *1406 or *0102	x	x				N/A	(Saha et al., 2019, Zhang et al., 2019, Hashimoto et al., 1977, Moraes et al., 1997, Lombardi et al., 1999, Loiseau et al., 2000)

Pemphigus vulgaris	Desmoglein 3/ skin/ mucosa	HLA-DRB1* 0402, HLA-DRB1*1401/ *1402, HLA-DQB1*05:03, HLA-DQB1*03 HLA-DRB1*03<br><br>DQB1*03:02,	x	x		x		DQB1*05:01, DQB1*02, DQB1*06:01, and DQB1*03:03	(Pollmann et al., 2018, Zhang et al., 2019, Hashimoto et al., 1977, Lombardi et al., 1999, Li et al., 2018)
Thrombic thrombocytopenic purpura	ADAMTS13 / blood circulation	HLA-DRB1*11, , HLA-DRB1*04, HLA-DRB1*14, HLA-DRB4, HLA-DQB1*03, and HLA-DQB1*05/*06/*02, HLA-A*03<br><br>DRB1∗15, HLA-DQB1*02:02	x	x	x	x		HLA-DRB1*03DRB1*07-DQB1*02 and DRB1 *13-DQB1 *06<br><br>HLA-DRB1*04 (John study)	(Dere et al., 2020, Lombardi et al., 2018, Coppo et al., 2010, Sinkovits et al., 2017, John et al., 2012)
CIDP,AIDP	Neurofascin 155 / PNS	HLA-DRB15*01 and *02			x			N/A	(Martinez-Martinez et al., 2017)
CIDP	CNTN1 (Contactin 1)/ CNS	N/A	N/A	N/A	N/A	N/A	N/A	N/A	N/A
Class II IgG4 autoimmune diseases									
Encephalitis, Morvan's syndrome	LGI1/ CNS	HLA-DRB1*07:01and linked alleles<br><br>(HLA-DRB1*07:01-DQB1*02:02)<br><br>HLA-DR7 and HLA-DRB4<br><br>HLA-B*44:03, C*07:06		x				N/A	(Binks et al., 2018, Mueller et al., 2018, van Sonderen et al., 2017, Kim et al., 2017)
Membranous nephropathy	PLA2R /kidneys	DRB1*15:01, DRB3*0202,			x			N/A	(Beck, 2017, Cui et al., 2017, Le et al., 2017)
CNS and PNS disorders	CASPR2/ CNS/ PNS	HLA-DRB1*11:01 (Linkage: DRB1*11:01-DQA1*05:01-DQB1*03:01)						N/A	(Binks et al., 2018)
Membranous nephropathy	THSD7A / kidneys	N/A	N/A	N/A	N/A	N/A	N/A	N/A	N/A
GPIHBP1 autoantibody syndrome	GPIHBP1 / blood circulation	N/A	N/A	N/A	N/A	N/A	N/A	N/A	N/A
CIDP	CASPR1/ CNS	N/A	N/A	N/A	N/A	N/A	N/A	N/A	N/A
Mucous membrane pemphigoid	Laminin 332 / skin/mucosa	Unclear/ N/A	N/A	N/A	N/A	N/A	N/A	N/A	N/A

Class III IgG4 autoimmune diseases									
Goodpasture syndrome	Type IV collagen/ kidneys	HLA-DRB1*15:01 (DRB1*0404) DRB1∗15:02		x	x			N/A	(Hellmark and Segelmark, 2014, Phelps and Rees, 1999, Xie et al., 2017, Yang et al., 2009, Kitagawa er al., 2008)
Iglon5 Parasomnia	Iglon5/ CNS	HLA-DRB1*10:01, HLA-DQB1*05:01				x		N/A	(Gaig et al., 2017)
Bullous pemphigoid	Bulloid pemphigoid antigen 180/230 /skin/ mucosa	DQB1*0301, DRB1*04, DRB1*11,<br><br>DQB1*0501 and DRB1*1001, DQA1*05,		x		x		HLA-DQA1*01:02 /03, HLA-DQB1*02:02 , and HLA-DRB1*07:01	(Fang et al., 2018, Setterfield et al., 2001, Zakka et al., 2001, Oyama et al., 2006)
Granulomatosis with Polyangiitis	ANCA/ blood circulation	HLA-DPB1*0401, HLA-DR1, HLA-DR4, HLA-DRB1*0901-HLA DQB1*0303 , HLA-DR9, HLA-DR13, HLA-DRB1*1202, HLA-DRB1*15, HLA-DRB1*0405		x	x				(Watts et al., 2015, Wu et al., 2017, Alberici et al., 2014)
Autoimmune polyendocrine syndrome type 1 / APECED	IFN I, IL-17A, IL-17F and IL-22/ blood circulation	DRB1*03:01, DRB1*04:01, DQA1*03:01, DQA1*05:01, DQB1*02:01, DQB1*03:02<br><br>Subtypes:<br><br>Addison's disease: HLA-DRB1*03, Alopecia: HLA-DRB1*04- DQB1*0302			x			Subtype with type 1 diabetes: HLA-DRB1*15-DQB1*0602	(Meloni et al., 2012, Flesch et al., 2014, Halonen et al., 2002)
CIDP	Neurofascin 140/186 / PNS	N/A	N / A	N / A	N / A	N / A	N / A	N/A	N/A
Anti-laminin γ1 / Anti-P200 pemphigoid	P200 (laminin γ1) / skin/ mucosa	N/A	N / A	N / A	N / A	N / A	N / A	N/A	N/A
DPPX encephalitis	DPPX/ CNS	N/A	N / A	N / A	N / A	N / A	N / A	N/A	N/A
Membranous nephropathy	αenolase, SOD2, AR/ kidneys	N/A	N A /	N / A	N / A	N / A	N / A	N/A	N/A

Methods

<u>Selection criteria</u>

<u>Inclusion criteria:</u>

1) Studies with a case-control design.
2) Studies reporting the association of gene variants of the HLA-DR and/or HLA-DQ gene locus, including allele, genotype or haplotype frequency.

3) Studies in which cases are patients with class I IgG4 autoimmune diseases that were tested positive for the relevant antibodies ((MuSK myasthenia gravis (antibodies to MuSK) pemphigus vulgaris (antibodies to desmoglein 3), pemphigus foliaceus (antibodies to desmoglein 1 and/or 3), peripheral neuropathies, including CIDP, (antibodies against Contactin 1), peripheral neuropathies, including CIDP, (antibodies against Neurofascin 155), thrombotic thrombocytopenic purpura (antibodies against ADAMTS13)) by standardized laboratory tests, including the following tests: ELISA, cell-based assay (CBA), radioimmuno(precipitation)assay (RIA), direct or indirect immunofluorescence test.
4) Studies with a minimum of 1 control per case, age and gender matched.
5) Studies with controls that are either healthy individuals or patients with a different type of the same disease that were negative for their relevant autoantibodies, as well as any other IgG4 associated autoantibody (listed in table 1) or with an unrelated disease.

<u>Exclusion criteria:</u>

1) studies in which the controls have any immunodeficiencies/abnormalities in the HLA locus.
2) studies in which data on an individual patient level is not available.
3) studies that have included overlapping subjects with already published studies.
4) Studies where full text is not available
5) Studies with insufficient data to calculate OR

<u>Identifying studies</u>

We will search in bibliographic databases and archives including PubMed/MEDLINE, Cochrane CENTRAL and Cochrane CDSR, Web of Science Core Collection, BIOSIS, Scopus, regional databases (IMEMR (EMRO), IMSEAR (SEARO), LILACS (AMRO/OPAS)), Ovid Global Health, clinical trial registries (ClinicalTrials.gov and WHO ICTRP), and databases of systematic reviews (Epistemonikos, PROSPERO), BioOne,, Centre for Reviews and Dissemination, CINAHL, Cochrane CDSR, Cochrane Library, DOAJ, EMBASE, Epistemonikos, EU Clinical Trials Register, GlaxoSmithKline's Clinical Study Register, Godort, HSRProj, JSTOR, Mendeley, metaRegister of Controlled Trials (Current controlled trials), Ovid global health, Prospero, Research gate, Science Citation Index (ISI), Science direct, TRIP Database, U.S. Government Documents, WHO ICTRP, Worldcat,
https://www.biorxiv.org/ and https://www.medrxiv.org as well as using other sources including grey literature (open grey) and hand searching.
We will search for key words, MeSH terms, including wild cards (*), regional variations (UK/US) and misspelling. These will include the terms "HLA," "human leucocyte antigen", "DRB1", "DQB1","myasthenia gravis", "pemphigus", "thrombotic thrombocytopenic purpura", " CIDP", "chronic inflammatory demyelinating polyneuropathy","Neuropathy", "MuSK", "Desmoglein 3", "Desmoglein 1", "blistering skin disease", "ADAMTS13", Neurofascin 155", "NF155", "Contactin-1", "CNTN1 and related terms in titles and abstracts using Boolean search strategies.
After identification of relevant publications, we will deduplicate obtained records. We will screen obtained records for eligibility based on inclusion/exclusion criteria over two phases using three independent reviewers that are blinded for authors/title. Discrepancies will be resolved via discussion. We will screen 1 – titles and abstracts, 2 – full texts (e.g. with Rayyan, EndNote). The search and selection of studies will be documented and visualized with a PRISMA flow chart (Moher et al., 2009).

<u>Data collection and analysis</u>

We will extract and collect data from tables and running text in the included manuscripts. If data is incomplete, unpublished or unavailable, we will attempt to retrieve the data by contacting the corresponding authors of the study by email.
We will extract the following information from each included study:
Primary author, year of publication, full bibliographic information, standardized (e.g. Vancouver), demographic information of patients and controls (sex, age), country, type of IgG4-AID and/or type of autoantibody, affected organ, type of control, HLA typing method, sample size, genotype frequency, allele frequency, haplotype frequency, HLA supertype frequency, OR and 95%CI.
If the data is combinable, we aim to conduct a meta-analysis for association between HLA variants (HLA allele, genotype, haplotype and/or supertype) and class I IgG4-AID individually and, if feasible, collectively.

Primary hypotheses:

1) HLA-DRB1*04,11,14,15 and/or HLA-DQB1*05 are associated with class I IgG4-AID individually.

Secondary hypothesis:

2) HLA-DRB1*04,11,14,15 and/or HLA-DQB1*05 are associated with class I IgG4-AID collectively.

Null hypothesis:
HLA-DRB1*04,11,14,15 and/or HLA-DQB1*05 are not associated with class I IgG4-AID individually or collectively.
We will calculate odds ratios (ORs) for allele, haplotype and genotype frequencies with 95% confidence intervals (95% CIs) to assess strength of association.
The heterogeneity of the included studies will be measured using Cochran Q's test and I^2 value.
The combined effect of the included studies (pooled OR) will be calculated with the Mantel-Haenszel test.
The publication bias will be tested by using funnel plots and the Egger's test.
P values will be adjusted for multiple comparisons by the false discovery rate (FDR) method and will be corrected separately for single locus analysis and haplotype analysis. Corrected P values <.05 will be considered statistically significant.

Potential conflicts of interest
The authors declare no conflict of interest.

References

1. Dere, G. *et al.* Assessment of HLA-A, HLA-DR, and HLA-DQ alleles in patients with pemphigus vulgaris from eastern of Turkey. *Journal of Cosmetic Dermatology* **n/a** (2020).

2. Ehsan, S. *et al.* Association of HLA class II (DRB1, DQA1, DQB1) alleles and haplotypes with myasthenia gravis and its subgroups in the Iranian population. *Journal of the Neurological Sciences* **359**, 335-342 (2015).

3. Alahgholi-Hajibehzad, M. *et al.* Association of HLA-DRB1∗14, -DRB1∗16 and -DQB1∗05 with MuSK-myasthenia gravis in patients from Turkey. *Human Immunology* **74**, 1633-1635 (2013).

4. Harfouch, E. & Daoud, S. Allelic variation in HLA-DRB1* loci in Syrian pemphigus vulgaris patients. *International Journal of Dermatology* **53**, 1460-1463 (2014).

5. González-Escribano, M.F. *et al.* Distribution of HLA class II alleles among Spanish patients with pemphigus vulgaris. *Tissue Antigens* **52**, 275-278 (1998).

6. Brochado, M.J.F. *et al.* Differential HLA class I and class II associations in pemphigus foliaceus and pemphigus vulgaris patients from a prevalent Southeastern Brazilian region. *Journal of Autoimmunity* **72**, 19-24 (2016).

7. Martel, P. *et al.* Epistasis between DSG1 and HLA class II genes in pemphigus foliaceus. *Genes & Immunity* **3**, 205-210 (2002).

8. Párnická, Z., Švecová, D., Javor, J., Shawkatová, I. & Buc, M. High susceptibility to pemphigus vulgaris due to HLA-DRB1*14:54 in the Slovak population. *International Journal of Immunogenetics* **40**, 471-475 (2013).

9. Nikolic, A.V. *et al.* High frequency of DQB1*05 and absolute absence of DRB1*13 in muscle-specific tyrosine kinase positive myasthenia gravis. *European Journal of Neurology* **22**, 59-63 (2015).

10. de Sena Nogueira Maehara, L. *et al.* HLA class II alleles of susceptibility and protection in Brazilian and Dutch pemphigus foliaceus. *British Journal of Dermatology* **178**, e212-e214 (2018).

11. Coppo, P. *et al.* HLA-DRB1*11: a strong risk factor for acquired severe ADAMTS13 deficiency-related idiopathic thrombotic thrombocytopenic purpura in Caucasians. *Journal of Thrombosis and Haemostasis* **8**, 856-859 (2010).

12. Kanai, T. *et al.* HLA-DRB1*14 and DQB1*05 are associated with Japanese anti-MuSK antibody-positive myasthenia gravis patients. *J Neurol Sci* **363**, 116-118 (2016).

13. Cotti Piccinelli, S. *et al.* Human leukocyte antigens class II in CIDP spectrum neuropathies. *Journal of the Neurological Sciences* **407**, 116533 (2019).

14. Zivanovic, D., Bojic, S., Medenica, L., Andric, Z. & Popadic, D. Human leukocyte antigen class II (DRB1 and DQB1) alleles and haplotypes frequencies in patients with pemphigus vulgaris among the Serbian population. *HLA* **87**, 367-374 (2016).

15. Saha, M. *et al.* Sporadic pemphigus foliaceus and class II human leucocyte antigen allele associations in the white British and Indo-Asian populations in the UK. *Clinical and Experimental Dermatology* **44**, 290-294 (2019).

16. Gil, J.M. *et al.* Study of the association between human leukocyte antigens (HLA) and pemphigus vulgaris in Brazilian patients. *International Journal of Dermatology* **56**, 557-562 (2017).

17. Torzecka, J.D. *et al.* Tumour necrosis factor-alpha polymorphism as one of the complex inherited factors in pemphigus. *Mediators Inflamm* **12**, 303-307 (2003).

18. Abida, O. *et al.* Tunisian endemic pemphigus foliaceus is associated with the HLA-DR3 gene: anti-desmoglein 1 antibody-positive healthy subjects bear protective alleles. *British Journal of Dermatology* **161**, 522-527 (2009).

19. Ogata, H. *et al.* Unique HLA haplotype associations in IgG4 anti-neurofascin 155 antibody-positive chronic inflammatory demyelinating polyneuropathy. *Journal of Neuroimmunology* **339**, 577139 (2020).

20. Priyadarshini, A., George, R., Daniel, D., Varughese, S. & Jayaseelan, V. Association between human leukocyte antigen-DRB1 and human leukocyte antigen-DQB1 alleles and pemphigus vulgaris in Indian patients: A case-control study. *Indian J Dermatol Venereol Leprol* **84**, 280-284 (2018).

21. Tunca, M., Musabak, U., Sagkan, R.I., Koc, E. & Akar, A. Association of human leukocyte antigen class II alleles with pemphigus vulgaris in a Turkish population. *The Journal of Dermatology* **37**, 246-250 (2010).

22. Haase, O. *et al.* Association with HLA-DRB1 in Egyptian and German pemphigus vulgaris patients. *Tissue Antigens* **85**, 283-286 (2015).

23. Martinez-Martinez, L. *et al.* Anti-NF155 chronic inflammatory demyelinating polyradiculoneuropathy strongly associates to HLA-DRB15. *J Neuroinflammation* **14**, 224-224 (2017).

24. Glorio, R.R. *et al.* Determinacion por PCR de la asociacion entre antigenos HLA clase II y penfigo vulgar. *Medicina (Buenos Aires)* **59**, 28-32 (1999).

25. Pavoni, D.P., Roxo, V.M.M.S., Marquart Filho, A. & Petzl-Erler, M.L. Dissecting the associations of endemic Pemphigus Foliaceus (Fogo Selvagem) with HLA-DRB1 alleles and genotypes. *Genes & Immunity* **4**, 110-116 (2003).

26. Thomas, G.J.J., Conejo-Mir, J.S., Escribano, F.G., Bernal, A.M.P. & Roldán, A.N. Estudio de los alelos de HLA de clase II que confieren susceptibilidad al pénfigo vulgar en una población andaluza. *ACTAS Dermo-Sifiliograficas* **89**, 531-538 (1998).

27. Zhang, S.Y. *et al.* Subtype-specific inherited predisposition to pemphigus in the Chinese population. *British Journal of Dermatology* **180**, 828-835 (2019).

28. Moraes, J.R. *et al.* HLA antigens and risk for development of pemphigus foliaceus (fogo selvagem) in endemic areas of Brazil. *Immunogenetics* **33**, 388-391 (1991).

29. Lee, C.W., Yang, H.Y., Kim, S.C., Jung, J.H. & Hwang, J.J. HLA Class II Allele Associations in Korean Patients with Pemphigus. *Dermatology* **197**, 349-352 (1998).

30. Shams, S. *et al.* HLA Class II (DRB, DQA1 and DQB1) Allele and Haplotype Frequencies in the Patients with Pemphigus Vulgaris. *Journal of Clinical Immunology* **29**, 175-179 (2009).

31. Orouji, E., Tavakkol Afshari, J., Schmieder, A. & Layegh, P. HLA-DQB1 gene and pemphigus vulgaris in patients with Mid-East origin. *Journal of Dermatological Science* **76**, 153-155 (2014).

32. Miyagawa, S. *et al.* HLA-DRB1*04 and DRB1*14 alleles are associated with susceptibility to pemphigus among Japanese. *J Invest Dermatol* **109**, 615-618 (1997).

33. Joly, B.S. *et al.* HLA-DRB1*11 is a strong risk factor for acquired thrombotic thrombocytopenic purpura in children. *Haematologica* (2020).

34. Glorio, R. *et al.* HLA Haplotypes and Class II Molecular Alleles in Argentinian Patients with Pemphigus Vulgaris. *Journal of Cutaneous Medicine and Surgery: Incorporating Medical and Surgical Dermatology* **6**, 422-426 (2002).

35. Sakai, K. *et al.* HLA loci predisposing to immune TTP in Japanese: potential role of the shared ADAMTS13 peptide bound to different HLA-DR. *Blood* **135**, 2413-2419 (2020).

36. Koc, C.K., Sallakci, N., Akman-Karakaş, A., Alpsoy, E. & Yegin, O. Human leukocyte antigens class I and class II in patients with pemphigus in southern Turkey. *International Journal of Dermatology* **52**, 53-58 (2013).

37. Scully, M. *et al.* Human leukocyte antigen association in idiopathic thrombotic thrombocytopenic purpura: evidence for an immunogenetic link. *Journal of Thrombosis and Haemostasis* **8**, 257-262 (2010).

38. Mobini, N. *et al.* Identical MHC Markers in Non-Jewish Iranian and Ashkenazi Jewish Patients with Pemphigus Vulgaris: Possible Common Central Asian Ancestral Origin. *Human Immunology* **57**, 62-67 (1997).

39. Delgado, J.C. *et al.* Pemphigus Vulgaris Autoantibody Response is Linked to HLA-DQB10503 in Pakistani Patients. *Human Immunology* **57**, 110-119 (1997).

40. Yamashina, Y. *et al.* Polymorphisms of HLA class II genes in Japanese patients with pemphigus vulgaris. *Tissue Antigens* **52**, 74-77 (1998).

41. Al Haddad, C., Finianos, P., Zgheib, E., Germanos, M. & Coppo, P. Risk factors associated with the human leucocyte antigen system in Lebanese patients with immune-mediated thrombotic thrombocytopenic purpura. *La Presse Médicale* **48**, 1182-1184 (2019).

42. John, M.-L., Hitzler, W. & Scharrer, I. The role of human leukocyte antigens as predisposing and/or protective factors in patients with idiopathic thrombotic thrombocytopenic purpura. *Annals of Hematology* **91**, 507-510 (2012).

43. Martino, S. *et al.* Thrombotic Thrombocytopenic Purpura in Black People: Impact of Ethnicity on Survival and Genetic Risk Factors. *PLoS One* **11**, e0156679-e0156679 (2016).

44. Cerna, M. *et al.* Genetic markers for susceptibility to endemic Brazilian pemphigus foliaceus (Fogo Selvagem) in Xavante Indians. *Tissue Antigens* **42**, 138-140 (1993).

45. Birol, A., Anadolu, R.Y., Tutkak, H. & Gürgey, E. HLA-class 1 and class 2 antigens in Turkish patients with pemphigus. *International Journal of Dermatology* **41**, 79-83 (2002).

46. Rangel-Gamboa, L., Vega-Memije, M.E., Acuña-Alonzo, V. & Granados-Arriola, J. [HLA class II in Mexican patients with pemphigus vulgaris: shared epitope for autoimmunity]. *Gac Med Mex* **152**, 587-591 (2016).

47. Khan, S.W., Iftikhar, N., Ahmed, T.A. & Bashir, M. HLA- DR Alleles in Pakistani Patients of Pemphigus Vulgaris. *J Coll Physicians Surg Pak* **25**, 233-236 (2015).

48. Carcassi, C. *et al.* HLA haplotypes and class II molecular alleles in Sardinian and Italian patients with pemphigus vulgaris. *Tissue Antigens* **48**, 662-667 (1996).

49. Lombardi, M.L. *et al.* Molecular analysis of HLA DRB1 and DQB1 in Italian patients with pemphigus vulgaris. *Tissue Antigens* **47**, 228-230 (1996).

50. Saha, M. *et al.* Pemphigus Vulgaris in White Europeans Is Linked with HLA Class II Allele HLA DRB1*1454 but Not DRB1*1401. *Journal of Investigative Dermatology* **130**, 311-314 (2010).

51. Niks, E.H. *et al.* Strong association of MuSK antibody–positive myasthenia gravis and HLA-DR14-DQ5. *Neurology* **66**, 1772 (2006).

52. Sinkovits, G. *et al.* The role of human leukocyte antigen DRB1-DQB1 haplotypes in the susceptibility to acquired idiopathic thrombotic thrombocytopenic purpura. *Human Immunology* **78**, 80-87 (2017).
